# Towards *trans*-dual deuterated cyclopropanes *via* photoredox synergistic deuteration with D_2_O†

**DOI:** 10.1039/d5sc00350d

**Published:** 2025-04-22

**Authors:** Yuanqing Wu, Chuxiong Peng, Qichen Zhan, Xudong Lou, Shijie Liu, Xiaofeng Lin, Yulin Han, Peng Cao, Tao Cao

**Affiliations:** a State Key Laboratory of Technologies for Chinese Medicine Pharmaceutical Process Control and Intelligent Manufacture, School of Pharmacy, Nanjing University of Chinese Medicine Nanjing Jiangsu 210023 China cao_peng@njucm.edu.cn caot@njucm.edu.cn; b The Quzhou Affiliated Hospital of Wenzhou Medical University, Quzhou People's Hospital Quzhou Zhejiang 324000 China; c Jiangsu Provincial Medical Innovation Center, Affiliated Hospital of Integrated Traditional Chinese and Western Medicine, Nanjing University of Chinese Medicine Nanjing Jiangsu 210028 China; d Gaoyou Hospital of Traditional Chinese Medicine Yangzhou Jiangsu 225600 China; e Key Laboratory of Pollution Exposure and Health Intervention of Zhejiang Province, Interdisciplinary Research Academy, Zhejiang Shuren University Hangzhou Zhejiang 310015 China yulin.han@zjsru.edu.cn

## Abstract

As the demand for deuterated compounds continues to rise in medicinal chemistry, various methods have been developed to incorporate deuterium atoms. Among these, achieving consecutive *trans*-dual deuteration remains a challenging task. We have designed a novel strategy to synthesize *trans*-dual deuterated cyclopropanes at adjacent carbon positions. This approach involves H/D exchange followed by a photocatalyzed deuteroaminomethylation of cyclopropenes, with deuterium oxide serving as the sole deuterium source. The reaction is carried out under mild conditions and exhibits a broad substrate scope, high diastereoselectivity, and promising potential for further applications, making it an attractive transformation for future studies.

## Abstract 2

As the demand for deuterated compounds continues to rise in medicinal chemistry, various methods have been developed to incorporate deuterium atoms. We report here a novel synergistic method for the synthesis of *trans*-dual deuterated cyclopropanes.

## Graphical abstract

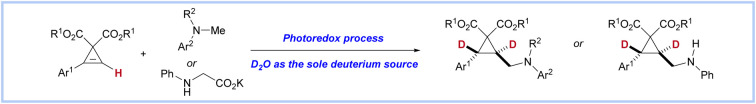

Due to the pronounced H/D isotope effect, deuterated compounds often exhibit significantly different thermodynamic and kinetic properties compared to their non-deuterated counterparts.^1^ Substituting hydrogen atoms with deuterium in biologically active compounds can alter their pharmacological profiles, offering a straightforward yet effective approach to improving pharmacokinetics and metabolic stability (Fig. 1a).^2^ Since the FDA's approval of deutetrabenazine in 2017 (ref. 3)—the first deuterated drug, used to treat Huntington's chorea and tardive dyskinesia—an increasing number of deuterated drugs have been developed for various diseases.^4^ Examples include donafenib (for non-small-cell lung cancer),^5^ deucravacitinib (for psoriasis and other autoimmune disorders),^6^ and VV-116 (for COVID-19 (ref. 7) and RSV^8^).

**Fig. 1:**
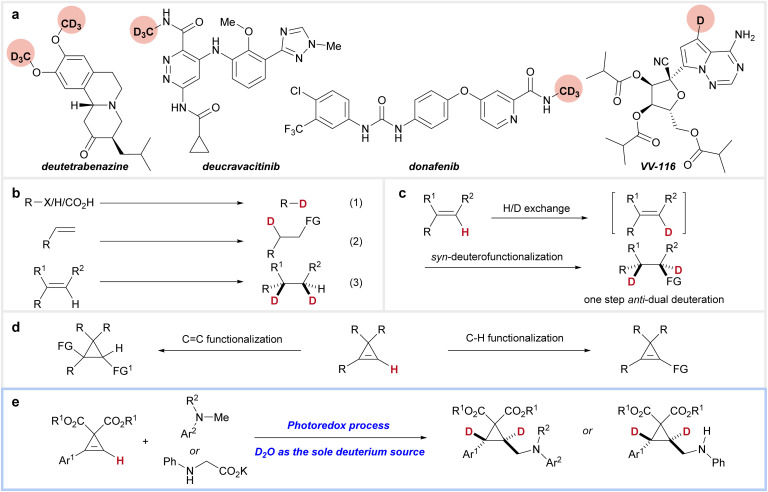
Background of this study. (a) Examples of deuterated drugs. (b) Reported strategies for synthesizing deuterated compounds. (c) Our strategy for *trans*-dual deuteration. (d) Functionalization pathways of cyclopropenes. (e) This work for the synthesis of *trans*-dual deuterated cyclopropanes.

Given the significance of deuterated compounds, strategies for their synthesis have garnered considerable attention from synthetic chemists.^9^ Approaches such as dehalogenative deuteration,^10^ H/D exchange (hydrogen isotope exchange),^11^ and decarboxylative deuteration^12^ offer straightforward methods for introducing a deuterium atom (Fig. 1b, eqn (1)). An alternative strategy involves the deuterofunctionalization of alkenes, a versatile method for synthesizing functionalized aliphatic chains (Fig. 1b, eqn (2)).^13^ While these methods typically incorporate only one deuterium atom into the products, reductive deuteration of alkenes provides a direct route to dual-deuterated compounds at adjacent carbon atoms with either *syn*-selectivity or a lack of stereoselectivity (Fig. 1b, eqn (3)).^11*e*,14^ However, to the best of our knowledge, highly selective *anti*-dual deuteration of alkenes remains a significant challenge.

We envisioned that a synergistic combination of H/D exchange at a reactive C–H bond in alkenes and *syn*-deuterofunctionalization could enable dual deuteration at consecutive carbons, yielding *trans*-selective dual deuteration that is unattainable by existing methods (Fig. 1c). This approach provides highly regio- and stereoselective dual deuteration while avoiding scrambling to undesired positions. Due to their strained structures, readily available cyclopropenes exhibit high reactivity toward both transition-metal-catalyzed and radical-mediated additions (Fig. 1d).^15^ Additionally, the alkenyl C–H bond in cyclopropenes is relatively reactive, making them excellent substrates for our strategy.^16^ Herein, we report our recent results, achieving the synthesis of *trans*-dual deuterated cyclopropanes *via* synergistic H/D exchange-photocatalyzed deuteroaminomethylation of cyclopropenes using deuterium oxide as the sole deuterium source (Fig. 1e).

Cyclopropene S1 was initially selected as the starting material to react with diphenylmethylamine (S2) under the catalysis of 4CzIPN and blue LED irradiation. Using potassium carbonate as the base and acetonitrile as the solvent, the addition product (1) was obtained in 40% yield (Table 1, entry 1). Optimization of the reaction conditions revealed that K_2_HPO_4_ and NMP were more effective as the base and solvent, respectively (Table 1, entries 2 and 3). Interestingly, the addition of a small amount of water slightly improved the reaction yield (Table 1, entry 4). Inspired by the beneficial effect of water, we hypothesized that replacing it with deuterium oxide (D_2_O) might yield deuterated products. Indeed, dual deuteration of 1 was observed, but the non-benzylic position exhibited only 44% deuterium incorporation (Table 1, entry 5). We attributed this low incorporation to the hydrogen atoms in K_2_HPO_4_. Consequently, we screened other hydrogen-free bases and found that potassium carbonate significantly improved deuterium incorporation (Table 1, entries 6–8). With potassium carbonate as the base and increasing the D_2_O content to 10%, we achieved 94% and 95% deuterium incorporation at the benzylic and non-benzylic positions, respectively, with a yield of 77% (Table 1, entry 9). However, increasing the D_2_O content further to 15% did not enhance deuterium incorporation and led to a significant decrease in yield (Table 1, entry 10). The reaction was completely suppressed when conducted under an air atmosphere or using pure D_2_O as the solvent (Table 1, entries 11 and 12). Alternative photocatalysts such as eosin Y and g-C_3_N_4_ proved ineffective (Table 1, entries 13 and 14). However, when Ir(ppy)_2_(dtbbpy)PF_6_ was used as the photocatalyst, the reaction proceeded smoothly, albeit with a slightly decreased yield of 1 and lower deuteration at the D^2^ position (Table 1, entry 15).

**Table 1:** Optimization of the reaction conditions^a^

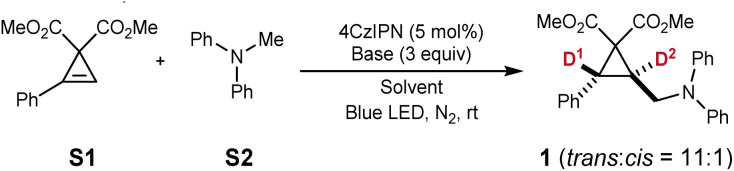					
Entry	Base	Solvent	Yield of 1^b^ (%)	D^1^ (%)	D^2^ (%)
1	K_2_CO_3_	CH_3_CN	40	N.D.^c^	
2	K_2_HPO_4_	CH_3_CN	66	N.D.	
3	K_2_HPO_4_	NMP	79	N.D.	
4	K_2_HPO_4_	NMP : H_2_O (95 : 5)	85	N.D.	
5	K_2_HPO_4_	NMP : D_2_O (95 : 5)	86	87	44
6	K_2_CO_3_	NMP : D_2_O (95 : 5)	83	88	86
7	Cs_2_CO_3_	NMP : D_2_O (95 : 5)	79	85	86
8	K_3_PO_4_	NMP : D_2_O (95 : 5)	76	84	85
**9**	**K**_**2**_ **CO**_**3**_	**NMP : D**_**2**_ **O (90 : 10)**	**77**	**94**	**95**
10	K_2_CO_3_	NMP : D_2_O (85 : 15)	61	95	95
11^d^	K_2_CO_3_	NMP : D_2_O (90 : 10)	0	N.D.	
12	K_2_CO_3_	D_2_O	0	N.D.	
13^e^	K_2_CO_3_	NMP : D_2_O (90 : 10)	0	N.D.	
14^f^	K_2_CO_3_	NMP : D_2_O (90 : 10)	0	N.D.	
**15^g^**	**K**_**2**_ **CO**_**3**_	**NMP : D**_**2**_ **O (90 : 10)**	**75**	**96**	**93**

a Reaction conditions: S1 (0.1 mmol), S2 (0.3 mmol), photocatalyst (0.005 mmol), and base (0.3 mmol) in solvent (1 mL) irradiated with blue LEDs at room temperature overnight.

b Isolated yield.

c Not detected.

d Under an air atmosphere.

e Eosin Y (5 mol%) as the photocatalyst.

f g-C_3_N_4_ (5 mg) as the photocatalyst.

g Ir(ppy)_2_(dtbbpy)PF_6_ (1 mol%) as the photocatalyst.

With the optimized conditions in hand, we investigated the scope of this reaction (Fig. 2). For phenyl-substituted cyclopropenes reacting with diphenylmethylamine, halogen substituents such as bromine (2) and fluorine (3) were well tolerated, and chlorine atoms at the *para*- (4), *meta*- (5), and *ortho*-positions (6) did not affect the reaction. Other electron-withdrawing groups, including trifluoromethyl (7) and ester groups (8), were also compatible with this transformation. Cyclopropenes bearing electron-donating substituents produced the corresponding dual-deuterated cyclopropanes efficiently (9–11). Additionally, substrates featuring ethyl, *tert*-butyl, and benzyl ester groups were suitable for this reaction (12–14). It should be noted that when 3 mmol of S1 was used, the yield remained unaffected, allowing for a gram-scale synthesis of 1.

**Fig. 2:**
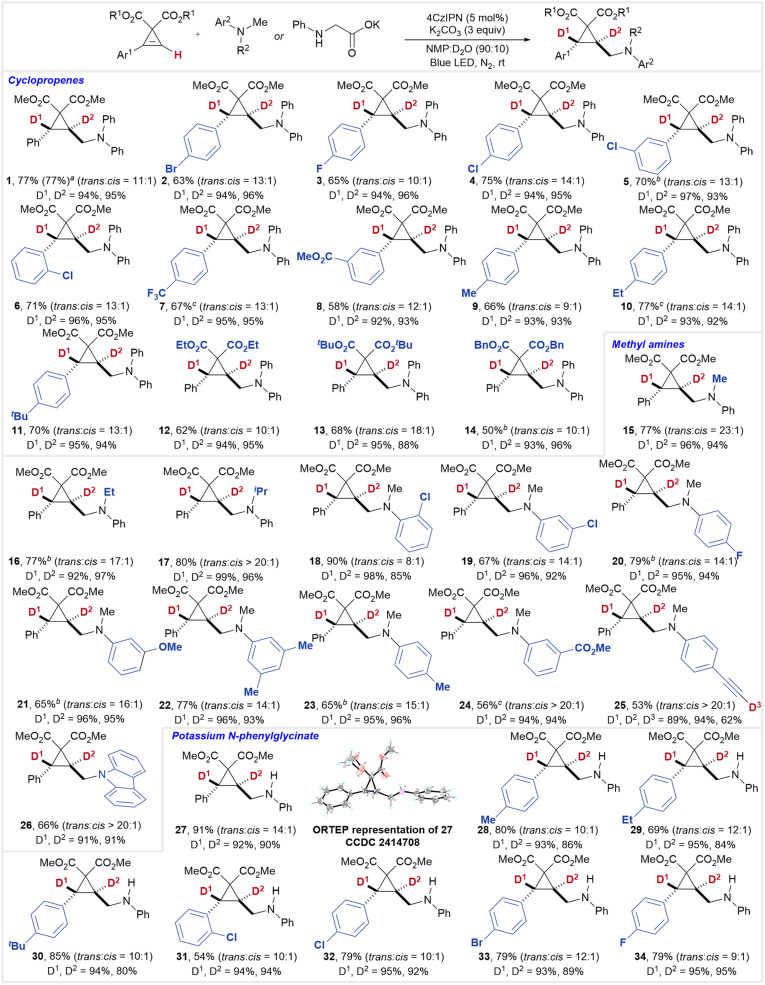
Substrate scope for the synthesis of *trans*-dual deuterated cyclopropanes. Reaction conditions: cyclopropene (0.1 mmol), amine (0.3 mmol), 4CzIPN (0.005 mmol), and K_2_CO_3_ (0.3 mmol) in a mixture of NMP and D_2_O (90 : 10, 1 mL) irradiated with blue LEDs at room temperature overnight. ^*a*^Reaction on a 3 mmol scale to afford 0.96 g of 1. ^*b*^Ir(ppy)_2_(dtbbpy)PF_6_ (1 mol%) was used. ^*c*^4CzIPN (10 mol%) was used.

Beyond diphenylmethylamine, other tertiary amines such as phenyldimethylamine (15), phenylethylmethylamine (16), and phenylisopropylmethylamine (17) also performed well under these conditions. Substituted phenyldimethylamines were further explored, with substrates bearing chlorine atoms at the *ortho*- and *meta*-positions (18 and 19) or a fluorine atom at the *para*-position (20) showing no adverse effects. Substrates with electron-donating groups such as methoxy (21), dimethyl (22), and methyl (23) groups, as well as electron-withdrawing ester groups (24), were also tolerated. Notably, an ethynyl group (25) on the substrate retained the C–C triple bond, demonstrating the higher reactivity of the cyclopropene moiety compared to the alkyne moiety. Notably, the alkynyl C–H bond was deuterated at 68% during the reaction. Additionally, a carbazole derivative (26) was successfully synthesized, further highlighting the versatility of this transformation.

Interestingly, under the standard conditions, potassium phenylglycinate was also found to be suitable for the *anti*-selective dual deuteration reaction *via* a photoredox decarboxylation process (27).^12,17^ This approach provides dual-deuterated cyclopropanes bearing a secondary aminomethyl substituent. Furthermore, cyclopropenes bearing alkyl groups (28–30) or halogen atoms (31–34) were successfully employed in this process, affording the corresponding products in good yields. The *trans*-selectivity of this transformation was unambiguously confirmed by the single-crystal X-ray diffraction analysis of compound 27.

When benzylmethylphenylamine S3 was used, product 35 was obtained smoothly. However, the deuteration content at D^1^ was only 65%, while the benzyl position (D^3^) exhibited 43% deuteration (eqn (4)). This outcome was attributed to the scrambling of reactive benzylic hydrogens. Nevertheless, substrates S4, S5, and S6 did not undergo the desired reaction (eqn (5)).
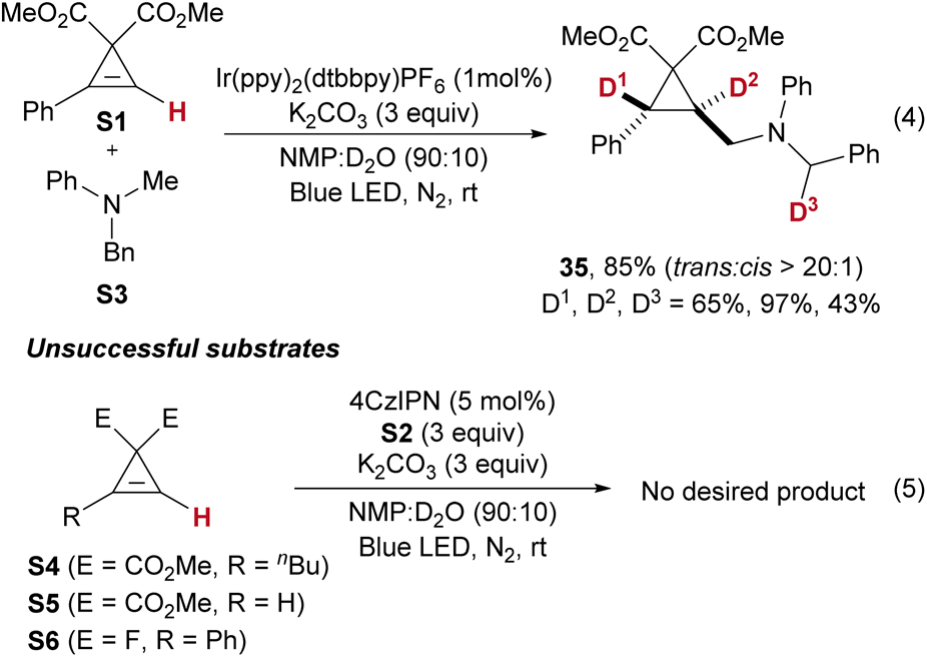


The applicability of this transformation was demonstrated through the modification of functional molecules (Fig. 3a and b). By introducing a phenylmethylamino group, several biologically active moieties were incorporated into the reaction, expanding its potential applications (Fig. 3a). Consequently, products derived from ibuprofen (36), gemfibrozil (37), adamantane (38), and dihydrocholesterol (39) were successfully synthesized. Furthermore, hydrolysis of compound 1 yielded the diacid intermediate 1-diacid, which subsequently underwent condensation with biologically active alcohols to afford compounds 40 and 41 (Fig. 3b). These transformations enabled the installation of a dual-deuterated cyclopropyl warhead.

**Fig. 3:**
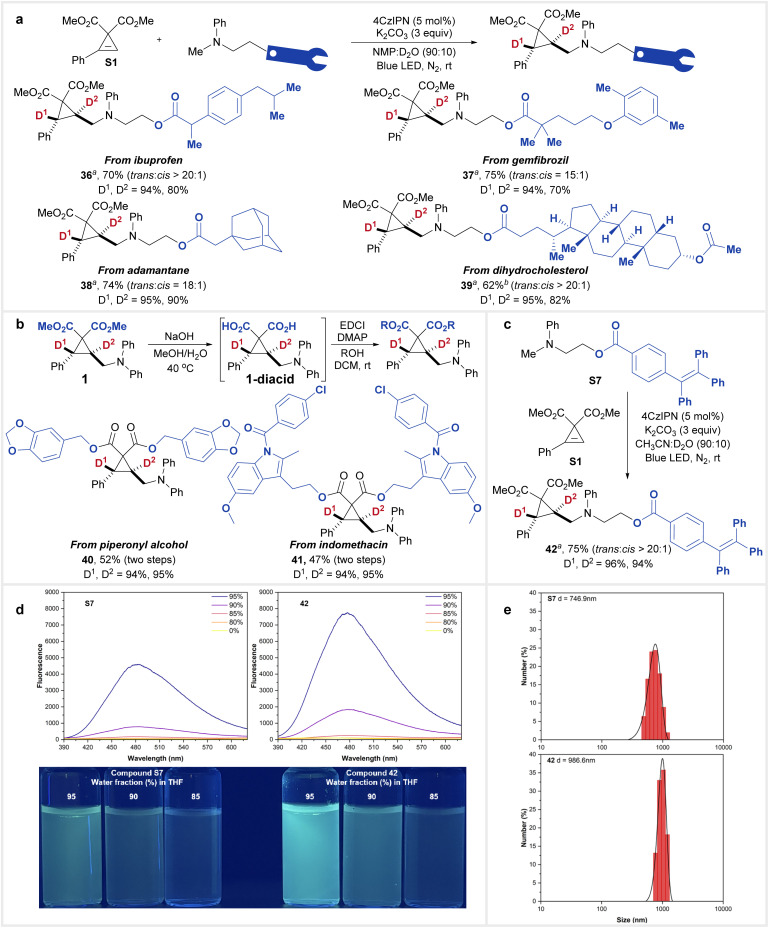
Synthetic application of this reaction. (a) Modification of complex molecules. (b) Transformation of 1 into compounds 40 and 41. (c) Synthesis of compound 42. (d) AIE properties of S7 and 42. (e) DLS analysis of S7 and 42. ^*a*^Reaction conditions: cyclopropene (0.1 mmol), amine (0.3 mmol), 4CzIPN (0.005 mmol), and K_2_CO_3_ (0.3 mmol) in a mixture of NMP and D_2_O (90 : 10, 1 mL) irradiated with blue LEDs at room temperature overnight. ^*b*^4CzIPN (10 mol%) was used.

Additionally, compound 42, incorporating a tetraphenylethylene moiety, was synthesized *via* the reaction of S1 with S7 under standard conditions (Fig. 3c). Given the widespread use of tetraphenylethylene in constructing materials with aggregation-induced emission (AIE) properties,^18^ we compared the AIE behavior of S7 and 42 (Fig. 3d). Notably, under identical conditions, 42 exhibited a stronger AIE effect than its precursor S7. The aggregation of both compounds was confirmed by DLS (Fig. 3e) and TEM (Fig. S1 in the ESI†) analyses, revealing that the average size of aggregated 42 (986.6 nm) was larger than that of S7 (746.9 nm). This increased aggregation size may contribute to the enhanced AIE effect, as larger aggregates often lead to a more restricted molecular environment. Additionally, we hypothesize that the rigid cyclopropane core in 42 reduces molecular flexibility and restricts intramolecular rotation or vibration in the aggregated state. This enhanced rigidity likely suppresses non-radiative decay pathways, which typically quench emission, thereby further amplifying the AIE effect compared to the more flexible S7.^19^

To investigate the possible mechanism of this reaction, several control experiments were conducted. The addition of TEMPO under standard conditions completely suppressed the reaction; instead, the adduct product S2-TEMPO was detected by HRMS, indicating that the transformation proceeds *via* a radical mechanism (Fig. 4a). In a separate experiment, the reaction of S1 with potassium carbonate in the absence of S2 and without blue LED irradiation led to the formation of S1-D, with deuteration occurring at the olefinic hydrogen at 96% (Fig. 4b). This result demonstrates that the acidic olefinic hydrogen can undergo H/D exchange during the reaction.^16*a*^ A light on/off experiment (Fig. 4c), along with a measured quantum yield of 0.30 (see the ESI† for details), ruled out the possibility of a chain process in this reaction. Furthermore, Stern–Volmer quenching experiments were performed. As shown in Fig. 4d, the fluorescence of the photocatalyst 4CzIPN was quenched by the addition of various concentrations of S2, while no quenching was observed upon the addition of S1. These results suggest that electron transfer occurs between the photocatalyst 4CzIPN and S2.

**Fig. 4:**
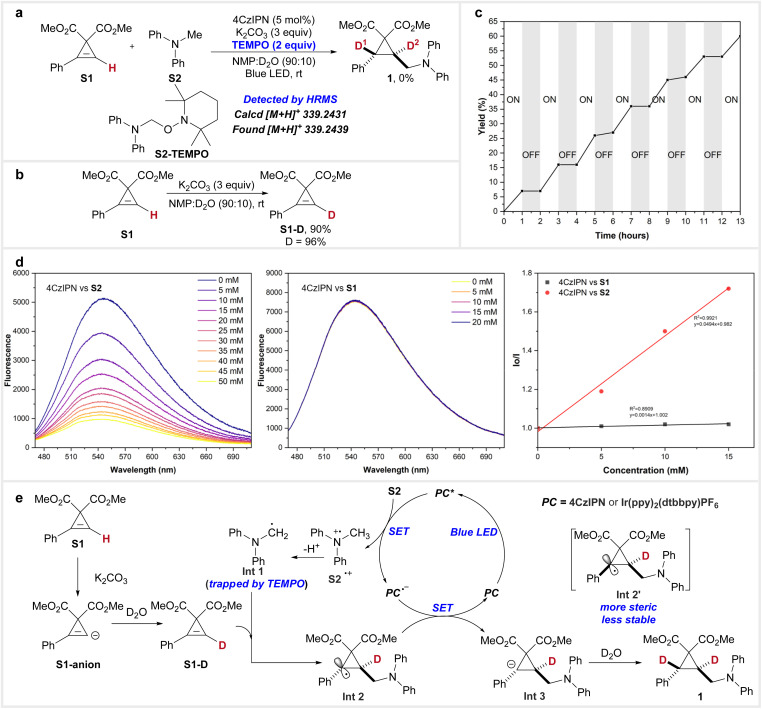
Mechanistic studies. (a) Trap of the radical intermediate by TEMPO. (b) Deuteration of S1. (c) A light on/off experiment. (d) Stern–Volmer quenching experiments. (e) A proposed mechanism.

Based on the above results, a plausible mechanism has been proposed (Fig. 4e). Potassium carbonate deprotonates the olefinic hydrogen of S1, generating the S1-anion, which is then deuterated by D_2_O to form S1-D. Concurrently, upon irradiation, the photocatalyst (PC) is activated to its excited state (PC*), where it undergoes a single electron transfer (SET) process with S2, followed by deprotonation, generating intermediate Int 1 and a radical anion (PC˙^−^). Int 1 then undergoes a radical addition to S1-D, forming a more thermodynamically stable *trans*-cyclopropyl radical intermediate (Int 2*vs.*Int 2′). Int 2 reacts with PC˙^−^*via* a SET process to yield the cyclopropyl anion intermediate (Int 3) and regenerate the ground-state PC. Finally, deuteration of Int 3 leads to the formation of product 1, with the thermodynamically favoured *trans*-selective product being formed during the process.

## Conclusions

In conclusion, we have developed a *trans*-selective dual deuteration strategy for cyclopropenes by combining H/D exchange and *syn*-deuterofunctionalization. The mild reaction conditions, broad substrate scope, high diastereoselectivity, and significant deuterium incorporation make this transformation highly attractive. Post-modification of biologically active molecules and the synthesis of an AIE compound further highlight its potential applications in related fields. Ongoing work in this laboratory focuses on extending this strategy to other unsaturated compounds and conducting more detailed mechanistic studies.

## Data availability

All experimental data associated with this work are available in the ESI.†

## Author contributions

Y. W., C. P., and Q. Z.: investigation, methodology, and writing – review and editing. X. Lou, S. L., and X. Lin: investigation and methodology. Y. H.: investigation, methodology, and project administration. P. C.: project administration, funding acquisition, and writing – review and editing. T. C.: project administration, funding acquisition, and writing – original draft.

## Conflicts of interest

There are no conflicts to declare.

## Supplementary Material

SC-016-D5SC00350D-s001

SC-016-D5SC00350D-s002

## References

[cit1] Yang J. , Deuterium: Discovery and Applications in Organic Chemistry, Elsevier, Amsterdam, 2016

[cit2] Atzrodt J., Derdau V., Kerr W. J., Reid M. (2018). Angew. Chem., Int. Ed..

[cit3] Heo Y.-A., Scott L. J. (2017). Drugs.

[cit4] Di Martino R. M. C., Maxwell B. D., Pirali T. (2023). Nat. Rev. Drug Discovery.

[cit5] Keam S. J., Duggan S. (2021). Drugs.

[cit6] Wrobleski S. T., Moslin R., Lin S., Zhang Y., Spergel S., Kempson J., Tokarski J. S., Strnad J., Zupa-Fernandez A., Cheng L., Shuster D., Gillooly K., Yang X., Heimrich E., McIntyre K. W., Chaudhry C., Khan J., Ruzanov M., Tredup J., Mulligan D., Xie D., Sun H., Huang C., D'Arienzo C., Aranibar N., Chiney M., Chimalakonda A., Pitts W. J., Lombardo L., Carter P. H., Burke J. R., Weinstein D. S. (2019). J. Med. Chem..

[cit7] Qian H.-j., Wang Y., Zhang M.-q., Xie Y.-c., Wu Q.-q., Liang L.-y., Cao Y., Duan H.-q., Tian G.-h., Ma J., Zhang Z.-b., Li N., Jia J.-y., Zhang J., Aisa H. A., Shen J.-s., Yu C., Jiang H.-l., Zhang W.-h., Wang Z., Liu G.-y. (2022). Acta Pharmacol. Sin..

[cit8] Zhang R., Zhang Y., Zheng W., Shang W., Wu Y., Li N., Xiong J., Jiang H., Shen J., Xiao G., Xie Y., Zhang L. (2022). Signal Transduction Targeted Ther..

[cit9] Prakash G., Paul N., Oliver G. A., Werz D. B., Maiti D. (2022). Chem. Soc. Rev..

[cit10] Li P., Guo C., Wang S., Ma D., Feng T., Wang Y., Qiu Y. (2022). Nat. Commun..

[cit11] Teja C., Kolb S., Colonna P., Grover J., Garcia-Argote S., Lahiri G. K., Pieters G., Werz D. B., Maiti D. (2024). Angew. Chem., Int. Ed..

[cit12] Li N., Ning Y., Wu X., Xie J., Li W., Zhu C. (2021). Chem. Sci..

[cit13] Guo J., Cheng Z., Chen J., Chen X., Lu Z. (2021). Acc. Chem. Res..

[cit14] Li H., Zhang B., Dong Y., Liu T., Zhang Y., Nie H., Yang R., Ma X., Ling Y., An J. (2017). Tetrahedron Lett..

[cit15] Cohen Y., Cohen A., Marek I. (2020). Chem. Rev..

[cit16] Liu K., Li T., Liu D.-Y., Li W., Han J., Zhu C., Xie J. (2021). Sci. China:Chem..

[cit17] Xuan J., Zhang Z. G., Xiao W. J. (2015). Angew. Chem., Int. Ed..

[cit18] Hong Y., Lam J. W. Y., Tang B. Z. (2011). Chem. Soc. Rev..

[cit19] Mei J., Leung N. L. C., Kwok R. T. K., Lam J. W.
Y., Tang B. Z. (2015). Chem. Rev..

