# Supplementary material for: Towards trans-dual deuterated cyclopropanes via photoredox synergistic deuteration with D2O
Source: Chem Sci. 2025 Apr 22;16(21):9535–42. doi: 10.1039/d5sc00350d (PMC12042209; doi:10.1039/d5sc00350d)
Supplement: SC-016-D5SC00350D-s001 [file SC-016-D5SC00350D-s001.pdf]

Supplementary Information (SI) for Chemical Science.
This journal is © The Royal Society of Chemistry 2025

Supplementary Information

Towards *trans*-Dual Deuterated Cyclopropanes via Photoredox Synergistic Deuteration with D_2O

Yuanqing Wu,[1,2]† Chuxiong Peng,[1,2]† Qichen Zhan,[1,2]† Xudong Lou,[1,2] Shijie Liu,[1,2] Xiaofeng Lin,[1,2] Yulin Han,[6]* Peng Cao,[1,3,4,5]* Tao Cao[1,2]*

1 State Key Laboratory on Technologies for Chinese Medicine Pharmaceutical Process Control and Intelligent Manufacture, Nanjing University of Chinese Medicine, Nanjing, Jiangsu 210023, China.

2 School of Pharmacy, Nanjing University of Chinese Medicine, Nanjing, Jiangsu 210023, China.

3 Jiangsu Provincial Medical Innovation Center, Affiliated Hospital of Integrated Traditional Chinese and Western Medicine, Nanjing University of Chinese Medicine, Nanjing, Jiangsu 210028, China.

4 The Quzhou Affiliated Hospital of Wenzhou Medical University, Quzhou People's Hospital, Quzhou, Zhejiang 324000, China.

5 Gaoyou Hospital of Traditional Chinese Medicine, Yangzhou, Jiangsu 225600, China.

6 Key Laboratory of Pollution Exposure and Health Intervention of Zhejiang Province, Interdisciplinary Research Academy, Zhejiang Shuren University, Hangzhou, Zhejiang 310015, China.

† These authors contributed equally to this work.

E-mail: yulin.han@zjsru.edu.cn; cao_peng@njucm.edu.cn; caot@njucm.edu.cn

Table of Contents

1. General Information. S3

2. Synthesis of *trans*-Dual Deuterated Cyclopropanes. S3

3. Synthetic applications. S32

4. Mechanistic investigations. S36

5. References. S41

6. ^{1}H NMR, ^{13}C NMR, ^{19}F NMR spectra for compounds. S42

1. General Information.

All the temperatures are referred to the preheated oil baths used. ^{1}H NMR spectra were recorded on a Bruker AVANCE III 500 or JNM-ECZ400S/L1 spectrometer in $CDCl_3$ (tetramethylsilane as an internal standard). The data are reported as follows: chemical shift (δ ppm), multiplicity (s = singlet, d = doublet, t = triplet, q = quartet, m = multiplet, dd = doublet of doublets, dt = doublet of triplets, td = triplet of doublets, tt = triplet of triplets), coupling constant(s) in Hz, integration. ^{13}C NMR spectra was recorded on a Bruker AVANCE III 500 or JNM-ECZ400S/L1 spectrometer and data are reported in terms of chemical shift relative to $CDCl_3$ (77.00 ppm). In a typical experiment, blue LEDs (450-465 nm) with a cooling fan were used as a visible light source. Commercially available reagents and solvents were purchased from Adamas, Sinopharm, and Sigma-Aldrich. Cyclopropenes were synthesized according to literatures.[1-6]

2. Synthesis of *trans*-Dual Deuterated Cyclopropanes.

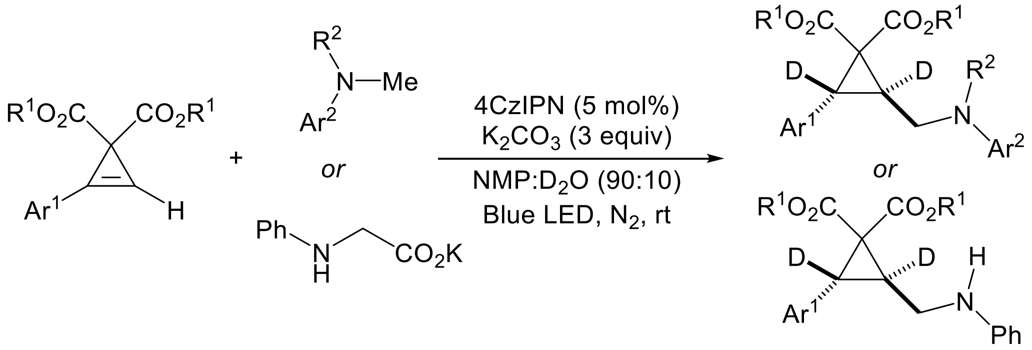

General Procedure I: Under nitrogen atmosphere, to a 10 mL Schlenk tube equipped with a stirring bar is added 4CzIPN (5 mol%), K_2CO_3 (0.3 mmol), cyclopropene (0.1 mmol), tertiary methylamine or potassium *N*-phenyl glycinate (0.3 mmol), *N*-methyl pyrrolidinone (0.9 mL), and D_2O (0.1 mL) successively, and the tube was sealed with a septum. The mixture was stirred at room temperature irradiated with a blue LED (6 W) overnight. The reaction was complete as monitored by TLC, and quenched with H_2O (2.5 mL). The aqueous layer was extracted with ethyl acetate (5

mL × 3) and the combined organic layer was washed with brine (5 mL), dried over anhydrous $MgSO_4$, filtered and concentrated in vacuo to afford corresponding product via chromatography on silica gel.

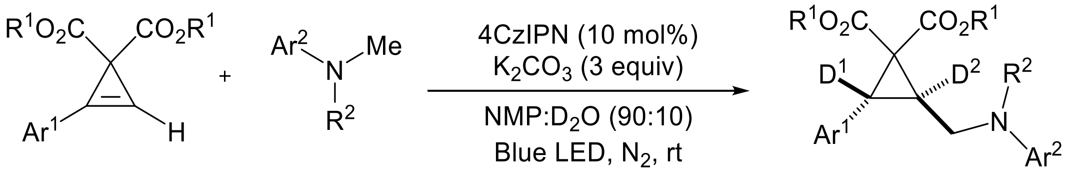

General Procedure II: Under nitrogen atmosphere, to a 10 mL Schlenk tube equipped with a stirring bar is added 4CzIPN (10 mol%), K_2CO_3 (0.3 mmol), cyclopropene (0.1 mmol), methylamine (0.3 mmol), *N*-methyl pyrrolidinone (0.9 mL), and D_2O (0.1 mL) successively, and the tube was sealed with a septum. The mixture was stirred at room temperature irradiated with a blue LED (6 W) overnight. The reaction was complete as monitored by TLC, and quenched with H_2O (2.5 mL). The aqueous layer was extracted with ethyl acetate (5 mL × 3) and the combined organic layer was washed with brine (5 mL), dried over anhydrous $MgSO_4$, filtered and concentrated in vacuo to afford corresponding product via chromatography on silica gel.

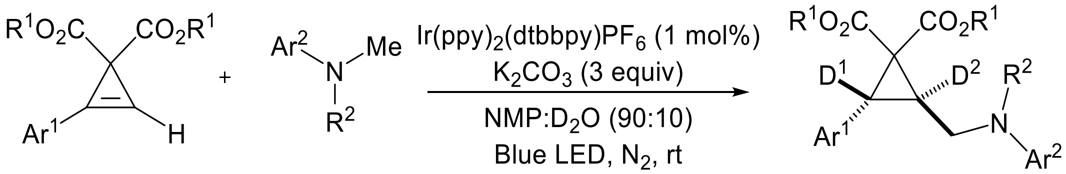

General Procedure III: Under nitrogen atmosphere, to a 10 mL Schlenk tube equipped with a stirring bar is added $Ir(ppy)_2(dtbbpy)PF_6$ (1 mol%), K_2CO_3 (0.3 mmol), cyclopropene (0.1 mmol), methylamine (0.3 mmol), *N*-methyl pyrrolidinone (0.9 mL), and D_2O (0.1 mL) successively, and the tube was sealed with a septum. The mixture was stirred at room temperature irradiated with a blue LED (6 W) overnight. The reaction was complete as monitored by TLC, and quenched with H_2O (2.5 mL). The aqueous layer was extracted with ethyl acetate (5 mL × 3) and the combined organic layer was washed with brine (5 mL), dried over anhydrous $MgSO_4$, filtered and concentrated in vacuo to afford corresponding product via chromatography on silica gel.

(1) Dimethyl (2*S,3*R**)-2-((diphenylamino)methyl)-3-phenylcyclopropane-1,1-dicarboxylate-2,3-*d*₂ (1) (wyq-3-34, wyq-5-43).**

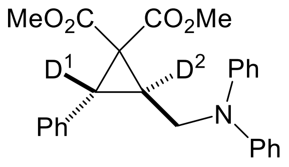

1

trans:cis = 11:1, D^1, D^2 = 94%, 95%

Following General Procedure I, **1** was afforded as a colorless oil (31.5 mg, 77%, D^1/D^2 = 94%/95%, *trans:cis* = 11:1), eluent (0-9% of ethyl acetate in petroleum ether).

The following signals are discernible for the *trans*-isomer: ^{1}H NMR (500 MHz, $CDCl_3$) δ 7.29 – 7.23 (m, 4 H), 7.20 (d, *J* = 7.4 Hz, 3 H), 7.05 (dd, *J* = 8.0, 1.4 Hz, 2 H), 7.02 (d, *J* = 7.4 Hz, 4 H), 6.96 (t, *J* = 7.3 Hz, 2 H), 3.98 (d, *J* = 15.3 Hz, 1 H), 3.88 (d, *J* = 15.2 Hz, 1 H), 3.61 (s, 3 H), 3.33 (s, 3 H), 3.17 (s, 0.06 H), 2.90 (t, *J* = 6.5 Hz, 0.05 H); ^{13}C NMR (126 MHz, $CDCl_3$) δ 168.23, 166.84, 147.65, 134.25, 129.40, 128.37, 128.12, 127.32, 121.76, 121.29, 52.76, 52.21, 49.20, 42.08, 36.35, 36.05 (t, J_{C-D} = 23.9 Hz), 29.21, 28.91 (t, J_{C-D} = 24.6 Hz); HRMS calcd for $C_{26}H_{24}D_2NO_4$ ($[M+H]^+$): 418.1982; found: 418.1988.

The following signals are discernible for the *cis*-isomer: ^{1}H NMR (500 MHz, $CDCl_3$) δ 3.75 (s, 3 H), 3.54 (s, 3 H), 4.36 (d, *J* = 15.2 Hz, 1 H); ^{13}C NMR (126 MHz, $CDCl_3$) δ 129.26, 121.51.

Gram-scale synthesis of 1 (wyq-9-28).

Under nitrogen atmosphere, to a 25 mL Schlenk tube equipped with a stirring bar is added 4CzIPN (126.8 mg, 0.15 mmol, 5 mol%), K_2CO_3 (1.2490 g, 9 mmol, 3 equiv), **S1** (696.5 mg, 3 mmol, 1 equiv), **S2** (1.6550 g, 9 mmol, 3 equiv), *N*-methyl pyrrolidinone (9 mL), and D_2O (1 mL) successively, and the tube was sealed with a septum. The mixture was stirred at room temperature irradiated with a blue LED for 48 h. The aqueous layer was extracted with ethyl acetate (10 mL × 3) and the combined organic layer was washed with brine, dried over anhydrous MgSO4, filtered and concentrated in vacuo to afford corresponding product via chromatography on silica

gel, **1** was afforded as a colorless oil (959.8 mg, 77%, D^1/D^2 = 94%/95%, *trans:cis* = 11:1), eluent (0-9% of ethyl acetate in petroleum ether).

(2) Dimethyl (2*R,3*S**)-2-(4-bromophenyl)-3-((diphenylamino)methyl) cyclopropane-1,1-dicarboxylate-2,3-*d*2 (2) (wyq-4-11).**

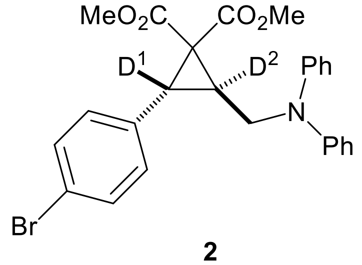

2

trans:cis = 13:1, D^1, D^2 = 94%, 96%

Following General Procedure I, **2** was afforded as a colorless oil (31.1 mg, 63%, D^1/D^2 = 94%/96%, *trans:cis* = 13:1), eluent (0-11% of ethyl acetate in petroleum ether).

The following signals are discernible for the *trans*-isomer: ^{1}H NMR (500 MHz, $CDCl_3$) δ 7.32 (d, *J* = 8.4 Hz, 2 H), 7.29 – 7.22 (m, 4 H), 7.03 – 6.93 (m, 6 H), 6.90 (d, *J* = 8.4 Hz, 2 H), 3.99 (d, *J* = 15.1 Hz, 1 H), 3.82 (d, *J* = 15.2 Hz, 1 H), 3.63 (s, 3 H), 3.38 (s, 3 H), 3.33 (s, 0.06 H), 2.83 (t, *J* = 6.5 Hz, 0.04 H); ^{13}C NMR (126 MHz, $CDCl_3$) δ 168.00, 166.66, 147.63, 133.40, 131.27, 130.12, 129.44, 121.89, 121.55, 121.33, 52.84, 52.41, 49.25, 41.99, 35.82, 35.52 (t, $J_{C\text{-}D}$ = 24.6 Hz), 29.25, 28.96 (t, $J_{C\text{-}D}$ = 23.2 Hz); HRMS calcd for $C_{26}H_{23}D_2BrNO_4$ ($[M+H]^+$): 496.1087; found: 496.1094.

The following signals are discernible for the *cis*-isomer: ^{1}H NMR (500 MHz, $CDCl_3$) δ 4.31 (d, *J* = 15.2 Hz, 2 H), 3.76 (s, 3 H), 3.56 (s, 3 H); ^{13}C NMR (126 MHz, $CDCl_3$) δ 131.34, 131.00, 129.35, 121.39, 53.03, 52.27.

(3) Dimethyl (2*S,3*R**)-2-((diphenylamino)methyl)-3-(4-fluorophenyl) cyclopropane-1,1-dicarboxylate-2,3-*d*2 (3) (wyq-4-16).**

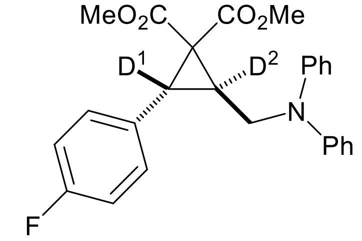

3

trans:cis = 10:1, D^1, D^2 = 94%, 96%

Following General Procedure I, **3** was afforded as a colorless oil (28.8 mg, 65%, D^1/D^2 = 94%/96%, *trans:cis* = 10:1), eluent (0-10% of ethyl acetate in petroleum ether).

The following signals are discernible for the *trans*-isomer: ^{1}H NMR (500 MHz, $CDCl_3$) δ 7.26 (m, *J* = 8.6, 7.2 Hz, 4 H), 7.04 – 6.97 (m, 6 H), 6.98 – 6.85 (m, 4 H), 3.99 (d, *J* = 15.2 Hz, 1 H), 3.83 (d, *J* = 15.2 Hz, 1 H), 3.63 (s, 3 H), 3.36 (s, 3 H), 3.12 (s, 0.06 H), 2.84 (t, *J* = 6.5 Hz, 0.04 H); ^{13}C NMR (126 MHz, $CDCl_3$) δ 168.12, 166.76, 162.08 (d, J_{C-F} = 246.1 Hz), 147.67, 130.07 (d, J_{C-F} = 8.2 Hz), 129.44, 121.87, 121.33, 115.05 (d, J_{C-F} = 21.4 Hz), 52.80, 52.30, 49.27, 41.95, 35.73, 35.43 (t, J_{C-D} = 24.6 Hz), 29.39, 29.10 (t, J_{C-D} = 25.2 Hz); ^{19}F NMR (471 MHz, $CDCl_3$) δ -114.79; HRMS calcd for $C_{26}H_{23}D_2FNO_4$ ($[M+H]^+$): 436.1888; found: 436.1897.

The following signals are discernible for the *cis*-isomer: ^{1}H NMR (500 MHz, $CDCl_3$) δ 4.31 (d, *J* = 15.2 Hz, 1 H), 3.76 (s, 3 H), 3.55 (s, 3 H); ^{13}C NMR (126 MHz, $CDCl_3$) δ 129.34, 121.94, 121.59, 52.99, 52.19.

(4) Dimethyl (2*R,3*S**)-2-(4-chlorophenyl)-3-((diphenylamino)methyl) cyclopropane-1,1-dicarboxylate-2,3-*d*$_2$ (4) (wyq-4-24).**

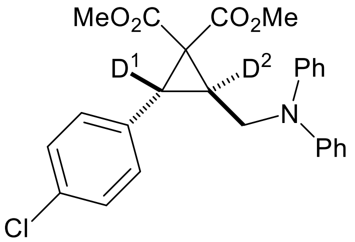

4

trans:cis = 14:1, D^1, D^2 = 94%, 95%

Following General Procedure I, **4** was afforded as a colorless oil (33.9 mg, 75%, D^1/D^2 = 94%/95%, *trans:cis* = 14:1), eluent (0-10% of ethyl acetate in petroleum ether).

The following signals are discernible for the *trans*-isomer: ^{1}H NMR (500 MHz, $CDCl_3$) δ 7.30 – 7.23 (m, 4 H), 7.20 – 7.15 (m, 2 H), 7.04 – 6.98 (m, 4 H), 7.00 – 6.94 (m, 4 H), 4.00 (d, *J* = 15.1 Hz, 1 H), 3.83 (d, *J* = 15.2 Hz, 1 H), 3.63 (s, 3 H), 3.38 (s, 3 H), 3.10 (s, 0.06 H), 2.84 (t, *J* = 6.5 Hz, 0.05 H); ^{13}C NMR (126 MHz, $CDCl_3$) δ 168.02, 166.67, 147.58, 133.25, 132.83, 129.76, 129.45, 128.32, 121.89, 121.30, 52.86, 52.41, 49.22, 42.00, 35.73, 35.43 (t, J_{C-D} = 24.6 Hz), 29.24, 28.95 (t, J_{C-D} = 24.6 Hz); HRMS calcd for $C_{26}H_{23}D_2ClNO_4$ ($[M+H]^+$): 452.1592; found: 452.1599.

The following signals are discernible for the *cis*-isomer: ^{1}H NMR (500 MHz, $CDCl_3$) δ 4.31 (d, *J* = 15.1 Hz, 1 H), 3.76 (s, 3 H), 3.56 (s, 3 H); ^{13}C NMR (126 MHz, $CDCl_3$) δ 130.65, 129.35, 121.54.

(5) Dimethyl (2*R,3*S**)-2-(3-chlorophenyl)-3-((diphenylamino)methyl) cyclopropane-1,1-dicarboxylate-2,3-*d*$_2$ (5) (wyq-9-21).**

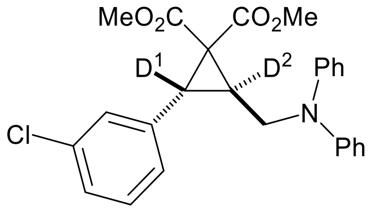

5

trans:cis = 13:1, D^1, D^2 = 97%, 93%

Following General Procedure III, **5** was afforded as a colorless oil (31.7 mg, 70%, D^1/D^2 = 97%/93%, *trans:cis* = 13:1), eluent (0-10% of ethyl acetate in petroleum ether).

The following signals are discernible for the *trans*-isomer: ^{1}H NMR (500 MHz, $CDCl_3$) δ 7.30 – 7.23 (m, 4 H), 7.14 (dt, *J* = 15.4, 8.1 Hz, 2 H), 7.04 – 6.99 (m, 5 H), 6.98 (t, *J* = 7.3 Hz, 2 H), 6.91 (d, *J* = 7.6 Hz, 1 H), 3.99 (d, *J* = 15.2 Hz, 1 H), 3.84 (d, *J* = 15.3 Hz, 1 H), 3.63 (s, 3 H), 3.40 (s, 3 H), 3.25 (s, 0.03 H), 2.83 (t, *J* = 6.5 Hz, 0.07 H); ^{13}C NMR (126 MHz, $CDCl_3$) δ 167.96, 166.61, 147.60, 136.46, 134.01, 129.46, 129.35, 128.71, 127.58, 126.47, 121.91, 121.34, 52.89, 52.40, 49.17, 41.99, 35.83, 35.52 (t, $J_{C\text{-}D}$= 24.6 Hz), 29.33, 29.04 (t, $J_{C\text{-}D}$= 24.6 Hz); HRMS calcd for $C_{26}H_{23}D_2ClNO_4$ ($[M+H]^+$): 452.1592; found: 452.1600.

The following signals are discernible for the *cis*-isomer: ^{1}H NMR (500 MHz, $CDCl_3$) δ 4.34 (d, *J* = 15.2 Hz, 1 H), 3.77 (s, 3 H), 3.58 (s, 3 H); ^{13}C NMR (126 MHz, $CDCl_3$) δ 121.52.

(6) Dimethyl (2*S,3*S**)-2-(2-chlorophenyl)-3-((diphenylamino)methyl) cyclopropane-1,1-dicarboxylate-2,3-*d*$_2$ (6) (wyq-4-29).**

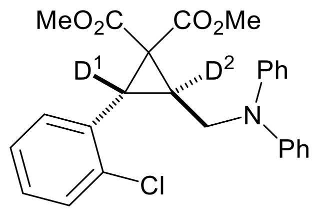

6

trans:cis = 13:1, D[1], D[2] = 96%, 95%

Following General Procedure I, **6** was afforded as a colorless oil (31.6 mg, 71%, D^1/D^2 = 96%/95%, *trans:cis* = 13:1), eluent (0-10% of ethyl acetate in petroleum ether).

The following signals are discernible for the *trans*-isomer: ^{1}H NMR (500 MHz, $CDCl_3$) δ 7.32 – 7.24 (m, 5 H), 7.15 (td, *J* = 7.7, 1.7 Hz, 1 H), 7.09 (td, *J* = 7.6, 1.4 Hz, 1 H), 7.04 – 7.01 (m, 4 H), 6.97 (t, *J* = 7.2 Hz, 2 H), 6.91 (dd, *J* = 7.7, 1.8 Hz, 1 H), 4.03 (d, *J* = 15.2 Hz, 1 H), 3.89 (d, *J* = 15.3 Hz, 1 H), 3.64 (s, 3 H), 3.35 (s, 3 H), 3.30 (s, 0.04 H), 2.94 (t, *J* = 6.5 Hz, 0.05 H); ^{13}C NMR (126 MHz, $CDCl_3$) δ 167.85, 166.82, 147.64, 136.00, 132.38, 129.44, 129.34, 129.30, 129.12, 128.69, 126.34, 122.05, 121.86, 121.74, 121.30, 52.79, 52.28, 49.37, 41.37, 35.24, 34.94 (t, J_{C-D} = 24.6 Hz), 29.28, 29.00 (t, J_{C-D} = 24.6 Hz); HRMS calcd for $C_{26}H_{23}D_2ClNO_4$ ($[M+H]^+$): 452.1592; found: 452.1601.

The following signals are discernible for the *cis*-isomer: ^{1}H NMR (500 MHz, $CDCl_3$) δ 4.18 (d, *J* = 14.8 Hz, 1 H), 4.11 (d, *J* = 14.8 Hz, 1 H), 3.76 (s, 3 H), 3.42 (s, 3 H); ^{13}C NMR (126 MHz, $CDCl_3$) δ 129.38, 122.08, 121.77.

(7) Dimethyl (2*S,3*R**)-2-((diphenylamino)methyl)-3-(4-(trifluoromethyl)phenyl) cyclopropane-1,1-dicarboxylate-2,3-*d*$_2$ (7) (wyq-7-37).**

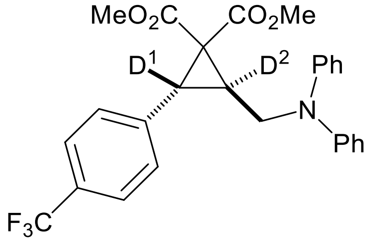

7

trans:cis = 13:1, D[1], D[2] = 95%, 95%

Following General Procedure II, **7** was afforded as a colorless oil (33.2 mg, 67%, D^1/D^2 = 95%/95%, *trans:cis* = 13:1), eluent (0-10% of ethyl acetate in petroleum ether).

The following signals are discernible for the *trans*-isomer: ^{1}H NMR (500 MHz, $CDCl_3$) δ 7.46 (d, *J* = 8.1 Hz, 2 H), 7.30 – 7.19 (m, 5 H), 7.14 (d, *J* = 8.0 Hz, 2 H), 7.03 – 6.98 (m, 3 H), 6.98 – 6.88 (m, 2 H), 4.02 (d, *J* = 15.2 Hz, 1 H), 3.84 (d, *J* = 15.2 Hz, 1 H), 3.65 (s, 3 H), 3.37 (s, 3 H), 3.17 (s, 0.05 H), 2.89 (t, *J* = 6.5 Hz, 0.05 H); ^{13}C NMR (126 MHz, $CDCl_3$) δ 167.87, 166.54, 147.57, 138.51, 129.57 (q, $J_{C\text{-}F}$ = 32.5 Hz), 129.47, 128.77, 125.06 (q, $J_{C\text{-}F}$ = 3.7 Hz), 124.01 (q, $J_{C\text{-}F}$ = 272.4 Hz) 121.94, 121.30, 52.95, 52.45, 49.18, 42.16, 35.85, 35.54 (t, $J_{C\text{-}D}$ = 24.6 Hz), 29.28, 28.99 (t, $J_{C\text{-}D}$= 24.6 Hz); ^{19}F NMR (471 MHz, $CDCl_3$) δ -62.57; HRMS calcd for $C_{27}H_{23}D_2F_3NO_4$ ($[M+H]^+$): 486.1856; found: 486.1864.

The following signals are discernible for the *cis*-isomer: ^{1}H NMR (500 MHz, $CDCl_3$) δ 4.34 (d, *J* = 15.3 Hz, 1 H), 3.78 (s, 3 H), 3.58 (s, 3 H); ^{13}C NMR (126 MHz, $CDCl_3$) δ 129.36, 129.25, 121.52, 121.47.

(8) Dimethyl (2*S,3*R**)-2-((diphenylamino)methyl)-3-(3-(methoxycarbonyl) phenyl)cyclopropane-1,1-dicarboxylate-2,3-*d*$_2$ (8) (wyq-4-26).**

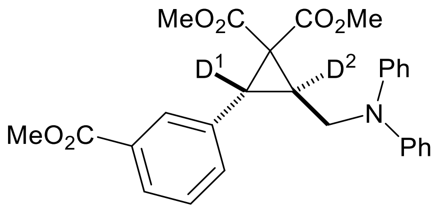

8

trans:cis = 12:1, D^1, D^2 = 92%, 93%

Following General Procedure I, **8** was afforded as a colorless oil (28.8 mg, 58%, D^1/D^2 = 92%/93%, *trans:cis* = 12:1), eluent (0-10% of ethyl acetate in petroleum ether).

The following signals are discernible for the *trans*-isomer: ^{1}H NMR (500 MHz, $CDCl_3$) δ 7.87 (d, *J* = 7.6 Hz, 1 H), 7.77 (s, 1 H), 7.30 – 7.27 (m, 2 H), 7.26 – 7.19 (m, 3 H), 7.03 (d, *J* = 8.0 Hz, 4 H), 6.99 – 6.87 (m, 3 H), 4.01 (d, *J* = 15.2 Hz, 1 H), 3.90 – 3.84 (m, 4 H), 3.63 (s, 3 H), 3.36 (s, 3 H), 3.19 (s, 0.08 H), 2.90 (t, *J* = 6.4 Hz, 0.07 H); ^{13}C NMR (126 MHz, $CDCl_3$) δ 168.00, 166.68, 147.61, 134.82, 132.71, 130.14, 129.76, 129.43, 128.60, 128.23, 121.87, 121.52, 121.34, 52.86, 52.34, 52.08, 49.20, 41.99, 35.99, 35.69 (t, $J_{C\text{-}D}$ = 23.9 Hz), 29.35, 29.04 (t, $J_{C\text{-}D}$ = 24.6 Hz); HRMS calcd for $C_{28}H_{26}D_2NO_6$ ($[M+H]^+$): 476.2037; found: 476.2046.

The following signals are discernible for the *cis*-isomer: ^{1}H NMR (500 MHz, $CDCl_3$) δ 3.77 (s, 3 H), 3.59 (s, 3 H), 3.37 (s, 3 H).

(9) Dimethyl (2*S,3*R**)-2-((diphenylamino)methyl)-3-(p-tolyl)cyclopropane-1,1-dicarboxylate-2,3-d_2 (9) (wyq-4-20).**

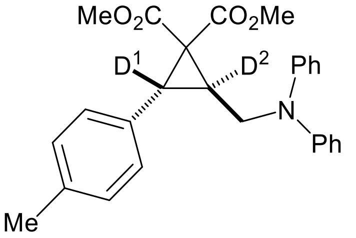

9

trans:cis = 9:1, D^1, D^2 = 93%, 93%

Following General Procedure I, **9** was afforded as a colorless oil (30.8 mg, 66%, D^1/D^2 = 93%/93%, *trans:cis* = 9:1), eluent (0-11% of ethyl acetate in petroleum ether).

The following signals are discernible for the *trans*-isomer: ^{1}H NMR (500 MHz, $CDCl_3$) δ 7.29 – 7.19 (m, 4 H), 7.01 (t, J = 6.2 Hz, 6 H), 6.96 (q, J = 7.6 Hz, 4 H), 3.97 (d, J = 15.0 Hz, 1 H), 3.87 (d, J = 15.2 Hz, 1 H), 3.60 (s, 3 H), 3.35 (s, 3 H), 3.13 (s, 0.07 H), 2.87 (t, J = 6.7 Hz, 0.07 H), 2.27 (s, 3 H); ^{13}C NMR (126 MHz, $CDCl_3$) δ 168.32, 166.94, 147.67, 136.95, 131.14, 129.39, 128.84, 128.24, 121.73, 121.29, 52.71, 52.22, 49.24, 46.15, 42.01, 36.20, 35.89 (t, $J_{C\text{-}D}$ = 23.9 Hz), 29.29, 29.00 (t, $J_{C\text{-}D}$ = 23.9 Hz), 21.04; HRMS calcd for $C_{27}H_{26}D_2NO_4$ ($[M+H]^+$): 432.2138; found: 432.2147.

The following signals are discernible for the *cis*-isomer: ^{1}H NMR (500 MHz, $CDCl_3$) δ 4.34 (d, J = 15.2 Hz, 1 H), 3.75 (s, 3 H), 3.54 (s, 3 H), 2.29 (s, 3 H); ^{13}C NMR (126 MHz, $CDCl_3$) δ 129.25, 129.09, 128.95, 121.52, 52.91, 52.12, 46.15.

(10) Dimethyl (2*S,3*R**)-2-((diphenylamino)methyl)-3-(4-ethylphenyl) cyclopropane-1,1-dicarboxylate-2,3-d_2 (10) (wyq-7-40).**

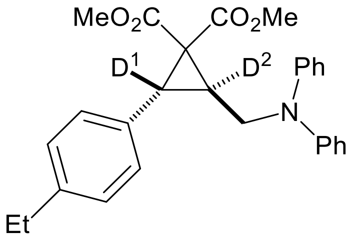

10

trans:cis = 14:1, D^1, D^2 = 93%, 92%

Following General Procedure II, **10** was afforded as a colorless oil (36.7 mg, 77%, D^1/D^2 = 93%/92%, *trans:cis* = 14:1), eluent (0-9% of ethyl acetate in petroleum ether).

The following signals are discernible for the *trans*-isomer: ^{1}H NMR (500 MHz, $CDCl_3$) δ 7.30 – 7.25 (m, 2 H), 7.27 – 7.22 (m, 2 H), 7.06 – 6.99 (m, 6 H), 7.00 – 6.91 (m, 4 H), 3.97 (d, J = 15.3 Hz, 1 H), 3.87 (d, J = 15.2 Hz, 1 H), 3.60 (s, 3 H), 3.34 (s, 3 H), 3.14 (s, 0.07 H), 2.88 (t, J = 6.5 Hz, 0.08 H), 2.57 (q, J = 7.6 Hz, 2 H), 1.18 (t, J = 7.6 Hz, 3 H); ^{13}C NMR (126 MHz, $CDCl_3$) δ 168.34, 166.96, 147.69, 143.36, 131.41, 129.41, 128.33, 127.63, 121.75, 121.31, 52.72, 52.21, 49.23, 42.07, 36.19, 35.88 (t, $J_{C\text{-}D}$ = 24.6 Hz), 29.36, 29.07 (t, $J_{C\text{-}D}$ = 24.6 Hz), 28.43, 15.41; HRMS calcd for $C_{28}H_{28}D_2NO_4$ ($[M+H]^+$): 446.2295; found: 446.2297.

The following signals are discernible for the *cis*-isomer: ^{1}H NMR (500 MHz, $CDCl_3$) δ 4.36 (d, J = 15.2 Hz, 1 H), 3.75 (s, 3 H), 3.56 (s, 3 H), 1.22 (t, J = 7.6 Hz, 3 H).

(11) Dimethyl (2*S,3*R**)-2-(4-(tert-butyl)phenyl)-3-((diphenylamino)methyl) cyclopropane-1,1-dicarboxylate-2,3-*d*$_2$ (11) (wyq-4-10).**

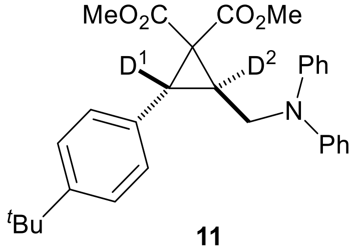

11

trans:cis = 13:1, D^1, D^2 = 95%, 94%

Following General Procedure I, **11** was afforded as a colorless oil (32.9 mg, 70%, D^1/D^2 = 95%/94%, *trans:cis* = 13:1), eluent (0-11% of ethyl acetate in petroleum ether).

The following signals are discernible for the *trans*-isomer: ^{1}H NMR (500 MHz, $CDCl_3$) δ 7.30 – 7.25 (m, 2 H), 7.27 – 7.20 (m, 4 H), 7.05 – 6.98 (m, 5 H), 7.00 – 6.93 (m, 3 H), 3.96 (d, J = 15.3 Hz, 1 H), 3.88 (d, J = 15.3 Hz, 1 H), 3.59 (s, 3 H), 3.32 (s, 3 H), 3.15 (s, 0.05 H), 2.89 (t, J = 6.4 Hz, 0.06 H), 1.26 (s, 9 H); ^{13}C NMR (126 MHz, $CDCl_3$) δ 168.36, 166.99, 150.29, 147.67, 131.19, 129.42, 128.06, 125.04, 121.78, 121.32, 52.74, 52.17, 49.20, 42.13, 35.97, 35.81, 35.69 (t, $J_{C\text{-}D}$ = 15.1 Hz), 35.57, 34.44,

31.25, 29.30, 29.00 (t, J_{C-D} = 14.5 Hz); HRMS calcd for $C_{30}H_{32}D_2NO_4$ ($[M+H]^+$): 474.2608; found: 474.2616.

The following signals are discernible for the *cis*-isomer: ^{1}H NMR (500 MHz, $CDCl_3$) δ 4.39 (d, *J* = 15.3 Hz, 2 H), 3.75 (s, 3 H), 3.58 (s, 3 H), 1.28 (s, 9 H).

(12) Diethyl (2*S,3*R**)-2-((diphenylamino)methyl)-3-phenylcyclopropane-1,1-dicarboxylate-2,3-*d*$_2$ (12) (wyq-4-17).**

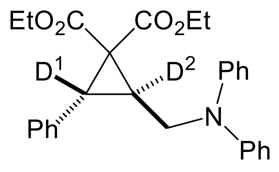

12

trans:cis = 10:1, D^1, D^2 = 94%, 95%

Following General Procedure I, **12** was afforded as a colorless oil (27.6 mg, 62%, D^1/D^2 = 94%/95%, *trans:cis* = 10:1), eluent (0-10% of ethyl acetate in petroleum ether).

The following signals are discernible for the *trans*-isomer: ^{1}H NMR (500 MHz, $CDCl_3$) δ 7.29 – 7.22 (m, 4 H), 7.22 – 7.14 (m, 3 H), 7.08 – 7.00 (m, 6 H), 6.99 – 6.91 (m, 2 H), 4.23 – 4.16 (m, 1 H), 4.06 – 3.96 (m, 2 H), 3.86 – 3.78 (m, 3 H), 3.15 (s, 0.06 H), 2.86 (t, *J* = 6.5 Hz, 0.05 H), 1.22 (t, *J* = 7.2 Hz, 3 H), 0.84 (t, *J* = 7.1 Hz, 3 H); ^{13}C NMR (126 MHz, $CDCl_3$) δ 167.87, 166.42, 147.77, 134.37, 129.38, 128.55, 128.04, 127.21, 121.75, 121.36, 61.75, 61.13, 49.33, 42.24, 42.24, 36.31, 36.00 (t, J_{C-D} = 24.6 Hz), 35.81, 28.76, 28.47 (t, J_{C-D} = 24.6 Hz), 14.05, 13.66; HRMS calcd for $C_{28}H_{28}D_2NO_4$ ($[M+H]^+$): 446.2295; found: 446.2297.

The following signals are discernible for the *cis*-isomer: ^{1}H NMR (500 MHz, $CDCl_3$) δ 1.27 – 1.25 (m, 3 H), 1.08 (t, *J* = 7.1 Hz, 3 H); ^{13}C NMR (126 MHz, $CDCl_3$) δ 129.26, 121.57.

(13) Di-*tert*-butyl (2*S,3*R**)-2-((diphenylamino)methyl)-3-phenylcyclopropane-1,1-dicarboxylate-2,3-*d*$_2$ (13) (wyq-4-33).**

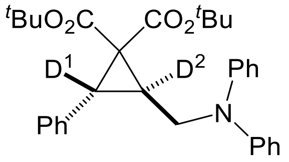

13

trans:cis = 18:1, D[1], D[2] = 95%, 88%

Following General Procedure I, **13** was afforded as a colorless oil (33.5 mg, 68%, D^1/D^2 = 95%/88%, *trans:cis* = 18:1), eluent (0-6% of ethyl acetate in petroleum ether).

The following signals are discernible for the *trans*-isomer: ^{1}H NMR (500 MHz, $CDCl_3$) δ 7.30 – 7.20 (m, 4 H), 7.20 – 7.07 (m, 3 H), 7.05 – 7.03 (m, 6 H), 6.95 (t, *J* = 7.3 Hz, 2 H), 4.06 (d, *J* = 14.8 Hz, 1 H), 3.70 (d, *J* = 14.8 Hz, 1 H), 3.05 (s, 0.05 H), 2.71 (dd, *J* = 8.5, 4.8 Hz, 0.12 H), 1.46 (s, 9 H), 1.10 (s, 9 H); ^{13}C NMR (126 MHz, $CDCl_3$) δ 167.17, 165.59, 147.81, 134.61, 129.38, 128.83, 127.84, 126.93, 121.74, 121.40, 82.06, 81.10, 49.39, 43.88, 35.78, 35.47 (t, J_{C-D} = 24.6 Hz), 30.20, 29.69 (t, J_{C-D} = 24.6 Hz), 28.04, 27.51; HRMS calcd for $C_{32}H_{36}D_2NO_4$ ($[M+H]^+$): 502.2921; found: 502.2925.

The following signals are discernible for the *cis*-isomer: ^{1}H NMR (500 MHz, $CDCl_3$) δ 1.48 (s, 9 H), 1.34 (s, 9 H); ^{13}C NMR (126 MHz, $CDCl_3$) δ 118.41, 27.87.

(14) Dibenzyl (2*S,3*R**)-2-((diphenylamino)methyl)-3-phenylcyclopropane-1,1-dicarboxylate-2,3-*d*$_2$ (14) (wyq-9-17).**

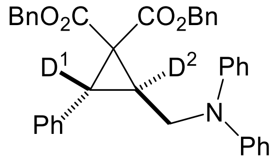

14

trans:cis = 10:1, D[1], D[2] = 93%, 96%

Following General Procedure III, **14** was afforded as a colorless oil (27.4 mg, 50%, D^1/D^2 = 93%/96%, *trans:cis* = 10:1), eluent (0-10% of ethyl acetate in petroleum ether).

The following signals are discernible for the *trans*-isomer: ^{1}H NMR (500 MHz, $CDCl_3$) δ 7.31 – 7.25 (m, 3 H), 7.23 (t, *J* = 7.8 Hz, 6 H), 7.17 (p, *J* = 7.9 Hz, 6 H), 7.05 – 7.00 (m, 2 H), 6.99 (d, *J* = 8.1 Hz, 4 H), 6.95 (t, *J* = 7.2 Hz, 2 H), 6.88 (d, *J* = 7.2 Hz,

2 H), 5.15 (d, J = 12.3 Hz, 1 H), 4.89 (d, J = 12.3 Hz, 1 H), 4.72 (q, J = 12.3 Hz, 2 H), 4.00 (d, J = 15.0 Hz, 1 H), 3.82 (d, J = 15.0 Hz, 1 H), 3.20 (s, 0.07 H), 2.92 (t, J = 6.7 Hz, 0.04 H); ^{13}C NMR (126 MHz, $CDCl_3$) δ 167.63, 166.24, 147.66, 135.35, 135.16, 134.04, 129.41, 128.49, 128.29, 128.16, 128.09, 128.05, 128.00, 127.36, 121.80, 121.36, 67.51, 67.18, 49.09, 42.23, 36.75, 36.45 (t, J_{C-D} = 24.6 Hz), 29.68, 28.99 (t, J_{C-D} = 24.6 Hz); HRMS calcd for $C_{38}H_{32}D_2NO_4$ ($[M+H]^+$): 570.2608; found: 570.2612.

The following signals are discernible for the *cis*-isomer: ^{1}H NMR (500 MHz, $CDCl_3$) δ 4.92 (s, 2 H), 4.36 (d, J = 15.2 Hz, 1 H).

(15) Dimethyl (2*S,3*R**)-2-((methyl(phenyl)amino)methyl)-3-phenylcyclopropane-1,1-dicarboxylate-2,3-*d*$_2$ (15) (wyq-3-37)**.

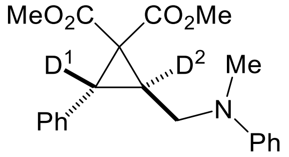

15

trans:cis = 23:1, D^1, D^2 = 96%, 94%

Following General Procedure I, **15** was afforded as a colorless oil (27.5 mg, 77%, D^1/D^2 = 96%/94%, *trans:cis* = 23:1), eluent (0-10% of ethyl acetate in petroleum ether).

The following signals are discernible for the *trans*-isomer: ^{1}H NMR (500 MHz, $CDCl_3$) δ 7.29 – 7.18 (m, 5 H), 7.12 (d, J = 6.6 Hz, 2 H), 6.84 (d, J = 8.1 Hz, 2 H), 6.75 (t, J = 7.3 Hz, 1 H), 3.77 (s, 3 H), 3.65 (d, J = 14.8 Hz, 1 H), 3.42 (d, J = 15.0 Hz, 1 H), 3.37 (s, 3 H), 3.30 (s, 0.04 H), 2.97 (s, 3 H), 2.79 (t, J = 6.7 Hz, 0.06 H); ^{13}C NMR (126 MHz, $CDCl_3$) δ 168.38, 167.01, 149.43, 134.24, 129.23, 128.47, 128.20, 127.39, 117.31, 113.36, 52.84, 52.29, 50.23, 41.43, 37.94, 36.52, 36.21 (t, J_{C-D} = 24.6 Hz), 28.51, 28.23 (t, J_{C-D} = 24.6 Hz); HRMS calcd for $C_{21}H_{22}D_2NO_4$ ($[M+H]^+$): 356.1825; found: 356.1837.

The following signals are discernible for the *cis*-isomer: ^{1}H NMR (500 MHz, $CDCl_3$) δ 2.81 (s, 3 H).

(16) Dimethyl (2*S,3*R**)-2-((ethyl(phenyl)amino)methyl)-3-phenylcyclopropane-1,1-dicarboxylate-2,3-*d*$_2$ (16) (wyq-9-3).**

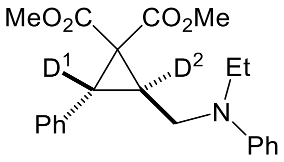

16

trans:cis = 17:1, D[1], D[2] = 92%, 97%

Following General Procedure III, **16** was afforded as a colorless oil (28.6 mg, 77%, D^1/D^2 = 92%/97%, *trans:cis* = 17:1), eluent (0-10% of ethyl acetate in petroleum ether).

The following signals are discernible for the *trans*-isomer: ^{1}H NMR (400 MHz, $CDCl_3$) δ 7.29 – 7.17 (m, 5 H), 7.14 (dd, *J* = 7.7, 1.9 Hz, 2 H), 6.81 (d, *J* = 7.7 Hz, 2 H), 6.72 (t, *J* = 7.2 Hz, 1 H), 3.79 (s, 3 H), 3.59 (d, *J* = 15.2 Hz, 1 H), 3.48 – 3.35 (m, 3 H), 3.37 (s, 3 H), 3.31 (s, 0.08 H), 2.81 (t, *J* = 6.4 Hz, 0.03 H) 1.15 (t, *J* = 7.0 Hz, 3 H); ^{13}C NMR (126 MHz, $CDCl_3$) δ 168.42, 167.00, 147.96, 134.31, 129.34, 128.49, 128.20, 127.39, 116.72, 113.06, 52.84, 52.28, 47.89, 44.75, 41.82, 36.78, 36.47 (t, J_{C-D} = 24.6 Hz), 29.67, 28.83 (t, J_{C-D} = 24.6 Hz), 12.13; HRMS calcd for $C_{22}H_{24}D_2NO_4$ ($[M+H]^+$): 370.1982; found: 370.1991.

The following signals are discernible for the *cis*-isomer: ^{1}H NMR (500 MHz, $CDCl_3$) δ 3.77 (s, 3 H), 3.67 (s, 3 H), 1.03 (t, *J* = 7.0 Hz, 3 H); ^{13}C NMR (126 MHz, $CDCl_3$) δ 129.08.

(17) Dimethyl (2*S,3*R**)-2-((isopropyl(phenyl)amino)methyl)-3-phenylcyclopropane-1,1-dicarboxylate-2,3-*d*$_2$ (17) (wyq-9-19).**

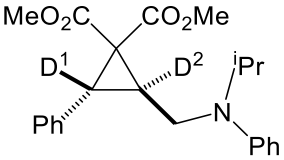

17

trans:cis > 20:1, D[1], D[2] = 99%, 96%

Following General Procedure I, **17** was afforded as a colorless oil (31.4 mg, 80%, D^1/D^2 = 99%/96%, *trans:cis* > 20:1), eluent (0-11% of ethyl acetate in petroleum ether); ^{1}H NMR (400 MHz, $CDCl_3$) δ 7.30 – 7.25 (m, 2 H), 7.22 – 7.17 (m, 3 H), 7.10 (dd, *J* = 7.4, 2.2 Hz, 2 H), 6.91 (d, *J* = 8.2 Hz, 2 H), 6.76 (t, *J* = 7.2 Hz, 1 H), 4.05 (hept, *J* = 6.7 Hz, 1 H), 3.84 (s, 3 H), 3.43 (d, *J* = 15.4 Hz, 1 H), 3.35 (s, 3 H), 3.25 (d, *J* = 15.4 Hz, 1

H), 3.16 (s, 0.01 H), 2.72 (dd, J = 7.2, 3.8 Hz, 0.04 H), 1.18 (t, J = 6.7 Hz, 6 H); ^{13}C NMR (126 MHz, $CDCl_3$) δ 68.53, 166.95, 148.49, 134.41, 129.31, 128.47, 128.15, 127.32, 117.24, 114.36, 52.81, 52.26, 48.85, 42.64, 42.11, 38.02, 37.70 (t, J_{C-D} = 30.2 Hz), 30.19, 29.89 (t, J_{C-D} = 30.7 Hz), 20.84, 19.77; HRMS calcd for $C_{23}H_{26}D_2NO_4$ ($[M+H]^+$): 384.2138; found: 384.2136.

(18) Dimethyl (2*S,3*R**)-2-(((2-chlorophenyl)(methyl)amino)methyl)-3-phenylcyclopropane-1,1-dicarboxylate-2,3-*d*₂ (18) (wyq-3-49).**

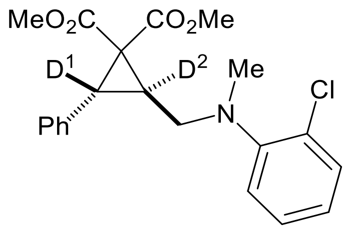

18

trans:cis = 8:1, D^1, D^2 = 98%, 85%

Following General Procedure I, **18** was afforded as a colorless oil (35.7 mg, 90%, D^1/D^2 = 98%/85%, *trans:cis* = 8:1), eluent (0-11% of ethyl acetate in petroleum ether).

The following signals are discernible for the *trans*-isomer: ^{1}H NMR (500 MHz, $CDCl_3$) δ 7.36 – 7.33 (m, 1 H), 7.26 – 7.18 (m, 4 H), 7.16 – 7.10 (m, 3 H), 6.97 – 6.93 (m, 1 H), 3.72 (s, 3 H), 3.42 – 3.34 (m, 4 H), 3.29 (d, J = 13.9 Hz, 1 H), 3.22 (s, 0.02 H), 2.89 (s, 3 H), 2.79 (t, J = 6.7 Hz, 0.15 H); ^{13}C NMR (126 MHz, $CDCl_3$) δ 168.17, 167.09, 149.04, 134.35, 130.66, 128.90, 128.37, 128.16, 128.13, 127.32, 123.68, 121.82, 52.74, 52.59, 52.27, 41.66, 40.47, 36.19, 35.88 (t, J_{C-D} = 23.9 Hz), 28.57, 28.28 (t, J_{C-D} = 23.9 Hz); HRMS calcd for $C_{21}H_{21}D_2ClNO_4$ ($[M+H]^+$): 390.1436; found: 390.1442.

The following signals are discernible for the *cis*-isomer: ^{1}H NMR (500 MHz, $CDCl_3$) δ 3.78 (s, 3 H), 3.61 (s, 3 H), 2.76 (s, 3 H); ^{13}C NMR (126 MHz, $CDCl_3$) δ 129.54, 127.03, 125.77, 123.86, 122.16, 120.27, 119.36, 110.55, 52.96, 52.11, 41.32.

(19) Dimethyl (2*S,3*R**)-2-(((3-chlorophenyl)(methyl)amino)methyl)-3-phenylcyclopropane-1,1-dicarboxylate-2,3-*d*₂ (19) (wyq-4-4-2).**

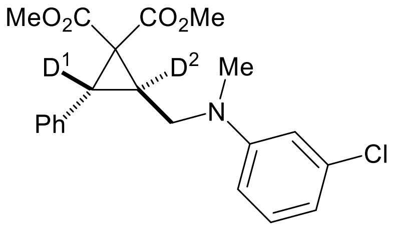

19

trans:cis = 14:1, D[1], D[2] = 96%, 92%

Following General Procedure I, **19** was afforded as a colorless oil (27.1 mg, 67%, D^1/D^2 = 96%/92%, *trans:cis* = 14:1), eluent (0-12% of ethyl acetate in petroleum ether).

The following signals are discernible for the *trans*-isomer: ^{1}H NMR (500 MHz, $CDCl_3$) δ 7.34 – 7.19 (m, 3 H), 7.18 – 7.10 (m, 3 H), 6.76 (s, 1 H), 6.73 – 6.64 (m, 2 H), 3.77 (s, 3 H), 3.63 (d, *J* = 15.0 Hz, 1 H), 3.43 (d, *J* = 15.1 Hz, 1 H), 3.38 (s, 3 H), 3.29 (s, 0.04 H), 2.96 (s, 3 H), 2.76 (t, *J* = 6.7 Hz, 0.08 H); ^{13}C NMR (126 MHz, $CDCl_3$) δ 168.33, 166.88, 150.46, 135.19, 134.10, 130.15, 128.44, 128.28, 127.50, 117.03, 113.00, 111.22, 52.89, 52.34, 49.90, 41.53, 37.87, 36.43, 36.12 (t, $J_{C\text{-}D}$ = 24.6 Hz), 28.47, 28.18 (t, $J_{C\text{-}D}$ = 24.6 Hz); HRMS calcd for $C_{21}H_{21}D_2ClNO_4$ ($[M+H]^+$): 390.1436; found: 390.1441.

The following signals are discernible for the *cis*-isomer: ^{1}H NMR (500 MHz, $CDCl_3$) δ 4.02 (d, *J* = 15.4 Hz, 1 H), 3.78 (s, 3 H), 3.67 (s, 3 H), 2.81 (s, 3 H).

(20) Dimethyl (2*S,3*R**)-2-(((4-fluorophenyl)(methyl)amino)methyl)-3-phenylcyclopropane-1,1-dicarboxylate-2,3-*d*$_2$ (20) (wyq-8-50).**

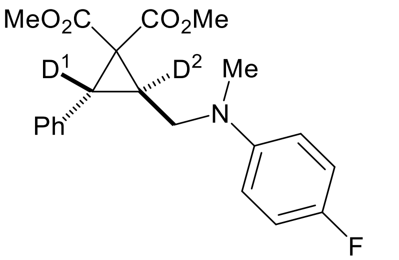

20

trans:cis = 14:1, D[1], D[2] = 95%, 94%

Following General Procedure III, **20** was afforded as a colorless oil (30.7 mg, 79%, D^1/D^2 = 95%/94%, *trans:cis* = 14:1), eluent (0-12% of ethyl acetate in petroleum ether).

The following signals are discernible for the *trans*-isomer: ^{1}H NMR (500 MHz, $CDCl_3$) δ 7.29 – 7.18 (m, 3 H), 7.11 (d, *J* = 6.2 Hz, 2 H), 6.95 (t, *J* = 8.7 Hz, 2 H), 6.81 – 6.75 (m, 2 H), 3.76 (s, 3 H), 3.57 (d, *J* = 14.7 Hz, 1 H), 3.39 – 3.35 (m, 4 H), 3.27 (s,

0.05 H), 2.94 – 2.90 (m, 3 H), 2.75 (t, J = 6.8 Hz, 0.06 H); ^{13}C NMR (126 MHz, $CDCl_3$) δ 168.36, 167.00, 156.06 (d, $J_{C\text{-}F}$ = 236.4 Hz), 146.36, 134.23, 128.42, 128.24, 127.44, 115.58 (d, $J_{C\text{-}F}$ = 22.1 Hz), 115.24 (d, $J_{C\text{-}F}$ = 7.4 Hz), 52.81, 52.30, 51.22, 41.51, 38.56, 36.57, 36.27 (t, $J_{C\text{-}D}$ = 24.6 Hz), 29.67, 28.13 (t, $J_{C\text{-}D}$ = 24.6 Hz); ^{19}F NMR (471 MHz, $CDCl_3$) δ -127.68; HRMS calcd for $C_{21}H_{21}D_2FNO_4$ ($[M+H]^+$): 374.1731; found: 374.1737.

The following signals are discernible for the *cis*-isomer: ^{1}H NMR (500 MHz, $CDCl_3$) δ 3.77 (s, 3 H), 3.65 (s, 3 H), 2.78 (s, 3 H).

(21) Dimethyl (2*S,3*R**)-2-(((3-methoxyphenyl)(methyl)amino)methyl)-3-phenylcyclopropane-1,1-dicarboxylate-2,3-*d*$_2$ (21) (wyq-9-4).**

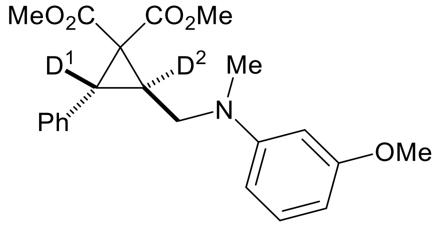

21

trans:cis = 16:1, D[1], D[2] = 96%, 95%

Following General Procedure III, **21** was afforded as a colorless oil (17.1 mg, 65%, D^1/D^2 = 96%/95%, *trans:cis* = 16:1), eluent (0-12% of ethyl acetate in petroleum ether).

The following signals are discernible for the *trans*-isomer: ^{1}H NMR (400 MHz, $CDCl_3$) δ 7.28 – 7.19 (m, 2 H), 7.24 – 7.09 (m, 4 H), 6.46 (dd, J = 8.3, 2.1 Hz, 1 H), 6.36 (t, J = 2.4 Hz, 1 H), 6.33 (dd, J = 8.1, 1.9 Hz, 1 H), 3.80 (s, 3 H), 3.78 (s, 3 H), 3.65 (d, J = 15.1 Hz, 1 H), 3.40 (d, J = 15.0 Hz, 1 H), 3.37 (s, 3 H), 3.31 (s, 0.04 H), 2.96 (s, 3 H), 2.39 (t, J = 7.5 Hz, 0.05 H); ^{13}C NMR (126 MHz, $CDCl_3$) δ 168.38, 166.98, 160.79, 150.79, 134.22, 129.91, 128.49, 128.21, 127.40, 106.31, 102.13, 99.84, 55.11, 52.85, 52.29, 50.19, 41.43, 38.01, 36.49, 36.18 (t, $J_{C\text{-}D}$ = 25.2 Hz), 28.52, 28.23 (t, $J_{C\text{-}D}$ = 25.2 Hz); HRMS calcd for $C_{22}H_{24}D_2NO_5$ ($[M+H]^+$): 386.1931; found: 386.1940.

The following signals are discernible for the *cis*-isomer: ^{1}H NMR (500 MHz, $CDCl_3$) δ 3.77 (s, 3 H), 3.76 (s, 3 H), 3.67 (s, 3 H), 2.80 (s, 3 H).

(22) Dimethyl (2*S,3*R**)-2-(((3,5-dimethylphenyl)(methyl)amino)methyl)-3-phenylcyclopropane-1,1-dicarboxylate-2,3-*d*$_2$ (22) (wyq-4-9).**

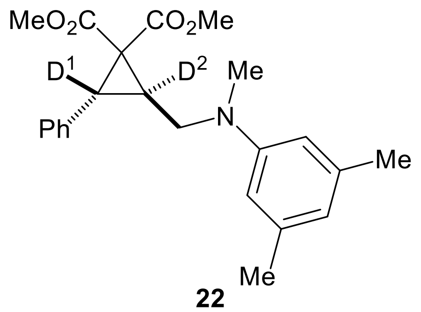

22

trans:cis = 14:1, D[1], D[2] = 96%, 93%

Following General Procedure I, **22** was afforded as a colorless oil (29.9 mg, 77%, D^1/D^2 = 96%/93%, *trans:cis* = 14:1), eluent (0-11% of ethyl acetate in petroleum ether).

The following signals are discernible for the *trans*-isomer: ^{1}H NMR (500 MHz, $CDCl_3$) δ 7.27 – 7.18 (m, 3 H), 7.14 (d, *J* = 6.7 Hz, 2 H), 6.47 (s, 2 H), 6.43 (s, 1 H), 3.78 (s, 3 H), 3.63 (d, *J* = 14.9 Hz, 1 H), 3.37 (s, 3 H), 3.36 (d, 1 H), 3.29 (s, 0.04 H), 2.94 (s, 3 H), 2.77 (t, *J* = 6.3 Hz, 0.07 H), 2.28 (s, 6 H); ^{13}C NMR (126 MHz, $CDCl_3$) δ 168.36, 167.04, 149.65, 138.70, 134.30, 128.50, 128.18, 127.35, 119.42, 111.46, 52.80, 52.26, 50.43, 41.49, 38.07, 36.53, 36.22 (t, $J_{C\text{-}D}$ = 25.2 Hz), 31.23, 28.63, 28.35 (t, $J_{C\text{-}D}$ = 23.9 Hz), 21.73; HRMS calcd for $C_{23}H_{26}D_2NO_4$ ($[M+H]^+$): 384.2138; found: 384.2147.

The following signals are discernible for the *cis*-isomer: ^{1}H NMR (500 MHz, $CDCl_3$) δ 3.76 (s, 3 H), 3.67 (s, 3 H), 2.81 (s, 3 H), 2.24 (s, 6 H). ^{13}C NMR (126 MHz, $CDCl_3$) δ 129.10, 125.08.

(23) Dimethyl (2*S,3*R**)-2-((methyl(p-tolyl)amino)methyl)-3-phenylcyclo propane-1,1-dicarboxylate-2,3-*d₂* (23) (wyq-9-2).**

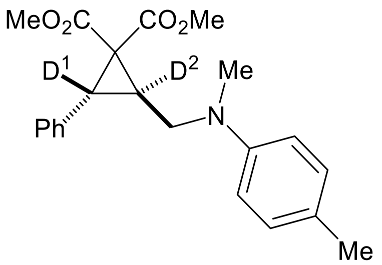

23

trans:cis = 15:1, D[1], D[2] = 95%, 96%

Following General Procedure III, **23** was afforded as a colorless oil (23.8 mg, 65%, D^1/D^2 = 95%/96%, *trans:cis* = 15:1), eluent (0-10% of ethyl acetate in petroleum ether).

The following signals are discernible for the *trans*-isomer: ^{1}H NMR (500 MHz, $CDCl_3$) δ 7.27 – 7.17 (m, 3 H), 7.15 – 7.10 (m, 2 H), 7.06 (d, *J* = 8.3 Hz, 2 H), 6.76 (d, *J* = 8.2 Hz, 2 H), 3.76 (s, 3 H), 3.60 (d, *J* = 14.8 Hz, 1 H), 3.39 – 3.31 (m, 4 H), 3.28 (s, 0.05 H), 2.93 (s, 3 H), 2.76 (t, *J* = 6.8 Hz, 0.04 H), 2.26 (s, 3 H); ^{13}C NMR (126 MHz, $CDCl_3$) δ 168.38, 167.05, 147.56, 134.36, 129.75, 129.21, 128.51, 128.19, 127.36, 114.03, 52.79, 52.24, 50.83, 41.49, 38.22, 36.59, 36.28 (t, $J_{C\text{-}D}$ = 24.6 Hz), 28.62, 28.21 (t, $J_{C\text{-}D}$ = 24.6 Hz), 20.24; HRMS calcd for $C_{22}H_{24}D_2NO_4$ ([M+H]$^+$): 370.1982; found: 370.1988.

The following signals are discernible for the *cis*-isomer: ^{1}H NMR (500 MHz, $CDCl_3$) δ 3.65 (s, 3 H), 2.79 (s, 3H), 2.24 (s, 3 H).

(24) Dimethyl (2*S,3*R**)-2-(((3-(methoxycarbonyl)phenyl)(methyl)amino)methyl) -3-phenylcyclopropane-1,1-dicarboxylate-2,3-*d*$_2$ (24) (wyq-7-45).**

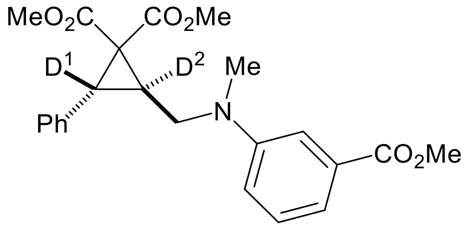

24

trans:cis > 20:1, D^1, D^2 = 94%, 94%

Following General Procedure II, **24** was afforded as a colorless oil (23.3 mg, 56%, D^1/D^2 = 94%/94%, *trans:cis* > 20:1), eluent (0-12% of ethyl acetate in petroleum ether); ^{1}H NMR (500 MHz, $CDCl_3$) δ 7.49 (s, 1 H), 7.42 (d, *J* = 8.0 Hz, 1 H), 7.31 (t, *J* = 7.9 Hz, 1 H), 7.25 – 7.18 (m, 3 H), 7.12 (dd, *J* = 8.0, 1.7 Hz, 2 H), 7.02 (dd, *J* = 8.3, 2.8 Hz, 1 H), 3.90 (s, 3 H), 3.77 (s, 3 H), 3.68 (d, *J* = 15.0 Hz, 1 H), 3.50 (d, *J* = 15.1 Hz, 1 H), 3.37 (s, 3 H), 3.31 (s, 0.06 H), 3.01 (s, 3 H), 2.78 (t, *J* = 6.7 Hz, 0.06 H); ^{13}C NMR (126 MHz, $CDCl_3$) δ 168.31, 167.57, 166.89, 149.30, 134.07, 131.03, 129.19, 128.41, 128.23, 127.44, 118.34, 117.52, 113.93, 52.87, 52.32, 52.00, 49.96, 41.45, 37.98, 36.41, 36.10 (t, $J_{C\text{-}D}$ = 24.6 Hz), 28.42, 28.13 (t, $J_{C\text{-}D}$ = 24.6 Hz); HRMS calcd for $C_{23}H_{24}D_2NO_6$ ([M+H]$^+$): 414.1880; found: 414.1887.

(25) Dimethyl (2*S,3*R**)-2-(((4-ethynylphenyl)(methyl)amino)methyl)-3-phenylcyclopropane-1,1-dicarboxylate-2,3-*d*$_2$ (25) (wyq-4-14-2).**

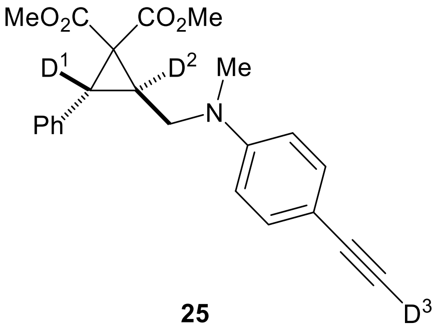

25

trans:cis > 20:1, D1, D2, D3 = 89%, 94%, 62%

Following General Procedure I and without the aqueous work-up procedure, the crude mixture was directly subjected to flash chromatography eluent (0-10% of ethyl acetate in petroleum ether) to afford **25** as a colorless oil (21.1 mg, 53%, $D^1/D^2/D^3$ = 89%/94%/62%, *trans:cis* > 20:1).

The following signals are discernible for the *trans*-isomer: ^{1}H NMR (400 MHz, $CDCl_3$) δ 7.39 (d, J = 8.7 Hz, 2 H), 7.25 – 7.18 (m, 3 H), 7.14 – 7.06 (m, 2 H), 6.73 (d, J = 8.8 Hz, 2 H), 3.77 (s, 3 H), 3.68 (d, J = 15.1 Hz, 1 H), 3.46 (d, J = 15.1 Hz, 1 H), 3.37 (s, 3 H), 3.31 (s, 0.11 H), 3.00 (s, 3 H), 2.99 (s, 0.38 H), 2.77 (t, J = 6.7 Hz, 0.06 H); ^{13}C NMR (126 MHz, $CDCl_3$) δ 168.34, 166.88, 149.29, 134.02, 133.40, 128.41, 128.28, 127.51, 112.34, 109.63, 75.00, 52.93, 52.36, 49.56, 41.43, 39.97 (t, $J_{C\text{-}D}$ = 25.2 Hz), 37.80, 29.67, 29.31 (t, $J_{C\text{-}D}$ = 23.9 Hz); HRMS calcd for $C_{23}H_{22}D_3NO_4$ ($[M+H]^+$): 381.1888; found: 381.1890.

(26) Dimethyl (2*S,3*R**)-2-((9H-carbazol-9-yl)methyl)-3-phenylcyclopropane-1,1-dicarboxylate-2,3-*d*$_2$ (26) (wyq-4-27).**

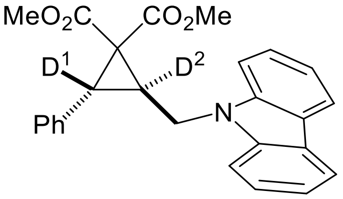

26

trans:cis > 20:1, D1, D2 = 91%, 91%

Following General Procedure I, **26** was afforded as a colorless oil (28.0 mg, 66%, D^1/D^2 = 91%/91%, *trans:cis* > 20:1), eluent (0-11% of ethyl acetate in petroleum ether).

The following signals are discernible for the *trans*-isomer: ^{1}H NMR (400 MHz, $CDCl_3$) δ 8.10 (d, *J* = 7.8 Hz, 2 H), 7.57 – 7.43 (m, 4 H), 7.30 – 7.20 (m, 2 H), 7.16 – 7.08 (m, 3 H), 6.97 – 6.88 (m, 2 H), 4.65 (d, *J* = 15.3 Hz, 1 H), 4.46 (d, *J* = 15.4 Hz, 1 H), 3.84 (s, 3 H), 3.52 (s, 0.09 H), 3.34 (s, 3 H), 3.16 – 3.10 (m, 0.09 H); ^{13}C NMR (126 MHz, $CDCl_3$) δ 168.45, 166.58, 140.28, 133.71, 128.43, 128.16, 127.47, 123.16, 120.40, 119.25, 108.73, 53.11, 52.36, 41.72, 40.55, 36.64, 36.34 (t, J_{C-D} = 24.6 Hz), 29.46, 29.16 (t, J_{C-D} = 24.6 Hz); HRMS calcd for $C_{26}H_{22}D_2NO_4$ ([M+H]$^+$): 416.1825; found: 416.1831.

The following signals are discernible for the *cis*-isomer: ^{13}C NMR (126 MHz, $CDCl_3$) δ 128.71, 125.61, 120.16, 119.05, 109.05.

(27) Dimethyl (2*S,3*R**)-2-phenyl-3-((phenylamino)methyl)cyclopropane-1,1-dicarboxylate-2,3-*d*$_2$ (27) (wyq-5-29).**

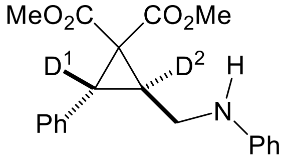

27

trans:cis = 14:1, D^1, D^2 = 92%, 90%

Following General Procedure I, **27** was afforded as a white solid (31.6 mg, 91%, D^1/D^2 = 92%/90%, *trans:cis* = 14:1), eluent (0-11% of ethyl acetate in petroleum ether): m.p. 72.9-73.4 °C.

The following signals are discernible for the *trans*-isomer: ^{1}H NMR (500 MHz, $CDCl_3$) δ 7.26 (q, *J* = 7.2, 6.1 Hz, 3 H), 7.21 – 7.15 (m, 4 H), 6.73 (t, *J* = 7.2 Hz, 1 H), 6.64 (d, *J* = 8.0 Hz, 2 H), 3.76 (s, 3 H), 3.48 (d, *J* = 13.2 Hz, 1 H), 3.40 (s, 3 H), 3.27 (s, 0.08 H), 3.23 (d, *J* = 13.2 Hz, 1 H), 2.86 (t, *J* = 7.0 Hz, 0.01 H); ^{13}C NMR (126 MHz, $CDCl_3$) δ 168.15, 167.03, 147.75, 134.12, 129.28, 128.48, 128.22, 127.45, 117.90, 113.05, 52.95, 52.38, 42.27, 41.91, 35.87, 35.57 (t, J_{C-D} = 24.6 Hz), 30.15, 29.84 (t, J_{C-D} = 24.6 Hz); HRMS calcd for $C_{20}H_{20}D_2NO_4$ ([M+H]$^+$): 342.1669; found: 342.1672.

The following signals are discernible for the *cis*-isomer: ^{1}H NMR (500 MHz, $CDCl_3$) δ 3.78 (s, 3 H), 3.66 (s, 3 H); ^{13}C NMR (126 MHz, $CDCl_3$) δ 129.62, 113.43.

(28) Dimethyl (2*S,3*R**)-2-((phenylamino)methyl)-3-(p-tolyl)cyclopropane-1,1-dicarboxylate-2,3-*d*$_2$ (28) (wyq-5-36).**

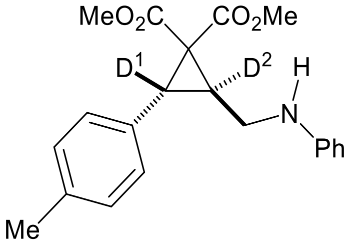

28

trans:cis = 10:1, D[1], D[2] = 93%, 86%

Following General Procedure I, **28** was afforded as a colorless oil (29.3 mg, 80%, D^1/D^2 = 93%/86%, *trans:cis* = 10:1), eluent (0-8% of ethyl acetate in petroleum ether).

The following signals are discernible for the *trans*-isomer: ^{1}H NMR (500 MHz, $CDCl_3$) δ 7.18 (t, *J* = 7.7 Hz, 2 H), 7.07 (s, 4 H), 6.72 (t, *J* = 7.2 Hz, 1 H), 6.64 (d, *J* = 7.9 Hz, 2 H), 3.75 (s, 3 H), 3.47 (d, *J* = 13.1 Hz, 1 H), 3.43 (s, 3 H), 3.34 (s, 0.07 H), 3.22 (d, *J* = 13.1 Hz, 1 H), 2.83 (t, *J* = 7.1 Hz, 0.14 H), 2.29 (s, 3 H); ^{13}C NMR (126 MHz, $CDCl_3$) δ 168.23, 167.12, 147.80, 137.11, 131.00, 129.26, 128.94, 128.34, 117.84, 113.03, 52.91, 52.39, 42.29, 41.83, 35.69, 35.38 (t, $J_{C\text{-}D}$ = 24.6 Hz), 30.22, 29.92 (t, $J_{C\text{-}D}$ = 24.6 Hz), 21.06; HRMS calcd for $C_{21}H_{22}D_2NO_4$ ([M+H]$^+$): 356.1825; found: 356.1834.

The following signals are discernible for the *cis*-isomer: ^{1}H NMR (500 MHz, $CDCl_3$) δ 3.77 (s, 3 H), 3.66 (s, 3 H), 2.31 (s, 3 H); ^{13}C NMR (126 MHz, $CDCl_3$) δ 129.48, 129.08, 113.37.

(29) Dimethyl (2*R,3*S**)-2-(4-ethylphenyl)-3-((phenylamino)methyl) cyclopropane-1,1-dicarboxylate-2,3-*d*$_2$ (29) (wyq-5-34).**

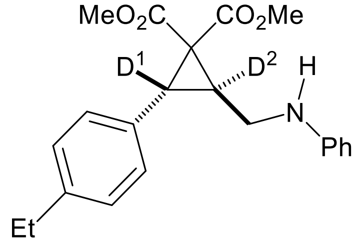

29

trans:cis = 12:1, D[1], D[2] = 95%, 84%

Following General Procedure I, **29** was afforded as a colorless oil (27.3 mg, 69%, D^1/D^2 = 95%/84%, *trans:cis* = 12:1), eluent (0-8% of ethyl acetate in petroleum ether).

The following signals are discernible for the *trans*-isomer: ^{1}H NMR (500 MHz, $CDCl_3$) δ 7.18 (t, *J* = 7.8 Hz, 2 H), 7.09 (s, 4 H), 6.73 (t, *J* = 7.2 Hz, 1 H), 6.64 (d, *J* = 7.8 Hz, 2 H), 3.75 (s, 3 H), 3.46 (d, *J* = 13.1 Hz, 1 H), 3.42 (s, 3 H), 3.32 (s, 0.05 H), 3.22 (d, *J* = 13.0 Hz, 1 H), 2.84 (t, *J* = 7.0 Hz, 0.16 H), 2.60 (q, *J* = 7.7 Hz, 2 H), 1.19 (t, *J* = 7.6 Hz, 3 H); ^{13}C NMR (126 MHz, $CDCl_3$) δ 168.22, 167.12, 147.80, 143.49, 131.23, 129.26, 128.40, 127.71, 117.83, 113.02, 52.91, 52.37, 42.27, 41.88, 35.71, 35.41 (t, $J_{C\text{-}D}$ = 24.6 Hz), 30.23, 29.93 (t, $J_{C\text{-}D}$ = 24.6 Hz), 28.43, 15.40; HRMS calcd for $C_{22}H_{24}D_2NO_4$ ($[M+H]^+$): 370.1982; found: 370.1988.

The following signals are discernible for the *cis*-isomer: ^{1}H NMR (500 MHz, $CDCl_3$) δ 3.77 (s, 3 H), 3.67 (s, 3 H); ^{13}C NMR (126 MHz, $CDCl_3$) δ 129.53, 113.34.

(30) Dimethyl (2*R,3*S**)-2-(4-(tert-butyl)phenyl)-3-((phenylamino)methyl) cyclopropane-1,1-dicarboxylate-2,3-*d*₂ (30) (wyq-5-35).**

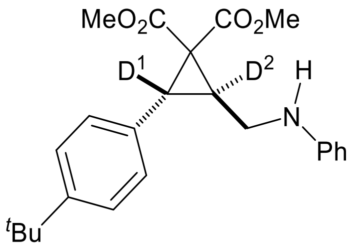

30

trans:cis = 10:1, D^1, D^2 = 94%, 80%

Following General Procedure I, **30** was afforded as a white solid (33.4 mg, 85%, D^1/D^2 = 94%/80%, *trans:cis* = 10:1), eluent (0-11% of ethyl acetate in petroleum ether): m.p. 87.5-89.0 °C.

The following signals are discernible for the *trans*-isomer: ^{1}H NMR (500 MHz, $CDCl_3$) δ 7.28 (d, *J* = 7.9 Hz, 2 H), 7.18 (t, *J* = 7.6 Hz, 2 H), 7.11 (d, *J* = 7.9 Hz, 2 H), 6.73 (t, *J* = 7.5 Hz, 1 H), 6.64 (d, *J* = 8.0 Hz, 2 H), 3.75 (s, 3 H), 3.45 (d, *J* = 13.1 Hz, 1 H), 3.40 (s, 3 H), 3.32 (s, 0.06 H), 3.23 (d, *J* = 13.2 Hz, 1 H), 2.85 (t, *J* = 7.1 Hz, 0.20 H), 1.28 (s, 9 H); ^{13}C NMR (126 MHz, $CDCl_3$) δ 168.21, 167.13, 150.41, 147.77, 130.98, 129.27, 128.12, 125.12, 117.86, 113.04, 52.92, 52.33, 42.26, 41.94, 35.60, 35.30 (t, $J_{C\text{-}D}$ = 24.6 Hz), 34.44, 31.25, 30.21, 29.90 (t, $J_{C\text{-}D}$ = 24.6 Hz); HRMS calcd for $C_{24}H_{28}D_2NO_4$ ($[M+H]^+$): 398.2295; found: 398.2300.

The following signals are discernible for the *cis*-isomer: ^{1}H NMR (500 MHz, $CDCl_3$) δ 3.77 (s, 3 H), 3.68 (s, 3 H), 1.29 (s, 9 H); ^{13}C NMR (126 MHz, $CDCl_3$) δ 125.29, 113.51.

(31) Dimethyl (2*S,3*S**)-2-(2-chlorophenyl)-3-((phenylamino)methyl) cyclopropane-1,1-dicarboxylate-2,3-*d*$_2$ (31) (wyq-5-31).**

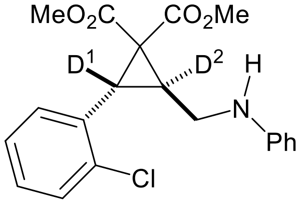

31

trans:cis = 10:1, D^1, D^2 = 94%, 94%

Following General Procedure I, **31** was afforded as a colorless oil (20.4 mg, 54%, D^1/D^2 = 94%/94%, *trans:cis* = 10:1), eluent (0-9% of ethyl acetate in petroleum ether).

The following signals are discernible for the *trans*-isomer: ^{1}H NMR (500 MHz, $CDCl_3$) δ 7.35 (dd, *J* = 7.2, 2.0 Hz, 1 H), 7.22 – 7.15 (m, 4 H), 7.13 – 7.07 (m, 1 H), 6.74 (t, *J* = 7.3 Hz, 1 H), 6.66 (d, *J* = 7.4 Hz, 2 H), 3.79 (s, 3 H), 3.53 (d, *J* = 13.3 Hz, 1 H), 3.44 (s, 3 H), 3.34 (s, 0.06 H), 3.24 (d, *J* = 13.3 Hz, 1 H), 2.89 (t, *J* = 7.1 Hz, 0.06 H); ^{13}C NMR (126 MHz, $CDCl_3$) δ 167.84, 167.10, 147.68, 135.88, 132.31, 129.63, 129.31, 129.31, 128.82, 126.42, 117.99, 113.09, 52.98, 52.47, 42.41, 41.18, 34.58, 34.29 (t, $J_{C\text{-}D}$ = 25.2 Hz), 30.51, 30.21 (t, $J_{C\text{-}D}$ = 24.6 Hz); HRMS calcd for $C_{20}H_{19}D_2ClNO_4$ ([M+H]$^+$): 376.1279; found: 376.1288.

(32) Dimethyl (2*R,3*S**)-2-(4-chlorophenyl)-3-((phenylamino)methyl) cyclopropane-1,1-dicarboxylate-2,3-*d*$_2$ (32) (wyq-5-33).**

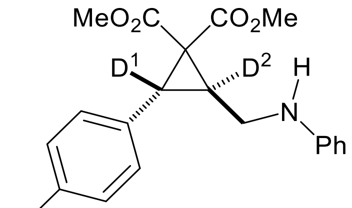

32

trans:cis = 10:1, D^1, D^2 = 95%, 92%

Following General Procedure I, **32** was afforded as a colorless oil (30.0 mg, 79%, D^1/D^2 = 95%/92%, *trans:cis* = 10:1), eluent (0-9% of ethyl acetate in petroleum ether).

The following signals are discernible for the *trans*-isomer: ^{1}H NMR (500 MHz, $CDCl_3$) δ 7.23 (d, J = 8.5 Hz, 2 H), 7.18 (t, J = 7.8 Hz, 2 H), 7.11 (d, J = 8.3 Hz, 2 H), 6.73 (t, J = 7.3 Hz, 1 H), 6.64 (d, J = 8.0 Hz, 2 H), 3.76 (s, 3 H), 3.44 (m, 4 H), 3.28 (s, 0.05 H), 3.23 (d, J = 13.4 Hz, 1 H), 2.81 (t, J = 6.9 Hz, 0.08 H); ^{13}C NMR (126 MHz, $CDCl_3$) δ 167.94, 166.84, 147.65, 133.36, 132.69, 129.88, 129.30, 128.41, 118.00, 113.06, 53.03, 52.54, 42.18, 41.86, 35.13, 34.83 (t, $J_{C\text{-}D}$ = 25.2 Hz), 30.26, 29.96 (t, $J_{C\text{-}D}$ = 25.2 Hz); HRMS calcd for $C_{20}H_{19}D_2ClNO_4$ ($[M+H]^+$): 376.1279; found: 376.1287.

The following signals are discernible for the *cis*-isomer: ^{1}H NMR (500 MHz, $CDCl_3$) δ 3.78 (s, 3 H), 3.66 (s, 3 H); ^{13}C NMR (126 MHz, $CDCl_3$) δ 131.12.

(33) Dimethyl (2*R,3*S**)-2-(4-bromophenyl)-3-((phenylamino)methyl) cyclopropane-1,1-dicarboxylate-2,3-*d*$_2$ (33) (wyq-5-38).**

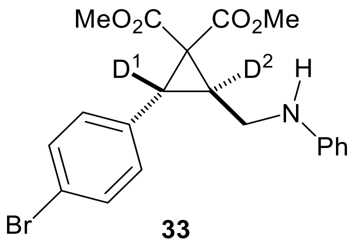

33

trans:cis = 12:1, D^1, D^2 = 93%, 89%

Following General Procedure I, **33** was afforded as a colorless oil (34.4 mg, 79%, D^1/D^2 = 93%/89%, *trans:cis* = 12:1), eluent (0-9% of ethyl acetate in petroleum ether).

The following signals are discernible for the *trans*-isomer: ^{1}H NMR (500 MHz, $CDCl_3$) δ 7.39 (d, J = 8.5 Hz, 2 H), 7.19 (t, J = 7.7 Hz, 2 H), 7.06 (d, J = 8.1 Hz, 2 H), 6.74 (t, J = 7.2 Hz, 1 H), 6.65 (d, J = 7.8 Hz, 2 H), 3.77 (s, 3 H), 3.50 – 3.42 (m, 4 H), 3.30 (s, 0.07 H), 3.23 (d, J = 13.3 Hz, 1 H), 2.81 (t, J = 7.0 Hz, 0.11 H); ^{13}C NMR (126 MHz, $CDCl_3$) δ 167.93, 166.83, 147.59, 133.22, 131.37, 130.23, 129.31, 121.50, 118.07, 113.12, 53.04, 52.57, 42.22, 41.84, 35.19, 34.88 (t, $J_{C\text{-}D}$ = 24.6 Hz), 30.20, 29.90 (t, $J_{C\text{-}D}$ = 24.6 Hz); HRMS calcd for $C_{20}H_{19}D_2BrNO_4$ ($[M+H]^+$): 420.0774; found: 420.0782.

The following signals are discernible for the *cis*-isomer: ^{1}H NMR (500 MHz, $CDCl_3$) δ 3.79 (s, 3 H), 3.67 (s, 3 H).

(34) Dimethyl (2*R,3*S**)-2-(4-fluorophenyl)-3-((phenylamino)methyl) cyclopropane-1,1-dicarboxylate-2,3-*d*$_2$ (34) (wyq-5-37).**

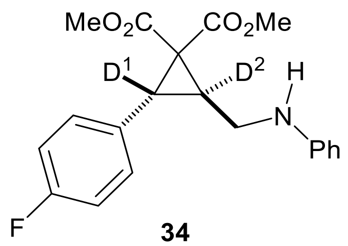

34

trans:cis = 9:1, D^1, D^2 = 95%, 95%

Following General Procedure I, **34** was afforded as a colorless oil (28.1 mg, 79%, D^1/D^2 = 95%/95%, *trans:cis* = 9:1), eluent (0-8% of ethyl acetate in petroleum ether).

The following signals are discernible for the *trans*-isomer: ^{1}H NMR (500 MHz, $CDCl_3$) δ 7.22 – 7.12 (m, 4 H), 6.95 (t, J = 8.5 Hz, 2 H), 6.73 (t, J = 7.2 Hz, 1 H), 6.64 (d, J = 8.0 Hz, 2 H), 3.76 (s, 3 H), 3.47 (d, J = 13.3 Hz, 1 H), 3.43 (s, 3 H), 3.31 (s, 0.05 H), 3.23 (d, J = 13.3 Hz, 1 H), 2.81 (t, 0.05 H); ^{13}C NMR (126 MHz, $CDCl_3$) δ 168.03, 166.93, 162.11 (d, $J_{C\text{-}F}$ = 246.4 Hz), 147.66, 130.17 (d, $J_{C\text{-}F}$ = 8.2 Hz), 129.86 (d, $J_{C\text{-}F}$ = 3.3 Hz), 129.30, 118.00, 115.16 (d, $J_{C\text{-}F}$ = 21.7 Hz), 113.08, 53.00, 52.47, 42.22, 41.78, 35.09, 34.79 (t, $J_{C\text{-}D}$ = 23.9 Hz), 30.33, 30.03 (t, $J_{C\text{-}D}$ = 25.2 Hz); ^{19}F NMR (471 MHz, $CDCl_3$) δ -114.60; HRMS calcd for $C_{20}H_{19}D_2FNO_4$ ($[M+H]^+$): 360.1575; found: 360.1582.

The following signals are discernible for the *cis*-isomer: ^{1}H NMR (500 MHz, $CDCl_3$) δ 3.78 (s, 3 H), 3.66 (s, 3 H); ^{13}C NMR (126 MHz, $CDCl_3$) δ 131.44 (d, $J_{C\text{-}F}$ = 8.2 Hz).

(35) Dimethyl (2*R,3*S**)-2-phenyl-3-((phenyl(phenylmethyl-d)amino)methyl)cyclopropane-1,1-dicarboxylate-2,3-*d*$_2$ (35) (wyq-9-8).**

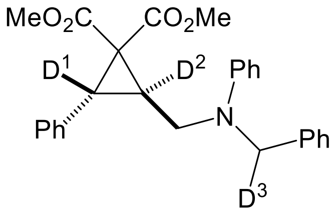

35

trans:cis > 20:1, D^1, D^2, D^3 = 65%, 97%, 43%

Following General Procedure III, **35** was afforded as a colorless oil (37.0 mg, 85%, $D^1/D^2/D^3$ = 65%/95%/43%, *trans:cis* > 20:1), eluent (0-10% of ethyl acetate in petroleum ether).

The following signals are discernible for the *trans*-isomer: ^{1}H NMR (400 MHz, $CDCl_3$) δ 7.28 – 7.22 (m, 2 H), 7.27 – 7.17 (m, 8 H), 6.82 (d, *J* = 8.0 Hz, 2 H), 6.74 (t, *J* = 7.2 Hz, 1 H), 4.61 (d, *J* = 7.4 Hz, 1.57 H), 3.73 (d, *J* = 15.6 Hz, 1 H), 3.68 (s, 3 H), 3.53 (d, *J* = 15.3 Hz, 1 H), 3.36 (s, 3 H), 3.25 (s, 0.35 H), 2.88 (t, *J* = 6.3 Hz, 0.03 H); ^{13}C NMR (126 MHz, $CDCl_3$) δ 168.27, 166.88, 148.50, 138.36, 134.13, 129.32, 128.51, 128.40, 128.17, 127.38, 126.81, 126.59, 117.16, 113.03, 54.45, 54.12 (t, $J_{C\text{-}D}$ = 21.4 Hz), 52.80, 52.29, 48.47, 41.85, 41.80, 36.67, 36.36 (t, $J_{C\text{-}D}$ = 23.7 Hz), 28.82, 28.53 (t, $J_{C\text{-}D}$ = 23.5 Hz); HRMS calcd for $C_{27}H_{26}D_3NO_4$ ($[M+H]^+$): 433.2201; found: 433.2190.

The following signals are discernible for the *cis*-isomer: ^{1}H NMR (400 MHz, $CDCl_3$) δ 3.76 (s, 3 H), 3.59 (s, 3 H).

(36) Dimethyl (2*S,3*R**)-2-(((2-((2-(4-isobutylphenyl)propanoyl)oxy)ethyl)(phenyl)amino)methyl)-3-phenylcyclopropane-1,1-dicarboxylate-2,3-*d*$_2$ (36) (wyq-4-45).**

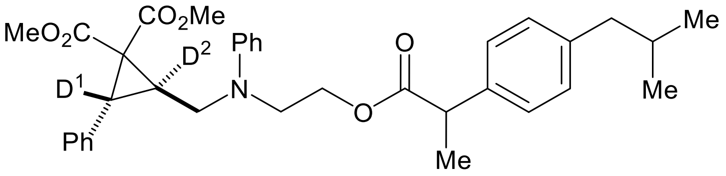

36

trans:cis > 20:1, D^1, D^2 = 94%, 80%

Following General Procedure I, **36** was afforded as a colorless oil (40.1 mg, 70%, D^1/D^2 = 94%/80%, *trans:cis* > 20:1), eluent (0-9% of ethyl acetate in petroleum ether); ^{1}H NMR (500 MHz, $CDCl_3$) δ 7.27 – 7.18 (m, 5 H), 7.18 – 7.08 (m, 4 H), 7.09 – 7.03 (m, 2 H), 6.81 (dd, *J* = 8.5, 2.7 Hz, 2 H), 6.75 (t, *J* = 7.3 Hz, 1 H), 4.28 – 4.13 (m, 2 H), 3.74 (d, *J* = 7.0 Hz, 3 H), 3.67 – 3.60 (m, 1 H), 3.57 (q, *J* = 6.0 Hz, 2 H), 3.50 (dd, *J* = 15.2, 4.7 Hz, 1 H), 3.45 – 3.38 (m, 1 H), 3.35 (d, *J* = 1.8 Hz, 3 H), 3.27 (s, 0.06 H), 2.78 (q, *J* = 6.0 Hz, 0.2 H), 2.42 (d, *J* = 7.2 Hz, 2 H), 1.83 (dt, *J* = 13.4, 6.9 Hz, 1 H), 1.46 – 1.39 (m, 3 H), 0.89 (dd, *J* = 6.5, 1.5 Hz, 6 H); ^{13}C NMR (126 MHz, $CDCl_3$) δ 174.61, 168.30, 166.87, 147.69, 140.52, 137.58, 134.16, 129.40, 129.31, 128.41, 128.21, 127.42, 127.13, 117.48, 113.21, 113.18, 61.79, 52.85, 52.28, 48.87, 48.83, 48.40, 48.37, 44.99, 41.85, 36.47, 36.16 (t, $J_{C\text{-}D}$ = 23.8 Hz), 30.11, 28.96, 28.89, 28.60 (t, $J_{C\text{-}D}$ = 23.2

Hz), 22.35, 18.43, 18.40; HRMS calcd for $C_{35}H_{40}D_2NO_6$ ($[M+H]^+$): 574.3132; found: 574.3137.

(37) Dimethyl (2*S,3*R**)-2-(((2-((5-(2,5-dimethylphenoxy)-2,2-dimethylpentanoyl)oxy)ethyl)(phenyl)amino)methyl)-3-phenylcyclopropane-1,1-dicarboxylate-2,3-*d*₂ (37) (wyq-4-46).**

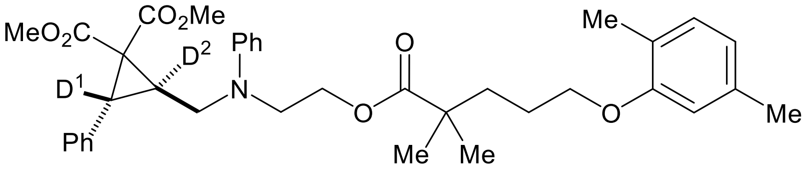

37

trans:cis = 15:1, D[1], D[2] = 94%, 70%

Following General Procedure I, **37** was afforded as a colorless oil (46.3 mg, 75%, D^1/D^2 = 94%/70%, *trans:cis* = 15:1), eluent (0-9% of ethyl acetate in petroleum ether).

The following signals are discernible for the *trans*-isomer: ^{1}H NMR (500 MHz, $CDCl_3$) δ 7.27 – 7.17 (m, 5 H), 7.11 (d, *J* = 8.0 Hz, 2 H), 6.98 (d, *J* = 7.4 Hz, 1 H), 6.87 (d, *J* = 8.2 Hz, 2 H), 6.75 (t, *J* = 7.3 Hz, 1 H), 6.64 (d, *J* = 7.3 Hz, 1 H), 6.58 (s, 1 H), 4.22 (t, *J* = 6.3 Hz, 2 H), 3.88 – 3.82 (m, 2 H), 3.77 (s, 3 H), 3.66 – 3.59 (m, 3 H), 3.49 (d, *J* = 15.2 Hz, 1 H), 3.36 (s, 3 H), 3.31 (s, 0.06 H), 2.81 (t, *J* = 6.3 Hz, 0.3 H), 2.29 (s, 3 H), 2.16 (s, 3 H), 1.67 (d, *J* = 3.0 Hz, 4 H), 1.18 (s, 6 H); ^{13}C NMR (126 MHz, $CDCl_3$) δ 177.66, 168.27, 166.84, 156.92, 147.81, 136.38, 134.17, 130.25, 129.42, 128.42, 128.20, 127.42, 123.53, 120.68, 117.57, 113.33, 112.01, 67.88, 61.57, 52.84, 52.26, 48.97, 48.46, 42.01, 41.78, 37.06, 36.60, 36.36 (t, J_{C-D} = 22.7 Hz), 28.90, 28.60 (t, J_{C-D} = 23.3 Hz), 25.06, 21.34, 15.70; HRMS calcd for $C_{37}H_{44}D_2NO_7$ ($[M+H]^+$): 618.3394; found: 618.3399.

The following signals are discernible for the *cis*-isomer: ^{1}H NMR (500 MHz, $CDCl_3$) δ 3.75 (s, 3 H), 3.66 (s, 3 H), 1.14 (s, 6 H).

(38) Dimethyl (2*S,3*R**)-2-(((2-(2-(adamantan-1-yl)acetoxy)ethyl)(phenyl)amino)methyl)-3-phenylcyclopropane-1,1-dicarboxylate-2,3-*d*₂ (38) (wyq-4-39).**

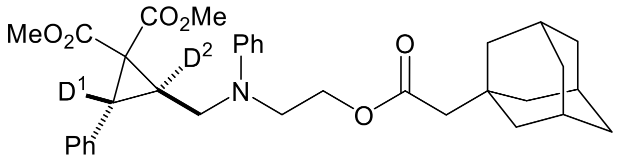

38

trans:cis = 18:1, D^1, D^2 = 95%, 90%

Following General Procedure I, **38** was afforded as a colorless oil (41.5 mg, 74%, D^1/D^2 = 95%/90%, *trans:cis* = 18:1), eluent (0-10% of ethyl acetate in petroleum ether).

The following signals are discernible for the *trans*-isomer: ^{1}H NMR (500 MHz, $CDCl_3$) δ 7.28 – 7.19 (m, 5 H), 7.15 – 7.09 (m, 2 H), 6.86 (d, *J* = 8.1 Hz, 2 H), 6.75 (t, *J* = 7.3 Hz, 1 H), 4.21 (t, *J* = 6.4 Hz, 2 H), 3.78 (s, 3 H), 3.66 – 3.59 (m, 3 H), 3.49 (d, *J* = 15.2 Hz, 1 H), 3.36 (s, 3 H), 3.31 (s, 0.05 H), 2.81 (t, *J* = 6.3 Hz, 0.1 H), 2.03 (s, 2 H), 1.94 (s, 3 H), 1.71 – 1.66 (m, 3 H), 1.62 – 1.56 (m, 9 H); ^{13}C NMR (126 MHz, $CDCl_3$) δ 171.55, 168.29, 166.86, 147.83, 134.20, 129.41, 128.44, 128.20, 127.41, 117.58, 113.36, 60.99, 52.84, 52.26, 49.12, 48.76, 48.71, 42.36, 41.77, 36.81, 36.69, 36.40 (t, J_{C-D} = 23.9 Hz), 32.67, 28.90, 28.54 (t, J_{C-D} = 20.2 Hz), 28.38; HRMS calcd for $C_{34}H_{40}D_2NO_6$ ($[M+H]^+$): 562.3132; found: 562.3137.

The following signals are discernible for the *cis*-isomer: ^{1}H NMR (500 MHz, $CDCl_3$) δ 3.76 (s, 3 H), 3.66 (s, 3 H); ^{13}C NMR (126 MHz, $CDCl_3$) δ 129.01, 113.03.

(39) Dimethyl (2*S,3*R**)-2-(((2-((4-((3*R**,10*S**,13*R**)-3-acetoxy-10,13-dimethylhexadecahydro-1H-cyclopenta[a]phenanthren-17-yl)pentanoyl)oxy) ethyl)(phenyl)amino)methyl)-3-phenylcyclopropane-1,1-dicarboxylate-2,3-*d*$_2$ (39) (wyq-7-47).**

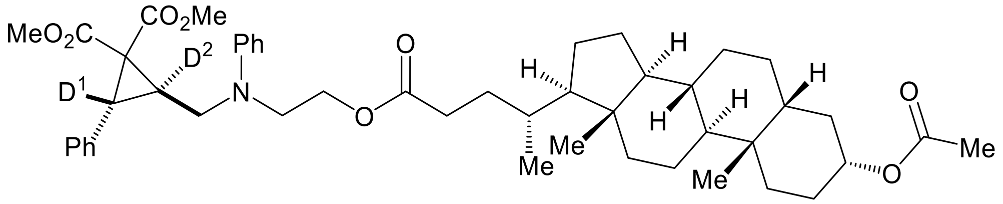

39

trans:cis > 20:1, D^1, D^2 = 95%, 82%

Following General Procedure II, **39** was afforded as a colorless oil (47.7 mg, 62%, D^1/D^2 = 95%/82%, *trans:cis* > 20:1), eluent (0-12% of ethyl acetate in petroleum ether); ^{1}H NMR (500 MHz, $CDCl_3$) δ 7.27 (s, 1 H), 7.26 – 7.19 (m, 4 H), 7.13 (d, *J* = 6.3 Hz,

2 H), 6.86 (d, J = 8.1 Hz, 2 H), 6.76 (t, J = 7.2 Hz, 1 H), 4.72 (tt, J = 11.3, 4.8 Hz, 1 H), 4.29 – 4.17 (m, 2 H), 3.79 (s, 3 H), 3.66 – 3.56 (m, 3 H), 3.48 (d, J = 15.2 Hz,1 H), 3.37 (s, 3 H), 3.28 (s, 0.05 H), 2.82 (t, J = 6.3 Hz, 0.18 H), 2.30 – 2.24 (m, 1 H), 2.20 – 2.09 (m, 1 H), 2.03 (s, 3 H), 1.95 (d, J = 12.4 Hz, 1 H), 1.82 (t, J = 12.3 Hz, 4 H), 1.75 – 1.65 (m, 2 H), 1.54 (d, J = 13.5 Hz, 3 H), 1.47 – 1.38 (m, 6 H), 1.31 – 1.22 (m, 5 H), 1.13 (t, J = 12.9 Hz, 3 H), 1.05 (t, J = 10.1 Hz, 3 H), 0.93 (s, 3 H), 0.88 (d, J = 6.5 Hz, 3 H), 0.63 (s, 3 H); ^{13}C NMR (126 MHz, $CDCl_3$) δ 174.17, 170.65, 168.33, 166.90, 147.84, 134.18, 129.45, 128.46, 128.24, 127.46, 117.53, 113.21, 74.40, 61.31, 56.48, 55.95, 52.91, 52.34, 49.08, 48.55, 42.72, 41.89, 40.41, 40.13, 36.41, 36.12 (t, J_{C-D} = 22.7 Hz), 35.78, 35.31, 35.03, 34.58, 32.25, 31.10, 30.87, 28.87, 28.59 (t, J_{C-D} = 23.9 Hz), 28.17, 27.01, 26.63, 26.31, 24.16, 23.32, 21.46, 20.82, 18.26, 12.03; HRMS calcd for $C_{48}H_{64}D_2NO_8$ ($[M+H]^+$): 786.4908; found: 786.4916.

3. Synthetic applications.

3.1 Transformation of 1.

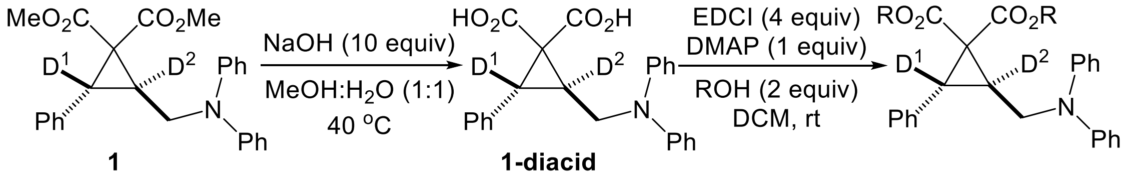

General Procedure IV: To a round bottom flask were added **1** (0.1 mmol), NaOH (1 mmol), MeOH (1.0 ml), and H_2O (1.0 mL). The mixture was stirred at 40 °C overnight until the completion of the reaction as monitored by TLC. The aqueous layer was extracted with dichloromethane (5 mL × 3) and the combined organic layer was washed with brine (5 mL), dried over anhydrous $MgSO_4$, filtered and concentrated in vacuo to afford corresponding **1-diacid**, which was used directly for the following step. To a round bottom flask were added **1-diacid**, alcohol (0.2 mmol), EDCI (0.4 mmol), DMAP (0.1 mmol), and dichloromethane (1.5 mL). The mixture was stirred at room temperature overnight until the completion of the reaction as monitored by TLC. Then the mixture was concentrated in vacuo to afford corresponding product via chromatography on silica gel.

(1) Bis(benzo[d][1,3]dioxol-5-ylmethyl) (2*S,3*R**)-2-((diphenylamino)methyl)-3-phenylcyclopropane-1,1-dicarboxylate-2,3-d_2 (40) (wyq-9-34).**

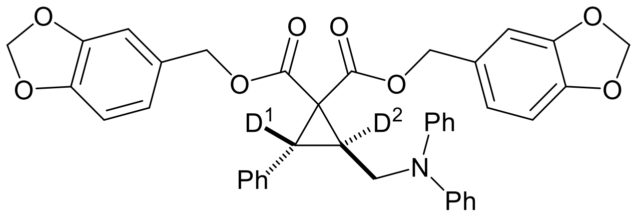

40 (D^1, D^2 = 94%, 95%)

Following General Procedure IV, **40** was afforded as a colorless oil (34.3 mg, 52% for two steps, D^1/D^2 = 94%/95%), eluent (0-25% of ethyl acetate in petroleum ether); ^{1}H NMR (500 MHz, $CDCl_3$) δ 7.24 (t, *J* = 7.8 Hz, 4 H), 7.18 – 7.13 (m, 3 H), 7.03 – 6.90 (m, 8 H), 6.75 – 6.70 (m, 3 H), 6.62 (d, *J* = 7.9 Hz, 1 H), 6.42 (dd, *J* = 7.9, 1.7 Hz, 1 H), 6.34 (d, *J* = 1.8 Hz, 1 H), 5.93 (d, *J* = 8.7 Hz, 4 H), 5.05 (d, *J* = 12.0 Hz, 1 H), 4.77 (d, *J* = 12.1 Hz, 1 H), 4.62 (q, *J* = 11.9 Hz, 2 H), 3.98 (d, *J* = 15.1 Hz, 1 H), 3.80 (d, *J* = 15.1 Hz, 1 H), 3.17 (s, 0.06 H), 2.88 (t, *J* = 6.6 Hz, 0.05 H); ^{13}C NMR (126 MHz, $CDCl_3$) δ 167.56, 166.20, 147.70, 147.63, 147.46, 133.96, 129.39, 129.04, 128.87, 128.45, 128.10, 127.31, 122.27, 122.16, 121.79, 121.34, 109.03, 108.88, 108.13, 107.91, 101.13, 101.02, 67.45, 67.13, 49.05, 42.15, 37.04, 36.57 (t, $J_{C\text{-}D}$ = 28.4 Hz), 29.65, 28.95 (t, $J_{C\text{-}D}$ = 28.7 Hz); HRMS calcd for $C_{40}H_{31}D_2NO_8$ ($[M+H]^+$): 658.2404; found: 658.2420.

(2) Bis(2-(1-(4-chlorobenzoyl)-5-methoxy-2-methyl-1H-indol-3-yl)ethyl) (2*S,3*R**)-2-((diphenylamino)methyl)-3-phenylcyclopropane-1,1-dicarboxylate-2,3-d_2 (41) (wyq-9-37).**

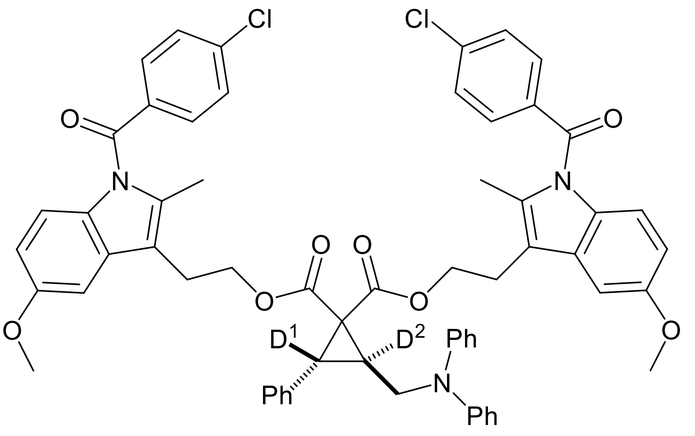

41 (D[1], D[2] = 94%, 95%)

Following General Procedure IV, **41** was afforded as a pale green oil (44.4 mg, 47% for two steps, D^1/D^2 = 94%/95%), eluent (0-25% of ethyl acetate in petroleum ether); ^{1}H NMR (500 MHz, $CDCl_3$) δ 7.57 (d, *J* = 8.2 Hz, 2 H), 7.53 (d, *J* = 8.2 Hz, 2 H), 7.42 (d, *J* = 8.2 Hz, 2 H), 7.36 (d, *J* = 8.2 Hz, 2 H), 7.27 – 7.17 (m, 5 H), 7.12 – 7.06 (m, 3 H), 7.00 – 6.91 (m, 7 H), 6.90 (d, *J* = 2.6 Hz, 1 H), 6.81 (t, *J* = 9.2 Hz, 2 H), 6.73 (d, *J* = 2.6 Hz, 1 H), 6.63 (d, *J* = 9.0 Hz, 2 H), 4.23 – 4.09 (m, 2 H), 3.89 (d, *J* = 15.1 Hz, 1 H), 3.82 (s, 3 H), 3.82 – 3.77 (m, 4 H), 3.79 – 3.72 (m, 1 H), 3.69 – 3.61 (m, 1 H), 3.16 (s, 0.06 H), 2.98 – 2.76 (m, 2.05 H), 2.36 – 2.21 (m, 5 H), 2.15 (s, 3 H); ^{13}C NMR (126 MHz, $CDCl_3$) δ 168.14, 168.11, 167.73, 166.21, 156.02, 155.95, 147.65, 139.11, 139.09, 135.35, 135.17, 134.03, 133.91, 133.84, 131.03, 131.02, 130.83, 130.79, 130.64, 129.40, 129.05, 129.03, 128.45, 128.07, 127.38, 121.85, 121.32, 115.05, 114.99, 114.90, 114.74, 111.36, 111.22, 101.16, 64.93, 63.95, 55.70, 55.67, 48.94, 42.24, 36.28, 35.98 (t, $J_{C\text{-}D}$ = 27.2 Hz), 29.67, 28.56 (t, $J_{C\text{-}D}$ = 24.8 Hz), 23.33, 22.76, 13.21, 12.98; HRMS calcd for $C_{62}H_{52}D_2Cl_2N_3O_8$ ($[M+H]^+$): 1040.3408; found: 1040.3410.

3.2 Synthesis and characterization of aggregation-induced emission (AIE) compounds.

(1) Synthesis of Dimethyl (2*R,3*S**)-2-phenyl-3-((phenyl(2-((4-(1,2,2-triphenylvinyl) benzoyl)oxy)ethyl)amino)methyl)cyclopropane-1,1-dicarboxylate-2,3-*d*₂ (42) (wyq-5-8).**

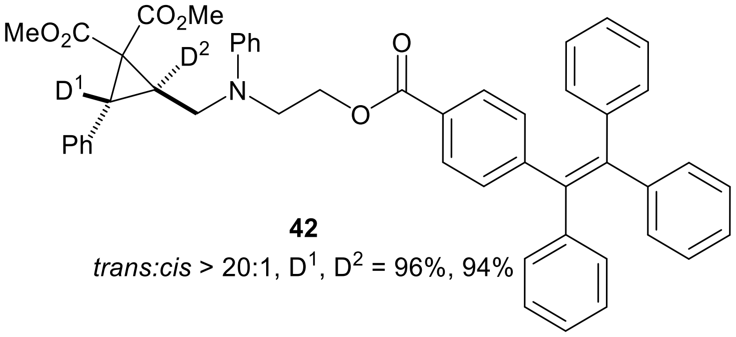

42

trans:cis > 20:1, D^1, D^2 = 96%, 94%

Following General Procedure I, **42** was afforded as a colorless oil (57.2 mg, 75%, D^1/D^2 = 96%/94%, *trans:cis* > 20:1), eluent (0-11% of ethyl acetate in petroleum ether); ^{1}H NMR (500 MHz, $CDCl_3$) δ 7.70 (d, J = 8.1 Hz, 2 H), 7.27 – 7.21 (m, 3 H), 7.19 (d, J = 5.6 Hz, 2 H), 7.12 – 7.05 (m, 12 H), 7.04 – 6.98 (m, 7 H), 6.89 (d, J = 8.2 Hz, 2 H), 6.74 (t, J = 7.3 Hz, 1 H), 4.42 (t, J = 6.3 Hz, 2 H), 3.78 – 3.71 (m, 5 H), 3.63 (d, J = 7.7 Hz, 1 H), 3.52 (d, J = 15.1 Hz, 1 H), 3.35 (s, 3 H), 3.27 (s, 0.04 H), 2.83 (t, J = 6.3 Hz, 0.06 H); ^{13}C NMR (126 MHz, $CDCl_3$) δ 168.28, 166.85, 166.31, 148.90, 147.79, 143.16, 143.06, 143.03, 142.44, 139.86, 134.09, 131.27, 131.21, 131.19, 129.45, 129.03, 128.41, 128.20, 127.82, 127.79, 127.66, 127.59, 127.41, 126.84, 126.67, 117.56, 113.27, 62.04, 52.88, 52.29, 49.07, 48.59, 41.73, 36.69, 36.36 (t, $J_{C\text{-}D}$ = 23.9 Hz), 28.88, 28.59 (t, $J_{C\text{-}D}$ = 23.3 Hz); HRMS calcd for $C_{49}H_{42}D_2NO_6$ ($[M+H]^+$): 744.3289; found: 744.3288.

(2) Characterization of aggregation-induced emission (AIE) properties of S7 and 42.

Stock solutions of compounds were prepared in THF: **S7** (1 M), **42** (1 M). Samples for fluorescence measurement (8 μM) were prepared by diluting the stock solutions with a mixture of THF and water at different ratios. All fluorescence emission spectra were measured with the exciting wavelength of 450 nm.

TEM images were measured in a mixture of water and THF (95/5) (Figure S1).

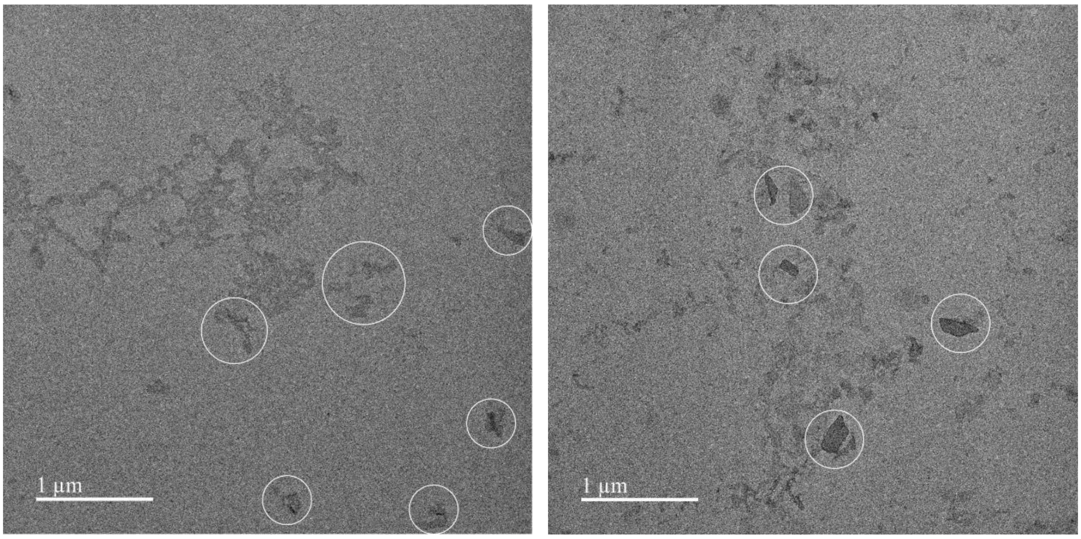

Figure S1. TEM images of **S7** (left) and **42** (right) in water/THF (95/5).

4. Mechanistic investigations.

4.1 Trap of the radical intermediate with TEMPO.

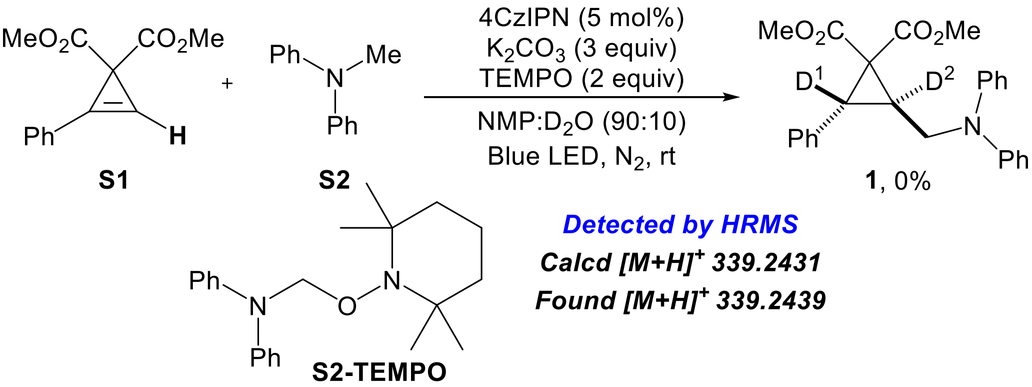

Under nitrogen atmosphere, to a 10 mL Schlenk tube equipped with a stirring bar is added 4CzIPN (5 mol%), K_2CO_3 (0.3 mmol), **S1** (0.1 mmol), **S2** (0.3 mmol), TEMPO (0.2 mmol), *N*-methyl pyrrolidinone (0.9 mL), and D_2O (0.1 mL) successively, and the tube was sealed with a septum. The mixture was stirred at room temperature overnight, and quenched with H_2O (2.5 mL). The aqueous layer was extracted with EtOAc (5 mL × 3) and the combined organic layer was washed with brine (5 mL), dried over anhydrous $MgSO_4$, filtered and concentrated in vacuo. The crude mixture was analyzed with ^{1}H NMR and HRMS and no product **1** was formed, instead, the adduct product **S2-TEMPO** was detected by HRMS: $C_{22}H_{31}N_2O^+$ ($[M+H]^+$) calcd: 339.2431; found: 339.2439 (Figure S2).

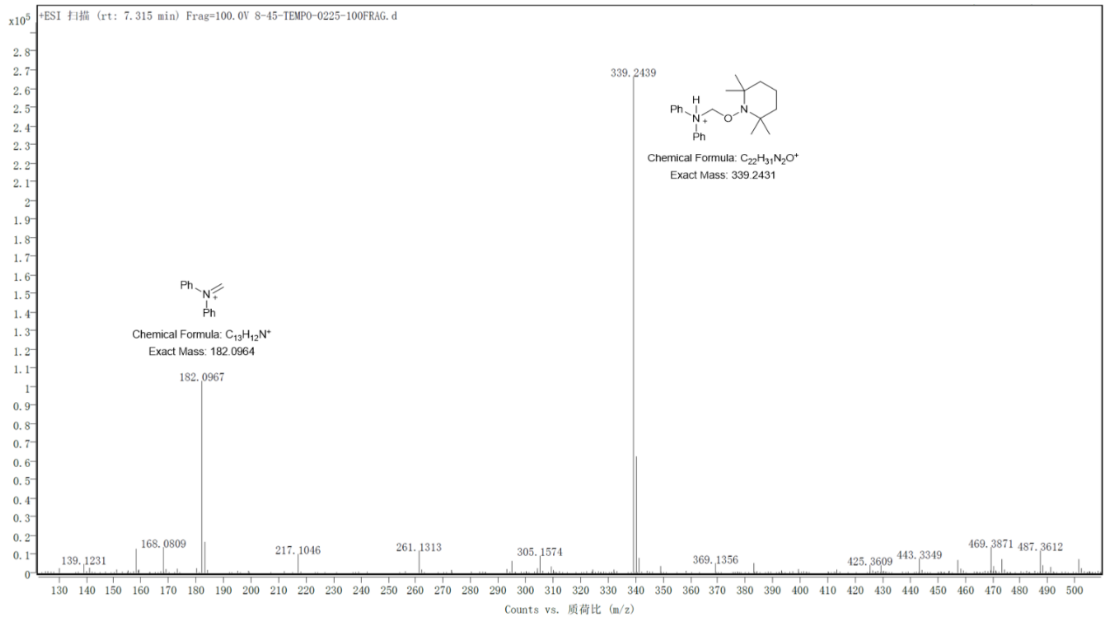

Figure S2. HRMS spectrum of **S2-TEMPO**.

4.2 Deuteration of S1 in the absence of S2.

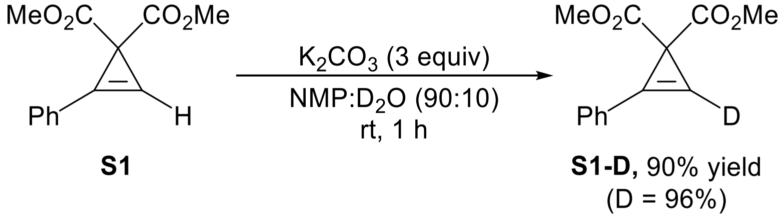

To a 10 mL Schlenk tube tube equipped with a stirring bar was added K_2CO_3 (0.3 mmol), **S1** (0.1 mmol), *N*-methyl pyrrolidinone (0.9 mL), and D_2O (0.1 mL) successively, and the tube was sealed with a septum. The mixture was stirred at room temperature for one hour. The residual was directly conducted chromatography on silica gel to afford **S1-D** as a white solid (22.6 mg, 90%, D = 96%) with eluent (0-15% of ethyl acetate in petroleum ether): ^{1}H NMR (500 MHz, $CDCl_3$) δ 7.63 (dd, J = 6.3, 2.9 Hz, 2 H), 7.47 – 7.41 (m, 3 H), 6.90 (s, 0.04 H), 3.73 (s, 6 H); ^{13}C NMR (126 MHz, $CDCl_3$) δ 171.12, 130.56, 130.32, 128.87, 123.91, 111.99, 95.23, 94.93 (t, J_{C-D} = 35.9 Hz), 52.37, 32.67; HRMS calcd for $C_{13}H_{12}DO_4$ ($[M+H]^+$): 234.0871; found: 234.0876.

4.3 Luminescence quenching studies of 4CzIPN with S1 and S2.

Stock solutions of compounds were prepared in *N*-methyl pyrrolidinone: 4CzIPN (10 μM), **S1** (1 M), and **S2** (1 M). All solutions with different concentrations of compounds were prepared by diluting these stock solutions with *N*-methyl

pyrrolidinone. Fluorescent quenching studies of 4CzIPN (10 μM) with different concentrations of **S1** and **S2** were performed with the exciting wavelength of 450 nm.

4.4 Determination of quantum yield:

The quantum yield of this reaction was determined according to a reported procedure.[7]

(1) Preparation of stock solutions:

A 0.15 M solution of potassium ferrioxalate was prepared by dissolving 1.11 g of potassium ferrioxalate hydrate in 15 mL of 0.05 M H_2SO_4. A buffered solution of phenanthroline was prepared by dissolving 10 mg of phenanthroline and 2.25 g of sodium acetate in 15 mL of 0.5 M H_2SO_4. Both solutions were stored in the dark.

(2) Determination of fraction of light absorbed at 456 nm for the ferrioxalate solution:

The absorbance (A) of the above ferrioxalate solution at 456 nm was measured to be 1.69 (Figure S3). The fraction of light absorbed (f) by this solution was calculated:

$$f = 1 - 10^{-A}$$

$$f = 1 - 10^{-1.69} = 0.98$$

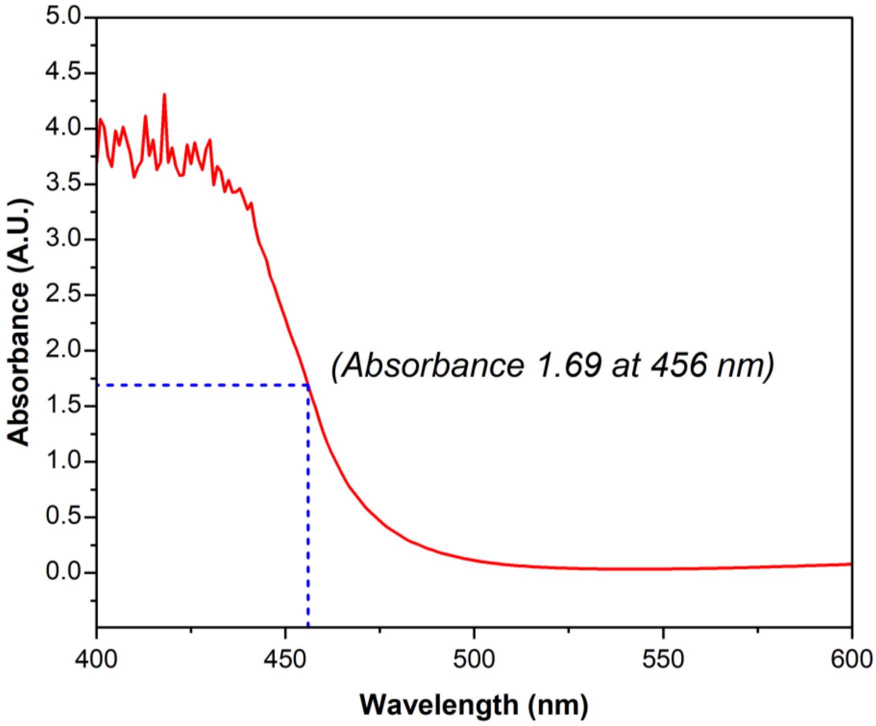

Figure S3. UV-vis spectrum of the potassium ferrioxalate solution.

(3) Determination of light intensity at 456 nm:

The photon flux of the photoreactor was determined by standard ferrioxalate actinometry. To determine the photon flux of the photoreactor, 1.0 mL of 0.15 M potassium ferrioxalate solution was placed in a quartz cuvette and was irradiated with blue LEDs for 10 seconds. After irradiation, 0.175 mL of the phenanthroline solution was added to the cuvette. The solution was then allowed to rest for 1 h, ensuring the complete coordination of phenanthroline to ferrous ions. The absorbance of the above solution was measured with a UV-vis spectrometer (Figure S4). A non-irradiated sample was also prepared and measured (Figure S5).

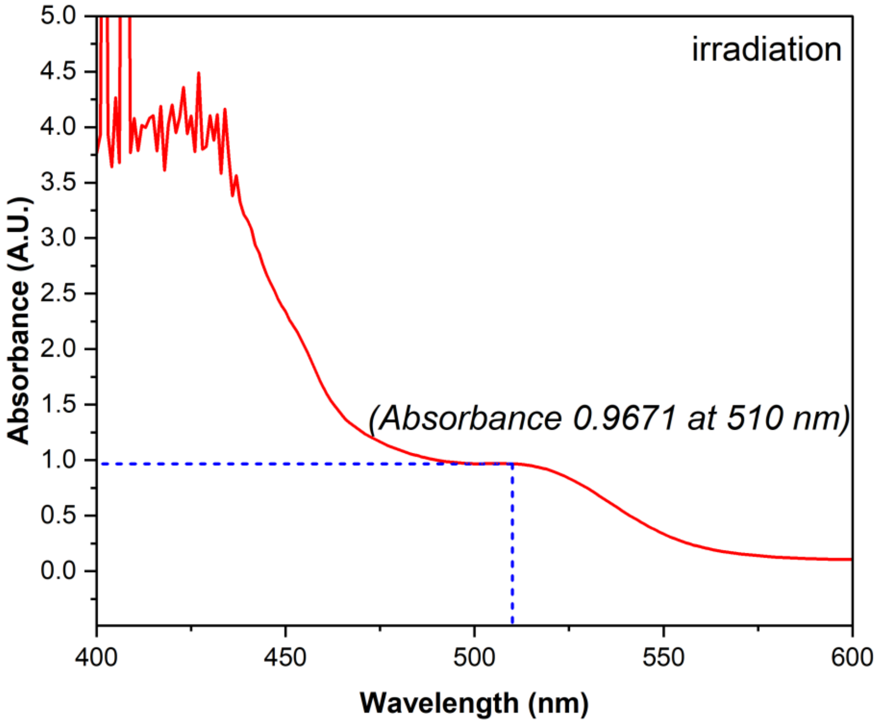

Figure S4. UV-vis spectrum of the potassium ferrioxalate-phenanthroline solution with irradiation.

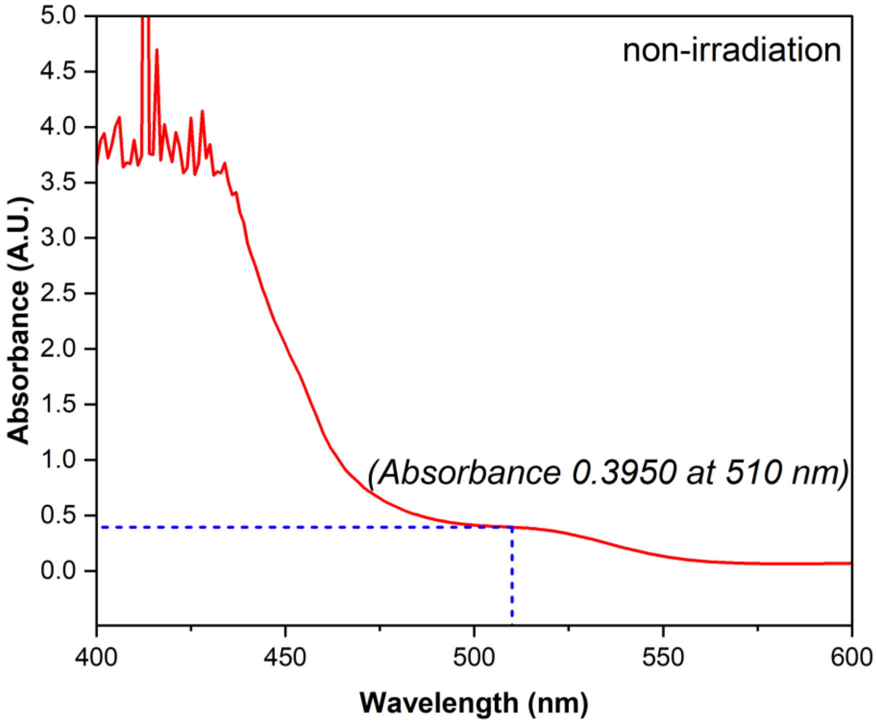

Figure S5. UV-vis spectrum of the potassium ferrioxalate-phenanthroline solution without irradiation.

The amount of Fe^{2+} formed during the irradiation has been calculated using the following equation:

$$\text{mol}\, Fe^{2+} = \frac{V \cdot \Delta A}{l \cdot \varepsilon}$$

Where V is the total volume (0.001175 L) of the solution after addition of phenanthroline, ΔA is the difference in absorbance at 510 nm between the irradiated and non-irradiated solutions ($A_{\text{irradiation}} = 0.9671$, $A_{\text{non-irradiation}} = 0.3950$, $\Delta A = 0.5721$), l is the path length (1.000 cm), and ε is the molar absorptivity at 510 nm (11,100 L/mol/cm). The photon flux has been calculated using the following equation

$$\text{photon flux} = \frac{\text{mol}\, Fe^{2+}}{\Phi \cdot t \cdot f}$$

Where Φ is the quantum yield for the ferrioxalate actinometer (0.92 for a 0.15 M solution at $\lambda = 456$ nm), t is the irradiation time (10 s), and f is the fraction of light absorbed at $\lambda = 456$ nm (0.98, *vide supra*). The photon flux of the photoreactor was calculated to be 6.7×10^{-9} einstein/s.

$$\text{mol}\, Fe^{2+} = \frac{V \cdot \Delta A}{l \cdot \varepsilon} = \frac{0.001175\ \text{L} \times 0.5721}{1.000\ \text{cm} \times 11100\ \text{L mol}^{-1} \cdot \text{cm}^{-1}} = 6.1 \times 10^{-8}\ \text{mol}$$

$$\text{photon flux} = \frac{\text{mol Fe}^{2+}}{\Phi \cdot t \cdot f} = \frac{6.1 \times 10^{-8}\ \text{mol}}{0.92 \times 10\text{s} \times 0.98} = 6.7 \times 10^{-9}\ \text{einstein} \cdot \text{s}^{-1}$$

(4) Determination of the quantum yield:

Under nitrogen atmosphere, to a 10 mL Schlenk tube equipped with a stirring bar were added 4CzIPN (4.1 mg, 0.005 mmol, 5 mol%), K_2CO_3 (41.9 mg, 0.3 mmol, 3 equiv), **S1** (24.0 mg, 0.1 mmol, 1 equiv), **S2** (55.1 mg, 0.3 mmol, 3 equiv), *N*-methyl pyrrolidinone (0.9 mL) and D_2O (0.1 mL) successively, and the tube was sealed with a septum. The mixture was stirred at room temperature irradiated with a blue LED for 1 h. The aqueous layer was extracted with ethyl acetate (5 mL × 3) and the combined organic layer was washed with brine (5 mL), dried over anhydrous $MgSO_4$, filtered and concentrated in vacuo to afford corresponding product via chromatography on silica gel to afford **1** (3.0 mg, 7%).

The quantum yield (Φ) has been calculated using the equation

$$\Phi = \frac{\text{mol product}}{\text{flux} \cdot t \cdot f}$$

Where *t* is the reaction time (3600 s), and *f* is the previously calculated fraction of light absorbed by the solution.

$$\Phi = \frac{\text{mol product}}{\text{flux} \cdot t \cdot f} = \frac{0.1 \times 0.07 \times 10^{-3}}{6.7 \times 10^{-9}\ \text{einstein} \cdot \text{s}^{-1} \times 3600\ \text{s} \times 0.98} = 0.30$$

5. References.

[1] L.-a. Liao, F. Zhang, N. Yan, J. A. Golen, J. M. Fox, *Tetrahedron.*, **2004**, *60*, 1803.

[2] S. Chuprakov, M. Rubin, V. Gevorgyan, *J. Am. Chem. Soc.*, **2005**, *127*, 3714.

[3] Y. Liu, Q. Yu, S. Ma, *Eur. J. Org. Chem.*, **2013**, 3033.

[4] F. Zhang, J. M. Fox, *Org. Lett.*, **2006**, *8*, 2965.

[5] Q. Ye, H. Ye, D. Cheng, X. Li, X. Xu, *Tetrahedron Lett.*, **2018**, *59*, 2546.

[6] C. Peng, F. Gu, X. Lin, N. Ding, Q. Zhan, P. Cao, T. Cao, *Green Chem.*, **2023**, *25*, 671.

[7] M. A. Cismesia, T. P. Yoon, *Chem. Sci.*, **2015**, *6*, 5426.

6. ^{1}H NMR, ^{13}C NMR, ^{19}F NMR spectra for compounds.

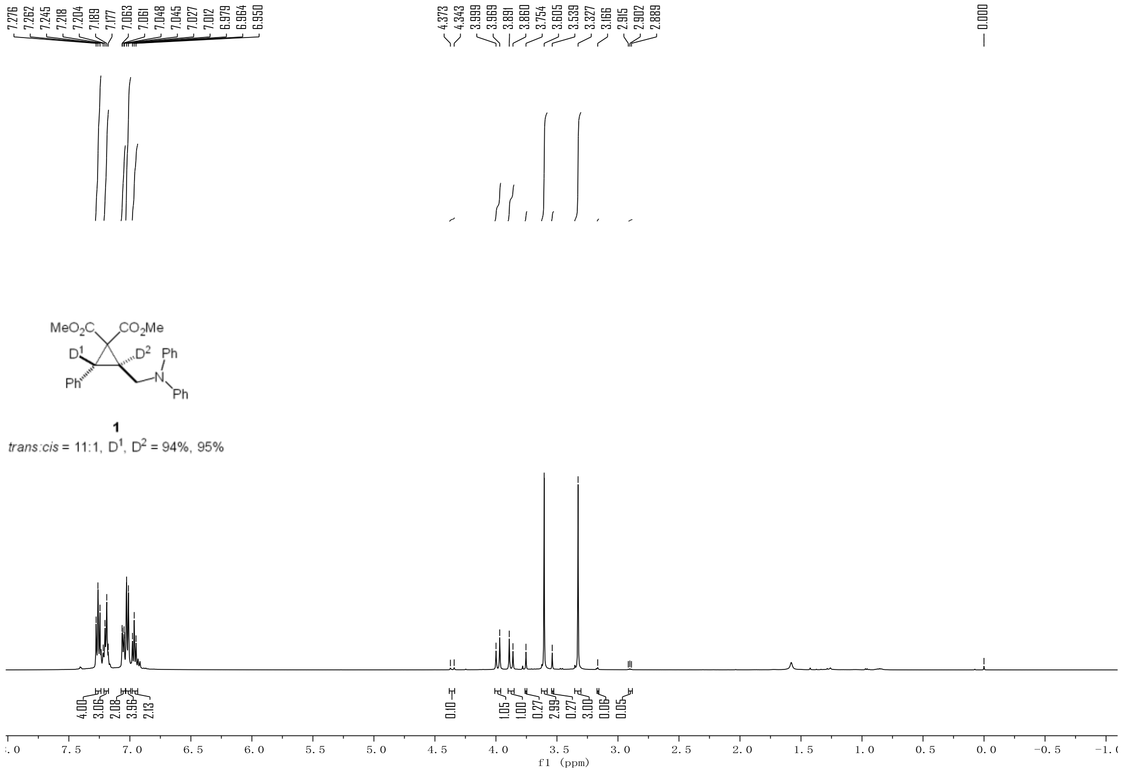

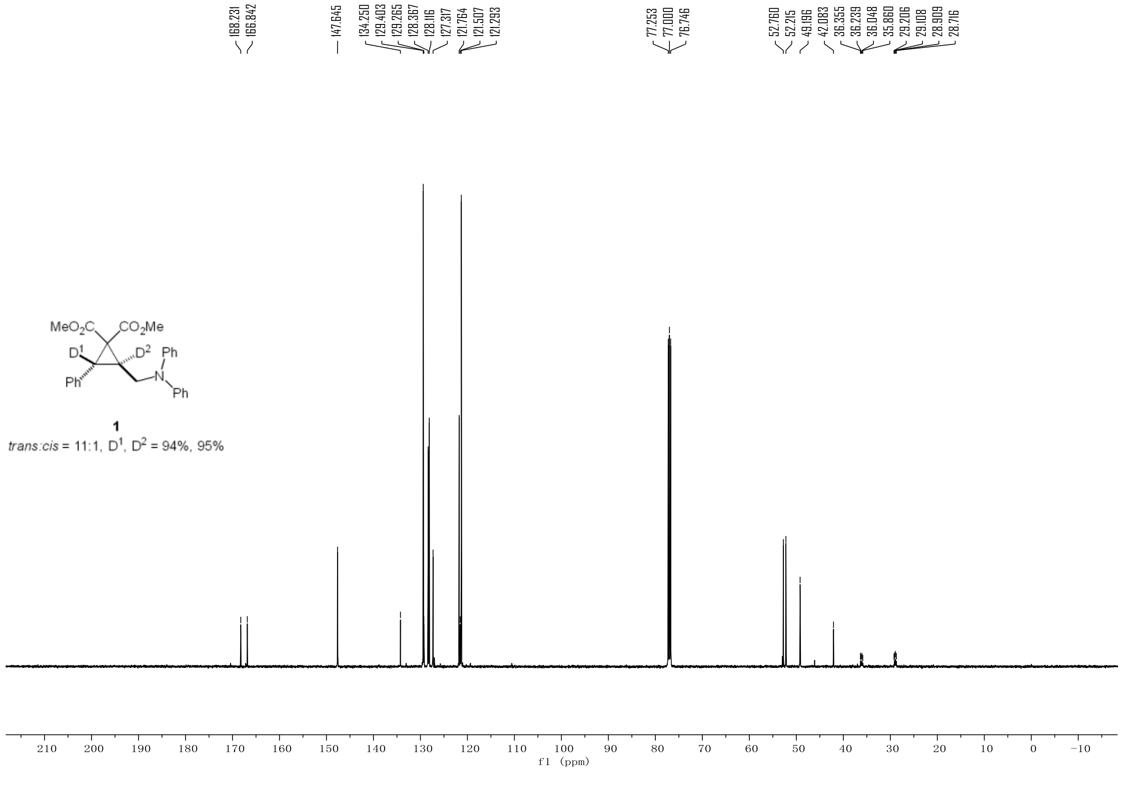

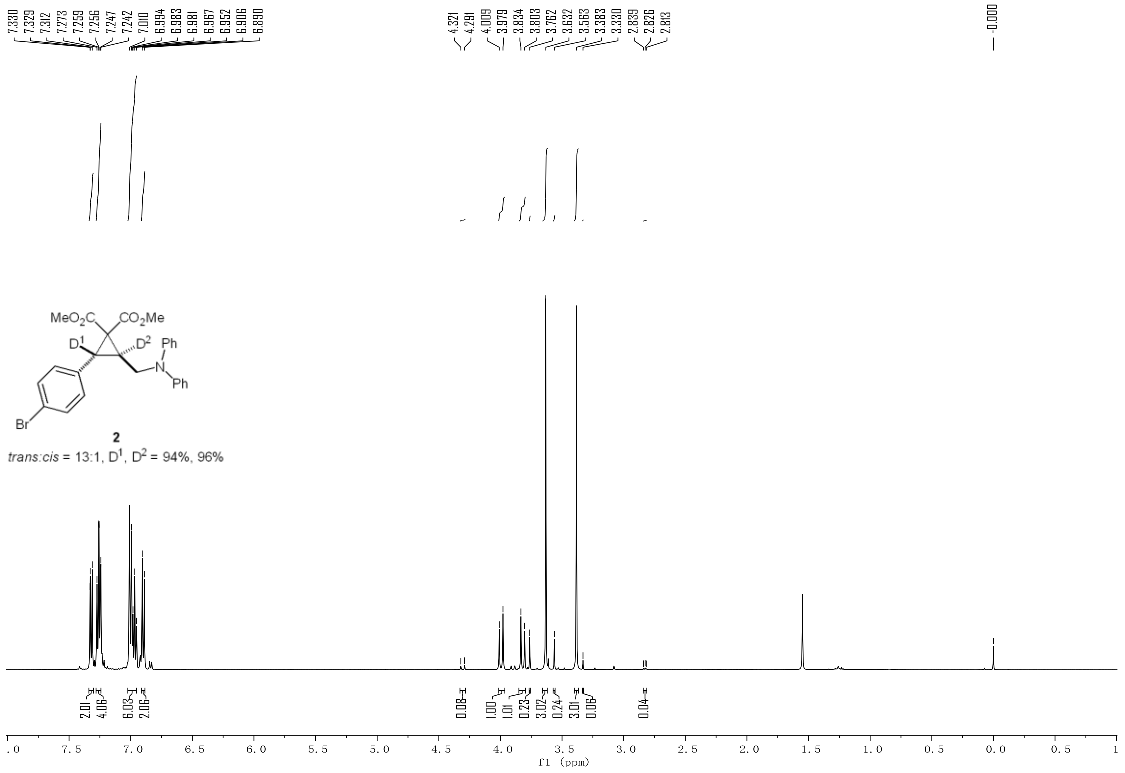
7.330 7.329 7.312 7.273 7.259 7.256 7.247 7.242 7.010 6.994 6.983 6.981 6.967 6.952 6.906 6.890
4.321 4.291 4.009 3.979 3.834 3.803 3.762 3.632 3.563 3.383 3.330 2.839 2.826 2.813
0.000
MeO2C
CO2Me
D1
D2
Ph
N
Ph
Br
2
trans:cis = 13:1, D1, D2 = 94%, 96%
2.01 4.06 6.03 2.06
0.08 1.00 1.01 0.23 3.02 0.24 3.01 0.06 0.04
f1 (ppm)

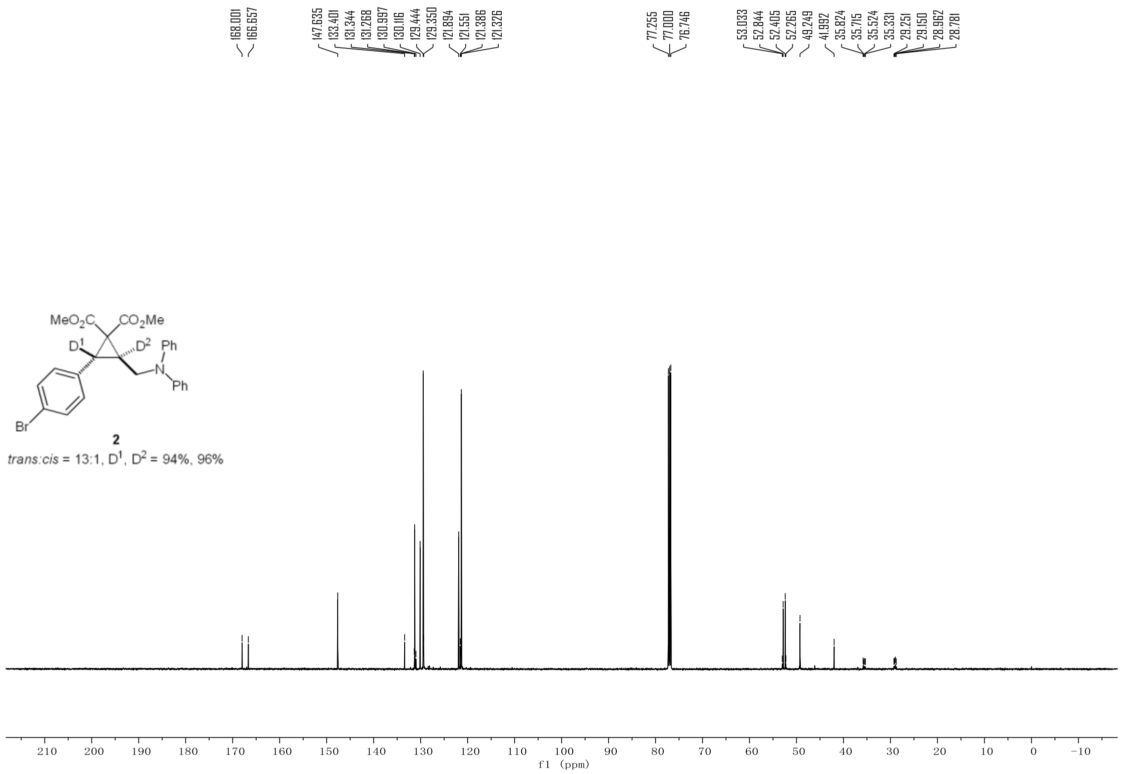
168.001 166.657
147.635 133.401 131.344 131.268 130.997 130.116 129.444 129.350 121.894 121.551 121.386 121.326
77.255 77.000 76.746
53.033 52.844 52.405 52.265 49.249 41.992 35.824 35.715 35.524 35.331 29.251 29.150 28.962 28.781
MeO2C
CO2Me
D1
D2
Ph
N
Ph
Br
2
trans:cis = 13:1, D1, D2 = 94%, 96%
f1 (ppm)

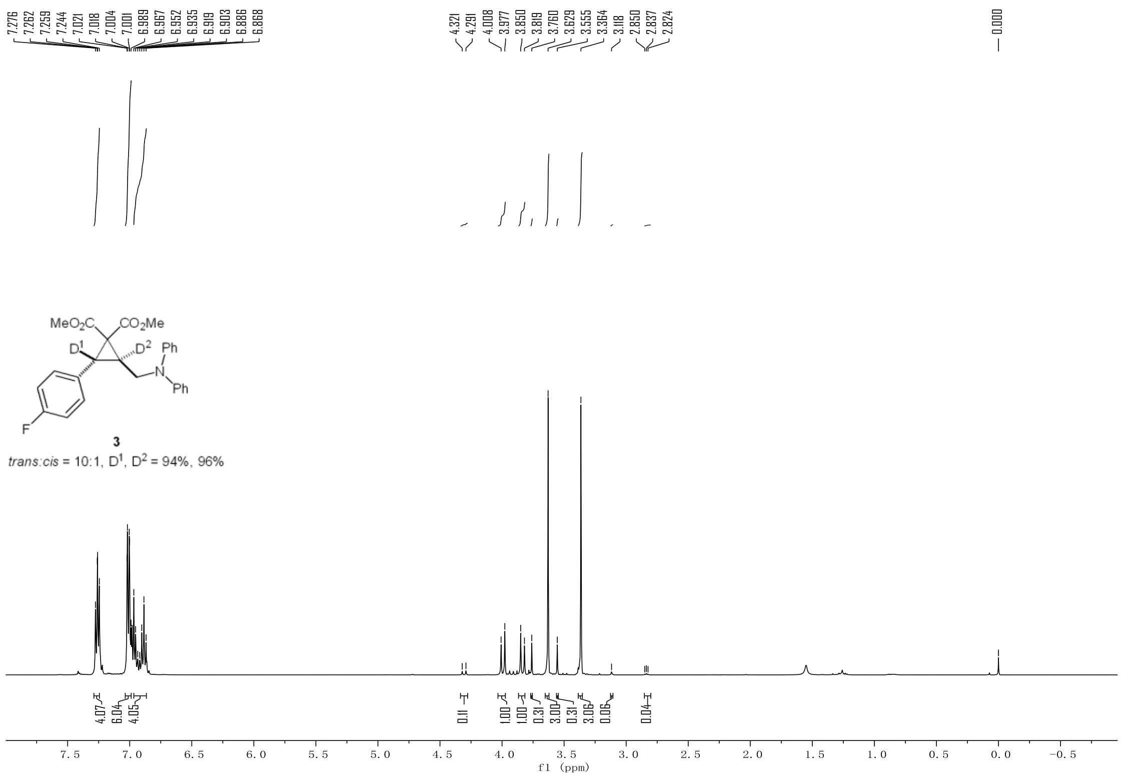

MeO2C CO2Me
D1 D2 Ph N Ph
F
3
trans:cis = 10:1, D1, D2 = 94%, 96%
f1 (ppm)

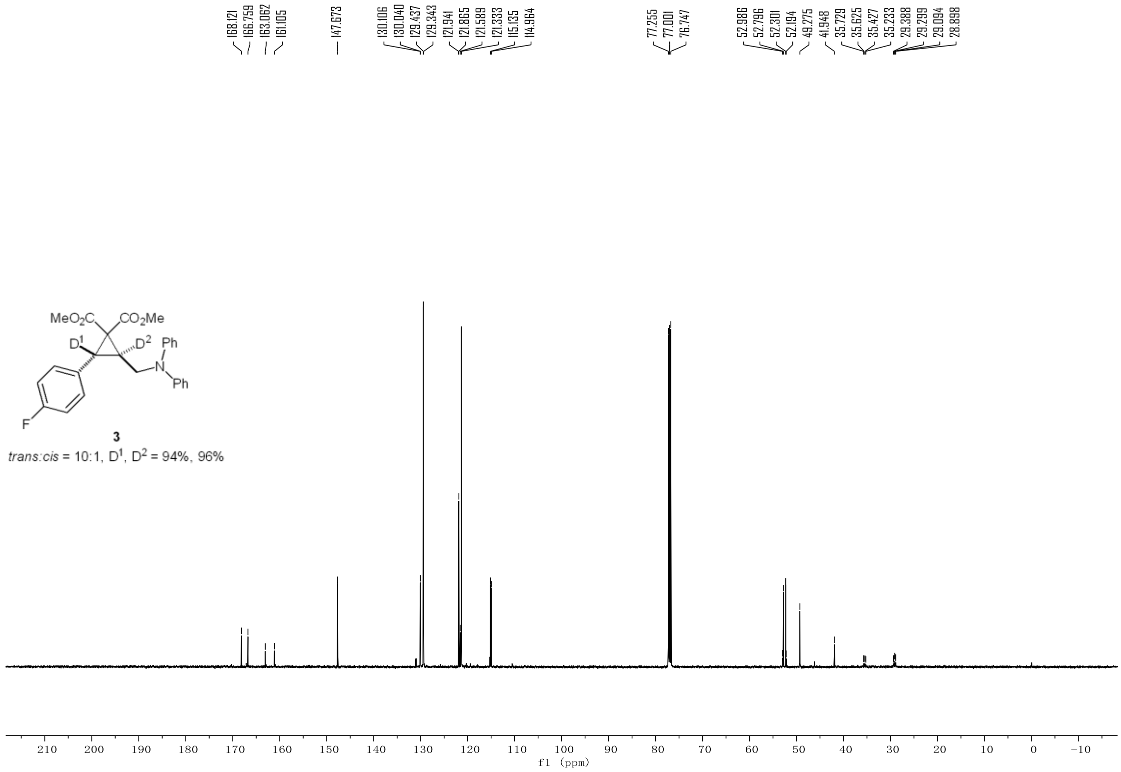

MeO2C CO2Me
D1 D2 Ph N Ph
F
3
trans:cis = 10:1, D1, D2 = 94%, 96%
f1 (ppm)

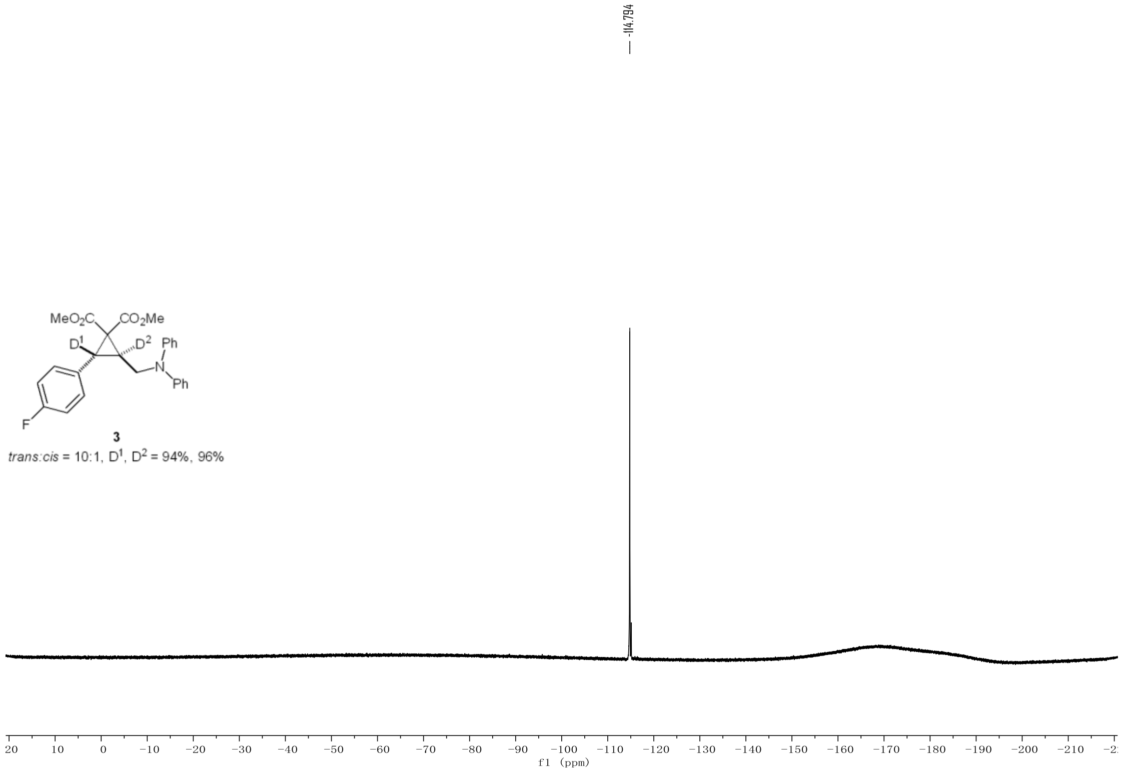

-114.794
MeO2C
CO2Me
D1
D2
Ph
N
Ph
F
3
trans:cis = 10:1, D1, D2 = 94%, 96%
f1 (ppm)

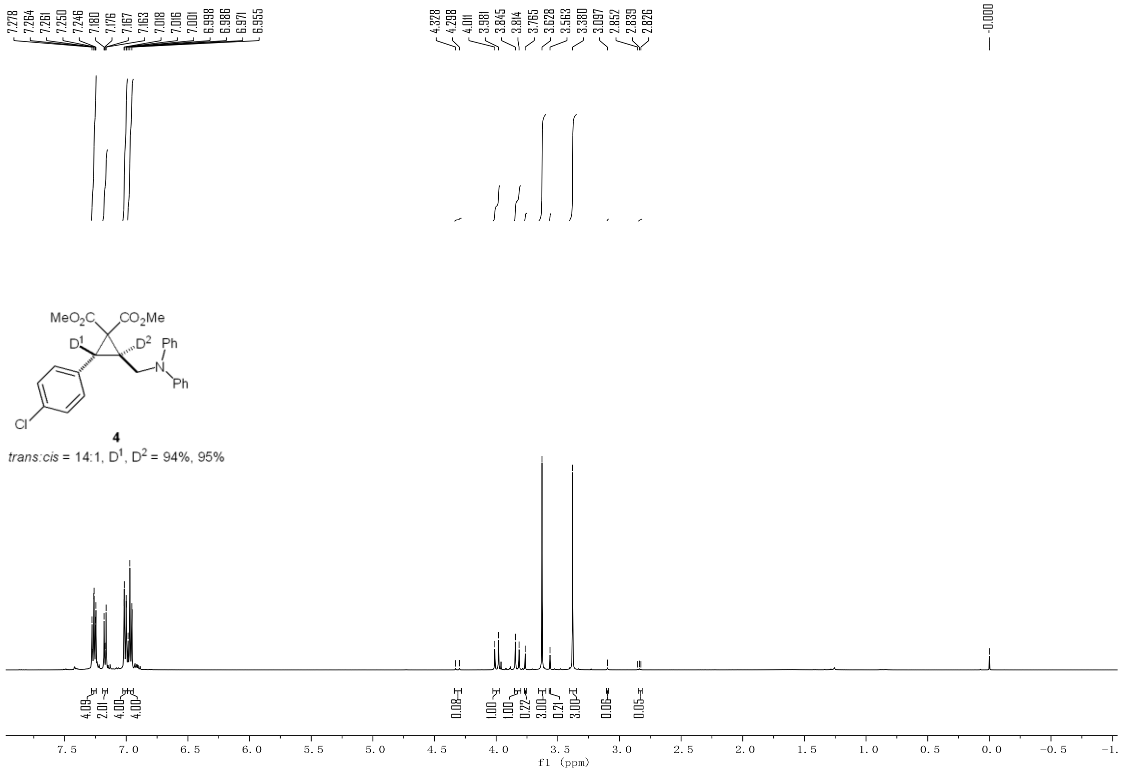

MeO2C
CO2Me
D1
D2
Ph
N
Ph
Cl
4
trans:cis = 14:1, D1, D2 = 94%, 95%
f1 (ppm)

4
trans:cis = 14:1, D^1, D^2 = 94%, 95%

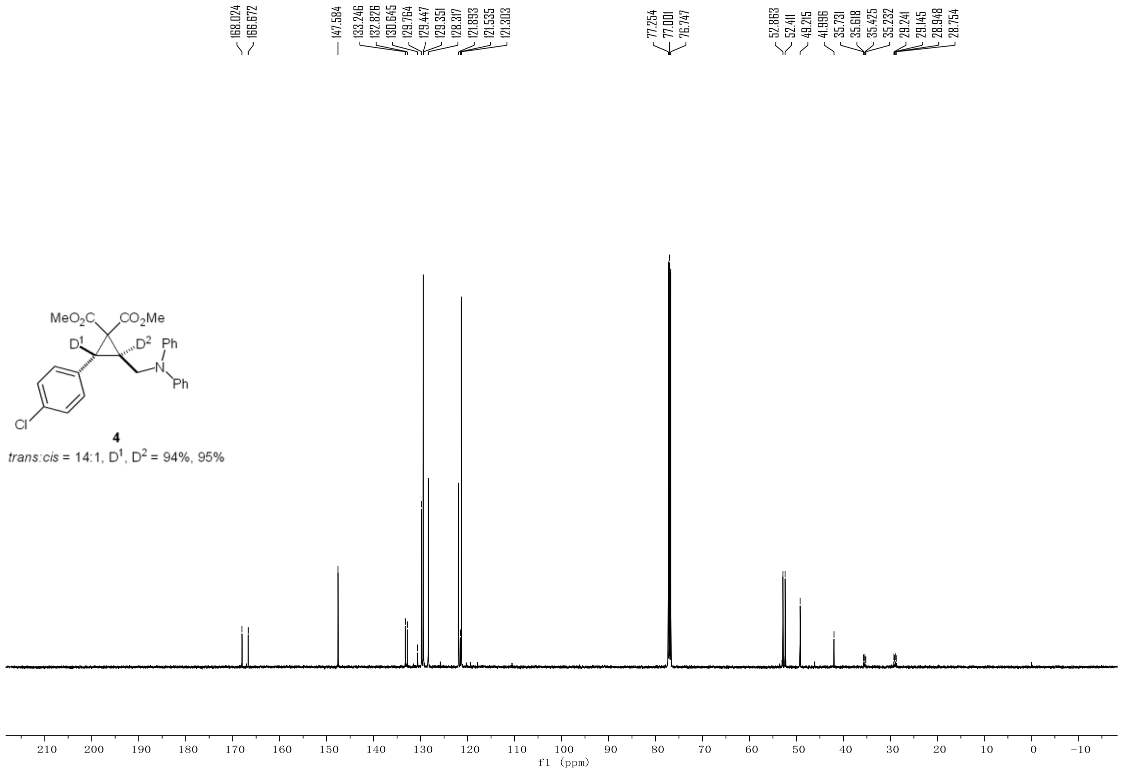

5
trans:cis = 13:1, D^1, D^2 = 97%, 93%

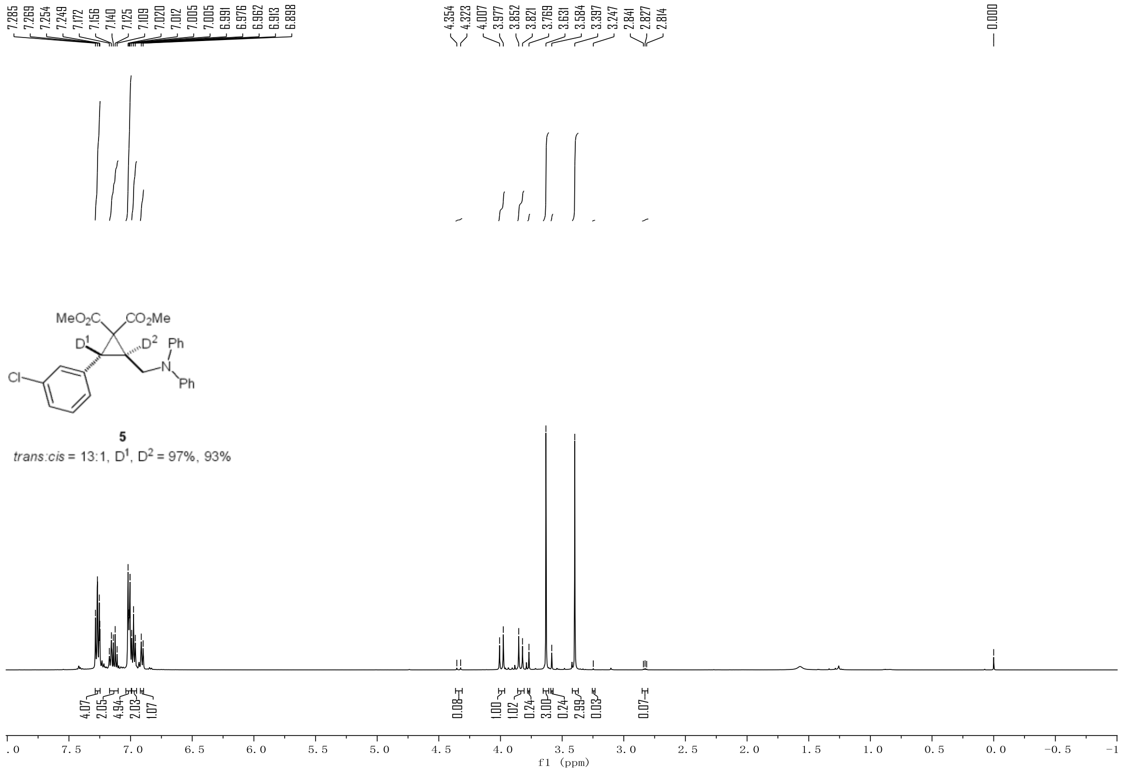

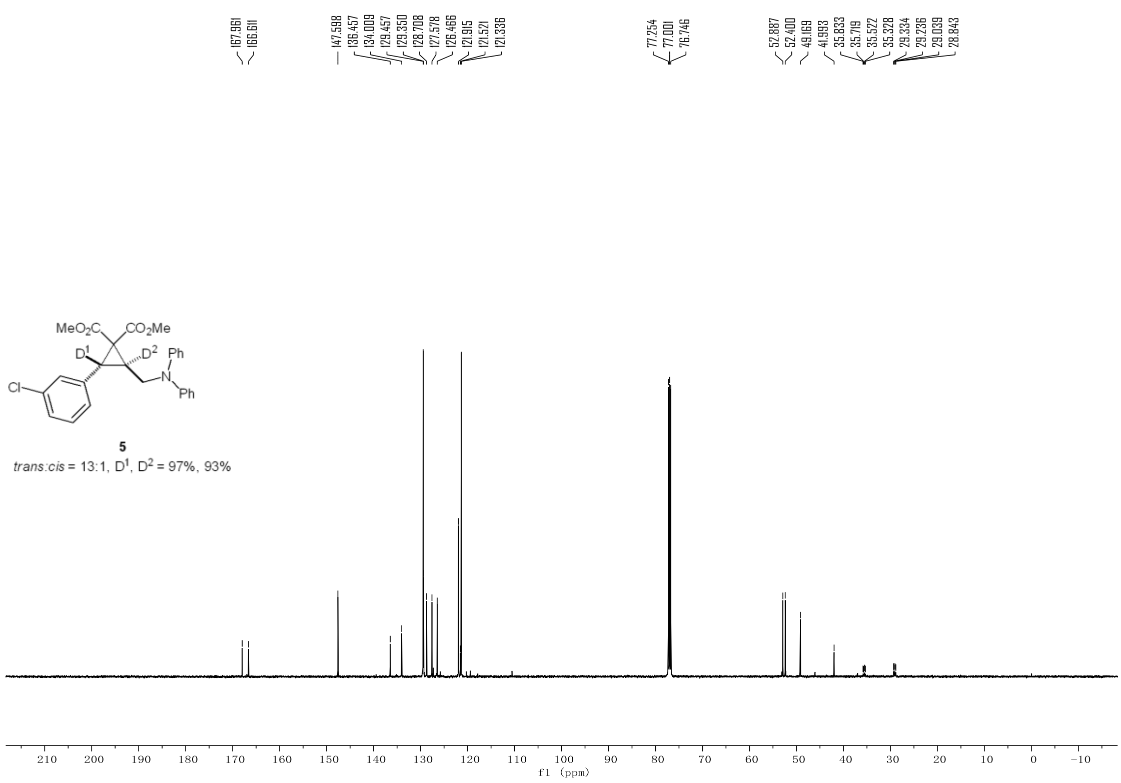

5

trans:cis = 13:1, D^1, D^2 = 97%, 93%

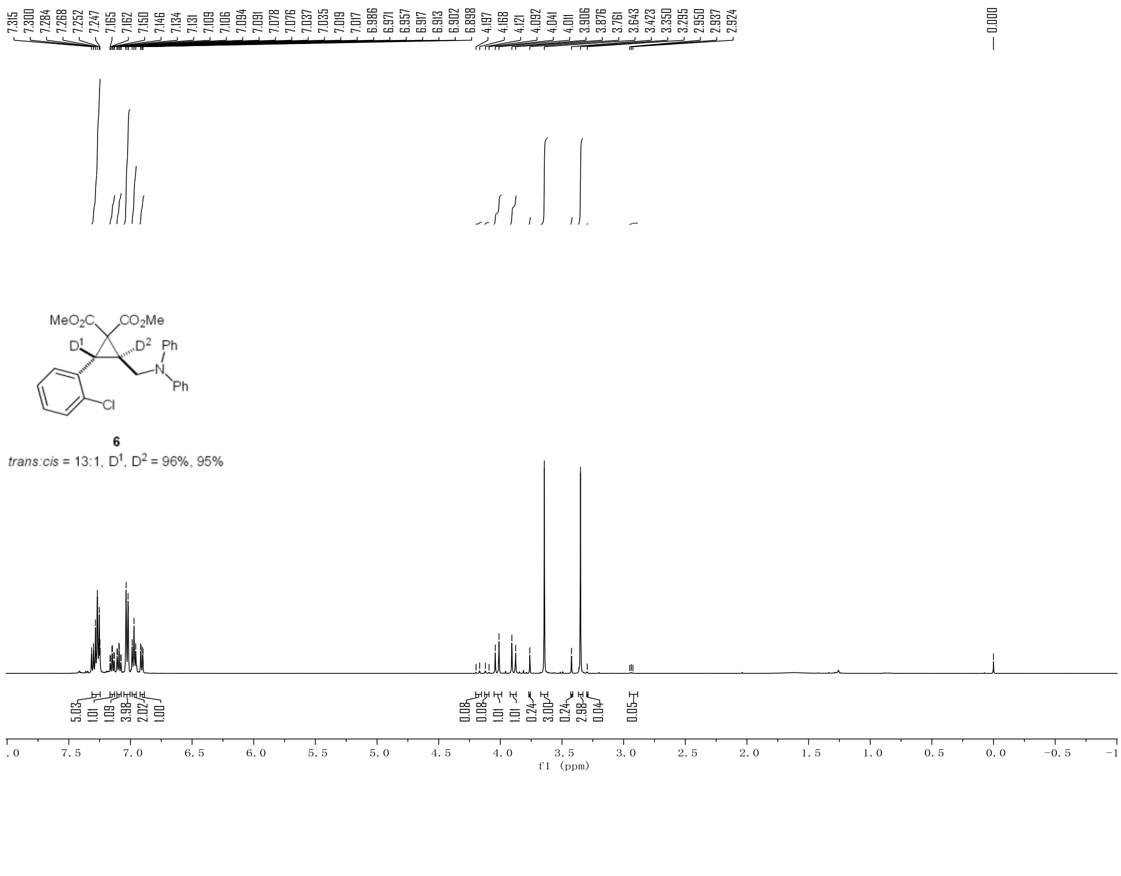

6

trans:cis = 13:1, D^1, D^2 = 96%, 95%

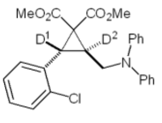

6

trans:cis = 13:1, D[1], D[2] = 96%, 95%

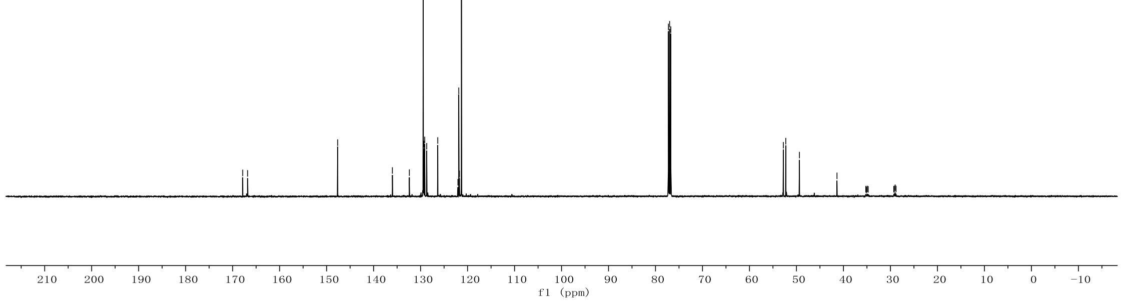

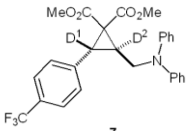

7

trans:cis = 13:1, D[1], D[2] = 95%, 95%

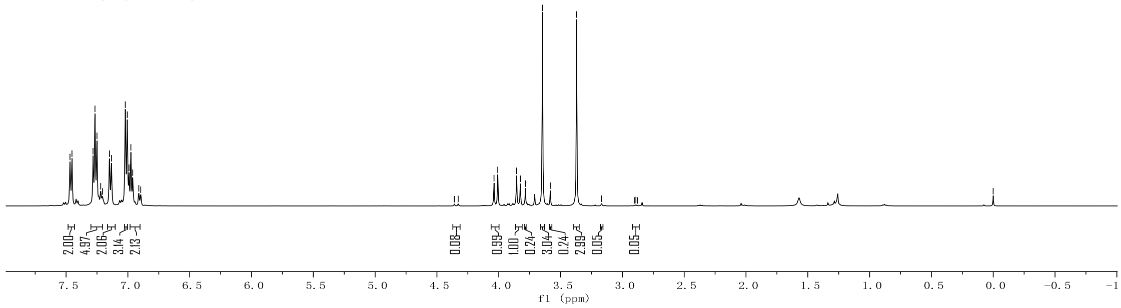

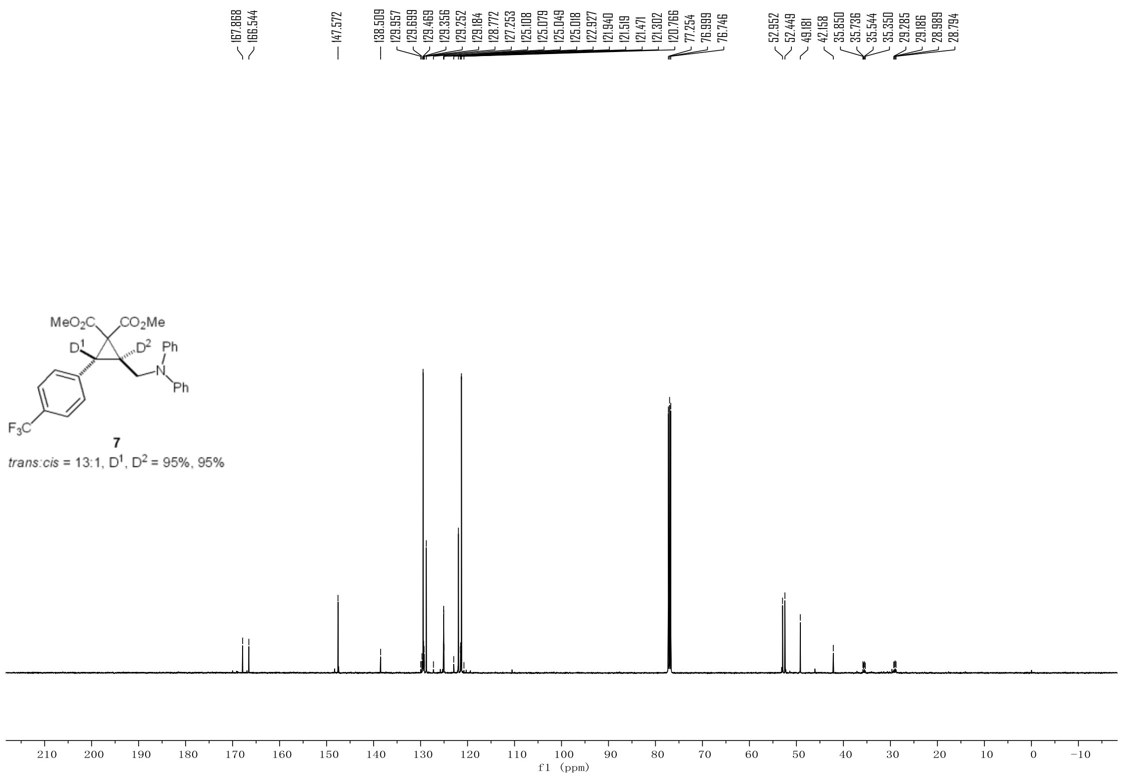
167.868
166.544
147.572
138.509
129.957
129.699
129.469
129.356
129.252
129.184
128.772
127.253
125.108
125.079
125.049
125.018
122.927
121.940
121.519
121.471
121.302
120.766
77.254
76.999
76.746
52.952
52.449
49.181
42.158
35.850
35.736
35.544
35.350
29.285
29.186
28.989
28.794
MeO2C
CO2Me
D1
D2
Ph
N
Ph
F3C
7
trans:cis = 13:1, D1, D2 = 95%, 95%
f1 (ppm)

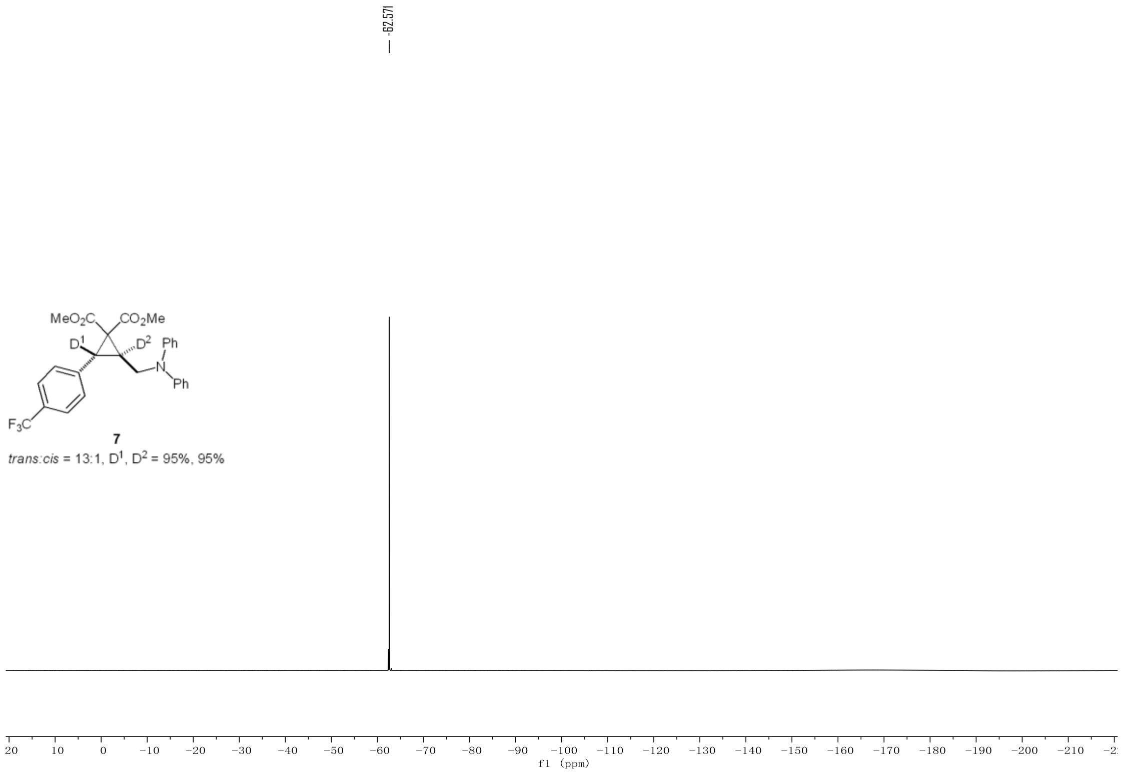
-62.571
MeO2C
CO2Me
D1
D2
Ph
N
Ph
F3C
7
trans:cis = 13:1, D1, D2 = 95%, 95%
f1 (ppm)

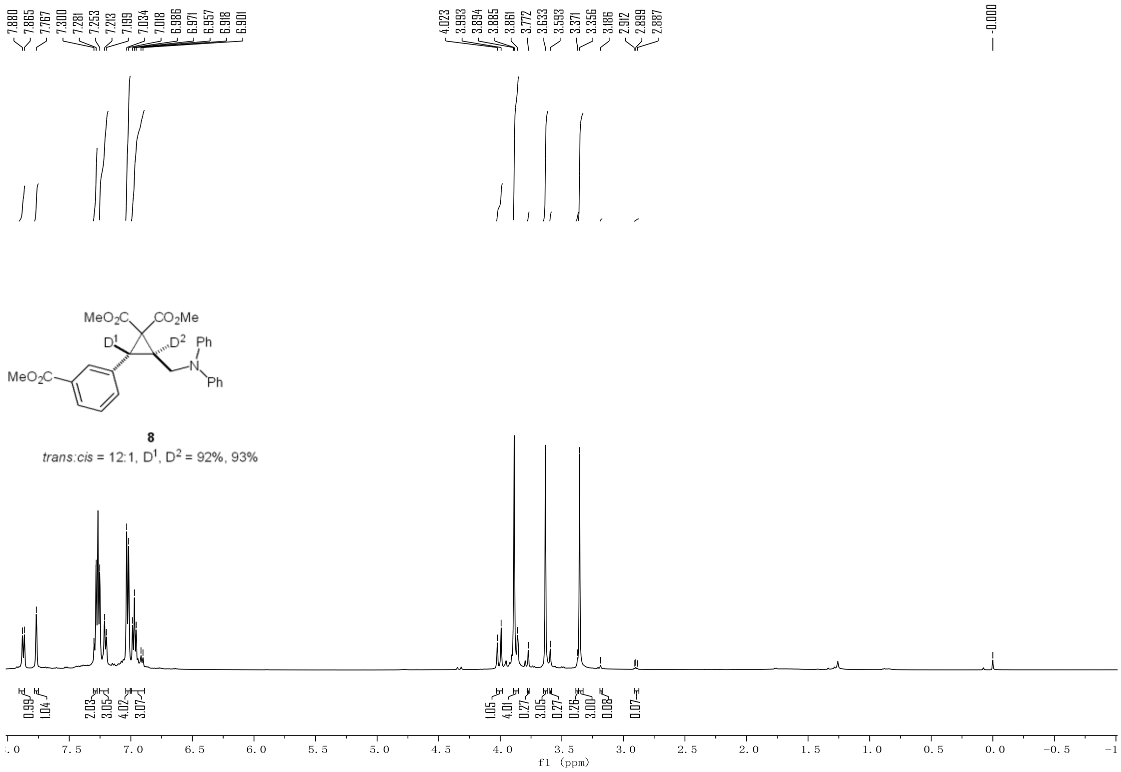
7.880
7.865
7.767
7.300
7.281
7.253
7.213
7.199
7.034
7.018
6.986
6.971
6.957
6.918
6.901
4.023
3.993
3.894
3.885
3.861
3.772
3.633
3.593
3.371
3.356
3.186
2.912
2.899
2.887
-0.000
MeO2C
CO2Me
D1
D2
Ph
N
Ph
MeO2C
8
trans:cis = 12:1, D1, D2 = 92%, 93%
0.99
1.04
2.03
3.05
4.02
3.07
1.05
4.01
0.27
3.05
0.27
0.26
3.00
0.08
0.07
f1 (ppm)

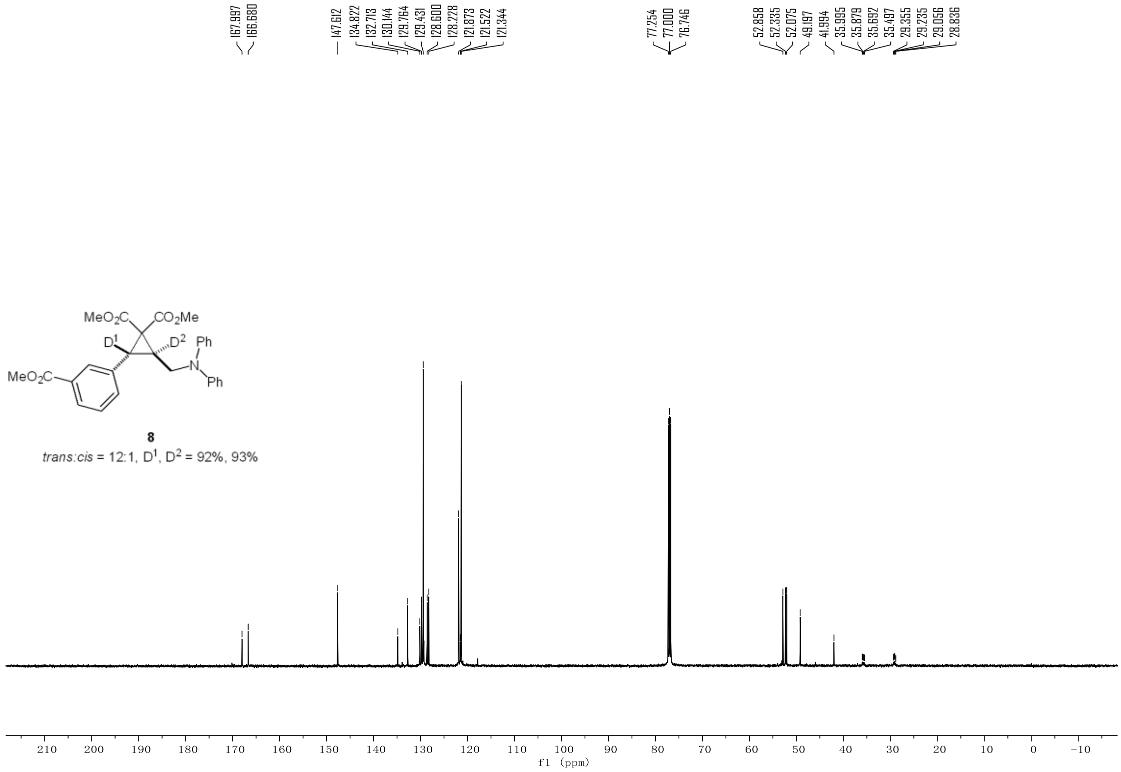
167.997
166.680
147.612
134.822
132.713
130.144
129.764
129.431
128.600
128.228
121.873
121.522
121.344
77.254
77.000
76.746
52.858
52.335
52.075
49.197
41.994
35.995
35.879
35.692
35.497
29.355
29.235
29.056
28.836
MeO2C
CO2Me
D1
D2
Ph
N
Ph
MeO2C
8
trans:cis = 12:1, D1, D2 = 92%, 93%
f1 (ppm)

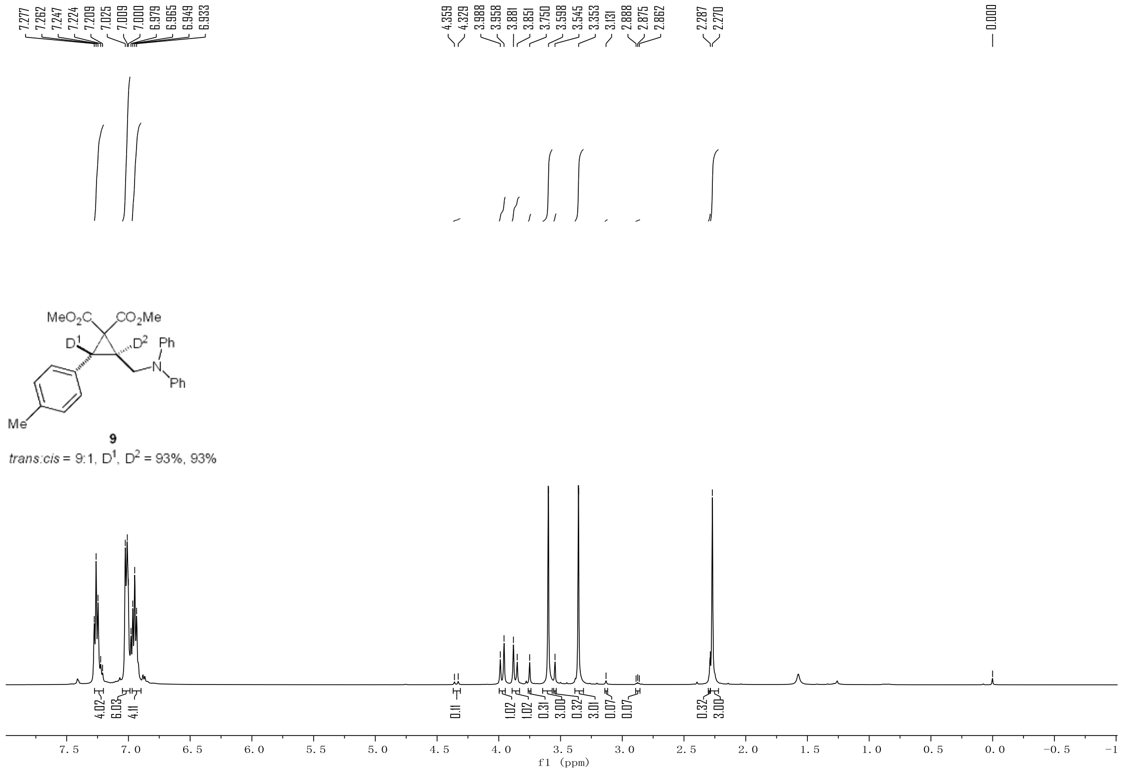
7.277
7.262
7.247
7.224
7.209
7.025
7.009
7.000
6.979
6.965
6.949
6.933
4.359
4.329
3.988
3.958
3.881
3.851
3.750
3.598
3.545
3.353
3.131
2.888
2.875
2.862
2.287
2.270
0.000
MeO2C
CO2Me
D1
D2
Ph
N
Ph
Me
9
trans:cis = 9:1, D1, D2 = 93%, 93%
4.02
6.03
4.11
0.11
1.02
1.02
0.31
3.00
0.32
3.01
0.07
0.07
0.32
3.00
f1 (ppm)

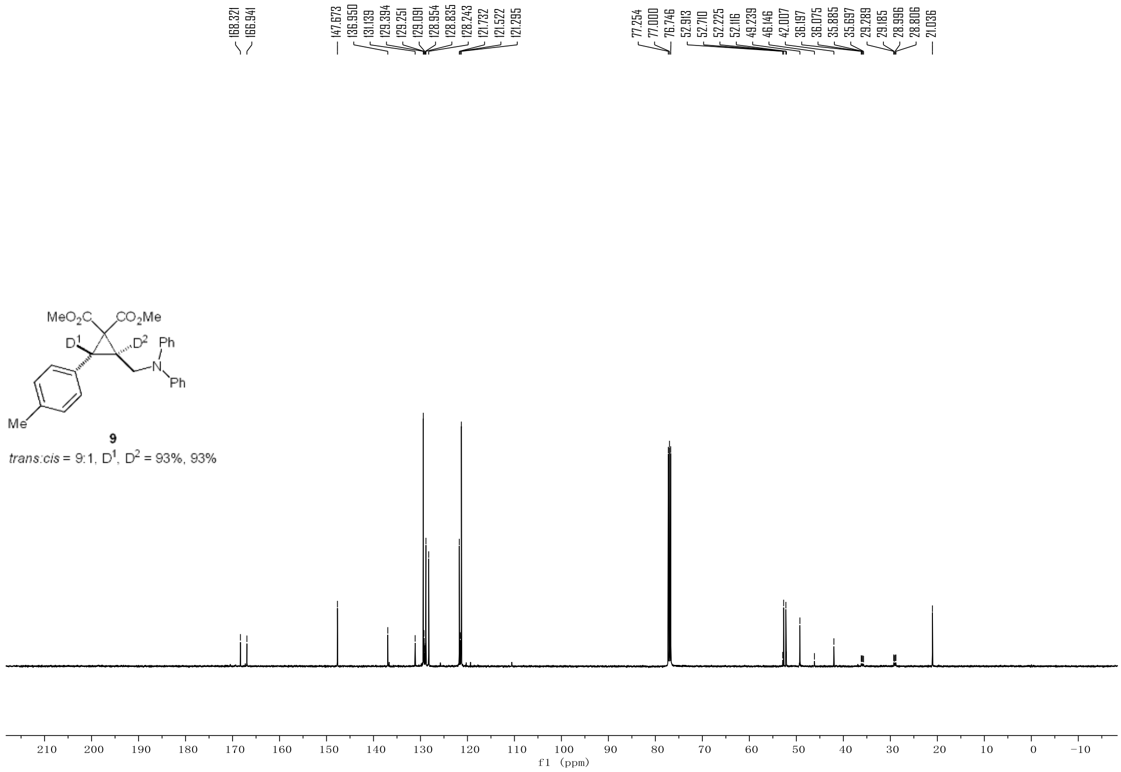
168.321
166.941
147.673
136.950
131.139
129.394
129.251
129.091
128.954
128.835
128.243
121.732
121.522
121.235
77.254
77.000
76.746
52.913
52.710
52.225
52.116
49.239
46.146
42.007
36.197
36.075
35.885
35.697
29.289
29.185
28.996
28.806
21.036
MeO2C
CO2Me
D1
D2
Ph
N
Ph
Me
9
trans:cis = 9:1, D1, D2 = 93%, 93%
f1 (ppm)

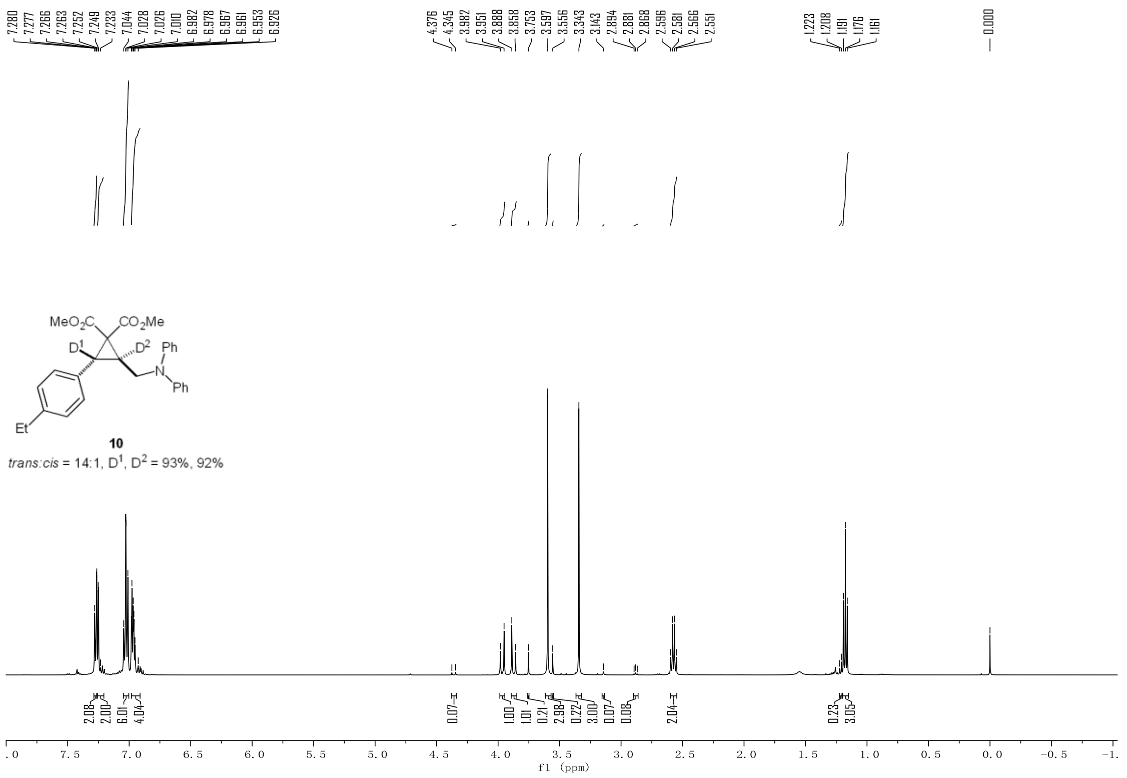
MeO2C CO2Me
D1 D2 Ph
N
Ph
Et
10
trans:cis = 14:1, D1, D2 = 93%, 92%
f1 (ppm)

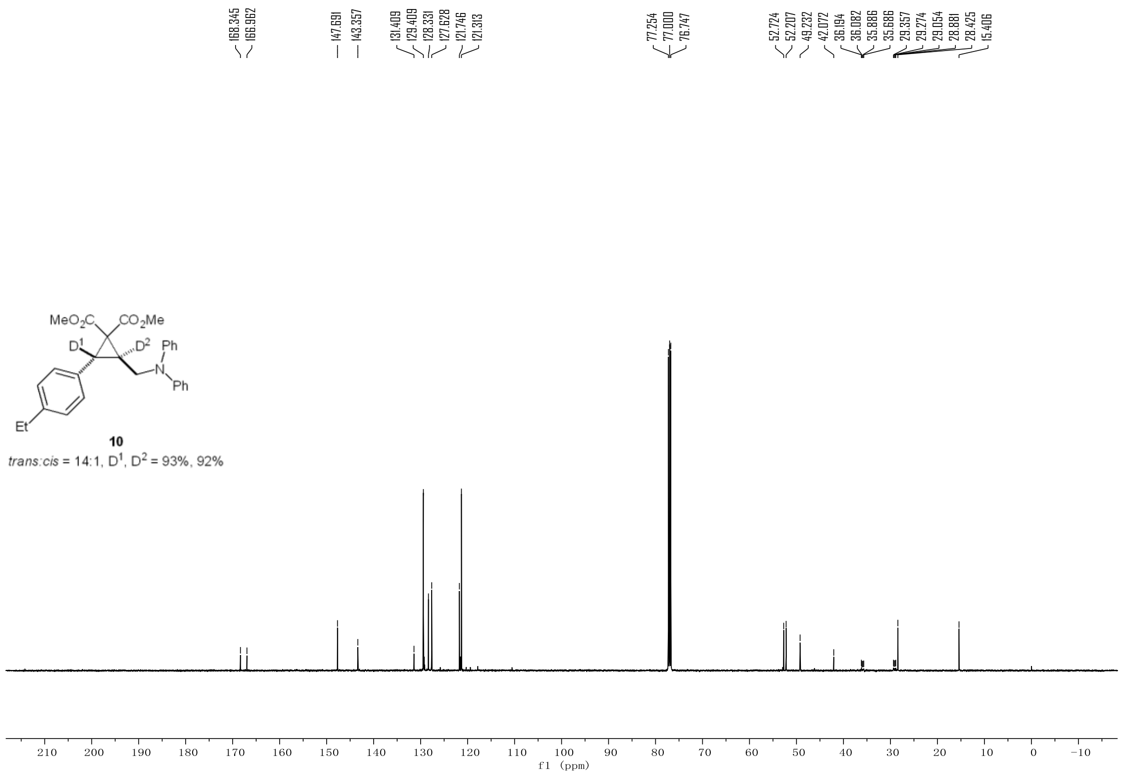
MeO2C CO2Me
D1 D2 Ph
N
Ph
Et
10
trans:cis = 14:1, D1, D2 = 93%, 92%
f1 (ppm)

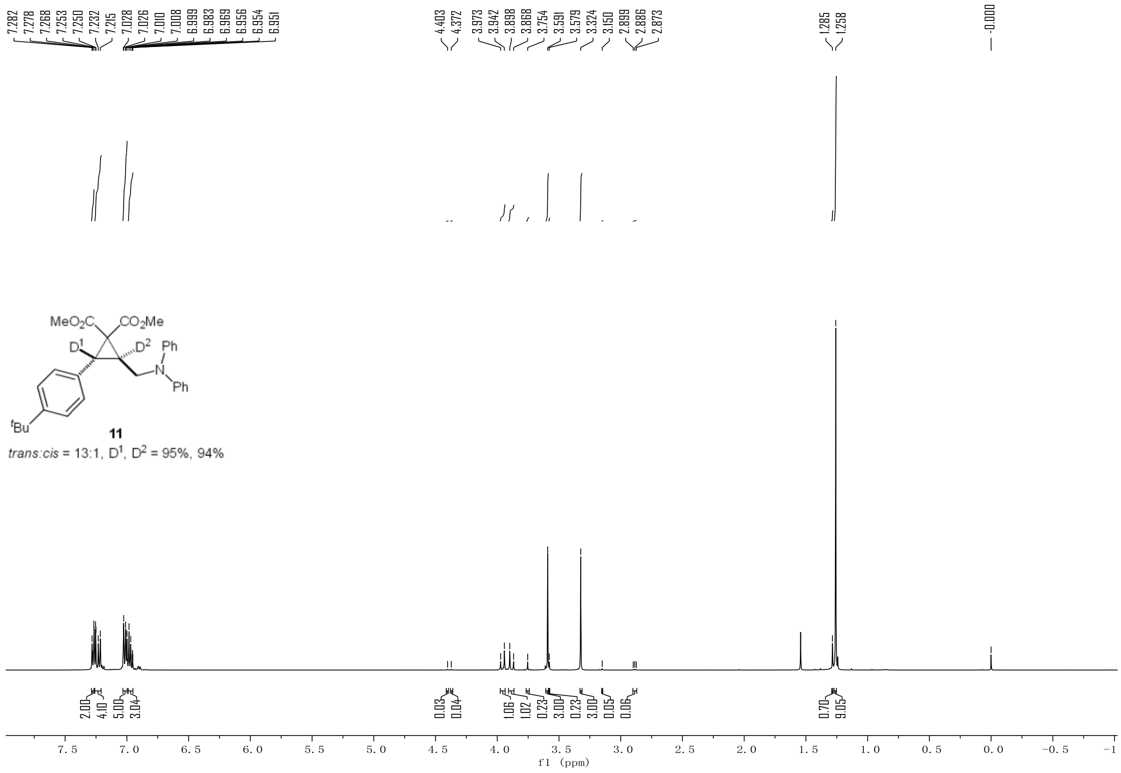
7.282 7.278 7.268 7.253 7.250 7.232 7.215 7.028 7.026 7.010 7.008 6.999 6.983 6.969 6.956 6.954 6.951
4.403 4.372 3.973 3.942 3.898 3.868 3.754 3.591 3.579 3.324 3.150 2.899 2.886 2.873
1.285 1.258
-0.000
MeO2C CO2Me D1 D2 Ph N Ph tBu
11
trans:cis = 13:1, D1, D2 = 95%, 94%
2.00 4.10 5.00 3.04 0.03 0.04 1.06 1.02 0.23 3.00 0.23 3.00 0.05 0.06 0.70 9.05
7.5 7.0 6.5 6.0 5.5 5.0 4.5 4.0 3.5 3.0 2.5 2.0 1.5 1.0 0.5 0.0 -0.5 -1
f1 (ppm)

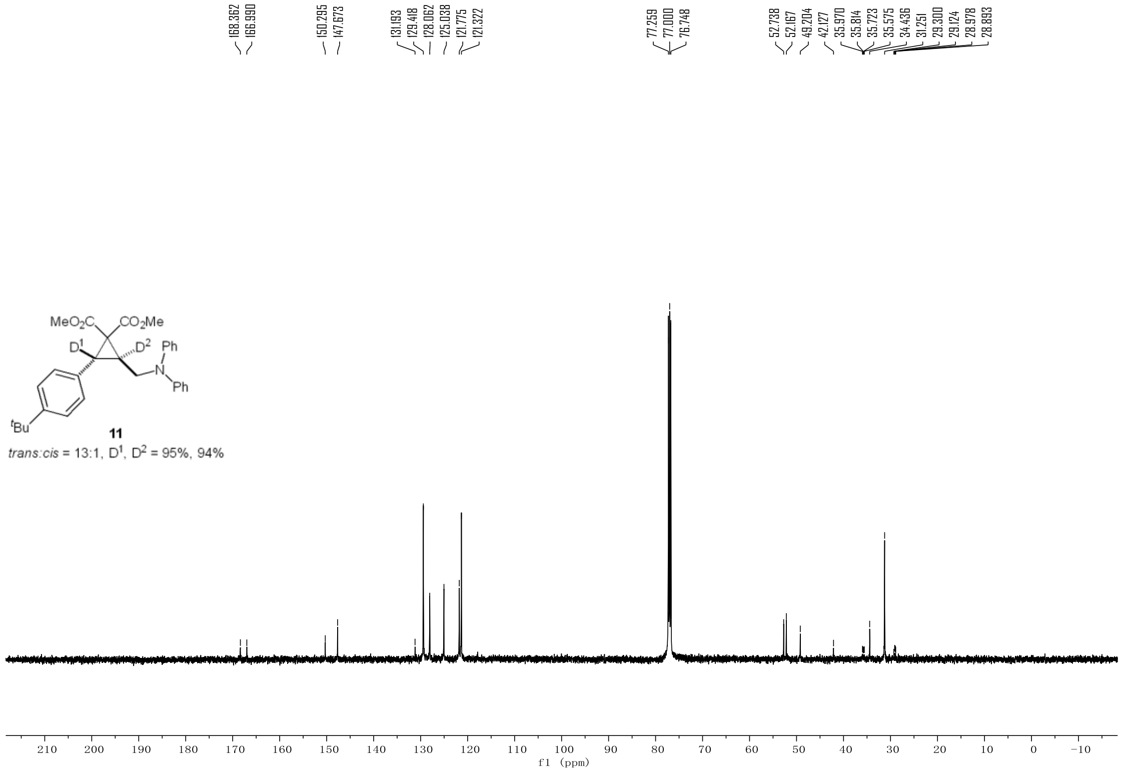
168.362 166.990 150.295 147.673 131.193 129.418 128.062 125.038 121.775 121.322
77.259 77.000 76.748
52.738 52.167 49.204 42.127 35.970 35.814 35.723 35.575 34.436 31.251 29.300 29.124 28.978 28.893
MeO2C CO2Me D1 D2 Ph N Ph tBu
11
trans:cis = 13:1, D1, D2 = 95%, 94%
210 200 190 180 170 160 150 140 130 120 110 100 90 80 70 60 50 40 30 20 10 0 -10
f1 (ppm)

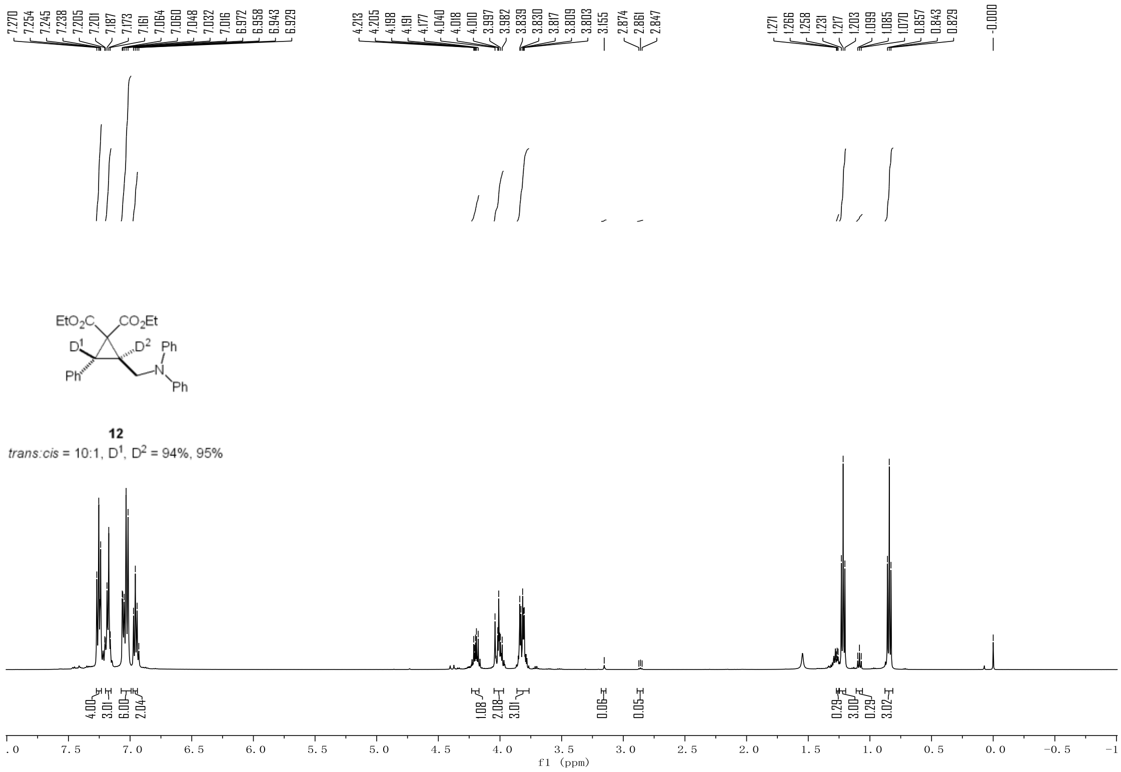
7.270 7.254 7.245 7.238 7.205 7.201 7.187 7.173 7.161 7.064 7.060 7.048 7.032 7.016 6.972 6.958 6.943 6.929
4.213 4.205 4.198 4.191 4.177 4.040 4.018 4.010 3.997 3.982 3.839 3.830 3.817 3.809 3.803 3.155 2.874 2.861 2.847
1.271 1.266 1.258 1.231 1.217 1.203 1.099 1.085 1.070 0.857 0.843 0.829 -0.000
EtO2C CO2Et D1 D2 Ph N Ph Ph
12
trans:cis = 10:1, D1, D2 = 94%, 95%
4.00 3.01 6.00 2.04 1.08 2.08 3.01 0.06 0.05 0.29 3.00 0.29 3.02
f1 (ppm)

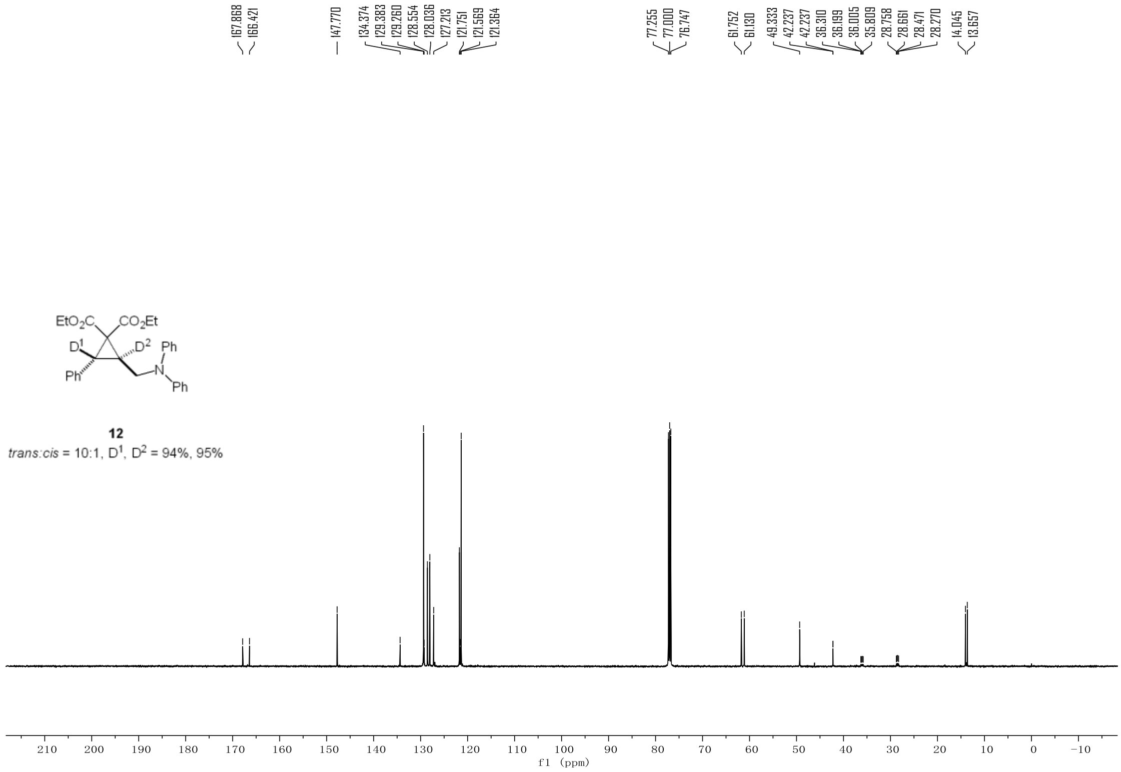
167.868 166.421 147.770 134.374 129.383 129.260 128.554 128.036 127.213 121.751 121.569 121.364 77.255 77.000 76.747 61.752 61.130 49.333 42.237 42.237 36.310 36.199 36.005 35.809 28.758 28.661 28.471 28.270 14.045 13.657
EtO2C CO2Et D1 D2 Ph N Ph Ph
12
trans:cis = 10:1, D1, D2 = 94%, 95%
f1 (ppm)

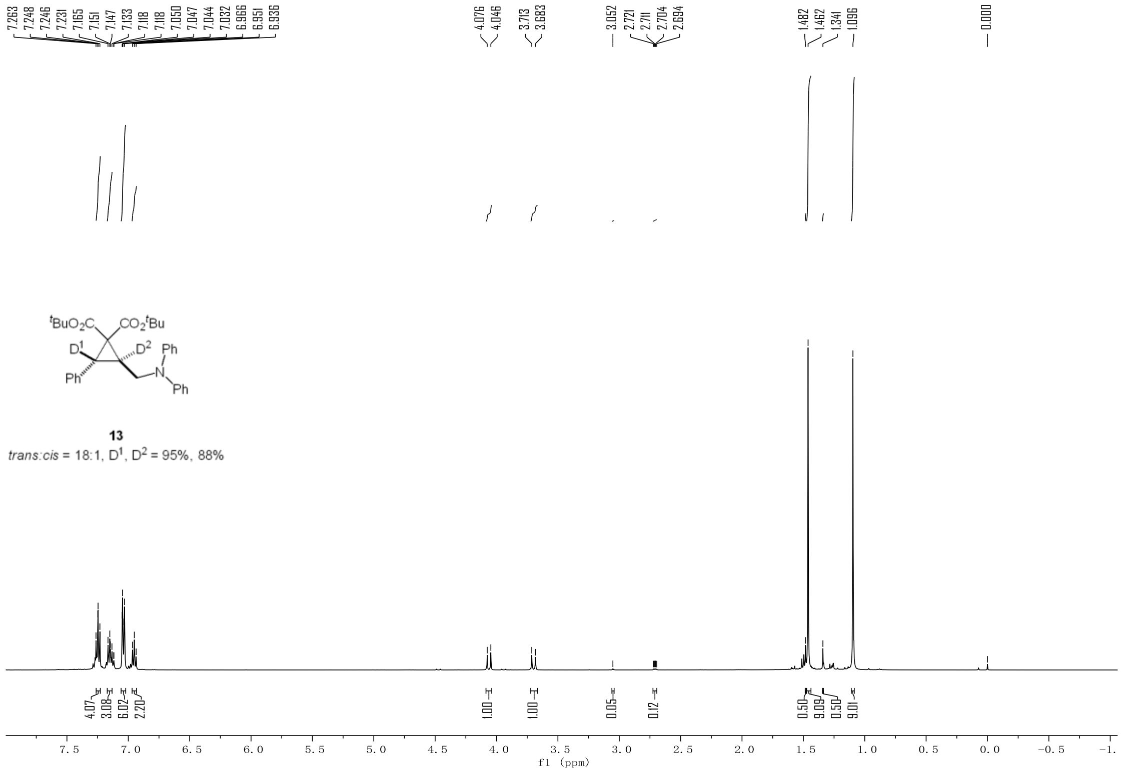

13
trans:cis = 18:1, D1, D2 = 95%, 88%
f1 (ppm)

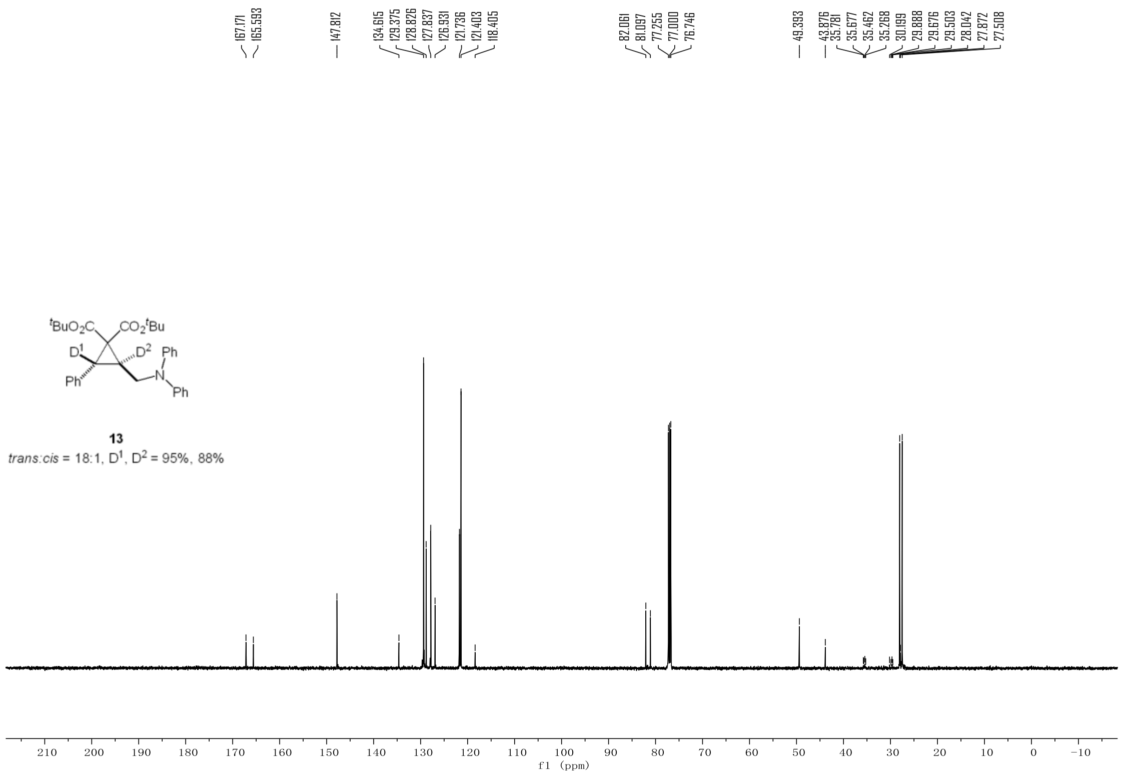

13
trans:cis = 18:1, D1, D2 = 95%, 88%
f1 (ppm)

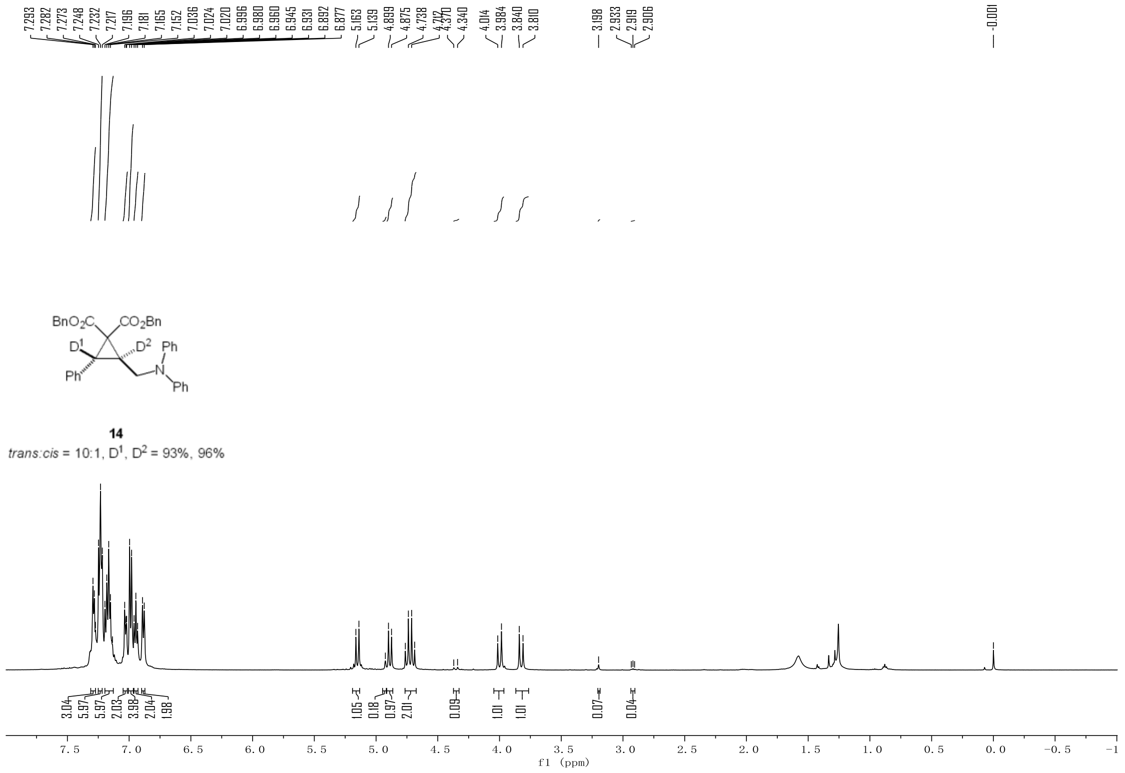

7.293 7.282 7.273 7.248 7.232 7.217 7.196 7.181 7.165 7.152 7.036 7.024 7.020 6.996 6.980 6.960 6.945 6.931 6.892 6.877 5.163 5.139 4.899 4.875 4.738 4.370 4.340 4.014 3.984 3.840 3.810 3.198 2.933 2.919 2.906 -0.001
BnO2C CO2Bn D1 D2 Ph N Ph Ph
14
trans:cis = 10:1, D1, D2 = 93%, 96%
3.04 5.97 5.97 2.03 3.98 2.04 1.98 1.05 0.18 0.97 2.01 0.09 1.01 1.01 0.07 0.04
7.5 7.0 6.5 6.0 5.5 5.0 4.5 4.0 3.5 3.0 2.5 2.0 1.5 1.0 0.5 0.0 -0.5 -1
f1 (ppm)

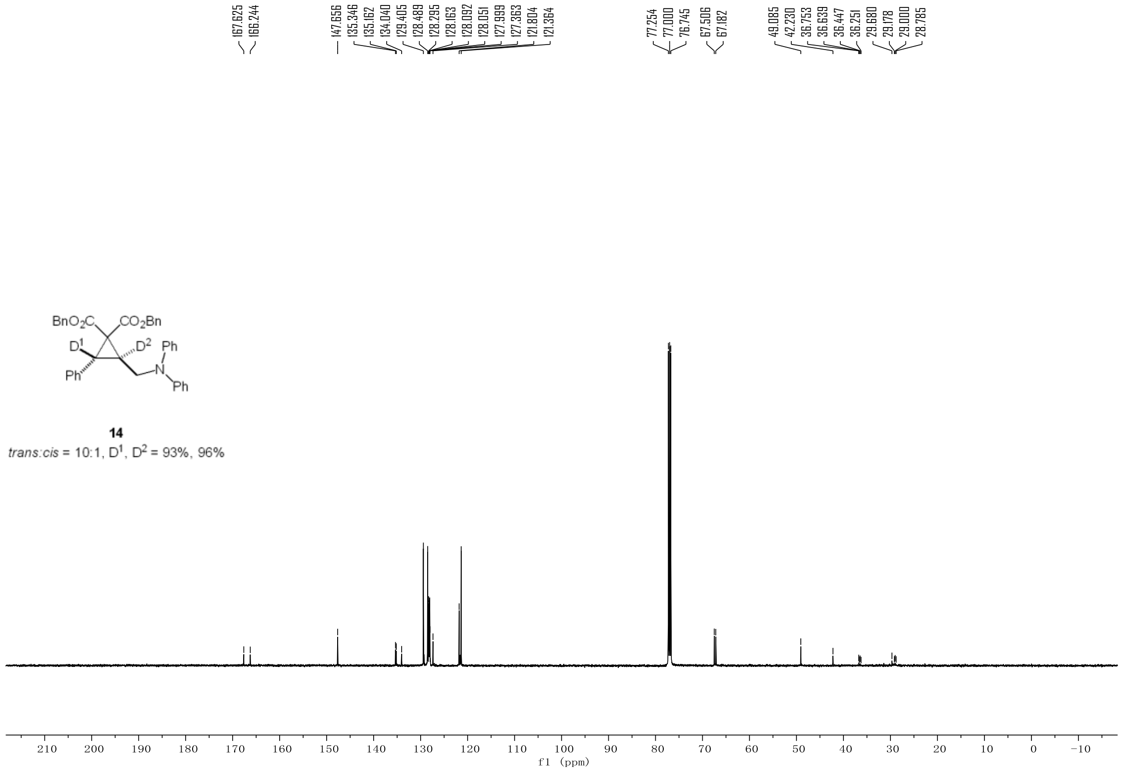

167.625 166.244 147.656 135.346 135.162 134.040 129.405 128.489 128.295 128.163 128.092 128.051 127.999 127.363 121.804 121.364 77.254 77.000 76.745 67.506 67.182 49.085 42.230 36.753 36.639 36.447 36.251 29.680 29.178 29.000 28.785
BnO2C CO2Bn D1 D2 Ph N Ph Ph
14
trans:cis = 10:1, D1, D2 = 93%, 96%
210 200 190 180 170 160 150 140 130 120 110 100 90 80 70 60 50 40 30 20 10 0 -10
f1 (ppm)

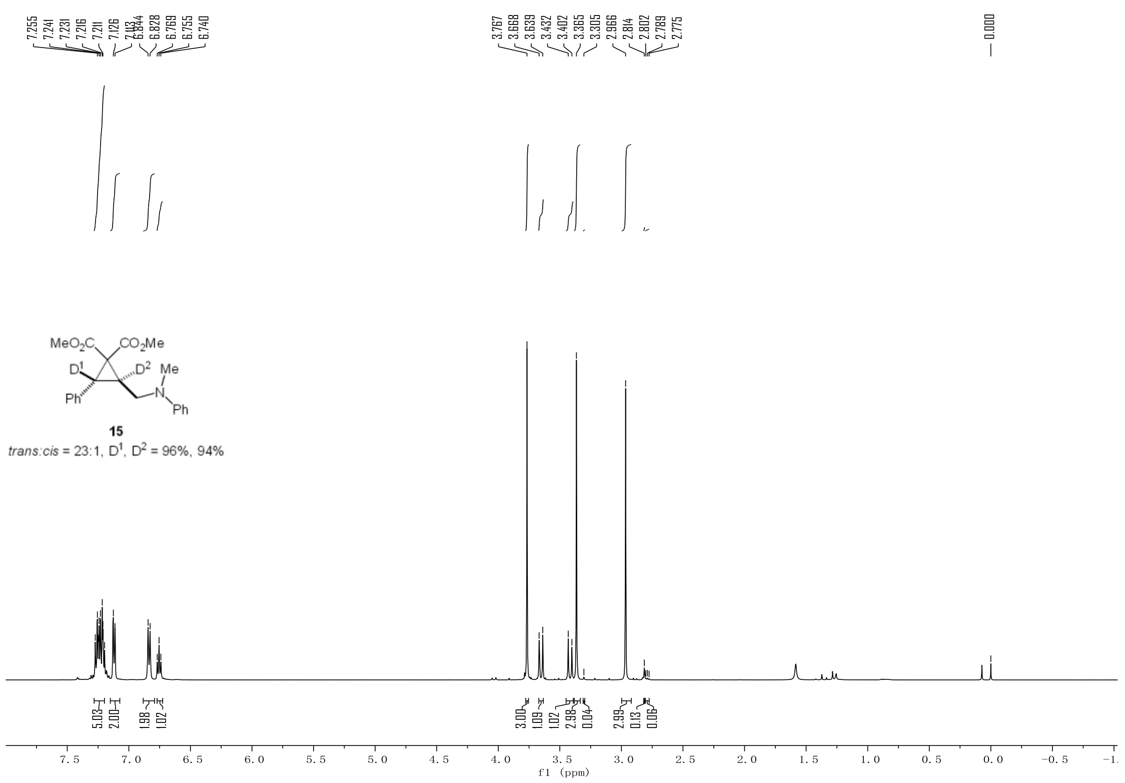

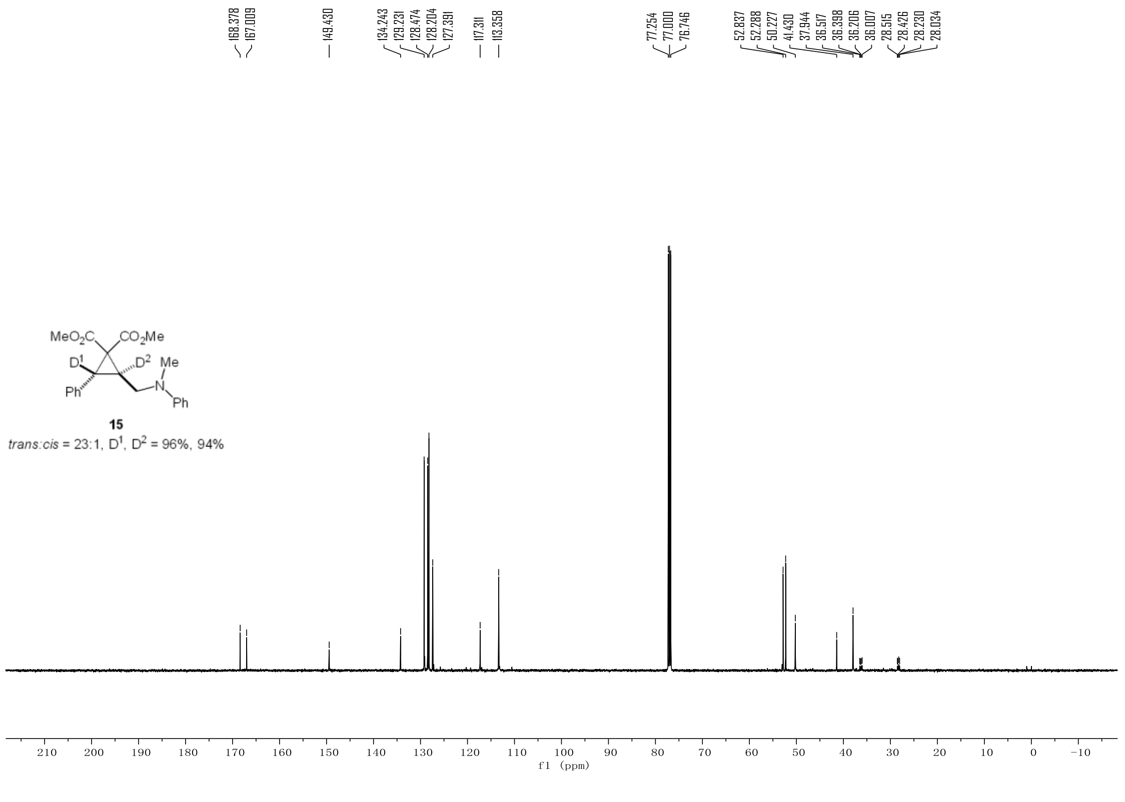

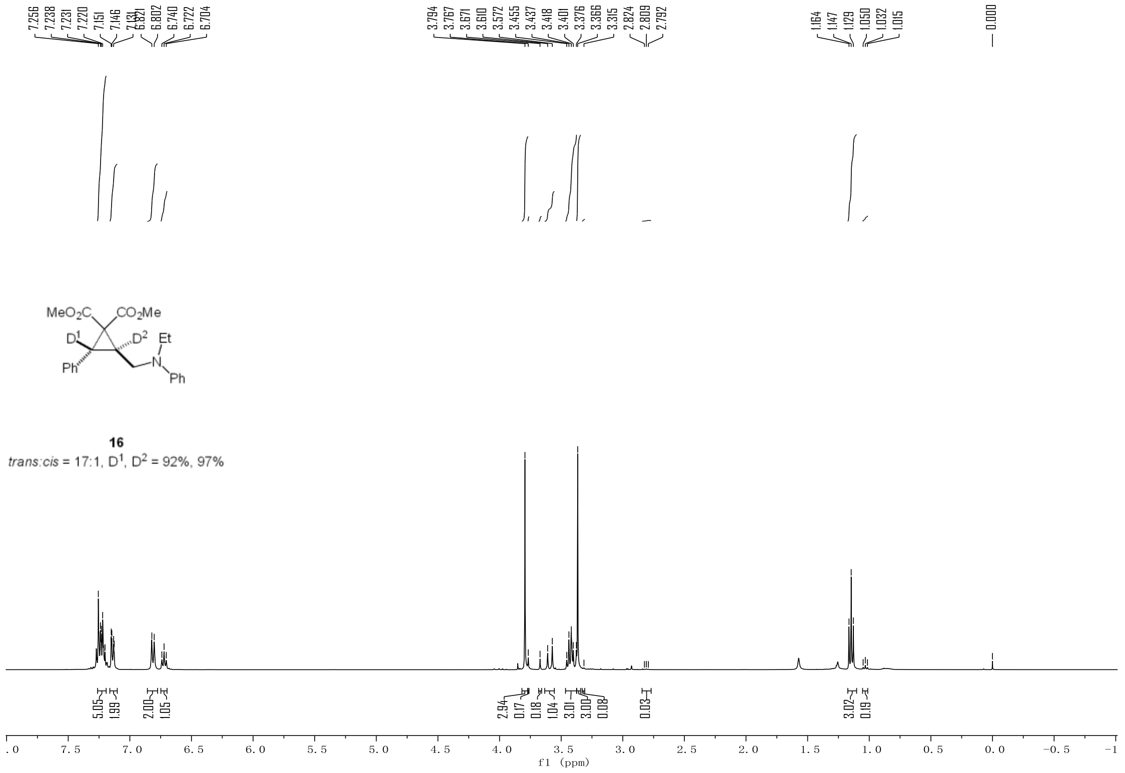
7.256
7.238
7.231
7.220
7.151
7.146
6.802
6.740
6.722
6.704
3.794
3.767
3.671
3.610
3.572
3.455
3.437
3.418
3.401
3.376
3.366
3.315
2.824
2.809
2.792
1.164
1.147
1.129
1.050
1.032
1.015
0.000
MeO2C
CO2Me
D1
D2
Et
N
Ph
Ph
16
trans:cis = 17:1, D1, D2 = 92%, 97%
f1 (ppm)

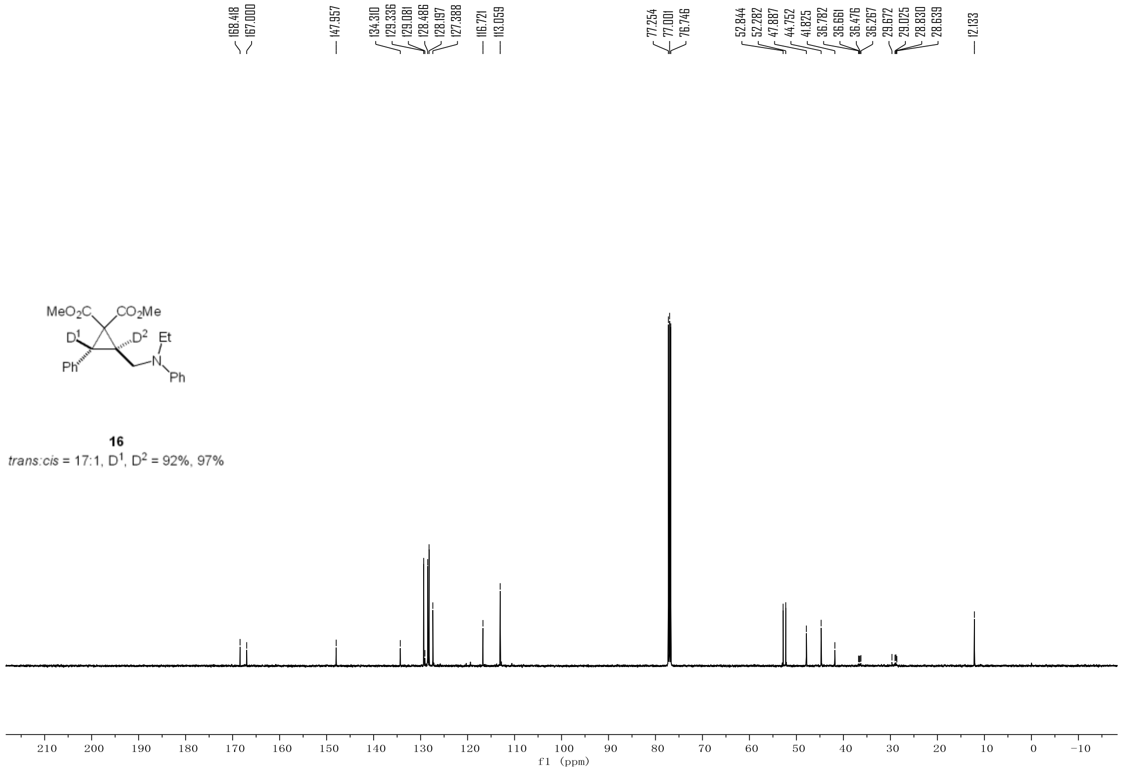
168.418
167.000
147.957
134.310
129.336
129.081
128.486
128.197
127.388
116.721
113.059
77.254
77.001
76.746
52.844
52.282
47.887
44.752
41.825
36.782
36.661
36.476
36.267
29.672
29.025
28.830
28.639
12.133
MeO2C
CO2Me
D1
D2
Et
N
Ph
Ph
16
trans:cis = 17:1, D1, D2 = 92%, 97%
f1 (ppm)

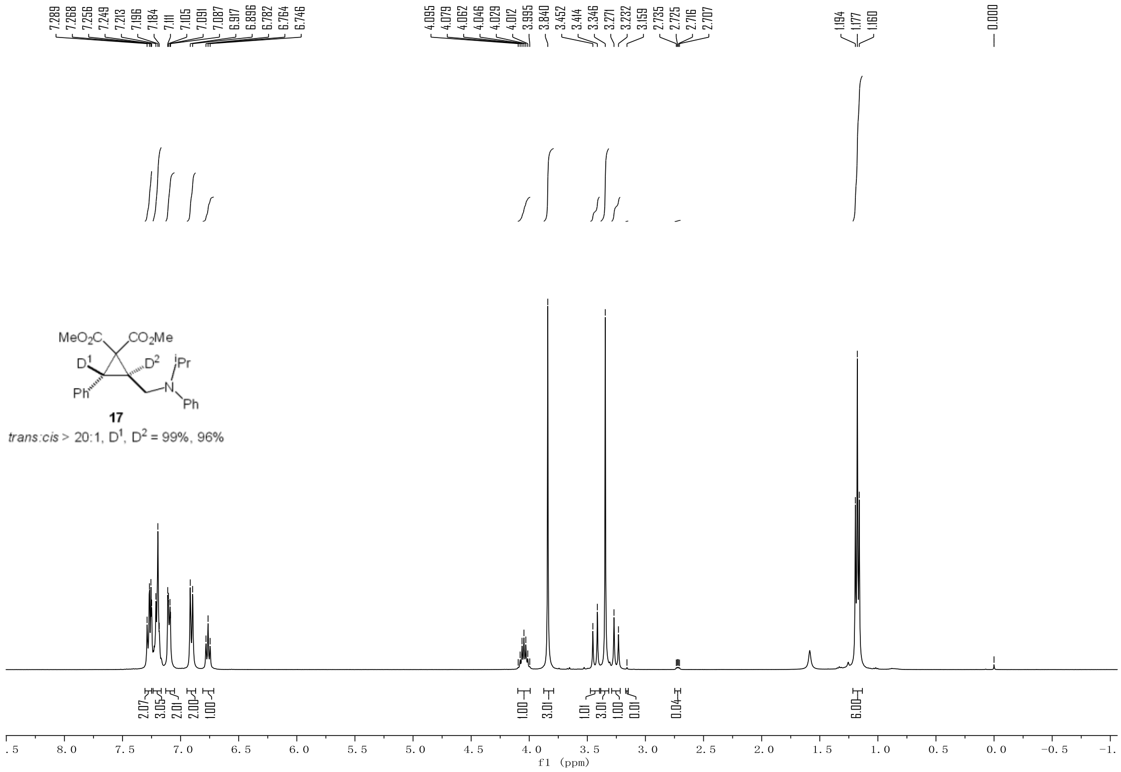
7.289
7.268
7.256
7.249
7.213
7.196
7.184
7.111
7.105
7.091
7.087
6.917
6.896
6.782
6.764
6.746
4.095
4.079
4.062
4.046
4.029
4.012
3.995
3.840
3.452
3.414
3.346
3.271
3.232
3.159
2.735
2.725
2.716
2.707
1.194
1.177
1.160
0.000
MeO2C
CO2Me
D1
D2
iPr
N
Ph
Ph
17
trans:cis > 20:1, D1, D2 = 99%, 96%
2.07
3.05
2.01
2.00
1.00
1.00
3.01
1.01
3.01
1.00
0.01
0.04
6.00
f1 (ppm)

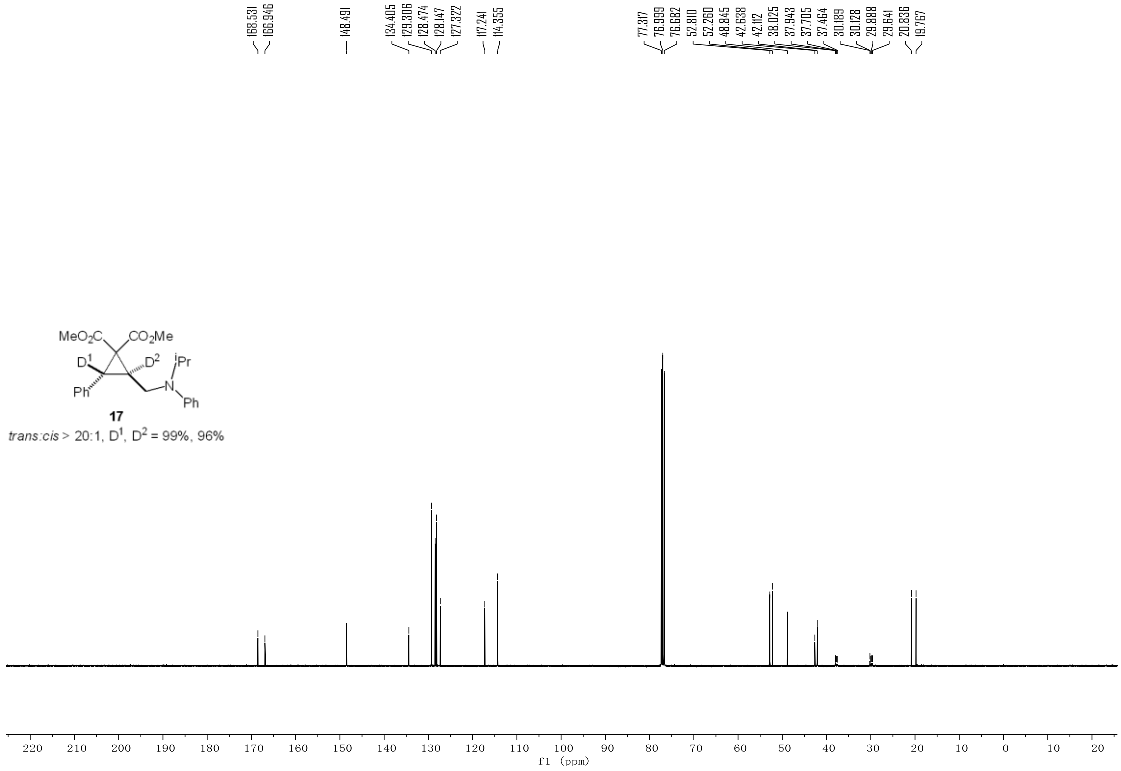
168.531
166.946
148.491
134.405
129.306
128.474
128.147
127.322
117.241
114.355
77.317
76.999
76.682
52.810
52.260
48.845
42.638
42.112
38.025
37.943
37.705
37.464
30.188
30.128
29.888
29.641
20.836
19.767
MeO2C
CO2Me
D1
D2
iPr
N
Ph
Ph
17
trans:cis > 20:1, D1, D2 = 99%, 96%
f1 (ppm)

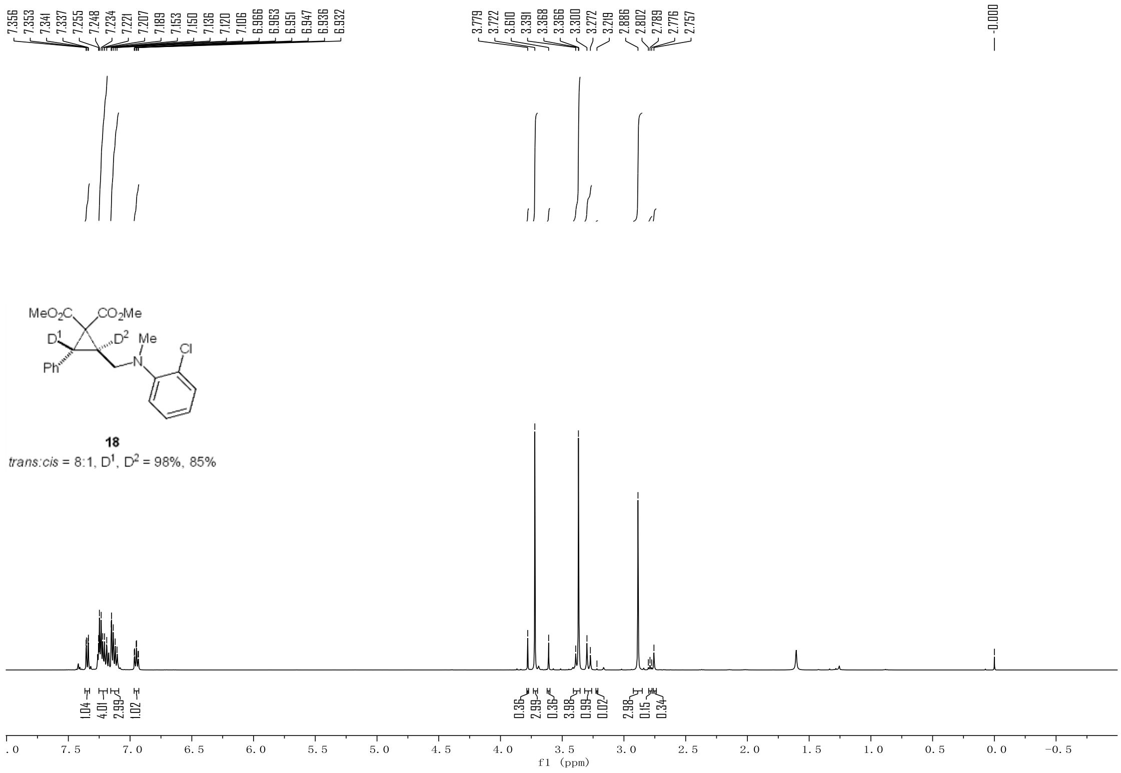
MeO2C
CO2Me
D1
D2
Me
Cl
Ph
N
18
trans:cis = 8:1, D1, D2 = 98%, 85%
f1 (ppm)

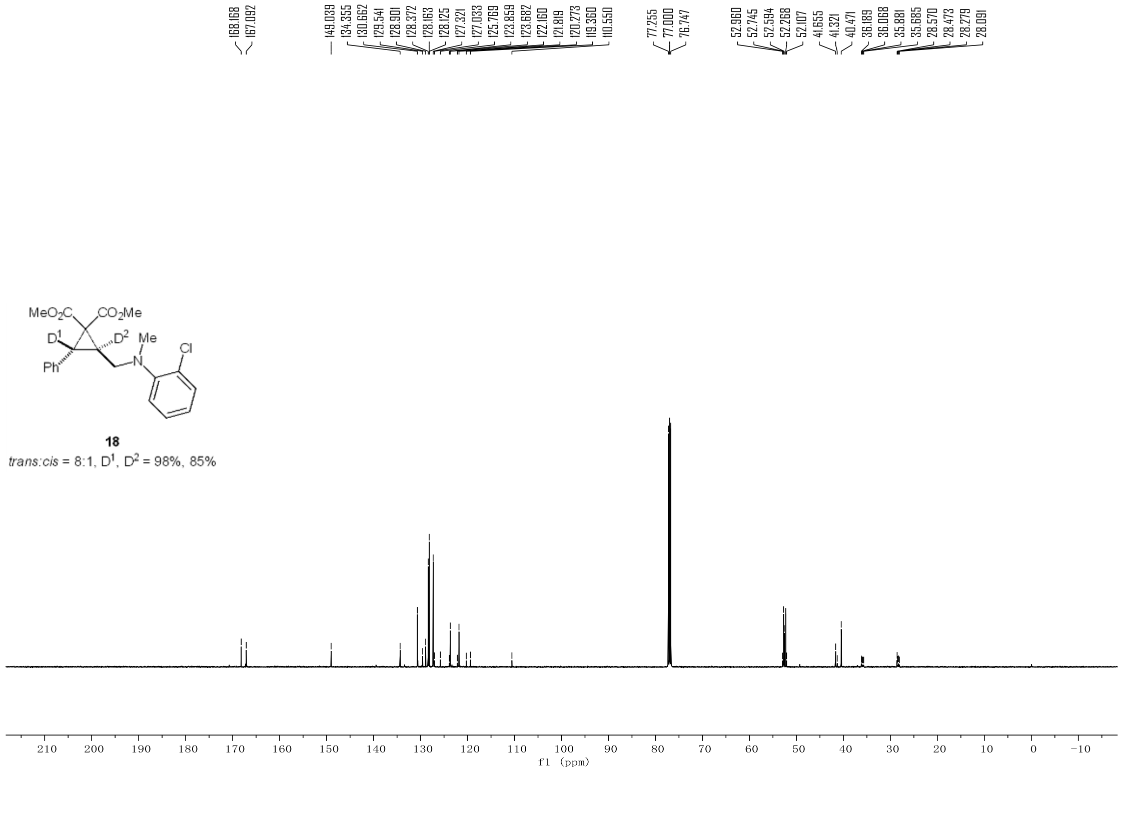
MeO2C
CO2Me
D1
D2
Me
Cl
Ph
N
18
trans:cis = 8:1, D1, D2 = 98%, 85%
f1 (ppm)

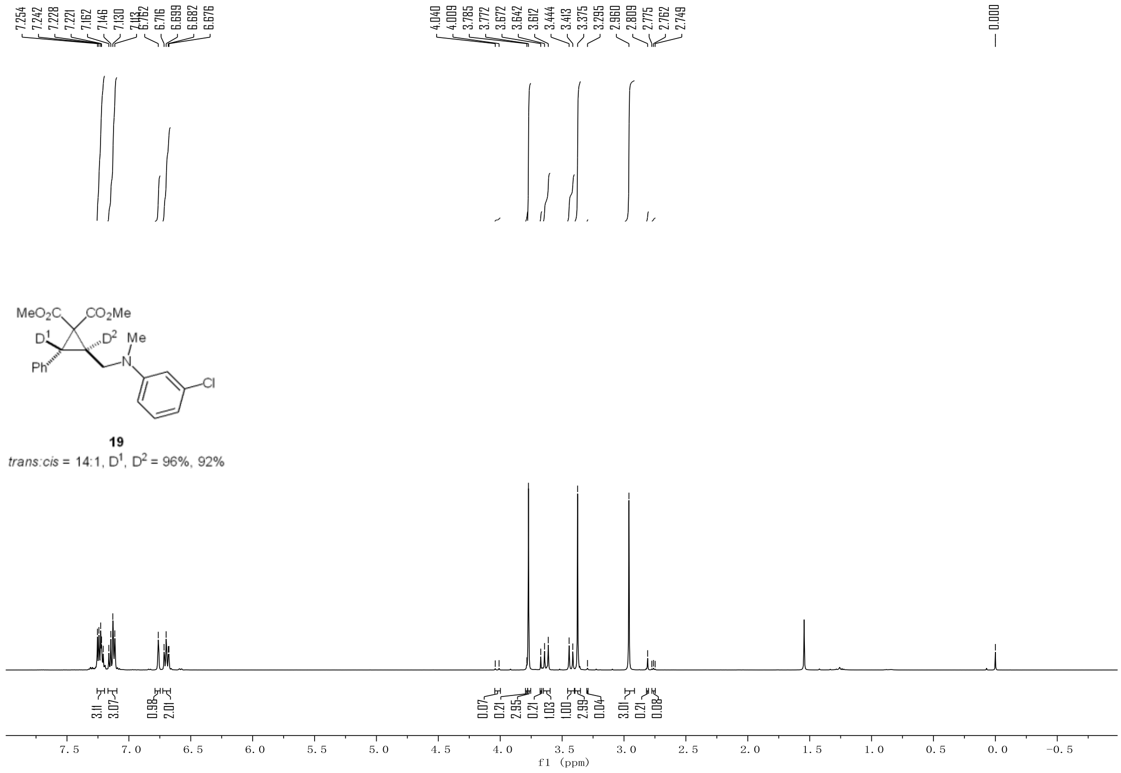

19
trans:cis = 14:1, D^1, D^2 = 96%, 92%

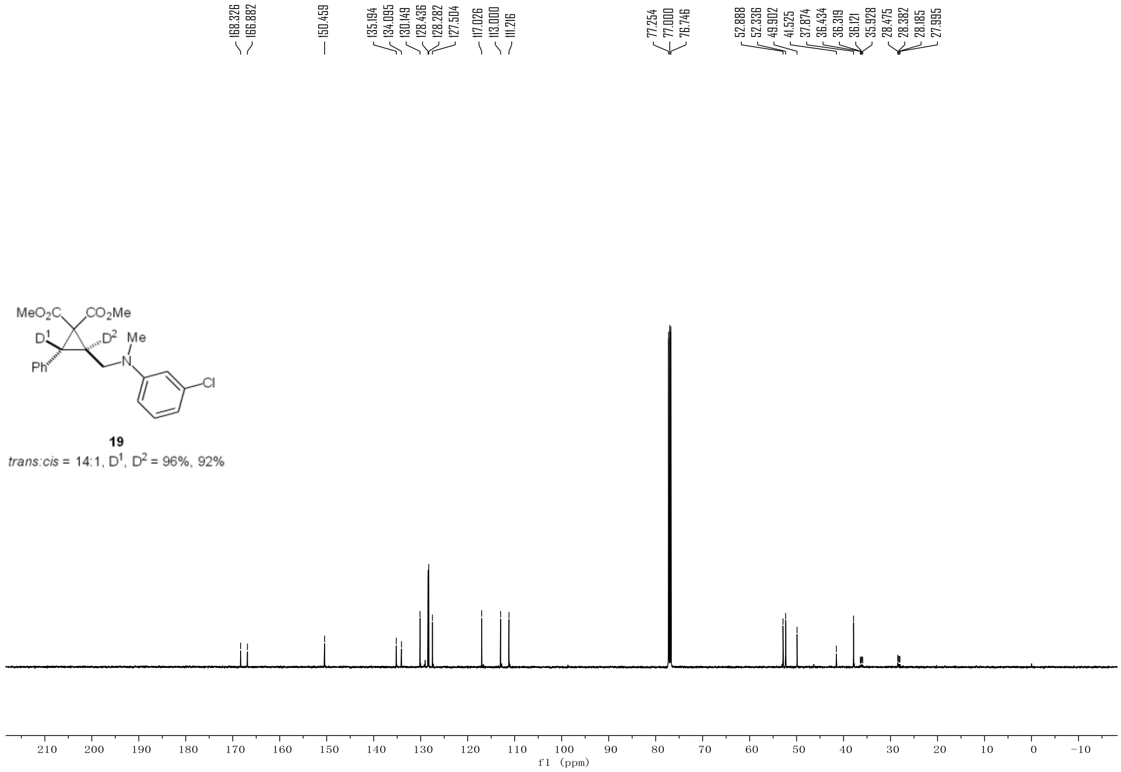

19
trans:cis = 14:1, D^1, D^2 = 96%, 92%

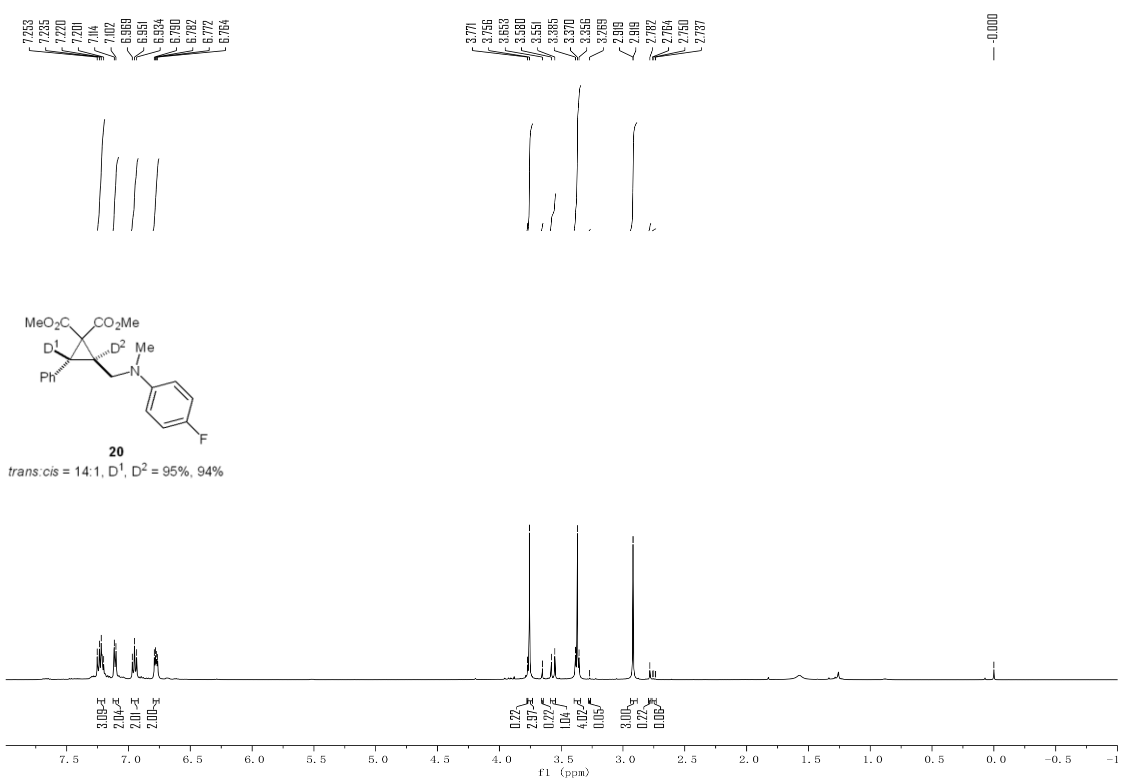
7.253 7.235 7.220 7.201 7.114 7.102 6.969 6.951 6.934 6.790 6.782 6.772 6.764
3.771 3.756 3.653 3.580 3.551 3.385 3.370 3.356 3.269 2.919 2.919 2.782 2.764 2.750 2.737
-0.000
MeO2C CO2Me D1 D2 Me N Ph F
20
trans:cis = 14:1, D1, D2 = 95%, 94%
3.09 2.04 2.01 2.00
0.22 2.97 0.22 1.04 4.02 0.05 3.00 0.22 0.06
7.5 7.0 6.5 6.0 5.5 5.0 4.5 4.0 3.5 3.0 2.5 2.0 1.5 1.0 0.5 0.0 -0.5 -1
f1 (ppm)

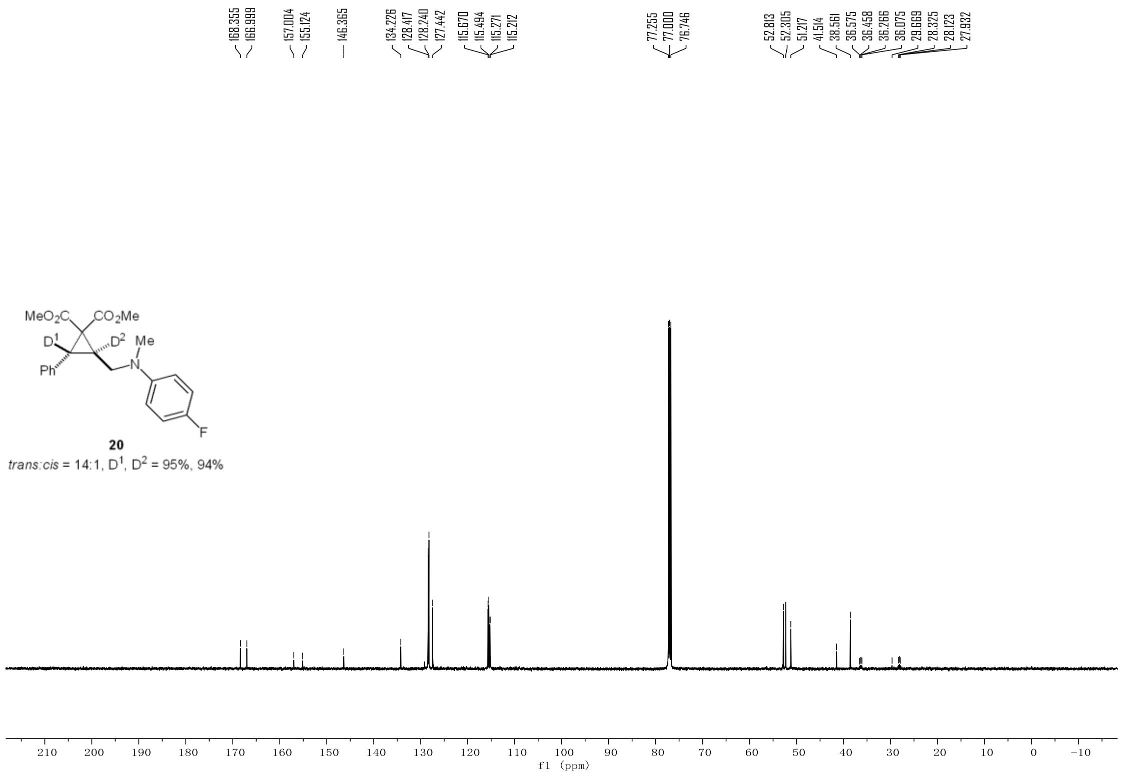
168.355 166.999 157.004 155.124 146.365 134.226 128.417 128.240 127.442 115.670 115.494 115.271 115.212
77.255 77.000 76.746
52.813 52.305 51.217 41.514 38.561 36.575 36.458 36.266 36.075 29.669 28.325 28.123 27.932
MeO2C CO2Me D1 D2 Me N Ph F
20
trans:cis = 14:1, D1, D2 = 95%, 94%
210 200 190 180 170 160 150 140 130 120 110 100 90 80 70 60 50 40 30 20 10 0 -10
f1 (ppm)

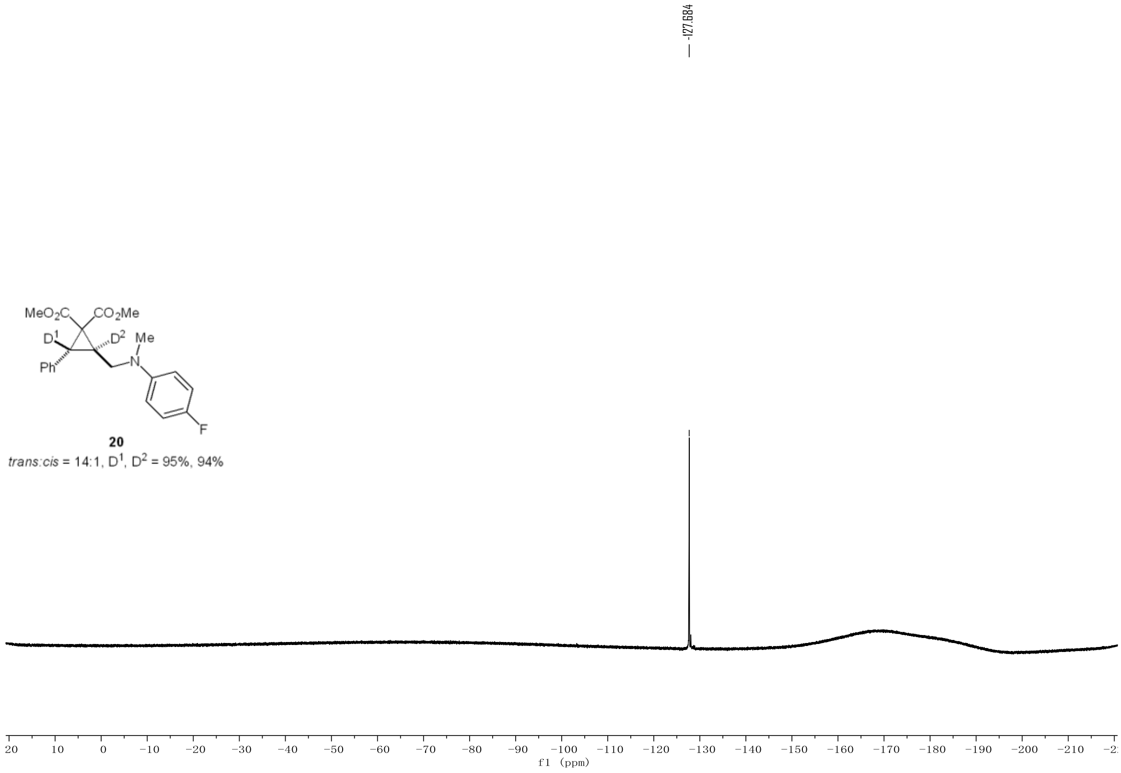

20

trans:cis = 14:1, D^1, D^2 = 95%, 94%

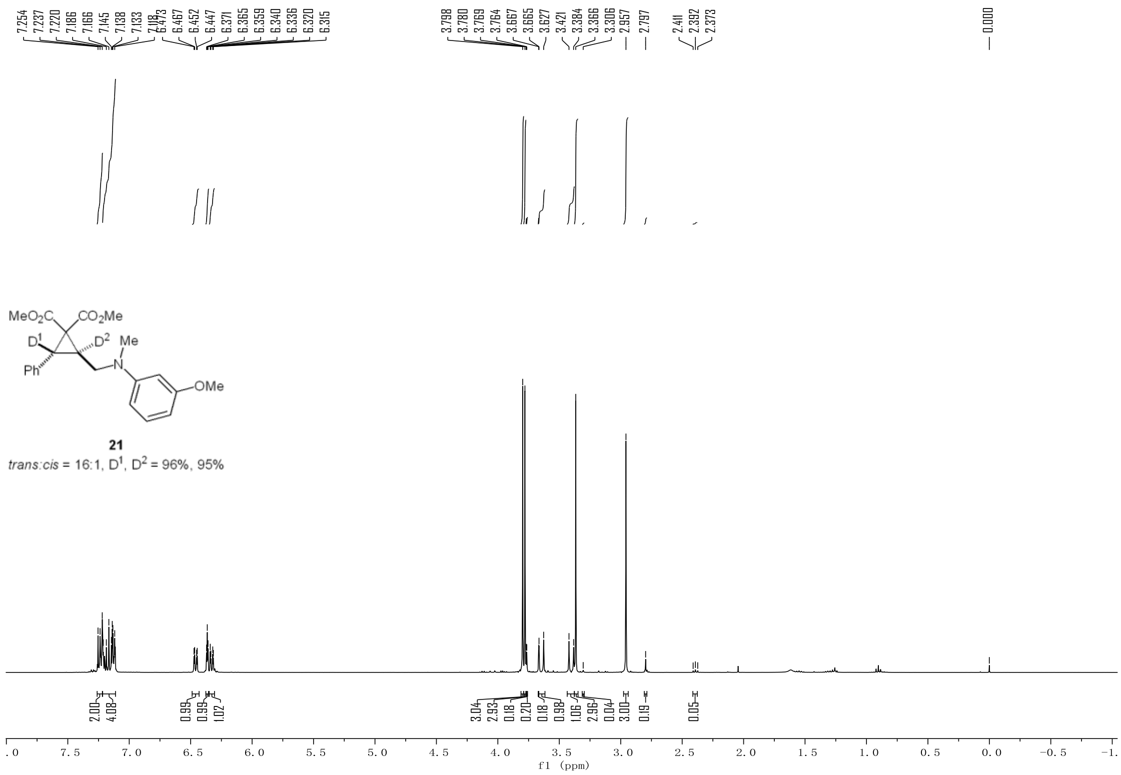

21

trans:cis = 16:1, D^1, D^2 = 96%, 95%

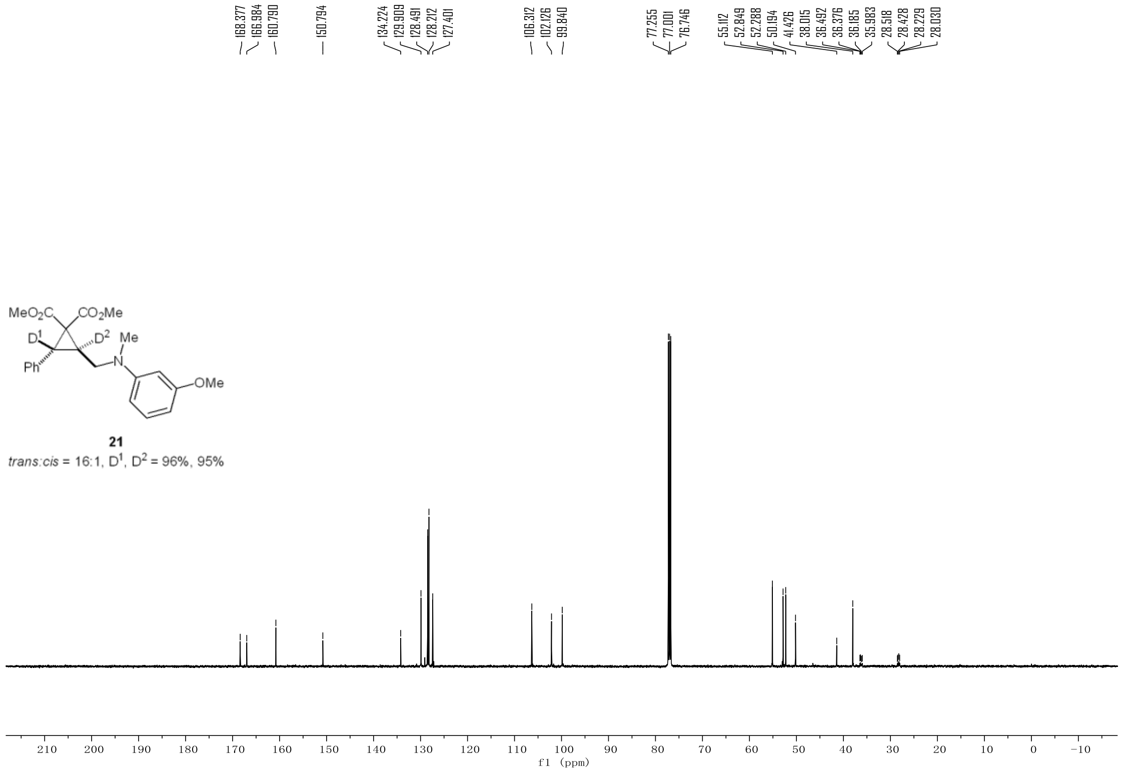

168.377
166.984
160.790
150.794
134.224
129.909
128.491
128.212
127.401
106.312
102.126
99.840
77.255
77.001
76.746
55.112
52.849
52.288
50.194
41.426
38.015
36.492
36.376
36.185
35.983
28.518
28.428
28.229
28.030
MeO2C
CO2Me
D1
D2
Me
N
Ph
OMe
21
trans:cis = 16:1, D1, D2 = 96%, 95%
210 200 190 180 170 160 150 140 130 120 110 100 90 80 70 60 50 40 30 20 10 0 −10
f1 (ppm)

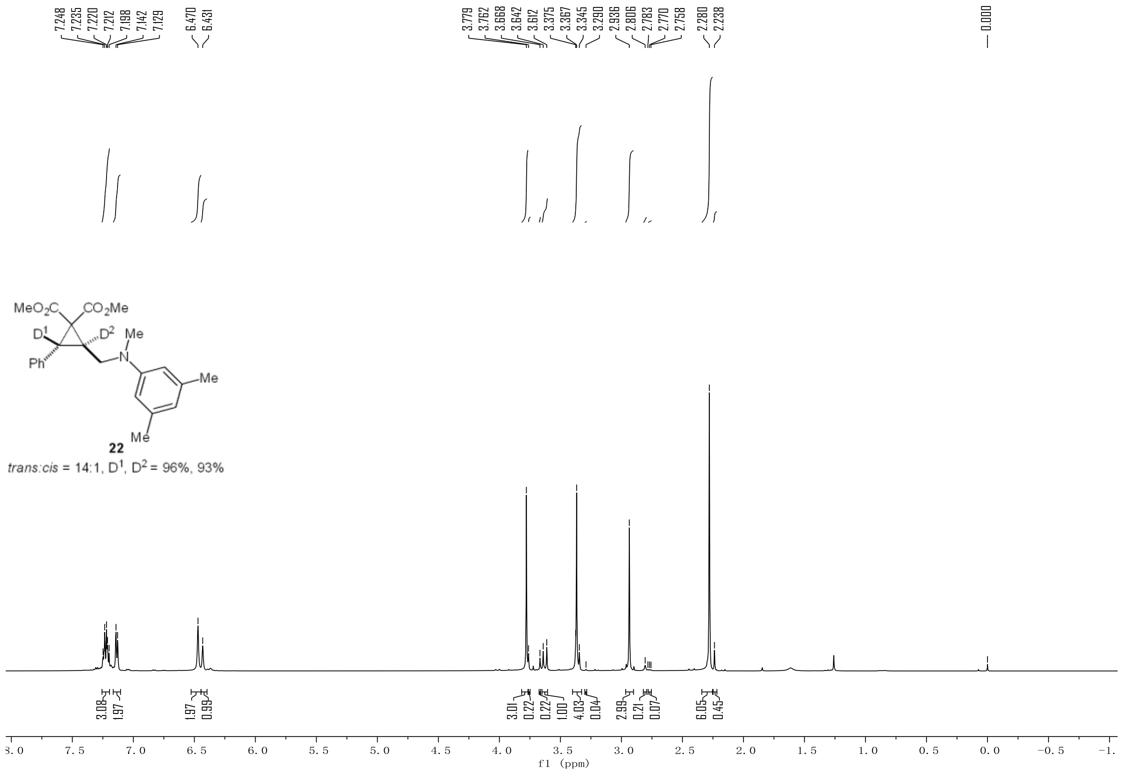

7.248
7.235
7.220
7.212
7.198
7.142
7.129
6.470
6.431
3.779
3.762
3.668
3.642
3.612
3.375
3.367
3.345
3.290
2.936
2.806
2.783
2.770
2.758
2.280
2.238
0.000
MeO2C
CO2Me
D1
D2
Me
N
Ph
Me
Me
22
trans:cis = 14:1, D1, D2 = 96%, 93%
3.08
1.97
1.97
0.99
3.01
0.22
0.22
1.00
4.03
0.04
2.99
0.21
0.07
6.05
0.45
7.5 7.0 6.5 6.0 5.5 5.0 4.5 4.0 3.5 3.0 2.5 2.0 1.5 1.0 0.5 0.0 −0.5
f1 (ppm)

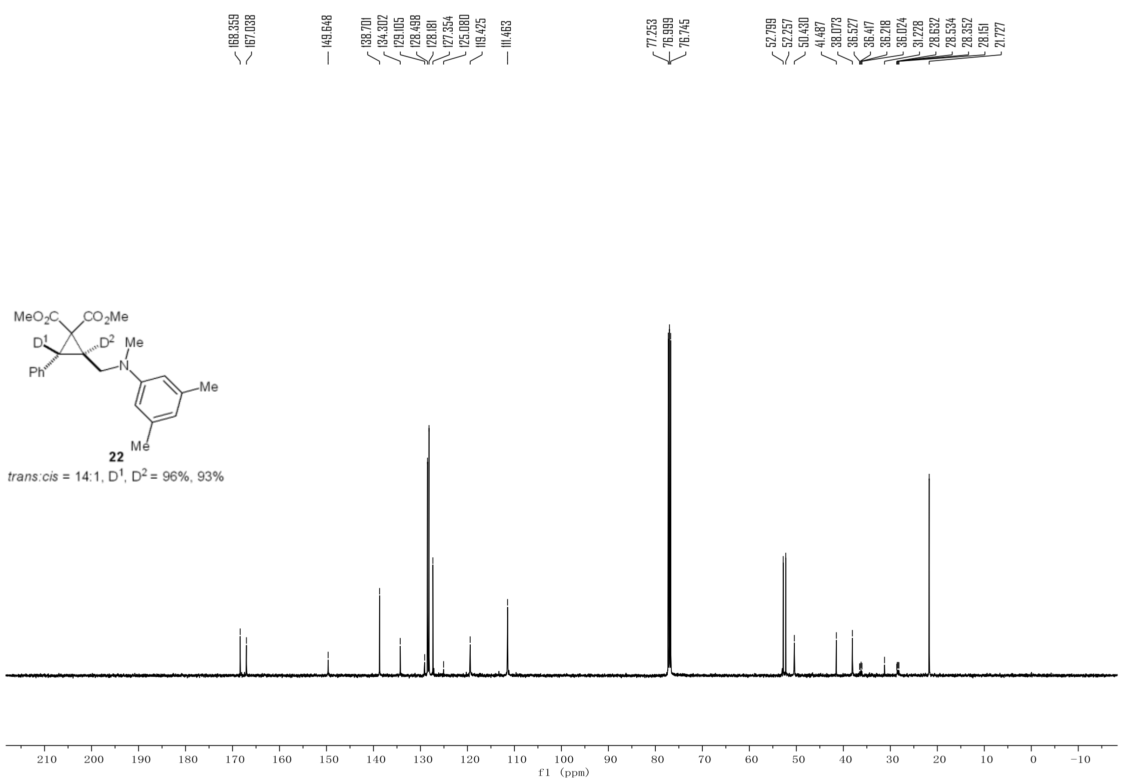
168.359
167.038
149.648
138.701
134.302
129.105
128.498
128.181
127.354
125.080
119.425
111.463
77.253
76.999
76.745
52.799
52.257
50.430
41.487
38.073
36.527
36.417
36.218
36.024
31.228
28.632
28.534
28.352
28.151
21.727
MeO2C
CO2Me
D1
D2
Me
N
Ph
Me
Me
22
trans:cis = 14:1, D1, D2 = 96%, 93%
f1 (ppm)

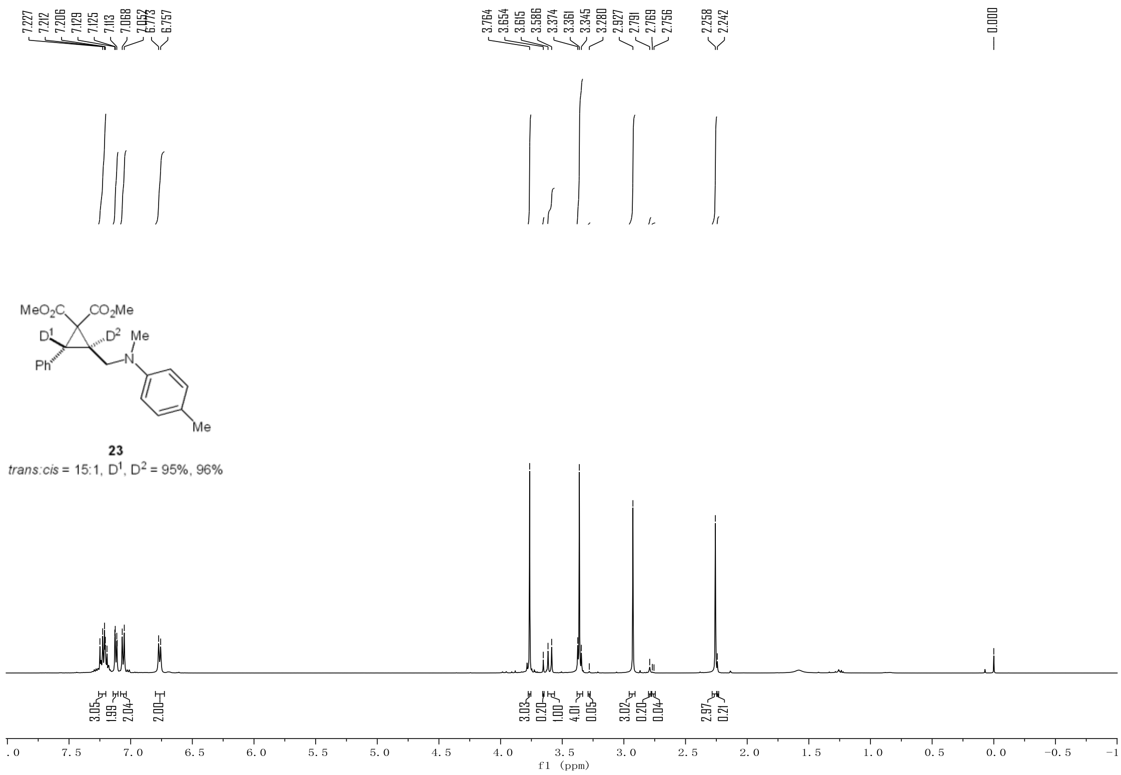
7.227
7.212
7.206
7.129
7.125
7.113
7.068
7.052
6.773
6.757
3.764
3.654
3.615
3.586
3.374
3.361
3.345
3.280
2.927
2.791
2.769
2.756
2.258
2.242
0.000
MeO2C
CO2Me
D1
D2
Me
N
Ph
Me
23
trans:cis = 15:1, D1, D2 = 95%, 96%
3.05
1.99
2.04
2.00
3.03
0.20
1.00
4.01
0.05
3.02
0.20
0.04
2.97
0.21
f1 (ppm)

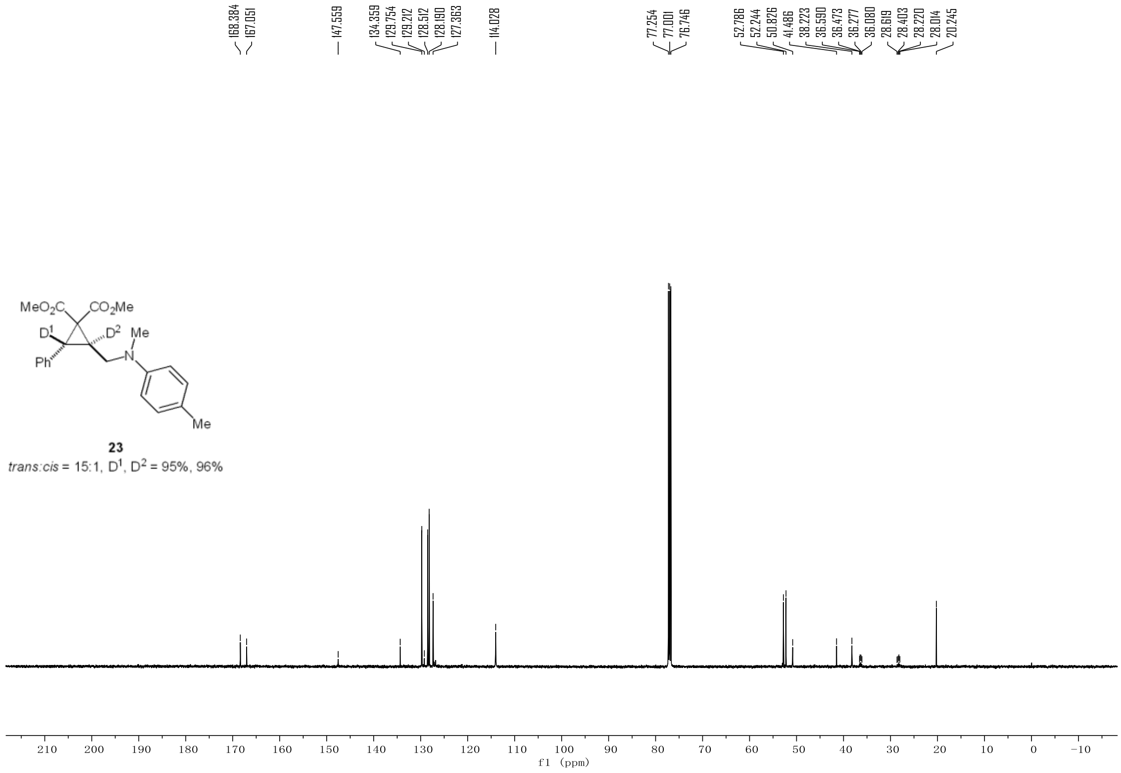
MeO2C
CO2Me
D1
D2
Me
N
Ph
Me
23
trans:cis = 15:1, D1, D2 = 95%, 96%
f1 (ppm)

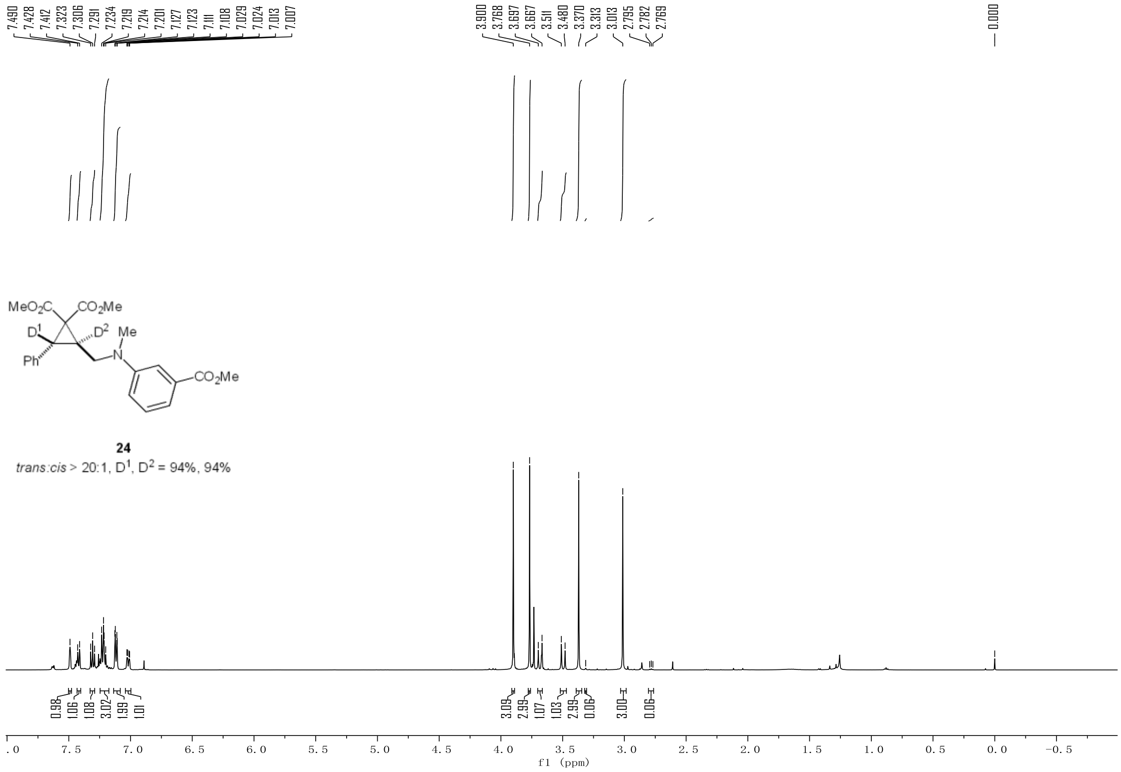
MeO2C
CO2Me
D1
D2
Me
N
Ph
CO2Me
24
trans:cis > 20:1, D1, D2 = 94%, 94%
f1 (ppm)

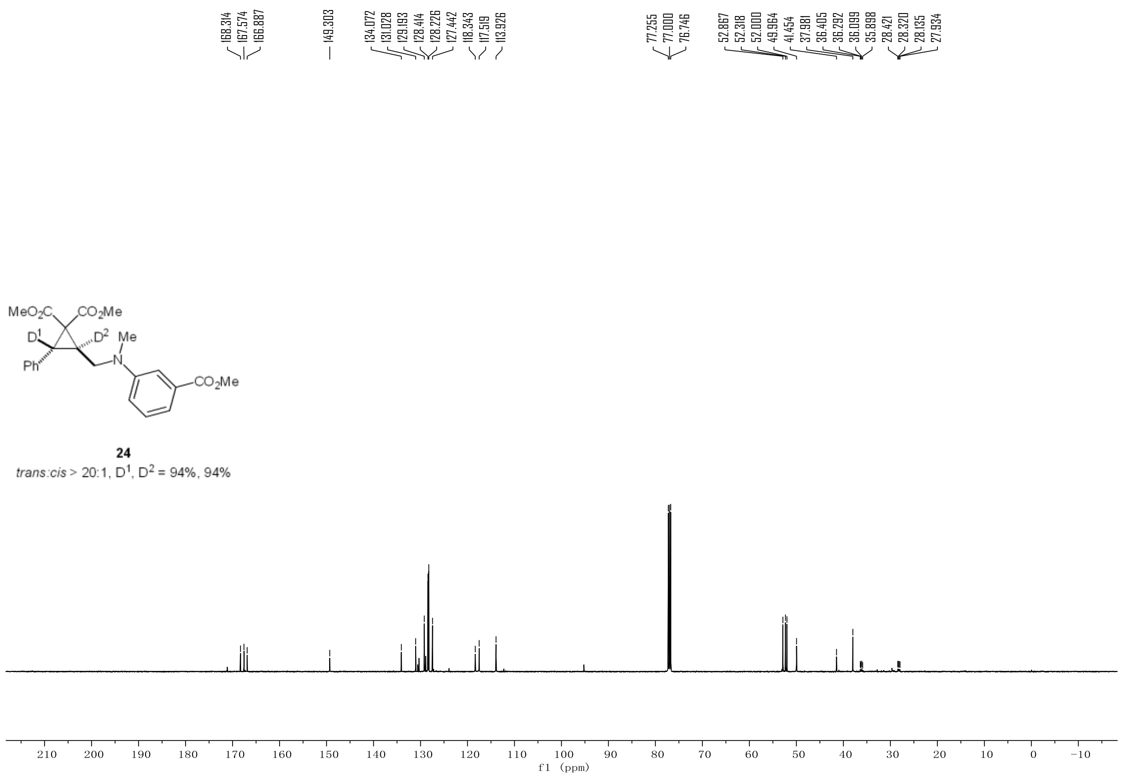

24
trans:*cis* > 20:1, D^1, D^2 = 94%, 94%

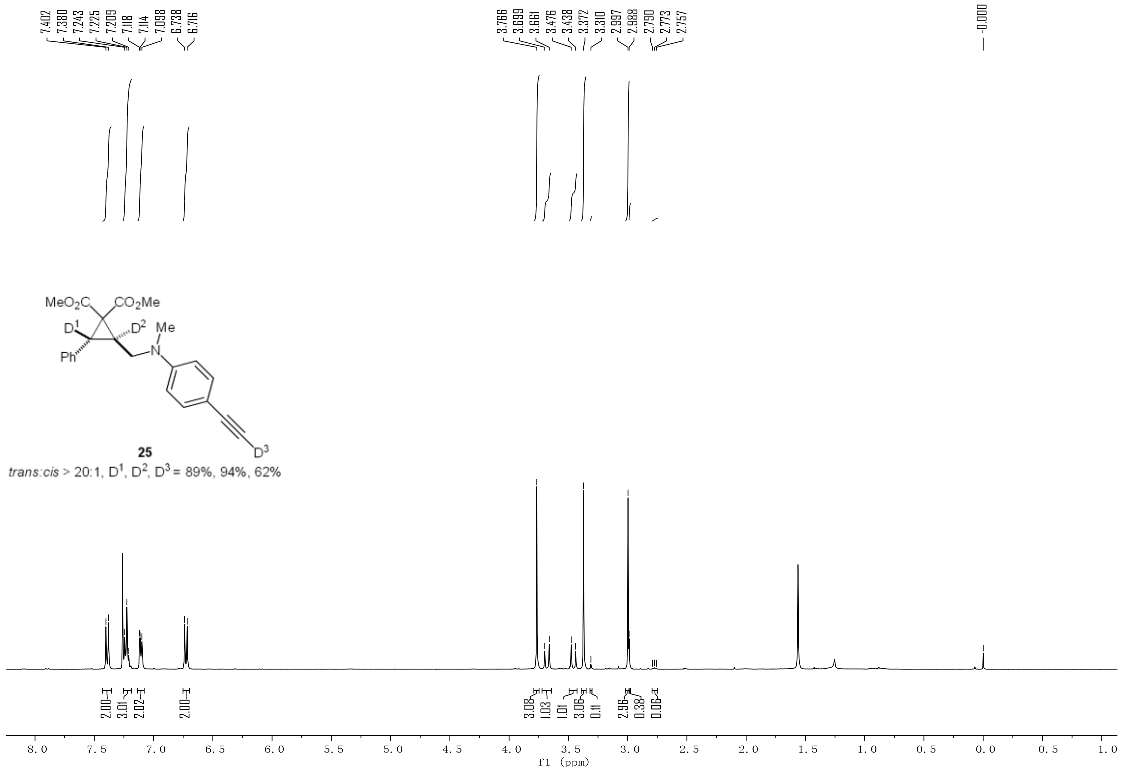

25
trans:*cis* > 20:1, D^1, D^2, D^3 = 89%, 94%, 62%

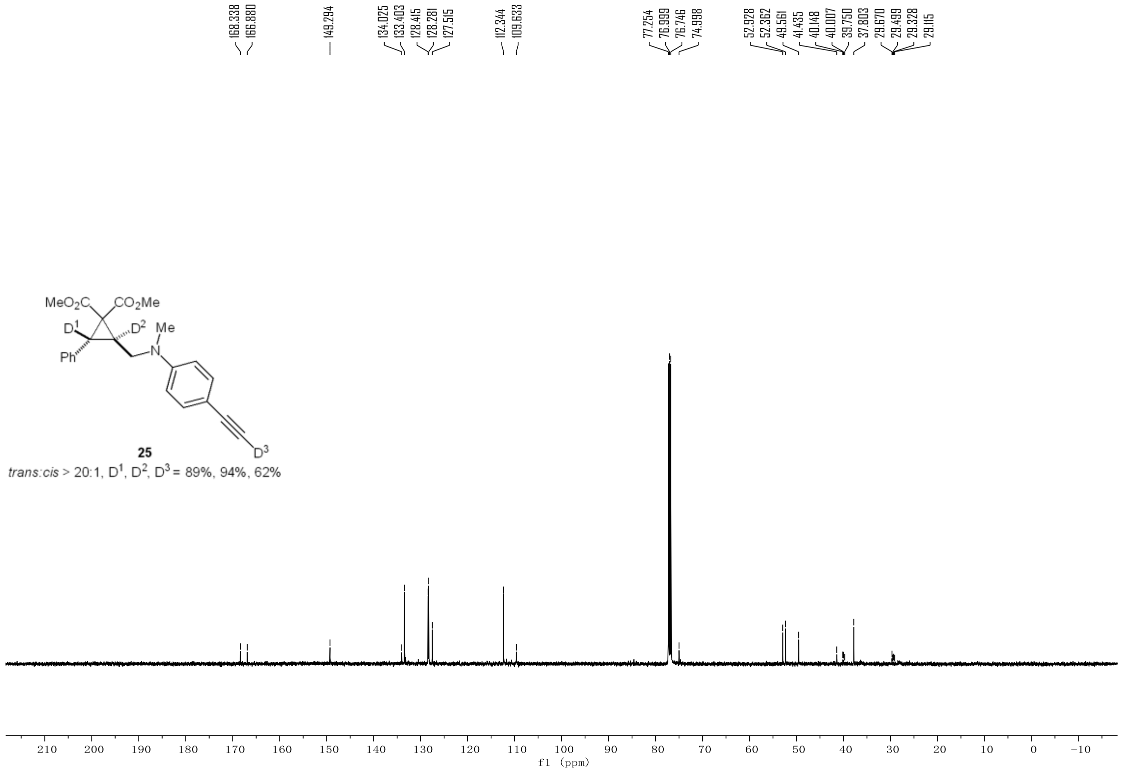

25
trans:cis > 20:1, D[1], D[2], D[3] = 89%, 94%, 62%

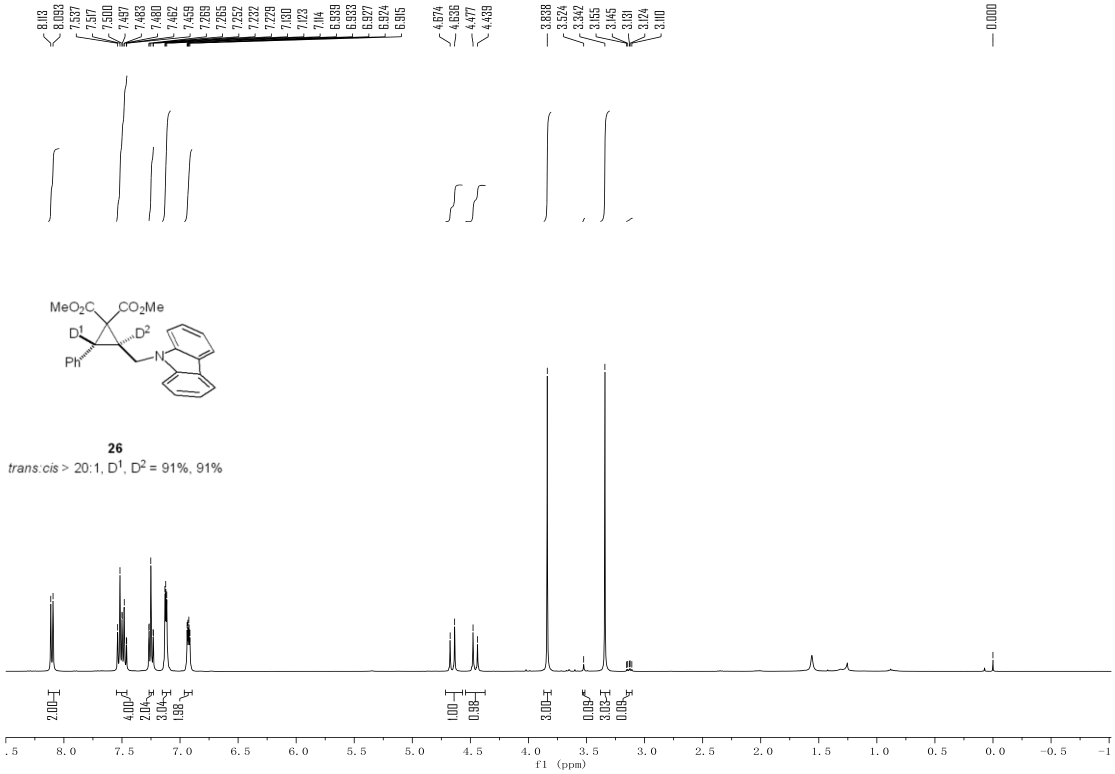

26
trans:cis > 20:1, D[1], D[2] = 91%, 91%

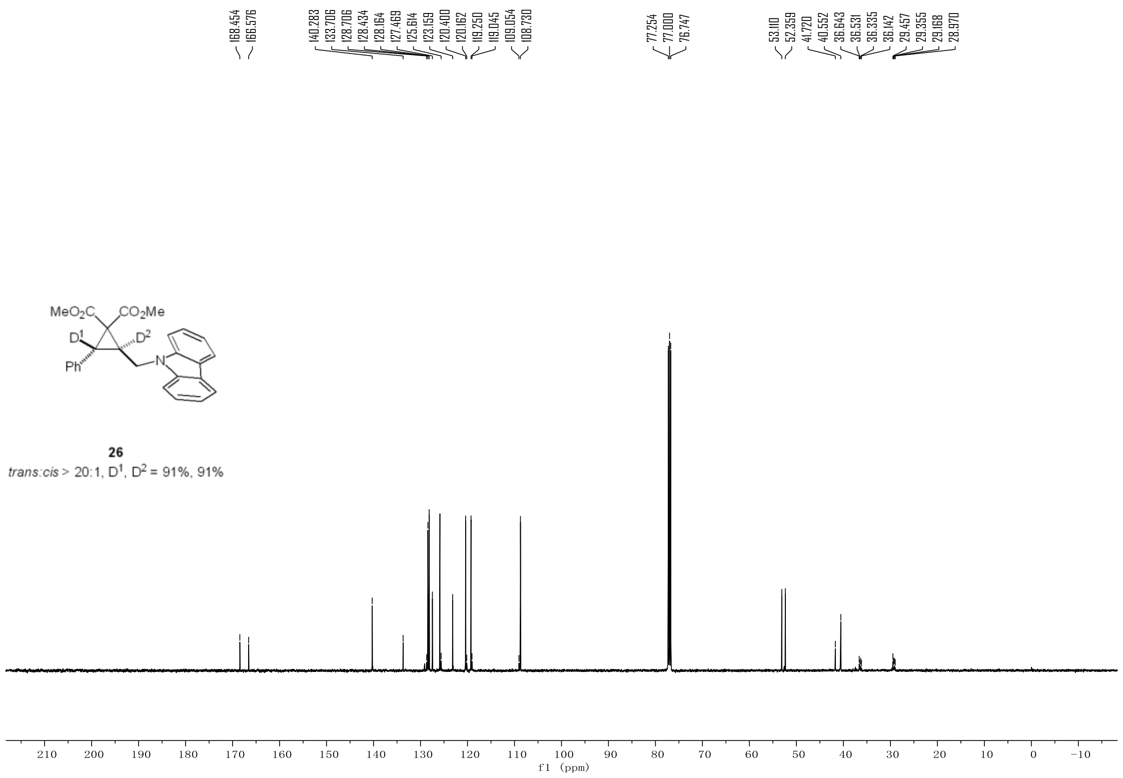
MeO2C CO2Me
D1 D2
Ph N
26
trans:cis > 20:1, D1, D2 = 91%, 91%
f1 (ppm)

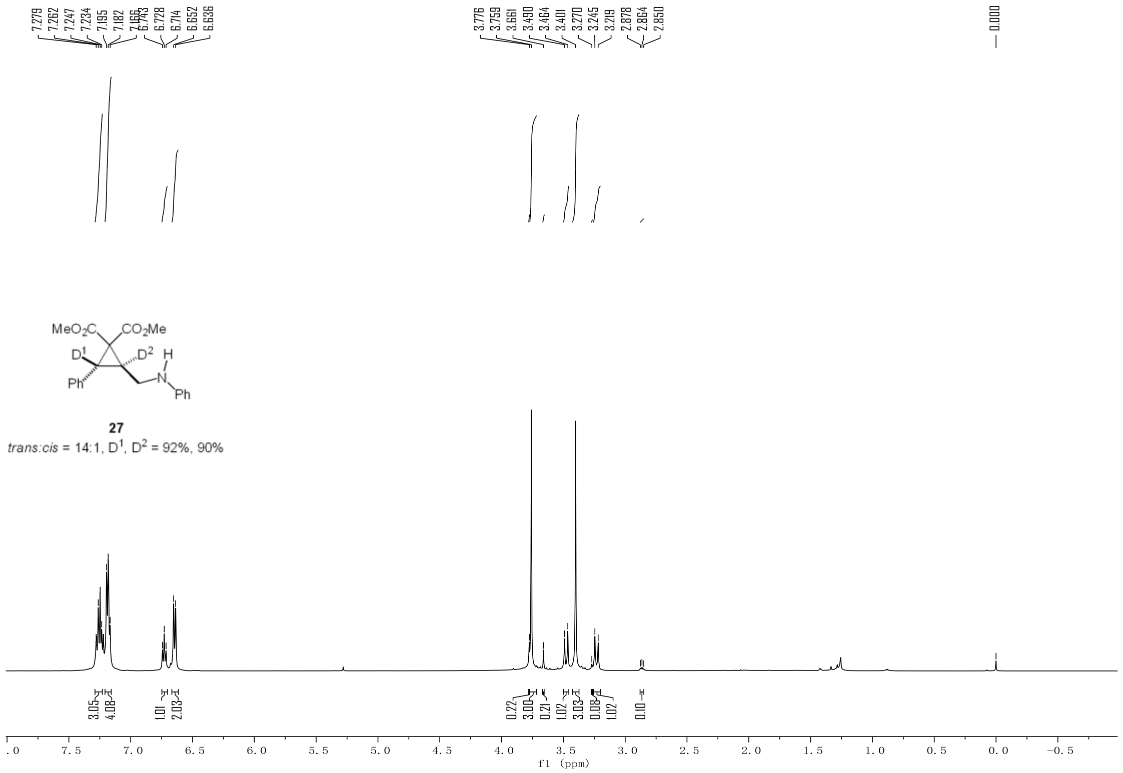
MeO2C CO2Me
D1 D2 H
Ph N
Ph
27
trans:cis = 14:1, D1, D2 = 92%, 90%
f1 (ppm)

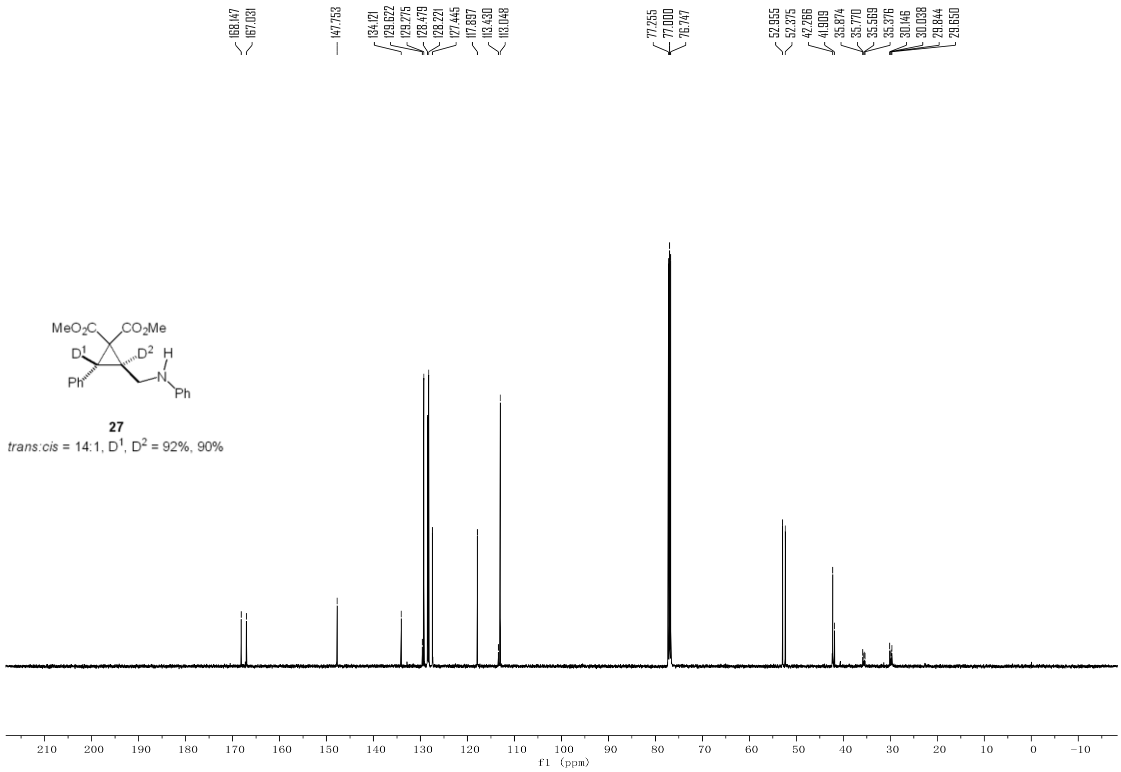

27

trans:cis = 14:1, D^1, D^2 = 92%, 90%

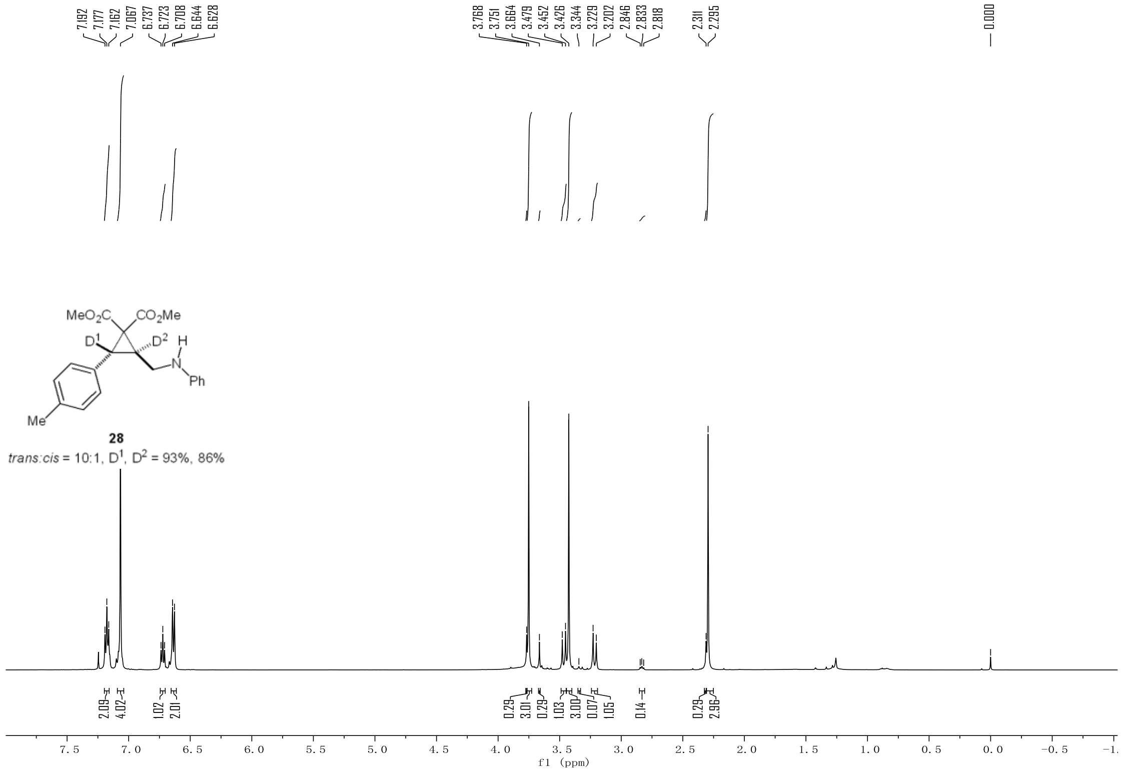

28

trans:cis = 10:1, D^1, D^2 = 93%, 86%

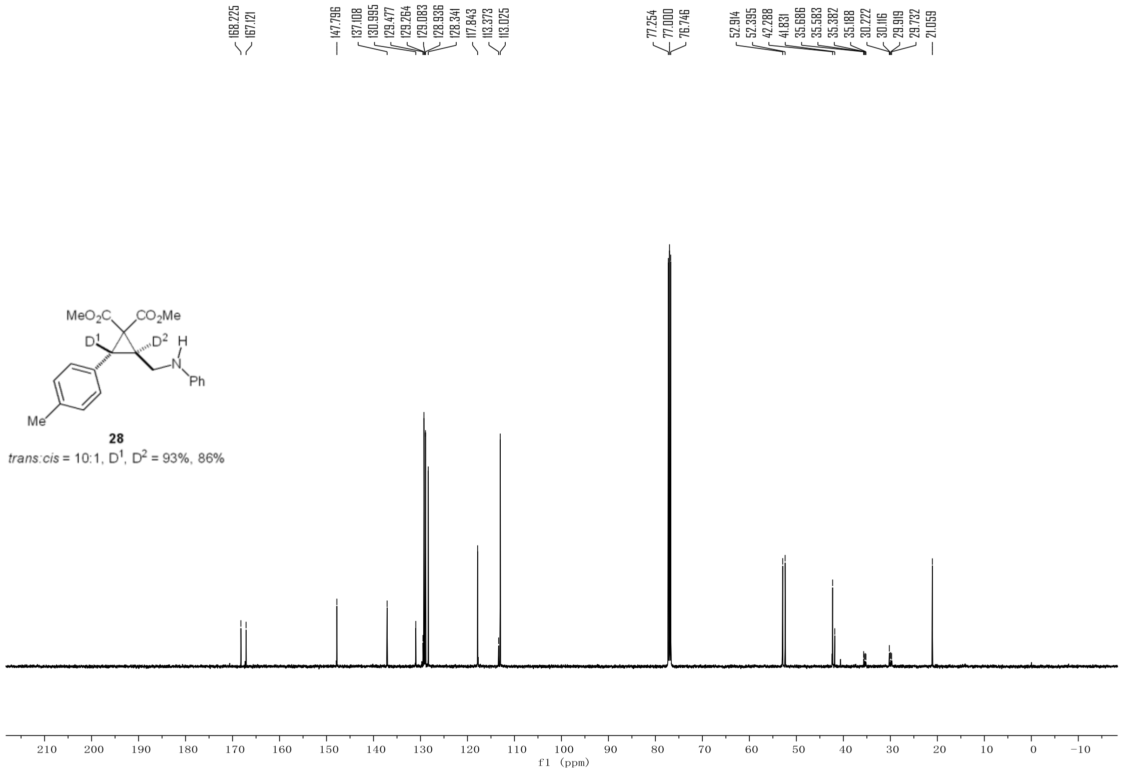

28
trans:cis = 10:1, D[1], D[2] = 93%, 86%

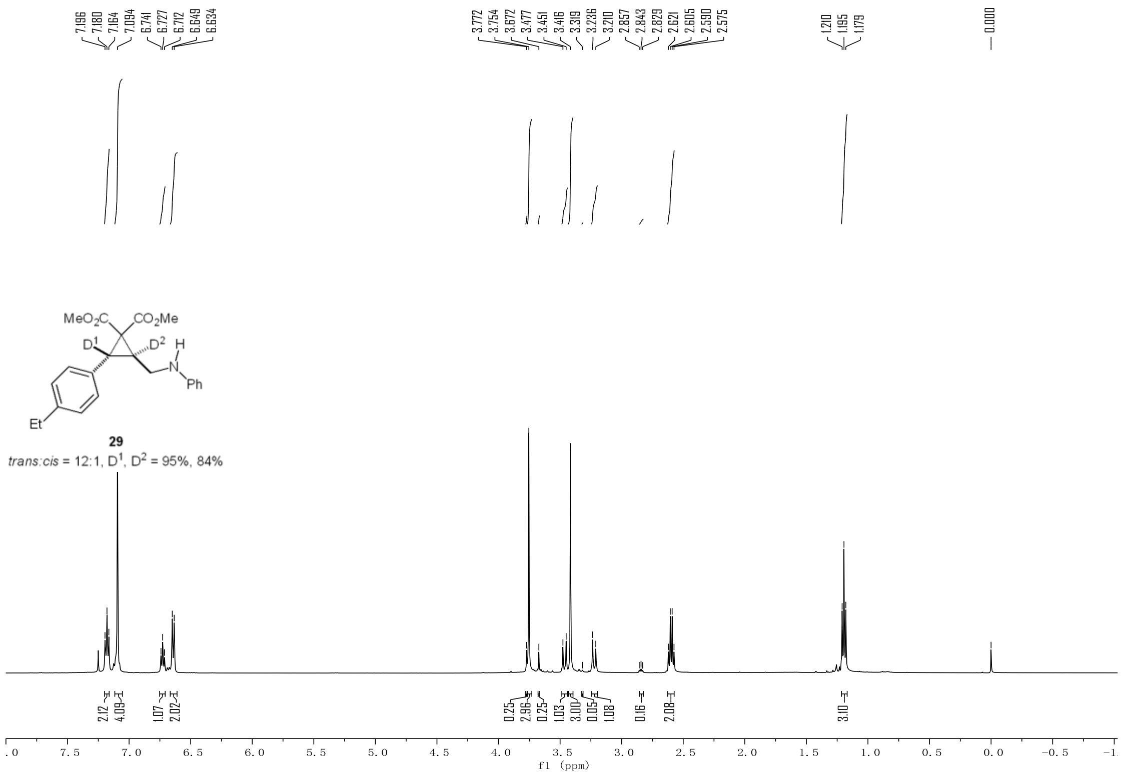

29
trans:cis = 12:1, D[1], D[2] = 95%, 84%

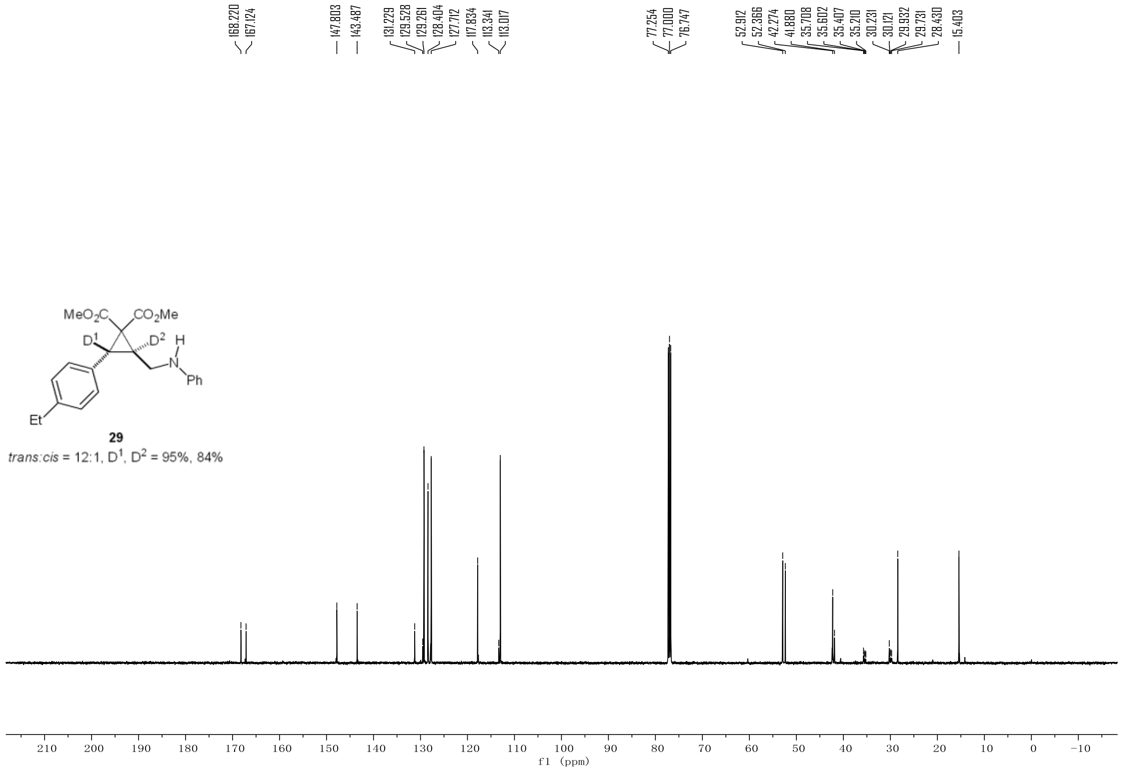
168.220 167.124 147.803 143.487 131.229 129.528 129.261 128.404 127.712 117.834 113.341 113.017 77.254 77.000 76.747 52.912 52.366 42.274 41.880 35.708 35.602 35.407 35.210 30.231 30.121 29.932 29.731 28.430 15.403
MeO2C CO2Me
D1 D2 H
N
Ph
Et
29
trans:cis = 12:1, D1, D2 = 95%, 84%
f1 (ppm)

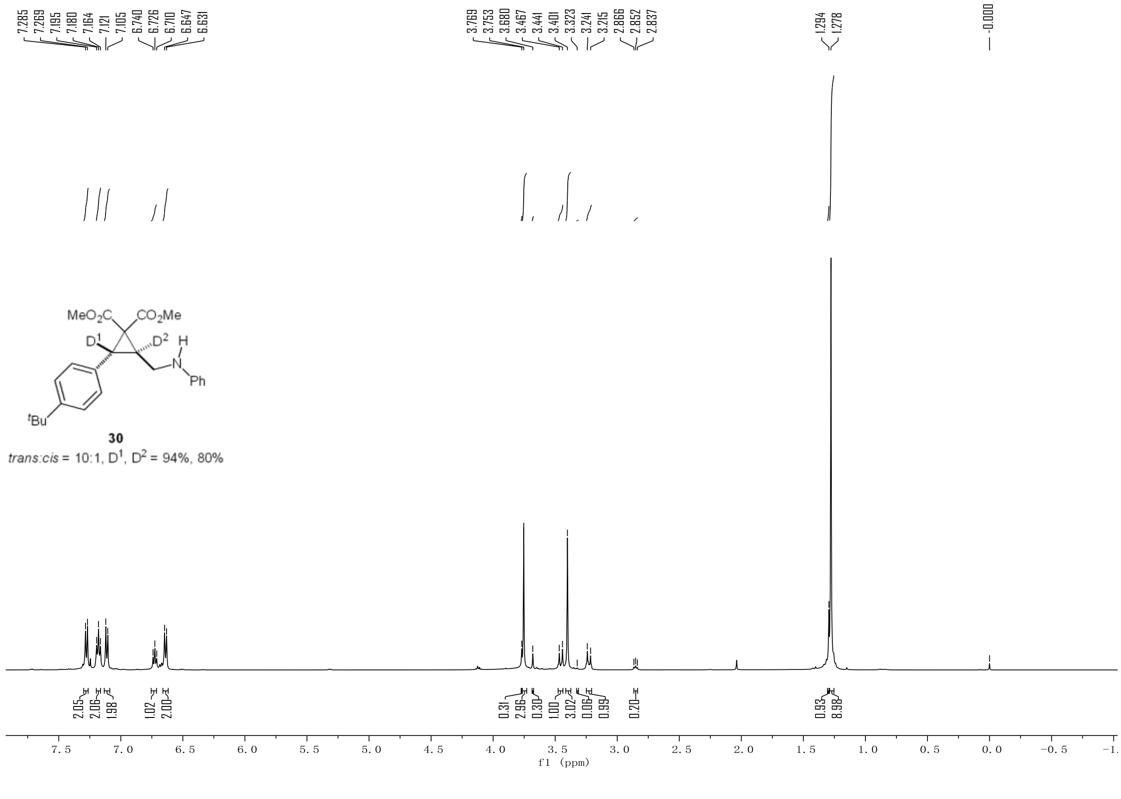
7.285 7.269 7.195 7.180 7.164 7.121 7.105 6.740 6.726 6.710 6.647 6.631 3.769 3.753 3.680 3.467 3.441 3.401 3.323 3.241 3.215 2.866 2.852 2.837 1.294 1.278 -0.000
MeO2C CO2Me
D1 D2 H
N
Ph
tBu
30
trans:cis = 10:1, D1, D2 = 94%, 80%
2.05 2.06 1.98 1.02 2.00 0.31 2.96 0.30 1.00 3.02 0.06 0.99 0.20 0.93 8.98
f1 (ppm)

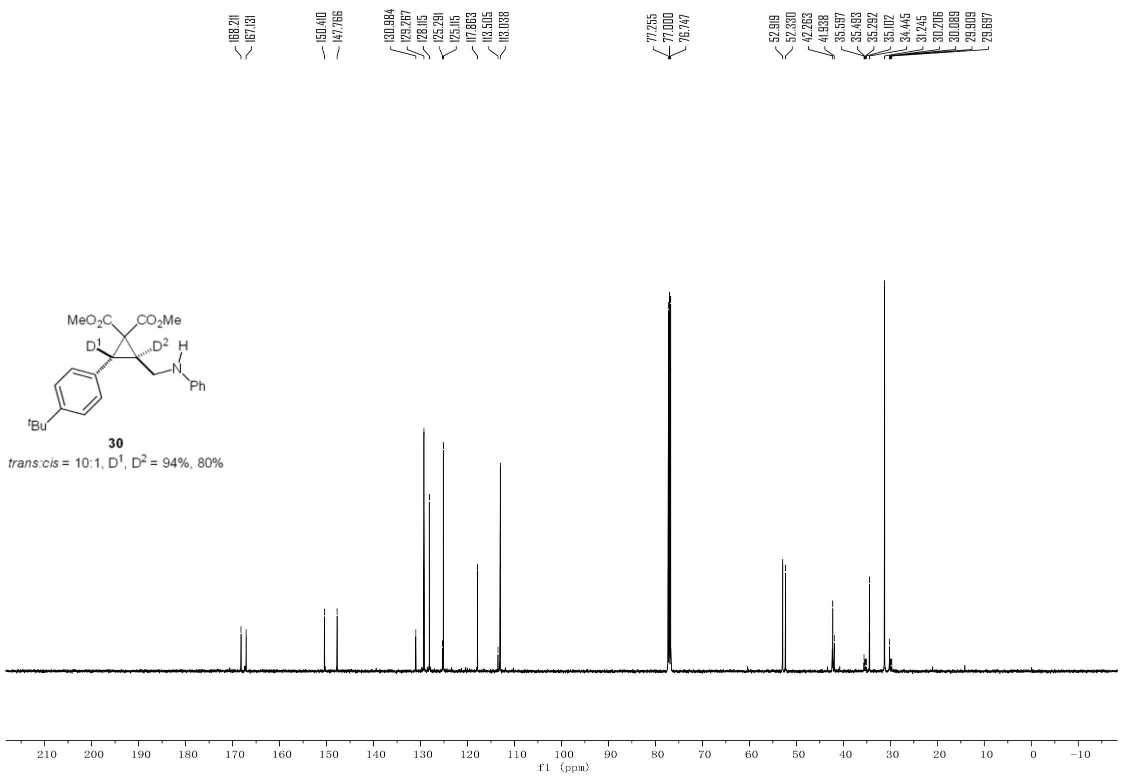
168.211
167.131
150.410
147.766
130.984
129.267
128.115
125.291
125.115
117.863
113.505
113.038
77.255
77.000
76.747
52.919
52.330
42.263
41.938
35.597
35.493
35.292
35.102
34.445
31.245
30.206
30.089
29.909
29.697
MeO2C
CO2Me
D1
D2
H
N
Ph
tBu
30
trans:cis = 10:1, D1, D2 = 94%, 80%
f1 (ppm)

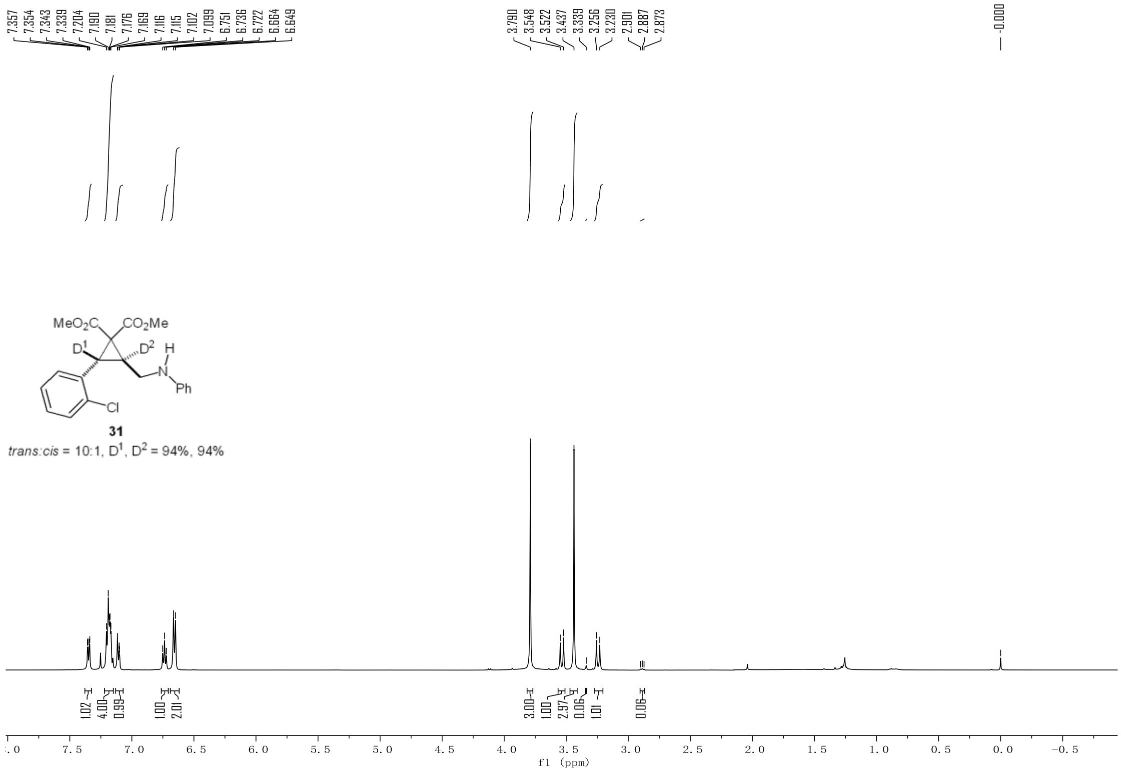
7.357
7.354
7.343
7.339
7.204
7.190
7.181
7.176
7.169
7.116
7.115
7.102
7.099
6.751
6.736
6.722
6.664
6.649
3.790
3.548
3.522
3.437
3.339
3.256
3.230
2.901
2.887
2.873
-0.000
MeO2C
CO2Me
D1
D2
H
N
Ph
Cl
31
trans:cis = 10:1, D1, D2 = 94%, 94%
1.02
4.00
0.99
1.00
2.01
3.00
1.00
2.97
0.06
1.01
0.06
f1 (ppm)

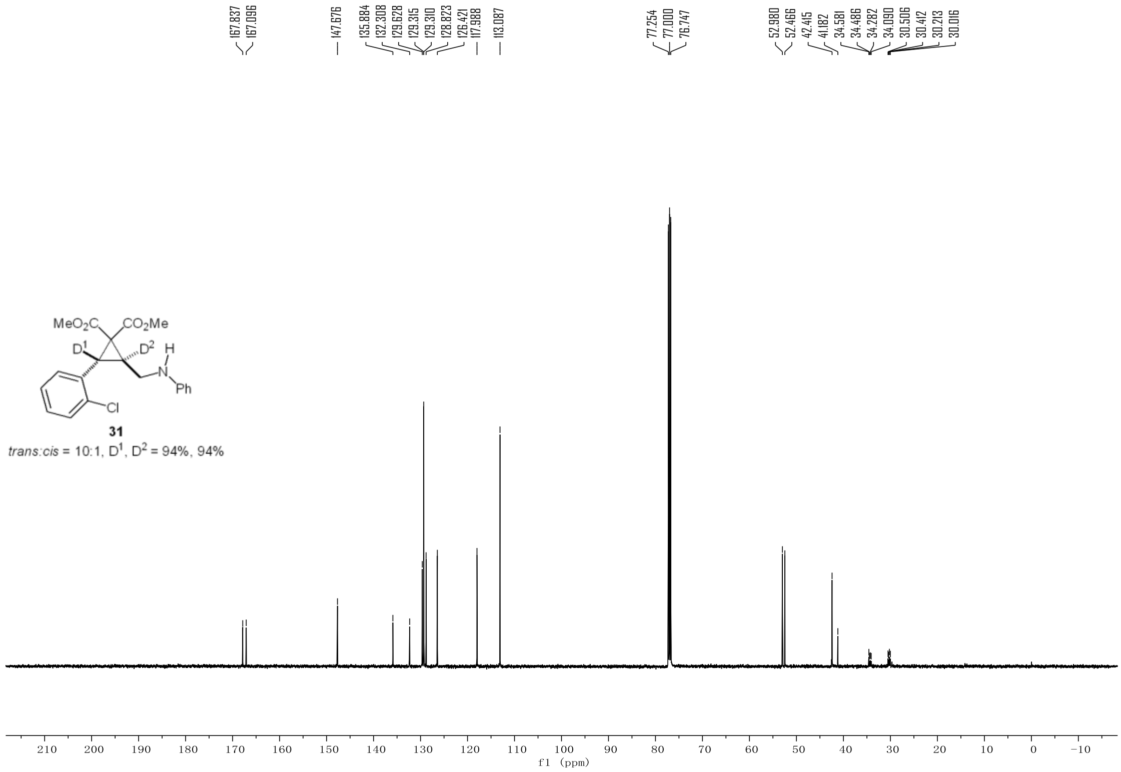

31

trans:cis = 10:1, D^1, D^2 = 94%, 94%

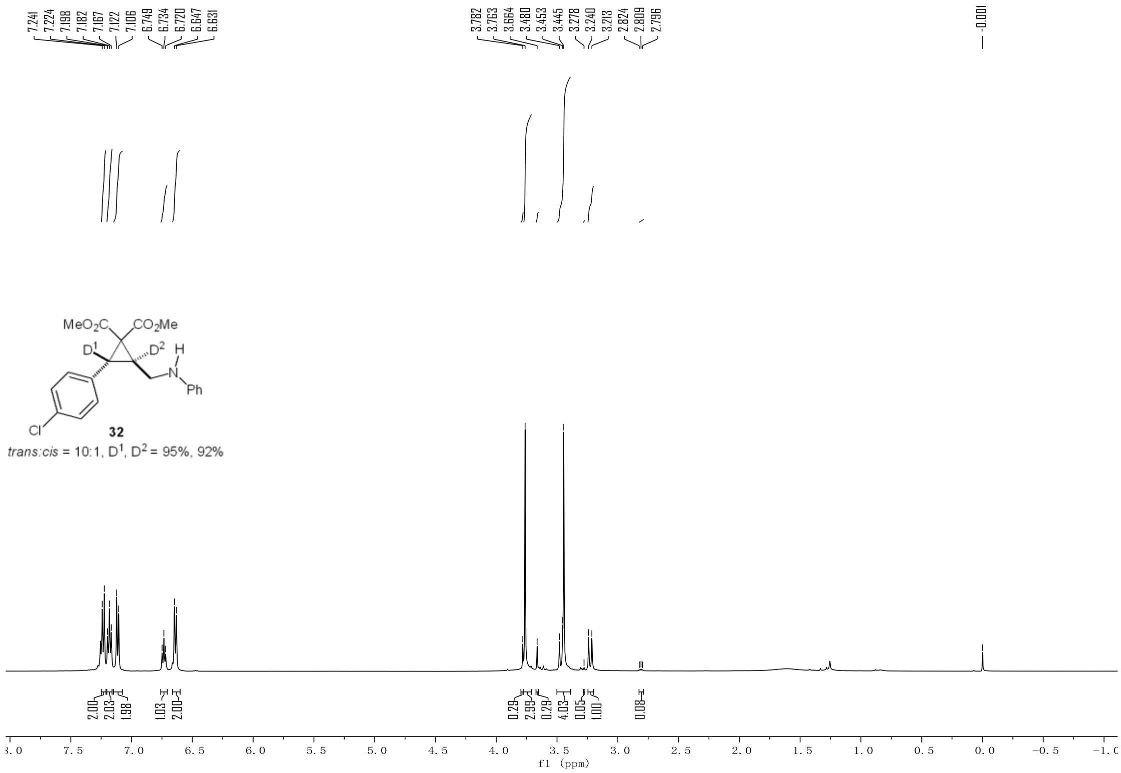

32

trans:cis = 10:1, D^1, D^2 = 95%, 92%

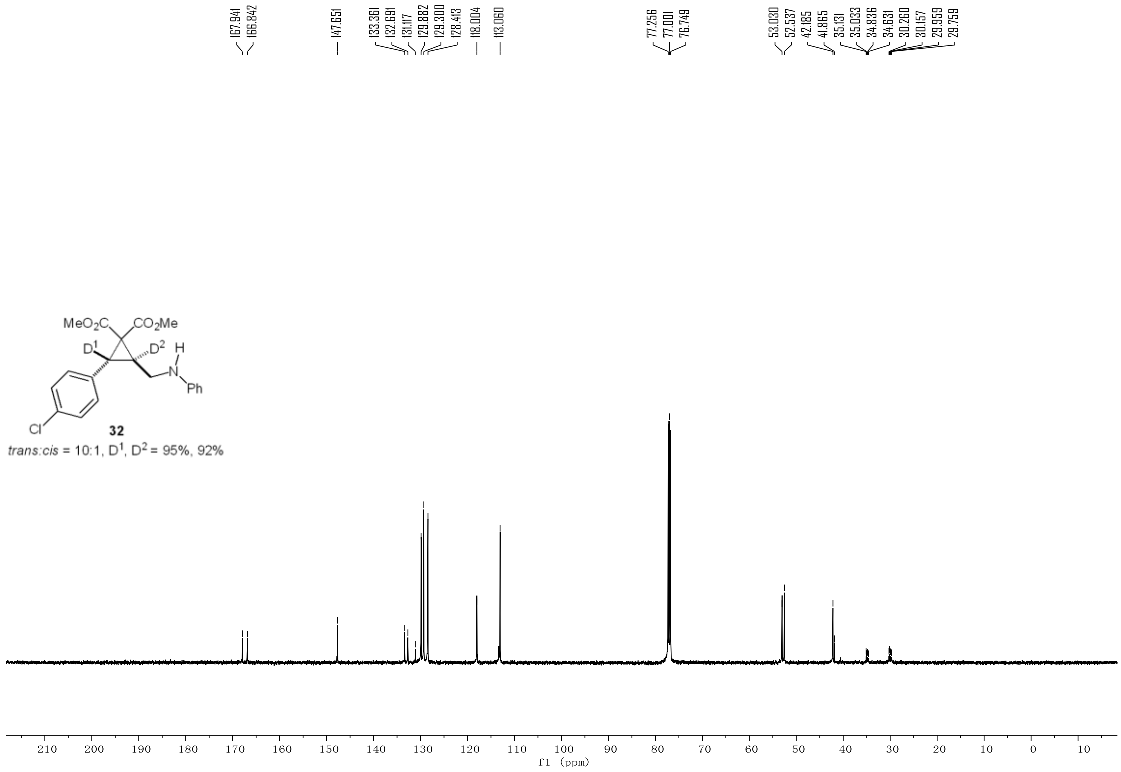

32

trans:cis = 10:1, D[1], D[2] = 95%, 92%

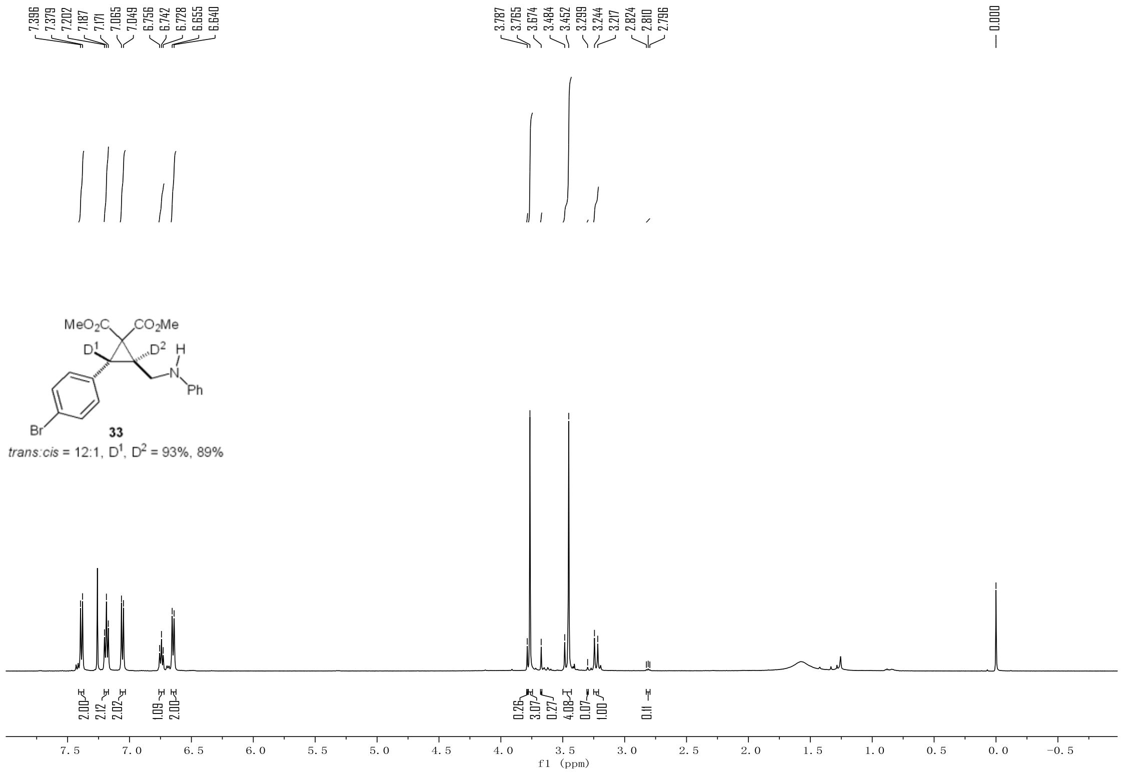

33

trans:cis = 12:1, D[1], D[2] = 93%, 89%

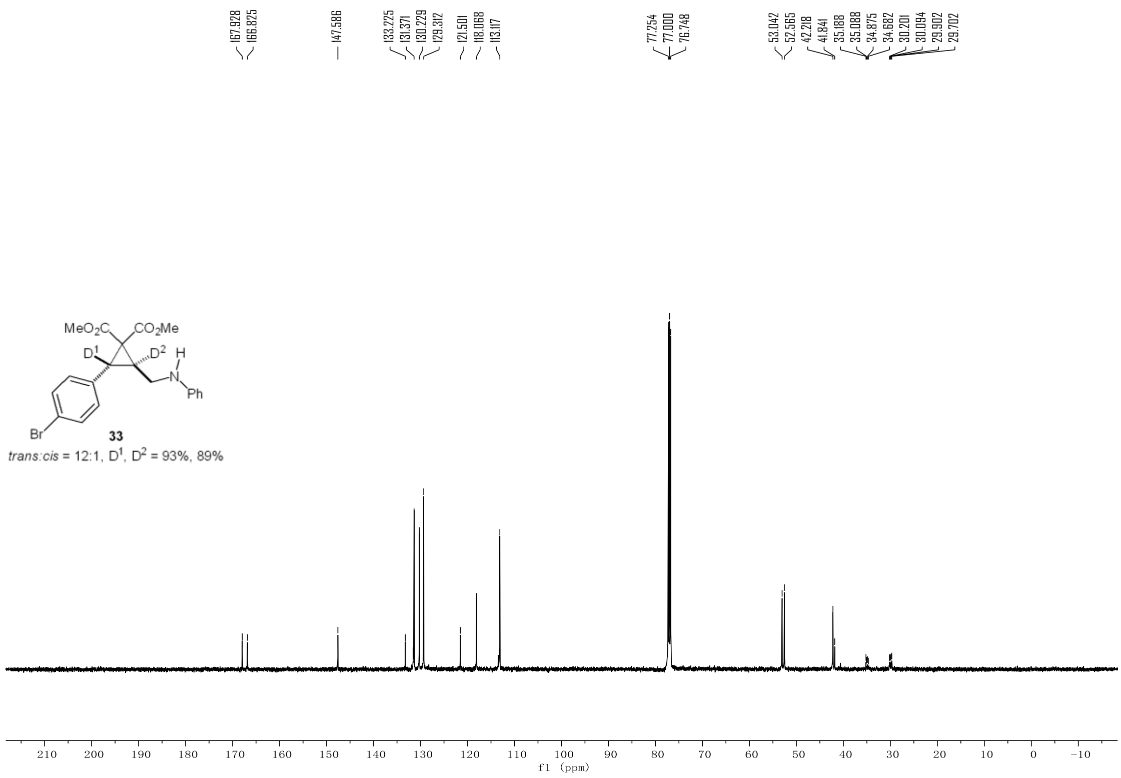

167.928
166.825
147.586
133.225
131.371
130.229
129.312
121.501
118.068
113.117
77.254
77.000
76.748
53.042
52.565
42.218
41.841
35.188
35.088
34.875
34.682
30.201
30.094
29.902
29.702
33
trans:cis = 12:1, D1, D2 = 93%, 89%
f1 (ppm)

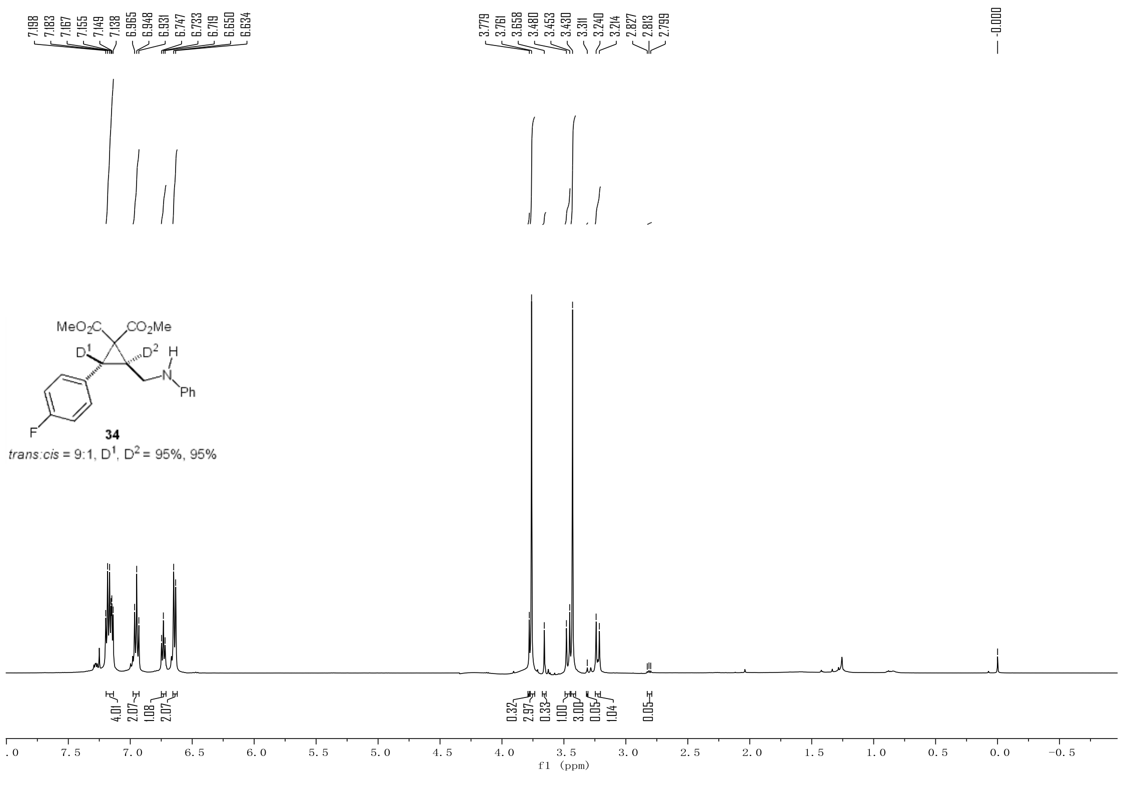

7.198
7.183
7.167
7.155
7.149
7.138
6.965
6.948
6.931
6.747
6.733
6.719
6.650
6.634
3.779
3.761
3.658
3.480
3.453
3.430
3.311
3.240
3.214
2.827
2.813
2.799
-0.000
34
trans:cis = 9:1, D1, D2 = 95%, 95%
f1 (ppm)

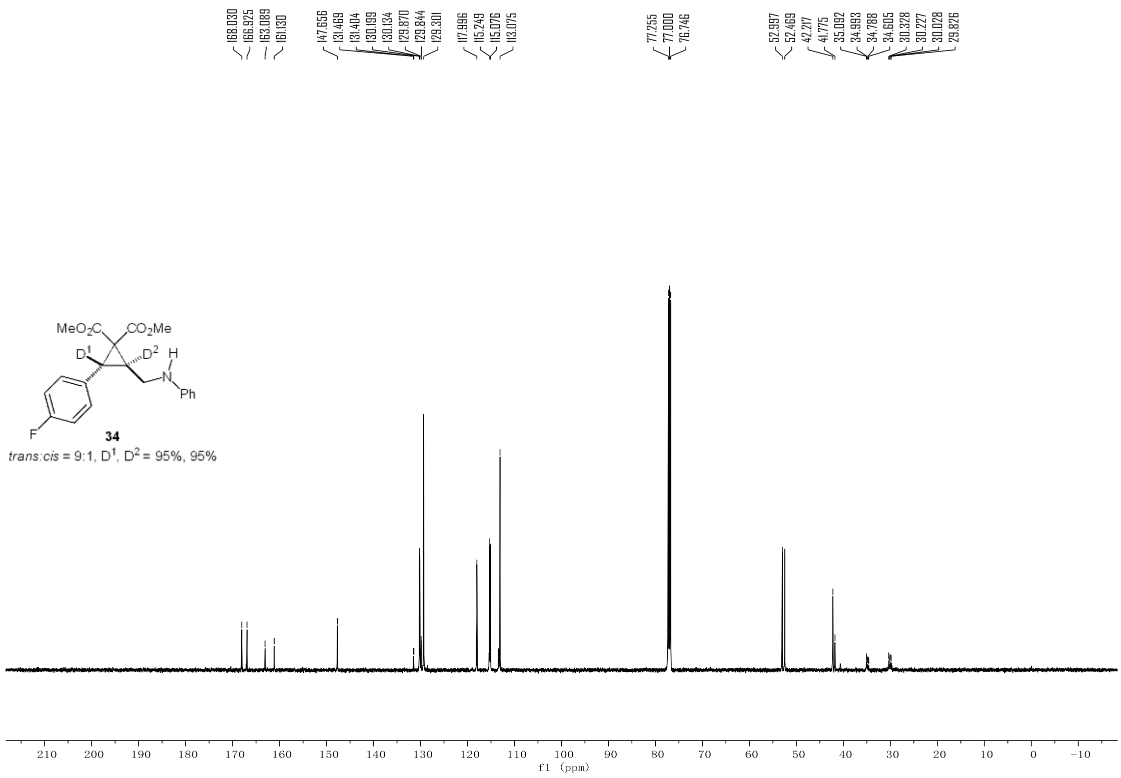
168.030
166.925
163.089
161.130
147.656
131.469
131.404
130.199
130.134
129.870
129.844
129.301
117.996
115.249
115.076
113.075
77.255
77.000
76.746
52.997
52.469
42.217
41.775
35.092
34.993
34.788
34.605
30.328
30.227
30.028
29.826
MeO2C
CO2Me
D1
D2
H
N
Ph
F
34
trans:cis = 9:1, D1, D2 = 95%, 95%
f1 (ppm)

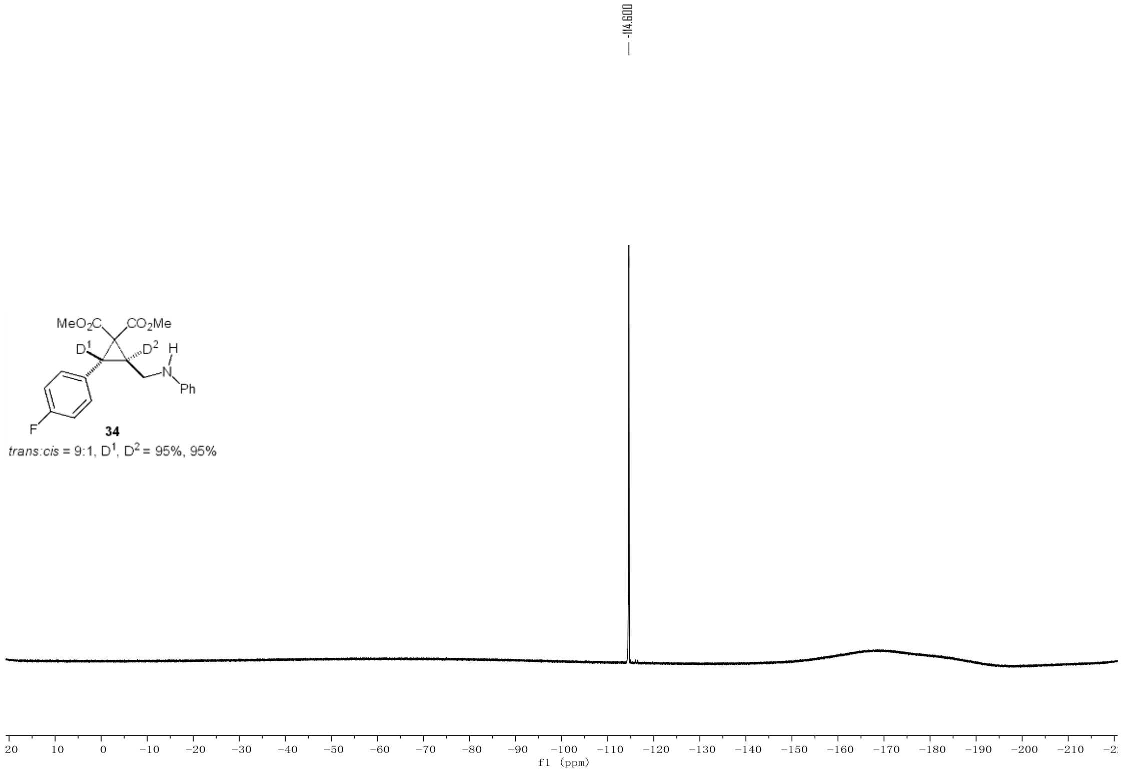
-114.600
MeO2C
CO2Me
D1
D2
H
N
Ph
F
34
trans:cis = 9:1, D1, D2 = 95%, 95%
f1 (ppm)

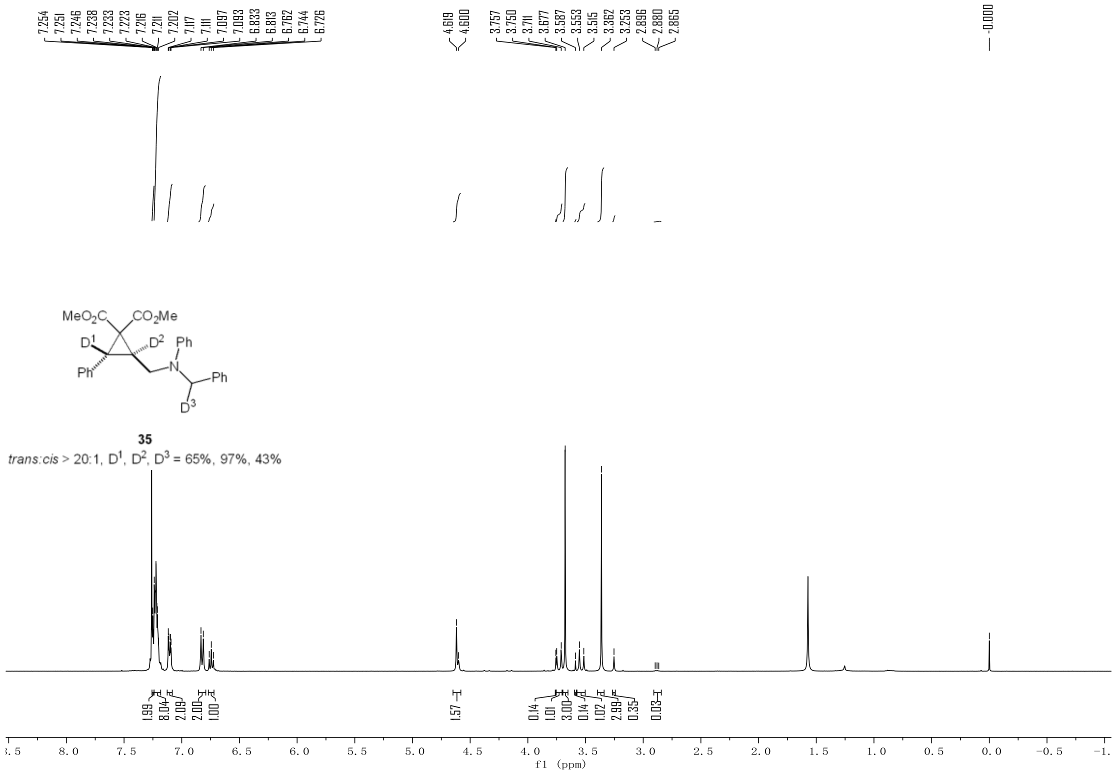
MeO2C CO2Me
D1 D2 Ph
Ph N Ph
D3
35
trans:cis > 20:1, D1, D2, D3 = 65%, 97%, 43%
f1 (ppm)

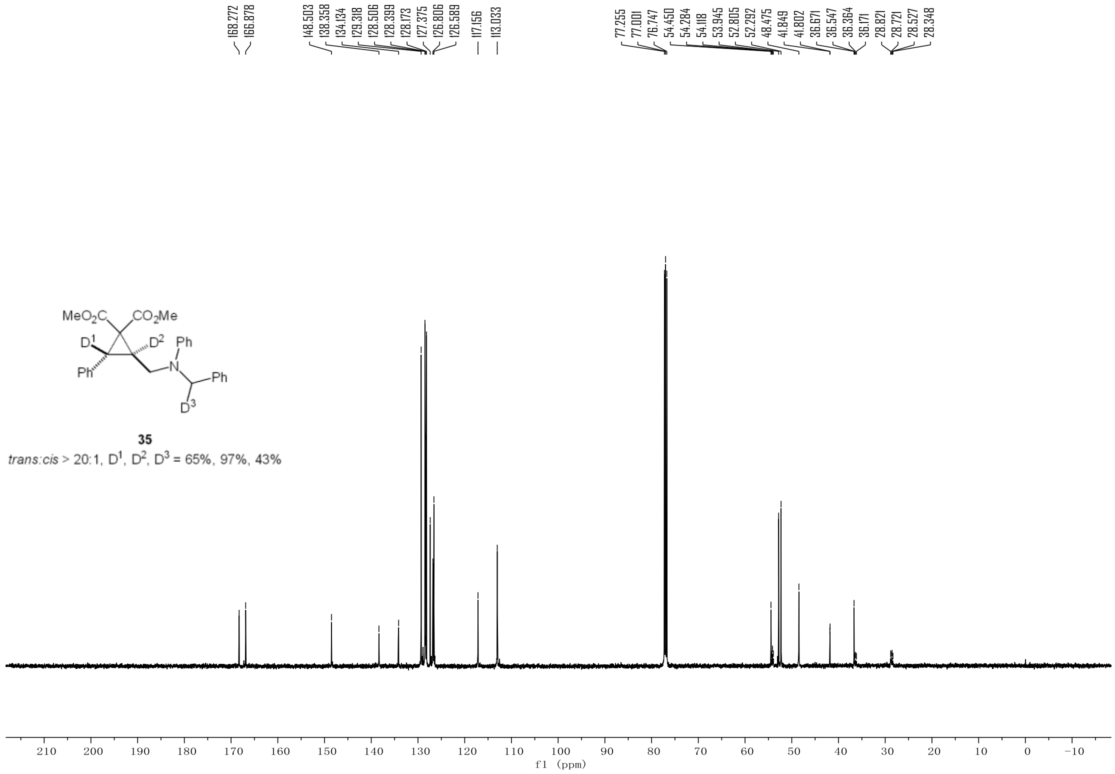
MeO2C CO2Me
D1 D2 Ph
Ph N Ph
D3
35
trans:cis > 20:1, D1, D2, D3 = 65%, 97%, 43%
f1 (ppm)

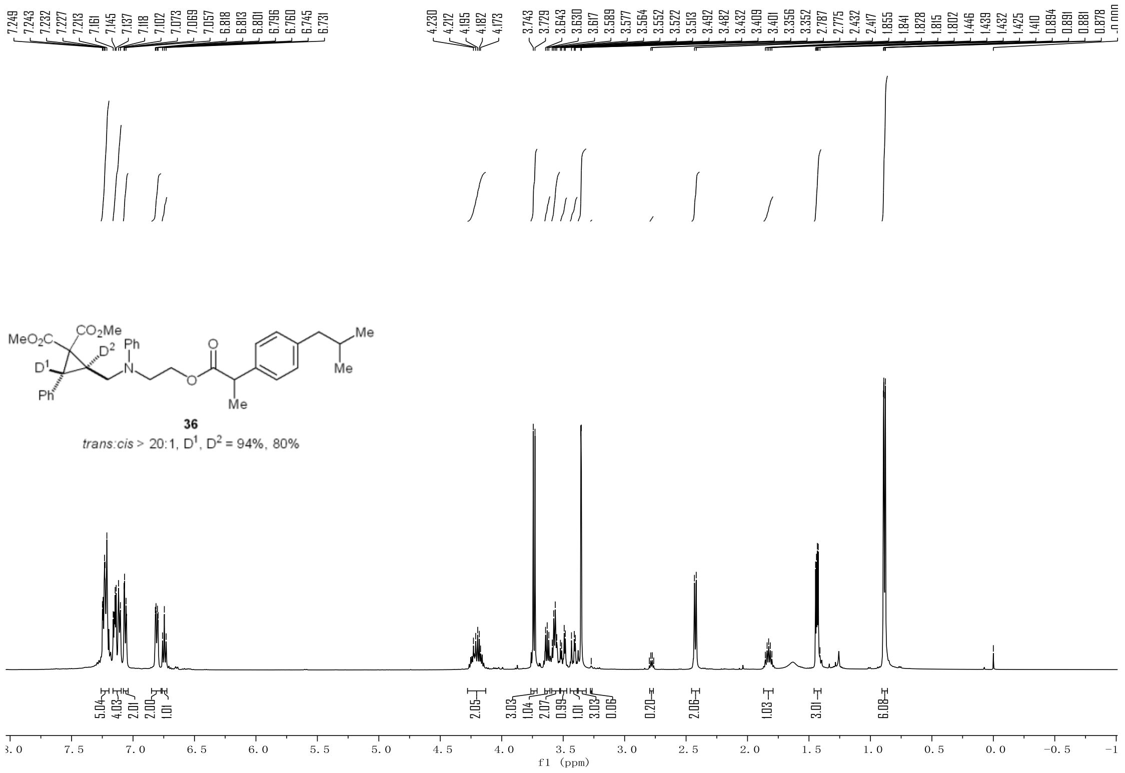

36
trans:cis > 20:1, D1, D2 = 94%, 80%
f1 (ppm)

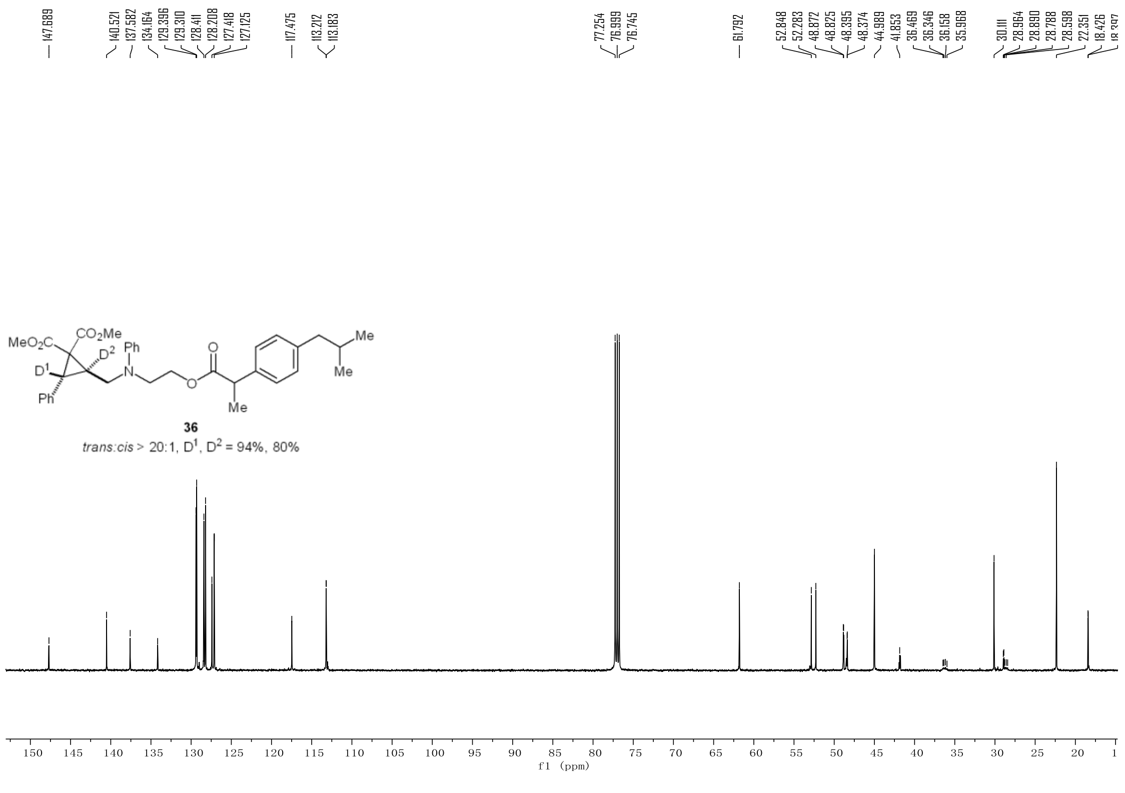

36
trans:cis > 20:1, D1, D2 = 94%, 80%
f1 (ppm)

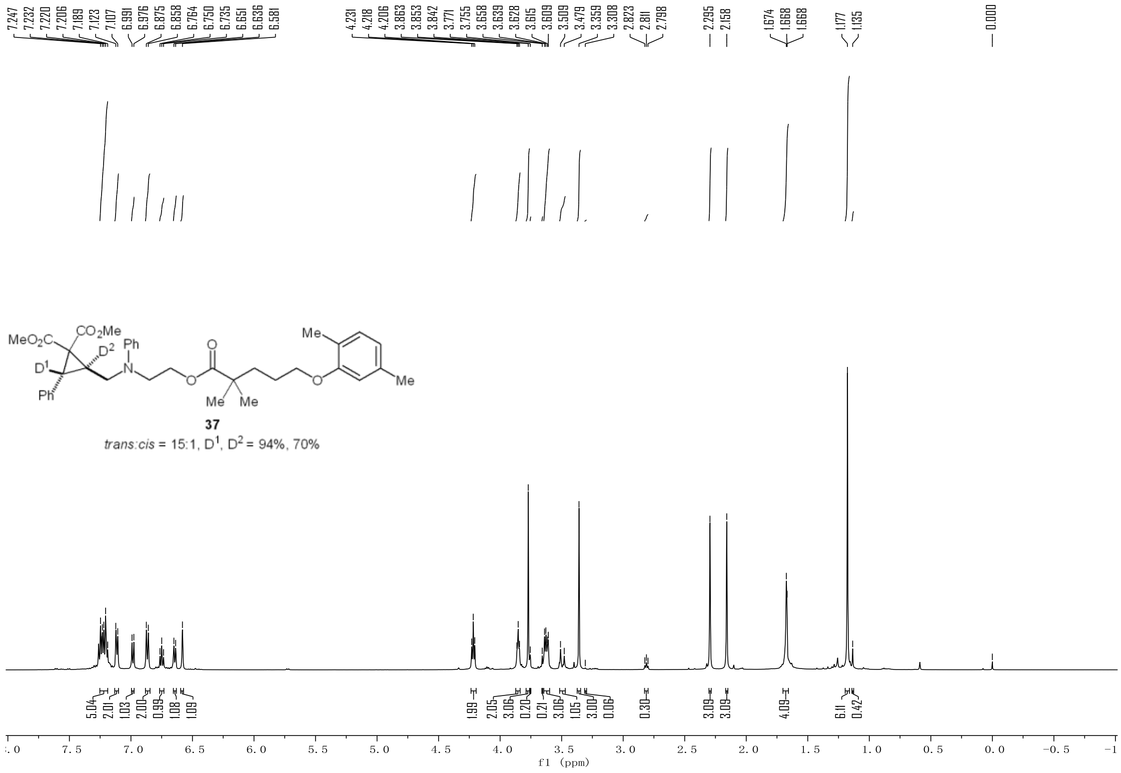
MeO2C
CO2Me
D2
Ph
N
D1
Ph
O
O
Me
Me
Me
O
Me
37
trans:cis = 15:1, D1, D2 = 94%, 70%
f1 (ppm)

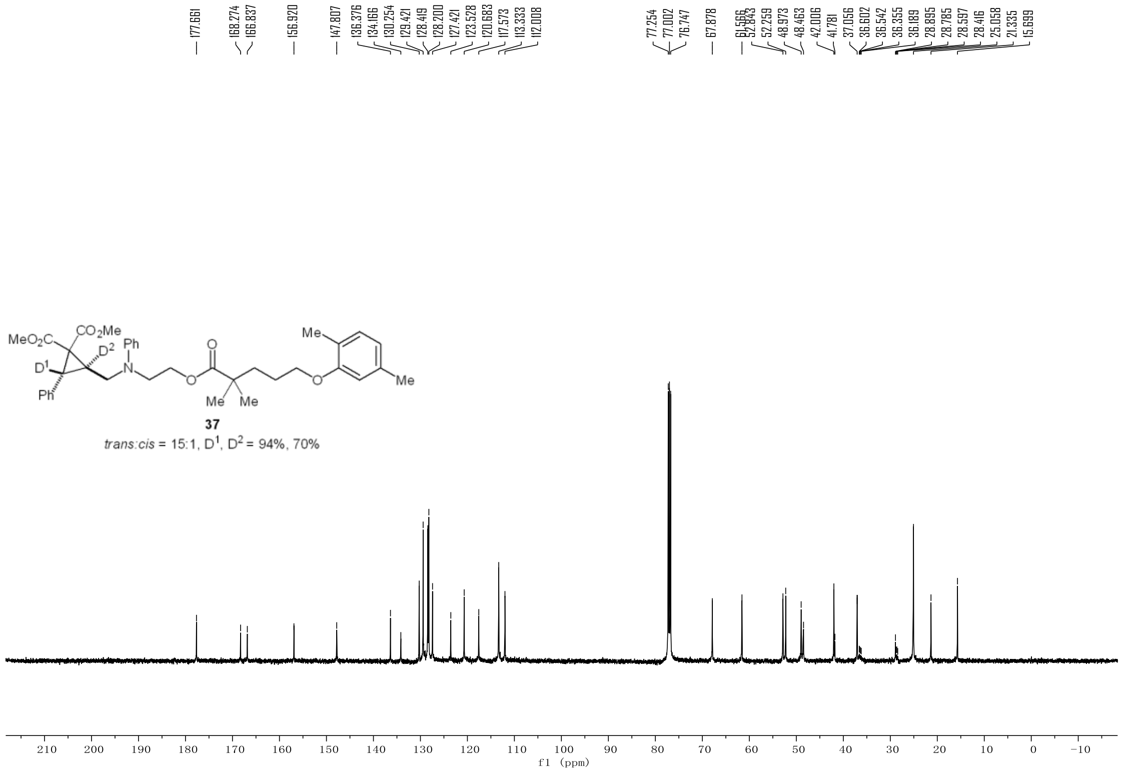
MeO2C
CO2Me
D2
Ph
N
D1
Ph
O
O
Me
Me
Me
O
Me
37
trans:cis = 15:1, D1, D2 = 94%, 70%
f1 (ppm)

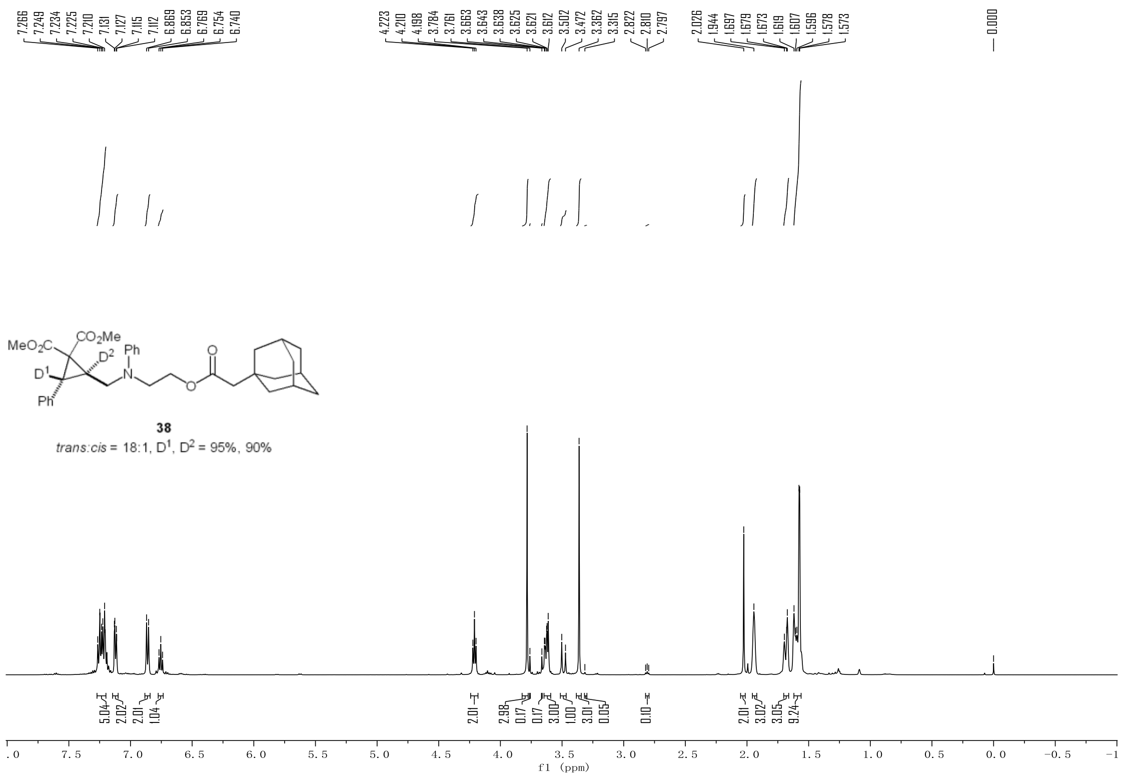
7.266 7.249 7.234 7.225 7.210 7.131 7.127 7.115 7.112 6.869 6.853 6.769 6.754 6.740
4.223 4.210 4.198 3.784 3.761 3.663 3.643 3.638 3.625 3.621 3.612 3.502 3.472 3.362 3.315 2.822 2.810 2.797
2.026 1.944 1.697 1.679 1.673 1.619 1.607 1.596 1.578 1.573
0.000
MeO2C CO2Me Ph D2 D1 N Ph O O
38
trans:cis = 18:1, D1, D2 = 95%, 90%
5.04 2.02 2.01 1.04 2.01 2.98 0.17 0.17 3.00 1.00 3.01 0.05 0.10 2.01 3.02 3.05 9.24
f1 (ppm)

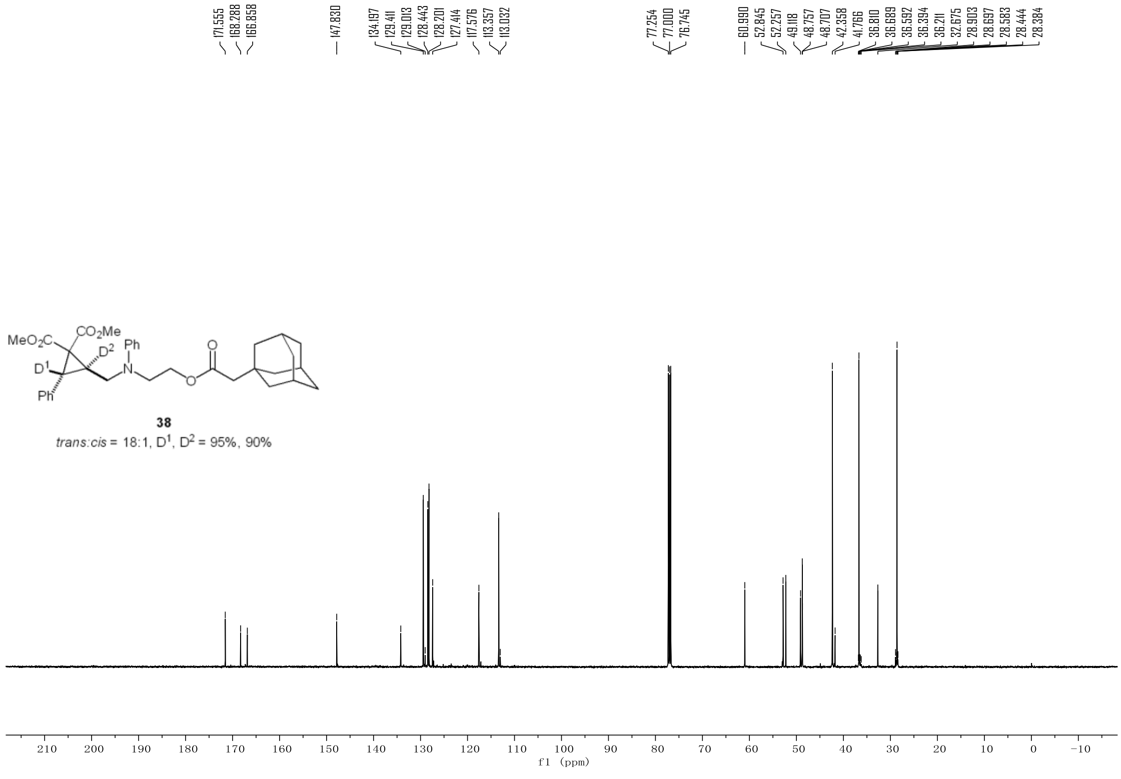
171.555 168.288 166.858
147.830
134.197 129.411 129.013 128.443 128.201 127.414 117.576 113.357 113.032
77.254 77.000 76.745
60.990 52.845 52.257 49.118 48.757 48.707 42.358 41.766 36.810 36.689 36.592 36.394 36.211 32.675 28.903 28.697 28.583 28.444 28.384
MeO2C CO2Me Ph D2 D1 N Ph O O
38
trans:cis = 18:1, D1, D2 = 95%, 90%
f1 (ppm)

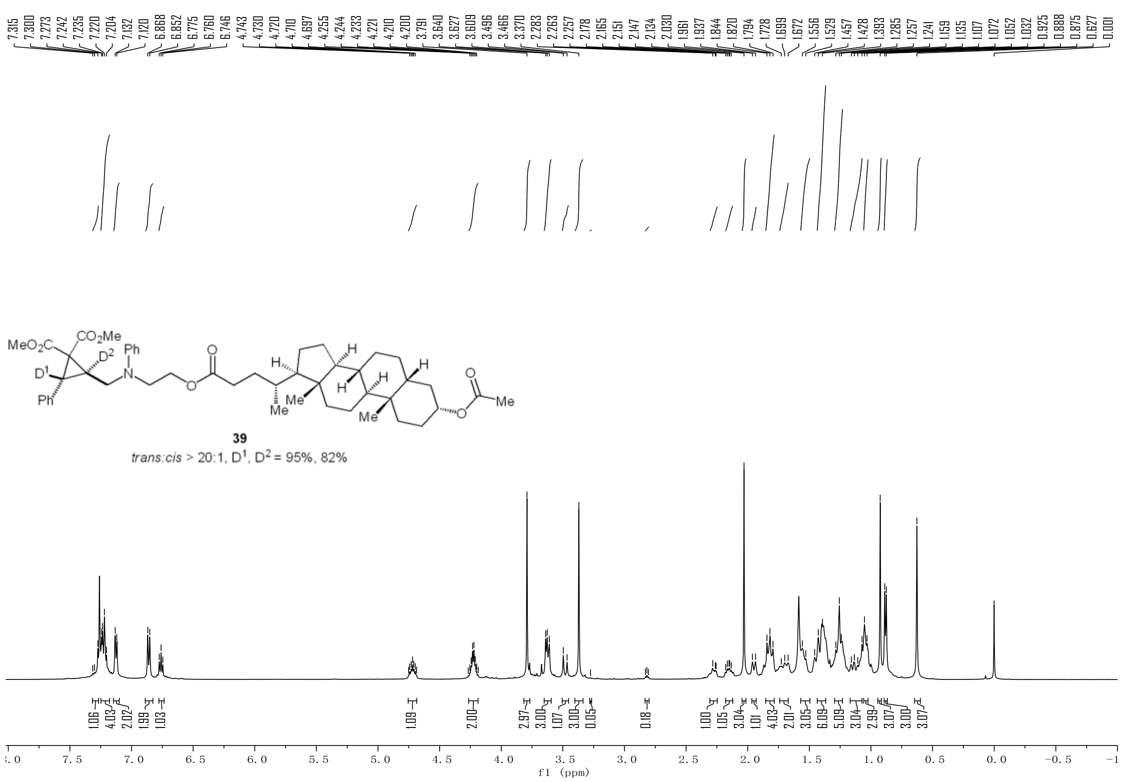
39
trans:cis > 20:1, D1, D2 = 95%, 82%
f1 (ppm)

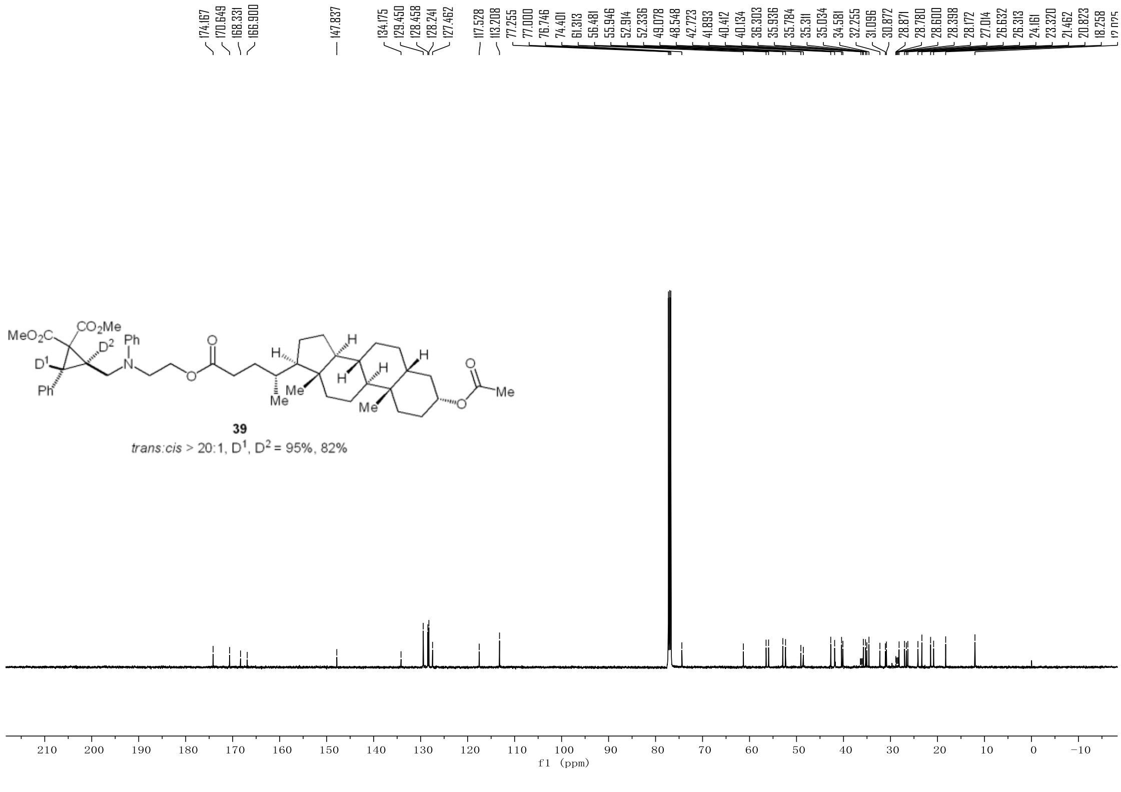
39
trans:cis > 20:1, D1, D2 = 95%, 82%
f1 (ppm)

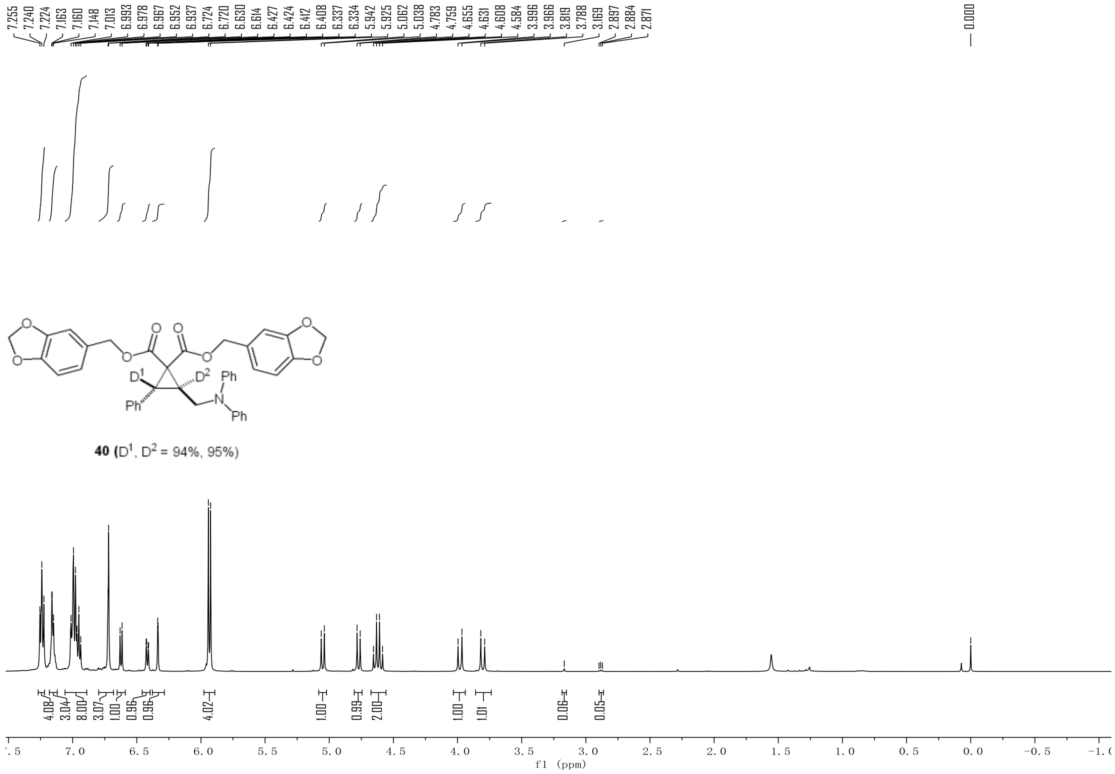

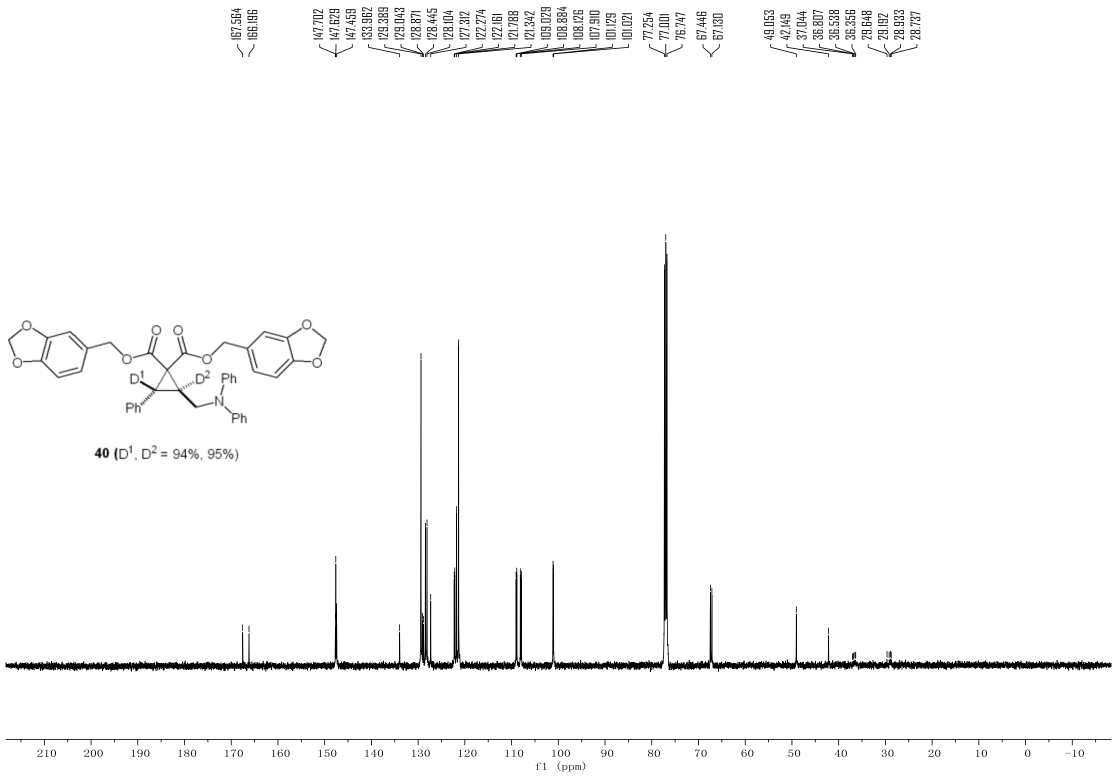

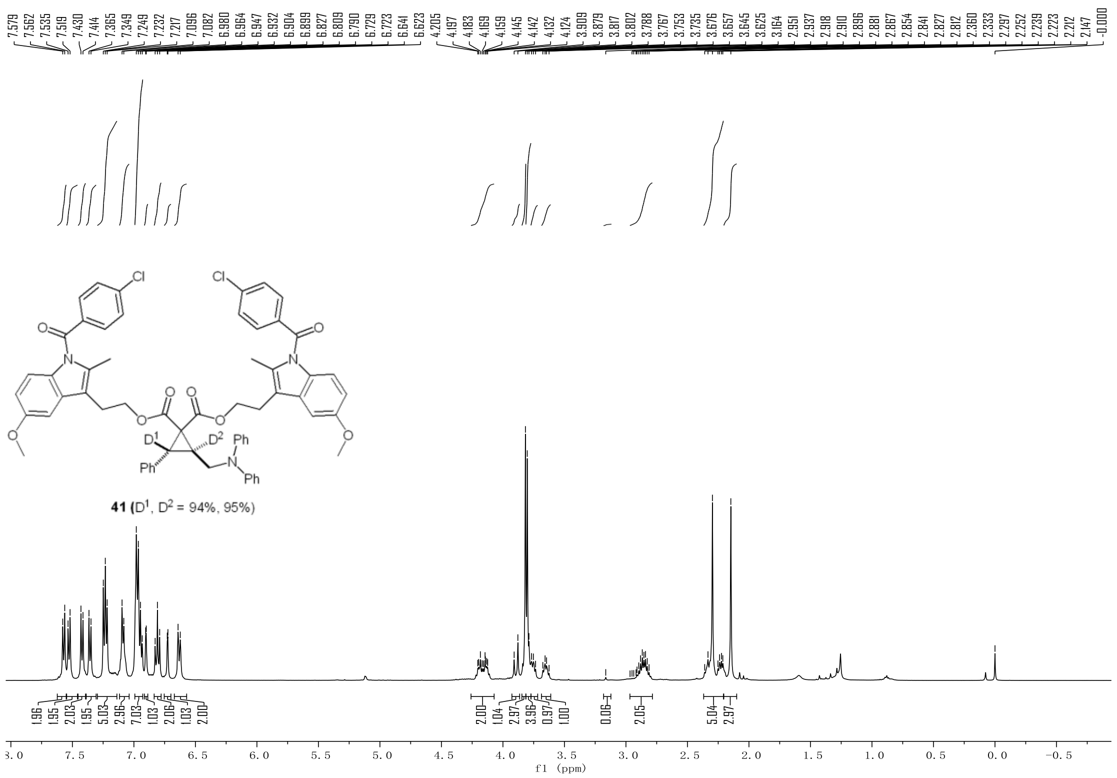
41 (D1, D2 = 94%, 95%)
f1 (ppm)

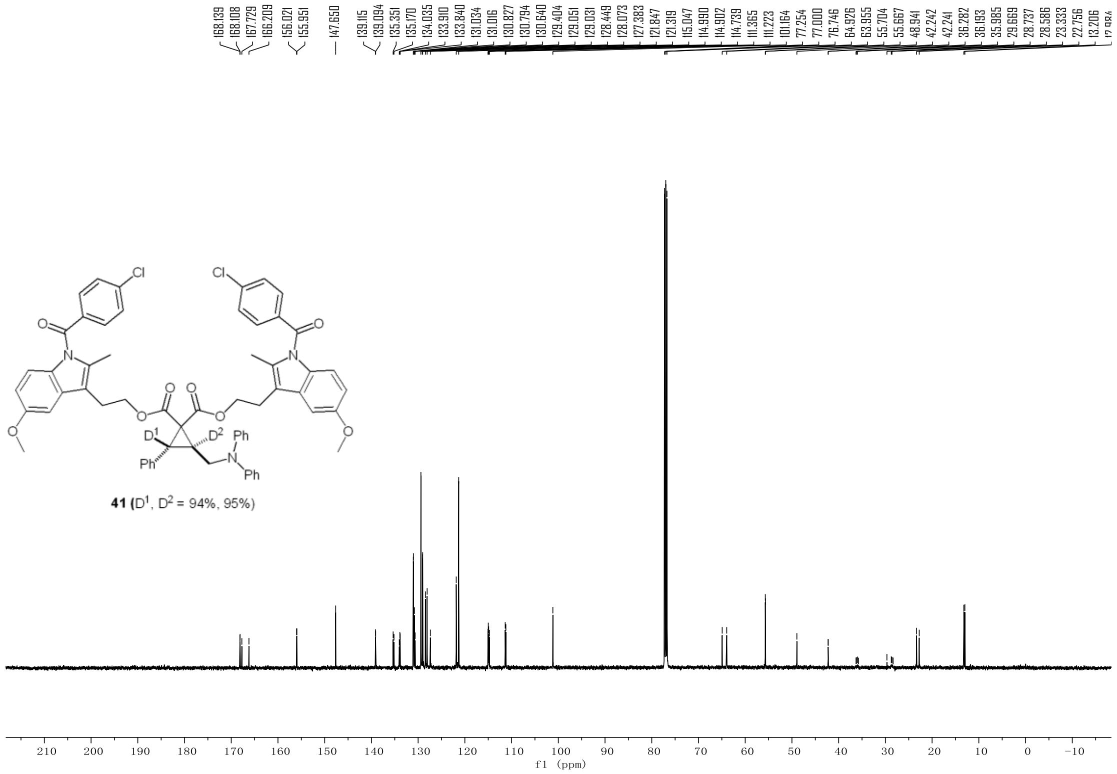
41 (D1, D2 = 94%, 95%)
f1 (ppm)

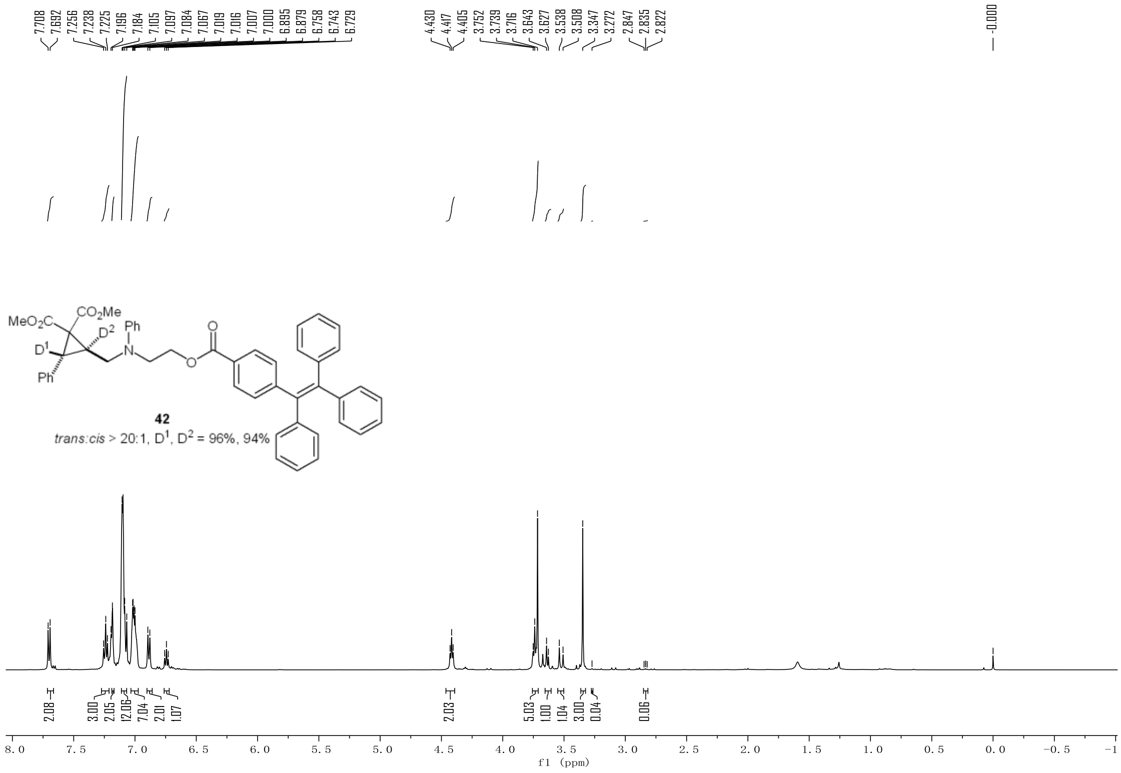
7.708 7.692 7.256 7.238 7.225 7.196 7.184 7.105 7.097 7.084 7.067 7.019 7.016 7.007 7.000 6.895 6.879 6.758 6.743 6.729
4.430 4.417 4.405 3.752 3.739 3.716 3.643 3.627 3.538 3.508 3.347 3.272 2.847 2.835 2.822
-0.000
MeO2C
CO2Me
D2
D1
Ph
N
O
42
trans:cis > 20:1, D1, D2 = 96%, 94%
2.08 3.00 2.05 12.06 7.04 2.01 1.07 2.03 5.03 1.00 1.04 3.00 0.04 0.06
f1 (ppm)

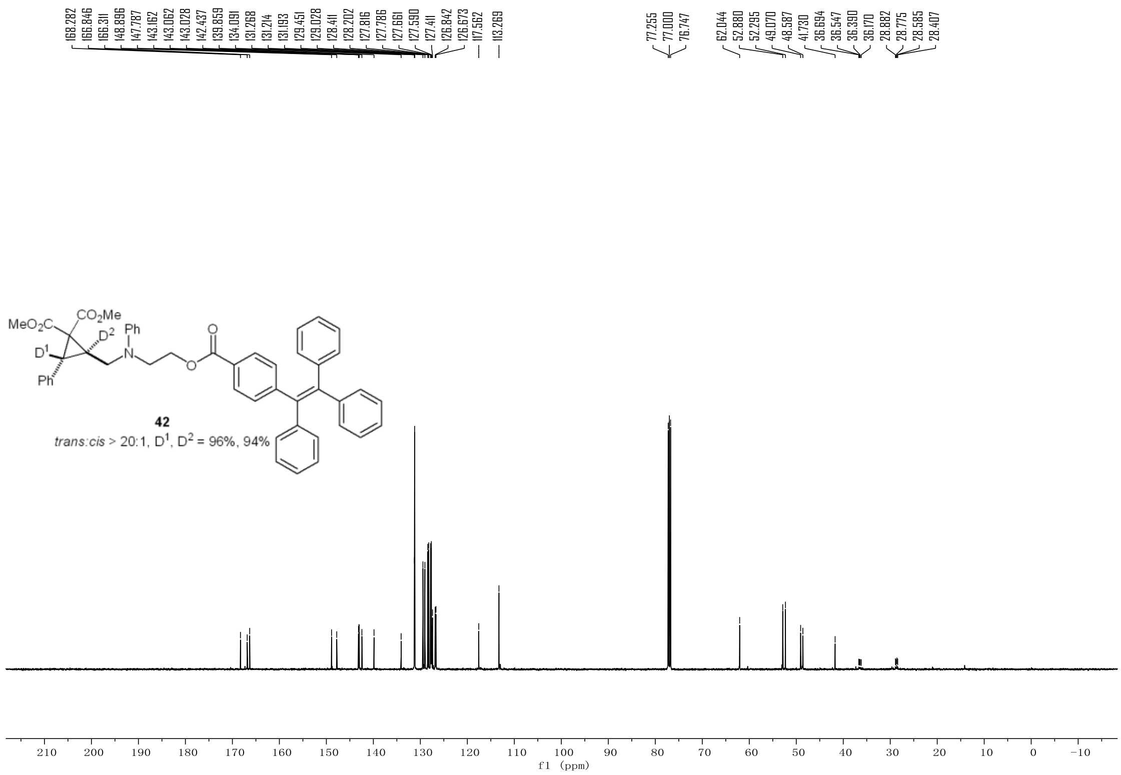
168.282 166.846 166.311 148.896 147.787 143.162 143.062 143.028 142.437 139.859 134.091 131.268 131.214 131.193 129.451 129.028 128.411 128.202 127.816 127.786 127.661 127.590 127.411 126.842 126.673 117.562 113.269
77.255 77.000 76.747
62.044 52.880 52.295 49.070 48.587 41.730 36.694 36.547 36.390 36.170 28.882 28.775 28.585 28.407
MeO2C
CO2Me
D2
D1
Ph
N
O
42
trans:cis > 20:1, D1, D2 = 96%, 94%
f1 (ppm)

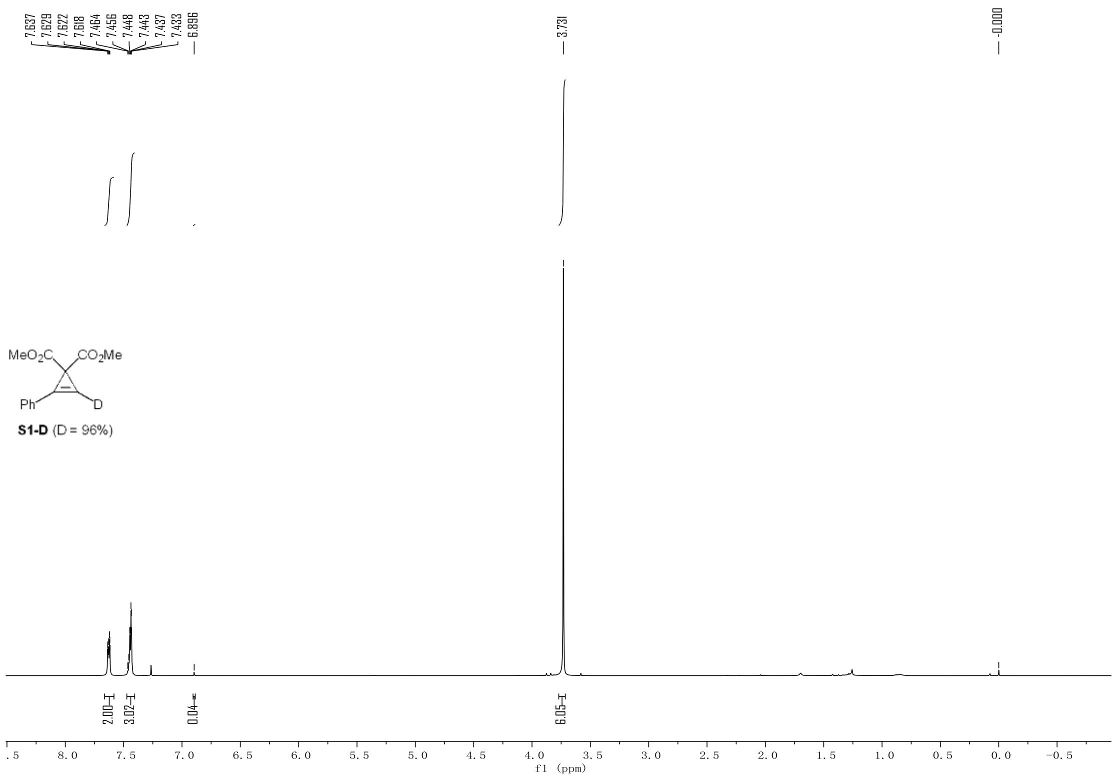
7.637
7.629
7.622
7.618
7.464
7.456
7.448
7.443
7.437
7.433
6.896
3.731
-0.000
MeO2C
CO2Me
Ph
D
S1-D (D = 96%)
2.00
3.02
0.04
6.05
f1 (ppm)

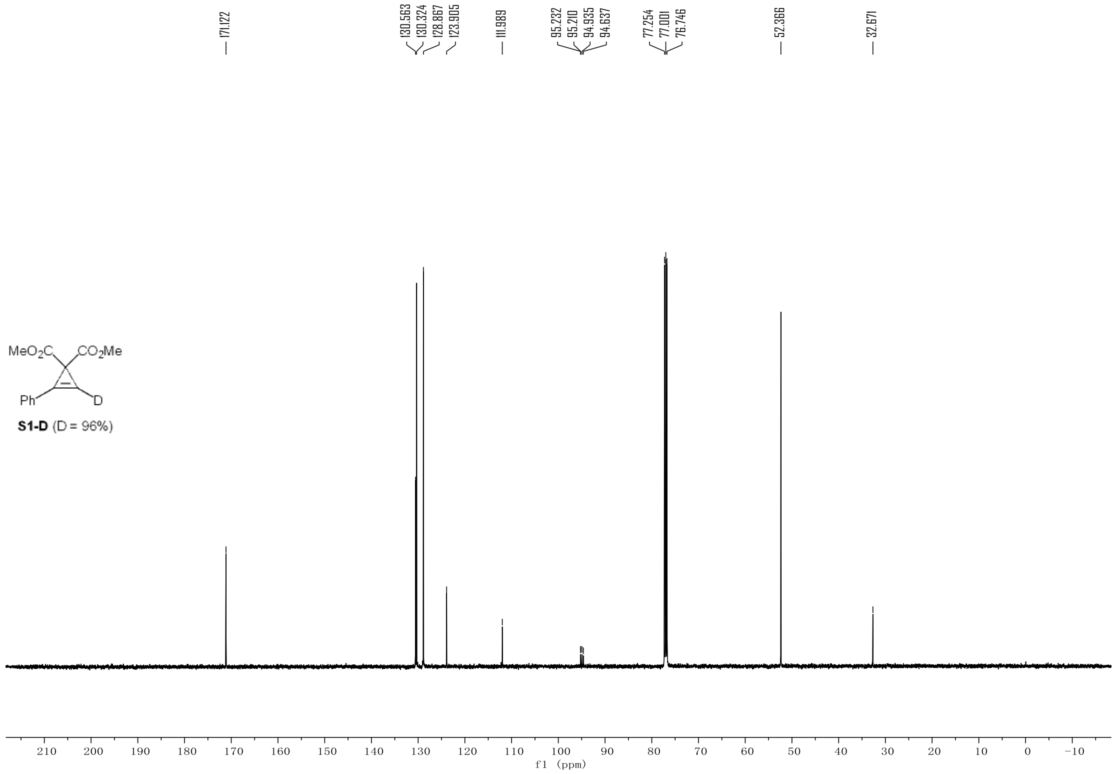
171.122
130.563
130.324
128.867
123.905
111.989
95.232
95.210
94.935
94.637
77.254
77.001
76.746
52.366
32.671
MeO2C
CO2Me
Ph
D
S1-D (D = 96%)
f1 (ppm)
